# Association between oral microbiome and seven types of cancers in East Asian population: a two-sample Mendelian randomization analysis

**DOI:** 10.3389/fmolb.2023.1327893

**Published:** 2023-11-21

**Authors:** Kexin Feng, Fei Ren, Xiang Wang

**Affiliations:** Department of Breast Surgical Oncology, National Cancer Center, National Clinical Research Center for Cancer, Cancer Hospital, Chinese Academy of Medical Sciences and Peking Union Medical College, Beijing, China

**Keywords:** Mendelian randomization, cancers, oral microbiome, case-control study, 16S

## Abstract

**Background:** The oral microbiome has been intricately linked to various pathological conditions, notably cancer, though clear causal links remain elusive. This study aimed to investigate the potential causal relationships between the oral microbiome and seven major cancers: breast, lung, pancreatic, colorectal, gastric, ovarian, and prostate cancers, leveraging Mendelian randomization (MR).

**Methods:** A two-sample MR analysis was conducted using genome-wide association study (GWAS) data specific to oral microbiota in individuals of East Asian descent. Single nucleotide polymorphisms (SNPs) independent of confounders served as instrumental variables (IVs) to deduce causality. MR methodologies such as the inverse variance weighted (IVW) method, weighted median (WM) method, and Mendelian randomization-Egger (MR-Egger) method were employed. The study utilized datasets encapsulating a multitude of cancer cases and controls, focusing on Asian populations.

**Results:** Our analysis revealed intricate associations between specific bacterial genera of the oral microbiome and diverse cancers. Notably, *Fusobacterium* showed mixed associations with various cancers, while genera like Prevotella and *Streptococcus* exhibited nuanced roles across malignancies. The genus Aggregatibacter demonstrated a multifaceted influence, positively correlating with some cancers while inhibiting others.

**Conclusion:** Our findings underscore the profound implications of the oral microbiome in systemic malignancies, suggesting potential modulatory roles in cancer etiology. These insights, though preliminary, accentuate the need for deeper exploration and could pave the way for novel therapeutic strategies.

## 1 Introduction

Globally, cancer incidence and mortality rates are escalating alarmingly, with projections indicating about a 50% surge in the global oncologic burden over the next two decades (Sung et al., 2021). As delineated in the Cancer Statistics 2022 report (Siegel et al., 2022), prostate cancer (PCa), lung cancer (LC), and colorectal cancer (CC) collectively account for an estimated 48% of all new cases among males. In females, cancers of the breast (BC), lung (LC), colon, and rectum comprise 52% of cancer diagnoses. Particularly in China, ovarian cancer (OC) has become the second leading cause of death among gynecological malignancies.

The intricate landscape of the human oral microbiome is a dynamic consortium of microorganisms that play pivotal roles in maintaining oral health and systemic wellness. Over recent decades, advances in metagenomic sequencing have unveiled the profound complexity and diversity of these microbial communities. While the oral microbiome has been historically associated with oral-specific diseases such as periodontitis and dental caries, emerging evidence has spotlighted its potential influence on systemic conditions, notably cancers. Traditionally linked to oral-specific diseases like periodontitis and dental caries, emerging evidence now highlights the potential influence of the oral microbiome on systemic conditions, notably cancers. The complex interactions between oral pathogens and host immunity, coupled with the metabolites produced, have been implicated in tumorigenesis, suggesting a plausible link between oral microbial dysbiosis and cancer progression (Teles et al., 2020). Previous research has demonstrated links between the oral microbiome and several types of cancers, including colorectal cancer (Warren et al., 2013), lung cancer (Vogtmann et al., 2022), and pancreatic cancer (Fan et al., 2018; Herremans et al., 2022). This connection, although nascent, poses profound implications for cancer diagnostics, therapeutics, and prevention strategies. In this context, understanding the intricate interplay between the oral microbiome and carcinogenic pathways emerges as a frontier in oncological research. However, traditional observational studies were impeded by inherent limitations, such as local confounding, and the potential for reverse causality.

Mendelian randomization (MR), based on the principles of Gregor Mendel’s laws of heredity, has become a useful approach in the field. By exploiting the randomness inherent in genetic classification during meiosis, MR transforms genetic variation into a powerful tool for inferring causality, avoiding traditional confounding and reducing the bias inherent in observational studies. The advent of widespread genome-wide association studies (GWAS) has amplified the potential of MR to dissect the complexities of genetic causation, enabling researchers to distinguish between pure correlation and true causation. Furthermore, as the line between genetics and epidemiology continues to blur, MR acts as a beacon, guiding the scientific community to more robust and reliable causal inferences (Bowden and Holmes, 2019; Burgess et al., 2019).

In delving into the research realm of the interrelations between the oral microbiome and various cancers, the shortcomings of existing studies begin to emerge. Initially, compared to the extensive research on gut microbiome, studies on the oral microbiome are relatively scarce. While the gut microbiome has been extensively studied, the exploration of the oral microbiome remains in its nascent stages. This research bias not only limits our understanding of the relationship between the oral microbiome and cancer, but also hinders the discovery of potential preventive and therapeutic methods. Secondly, most existing studies tend to focus on a single type of cancer or 2-3 types of gastrointestinal cancers, lacking a comprehensive understanding of the microbial interactions across different cancer types. This limitation reduces our ability to understand how the oral microbiome functions across different cancer types, thereby restricting the potential to translate research findings into practical applications. Additionally, traditional observational studies usually rely on microbiome sequencing technologies, which, due to technical and sampling limitations, result in significant heterogeneity in research outcomes. For instance, different sequencing platforms and data processing workflows may yield varying results, while the collection, handling, and storage of samples may also affect the composition and diversity analysis of the microbiom. These technical and methodological constraints diminish the repeatability and comparability of research findings, posing challenges for researchers to obtain more accurate, consistent, and interpretable data.

The primary objective of our inquiry is to explore the putative causal relationship between the oral microbiome and a spectrum of predominant malignancies, including breast, lung, pancreatic, colorectal, gastric (GC), prostate, and ovarian cancers. By constructing a theoretical framework underscoring the role of oral microbial communities in oncogenesis, our scholarly endeavors aim to catalyze the development of innovative therapeutic strategies. In the emerging field of microbiome-cancer interconnections, our study serves as a forefront endeavor exploring the diverse associations between the oral microbiome and a broad range of prevalent cancers. Unlike previous studies that often focused on individual cancer types or specific microbial species, our research adopts a comprehensive approach, examining the interplay between the diverse oral microbial communities and various cancer types. This holistic perspective is crucial for unveiling the widespread implications of oral microbial dysbiosis across various oncologic landscapes. Moreover, our study pioneers the use of MR to delineate the causal relationships between the oral microbiome and cancer, overcoming the inherent limitations of traditional observational studies. This innovative methodology not only enhances the robustness of our findings but also advances the scientific discourse towards more precise causal inferences in the microbiome-cancer nexus.

## 2 Materials and methods

### 2.1 Study design and data sources

In this study, the oral microbiota represented the exposure variable, whereas seven different types of cancers were the outcomes. We have taken into consideration the specific genetic and environmental factors present in Asian populations, which may influence the relationship between oral microbiota and cancer. Previous research has demonstrated that the composition of oral microbiota in Asian populations may differ from other ethnic groups, potentially impacting the onset and progression of cancer. Therefore, both our exposure and outcome data are derived from GWAS studies conducted on East Asian populations.

A two-sample MR analysis was conducted using summary statistics derived from a previously published GWAS that investigated the oral microbiota of individuals of East Asian descent. This GWAS, being the first of its kind on a large scale within an East Asian population, targeted 2017 tongue dorsum samples and 1915 salivary samples, employing high-depth whole-genome sequencing. The dataset utilized in this study comprised 309 tongue dorsum microbiomes (*N* = 2,017) and 285 salivary microbiomes (*N* = 1,915) (Liu X. et al., 2021). The samples underwent rigorous inclusion criteria, which involved ensuring a variant calling rate of at least 98%, a mean sequencing depth of over 20×, absence of population stratification in principle component analysis (PCA), and the removal of related individuals based on pairwise identity by descent estimates. In addition, the study used stringent criteria, including a minimum mean depth of 8×, Hardy-Weinberg equilibrium (HWE) values over 10–5, and a genotype calling rate higher than 98% for the analyzed variations. Following stringent quality control protocols, a comprehensive cohort of 2,984 participants was assembled, consisting of 2,017 individuals with tongue dorsum samples and 1,915 persons with salivary samples. Subsequently, a dataset comprising about 10 million variants, encompassing both common and low-frequency variants with a minor allele frequency (MAF) of at least 0.5%, was maintained for further analysis. For a more comprehensive understanding of the methods employed in this study, including sample collection, sequencing techniques, creation of microbiome traits, and observational and genotyping studies, readers are referred to the work of Liu X. et al. (2021).

The data included in this investigation for the sevencancers originated from a comprehensive GWAS performed on an Asian population. This is a large-scale GWAS conducted on an East Asian population for 27 diseases (Ishigaki et al., 2020). The study included a total of 5552 cases and 89731 controls of BC; 6563 cases and 195745 controls of GC, 442 cases and 195745 cotrols of PC, 4050 cases and 208403 controls of LC; 720 cases and 89731 controls of OC, 5408 cases and 103903 controls of PCa; 7062 cases and 195745 controls of CC (Ishigaki et al., 2020). The summary of the GWAS included in this MR study was in Table 1.

**TABLE 1:** Summary of the GWAS included in this Mendelian randomization study.

Exposures/outcomes	Consortium	Ethnicity	Sample sizes	N. SNPs	Year
Oral microbiome	CNGBdb	East Asian	2948	Tongue *N* = 8426	2021
			Tongue *N* = 2017	Saliva *N* = 8009	
			Saliva *N* = 1914		
Breast cancer	NBDC Human Database	East Asian	*N* = 95283	*N* = 8919992	2020
Lung cancer	NBDC Human Database	East Asian	*N* = 212453	*N* = 8885805	2020
Pancreatic cancer	NBDC Human Database	East Asian	*N* = 196187	*N* = 8885075	2020
Colorectal cancer	NBDC Human Database	East Asian	*N* = 202807	*N* = 8885369	2020
Gastric cancer	NBDC Human Database	East Asian	*N* = 202308	*N* = 8885324	2020
Prostate cancer	NBDC Human Database	East Asian	*N* = 109347	*N* = 8878753	2020
Ovarian cancer	NBDC Human Database	East Asian	*N* = 90451	*N* = 8876088	2020

GWAS, genome-wide association studies; SNPs, single nucleotide polymorphisms; IVs, instrumental variables; CNGBdb, China National GeneBank DataBase; NBDC, national bioscience database centre.

### 2.2 Selection of genetic instrumental variables

In MR, single nucleotide polymorphisms (SNPs) are harnessed as instrumental variables (IVs) to attenuate reverse causation and a litany of inaccuracies endemic to observational epidemiological analyses. For the purpose of to validate the initial assumption, SNPs were initially chosen based on a genome-wide significance threshold of *p* < 5 × 10^−8^. Nevertheless, given the limited number of SNPs that exhibited an association with the oral microbiome at this particular threshold, a less stringent criterion of *p* < 5 × 10^−6^ was employed. In order to account for potential linkage disequilibrium (LD) among the chosen SNPs, a clumping technique was performed. This procedure involved utilizing a window size of 10,000 kilobases and setting a threshold of an R2 value less than 0.001, as outlined by Abecasis et al. (2010) and Purcell et al. (2007). In addition, in order to maintain allele consistency, the exposure and outcome datasets underwent a process of harmonization. This involved removing SNPs with non-concordant alleles and SNPs with intermediate allele frequencies, hence reducing ambiguity. The SNPs that were selected with great care were afterwards employed as the definitive genetic IVs for the subsequent MR analysis. Furthermore, the F statistics were computed for each SNP both individually and cumulatively. The calculation was performed using the formula: F = R2 * (N–2)/(1–R2), where R2 denotes the proportion of the variance in the exposure variable that is accounted for by each IV. In the study conducted by Burgess et al. (2018) and Burgess et al. (2011), IVs with F statistics below ten were deemed to be poor instruments and were consequently removed from the MR analysis.

### 2.3 MR analysis

Various statistical methodologies were utilized to examine the causal relationship between breast cancer and the oral microbiome. These methodologies encompassed the inverse variance weighted (IVW) method (Burgess and Thompson, 2015), the Simple mode, Weighted mode, weighted median (WM) method (Bowden et al., 2016), and the Mendelian randomization-Egger (MR-Egger) method (Burgess and Thompson, 2017). The IVW method is a widely utilized technique, most effective when all IVs adhere to the fundamental assumptions of MR: absence of horizontal pleiotropy and unbiased estimations. Alternatively, the WM method, by computing the median of all instrumental variable effect estimates, emerges as a commendable choice when some instrumental variables do not satisfy MR assumptions, such as exhibiting horizontal pleiotropy. The MR-Egger method, besides estimating causal effects, detects and corrects for horizontal pleiotropy, proving valuable when horizontal pleiotropy is suspected (Burgess and Thompson, 2015). Associations between variables were deemed significant if the resulting *p*-value of IVW method was less than 0.05 with the estimate direction of other four MR methods were consistent with IVW.

### 2.4 Sensitivity analysis

Multiple tests were employed, encompassing the heterogeneity test, pleiotropy test, and leave-one-out sensitivity test. The Cochrane’s Q test was employed to evaluate the comprehensive pleiotropy in the IVW MR findings as *p*-value <0.05 implying the presence of heterogeneity. The determination of the average horizontal pleiotropy of the IVs in MR-Egger regression was based on the intercept term and the evaluation of funnel plot asymmetry (Hemani et al., 2018). The existence of heterogeneity was considered significant if the significance level was below *p* < 0.05. Furthermore, the MR-PRESSO approach was employed to assess the existence of pleiotropy and address the issue of horizontal pleiotropy through the identification and exclusion of probable outliers. Following this, we conducted leave-one-out assessment to determine if significant alterations in the causal effects were observed both before and after the elimination of outliers (Verbanck et al., 2018).

The study provides estimates of effect sizes or odds ratios (ORs) together with their respective 95% confidence intervals (CIs). The statistical tests employed in this study were conducted using a two-sided approach. The studies were conducted using the open-source statistical software R (version: 4.2.2). The analyses were mostly conducted using the TwoSampleMR program (version: 0.5.6) (Verbanck et al., 2018) and MR-PRESSO (version 1.0).

## 3 Results

### 3.1 Instrumental variables selection

**FIGURE 1:**
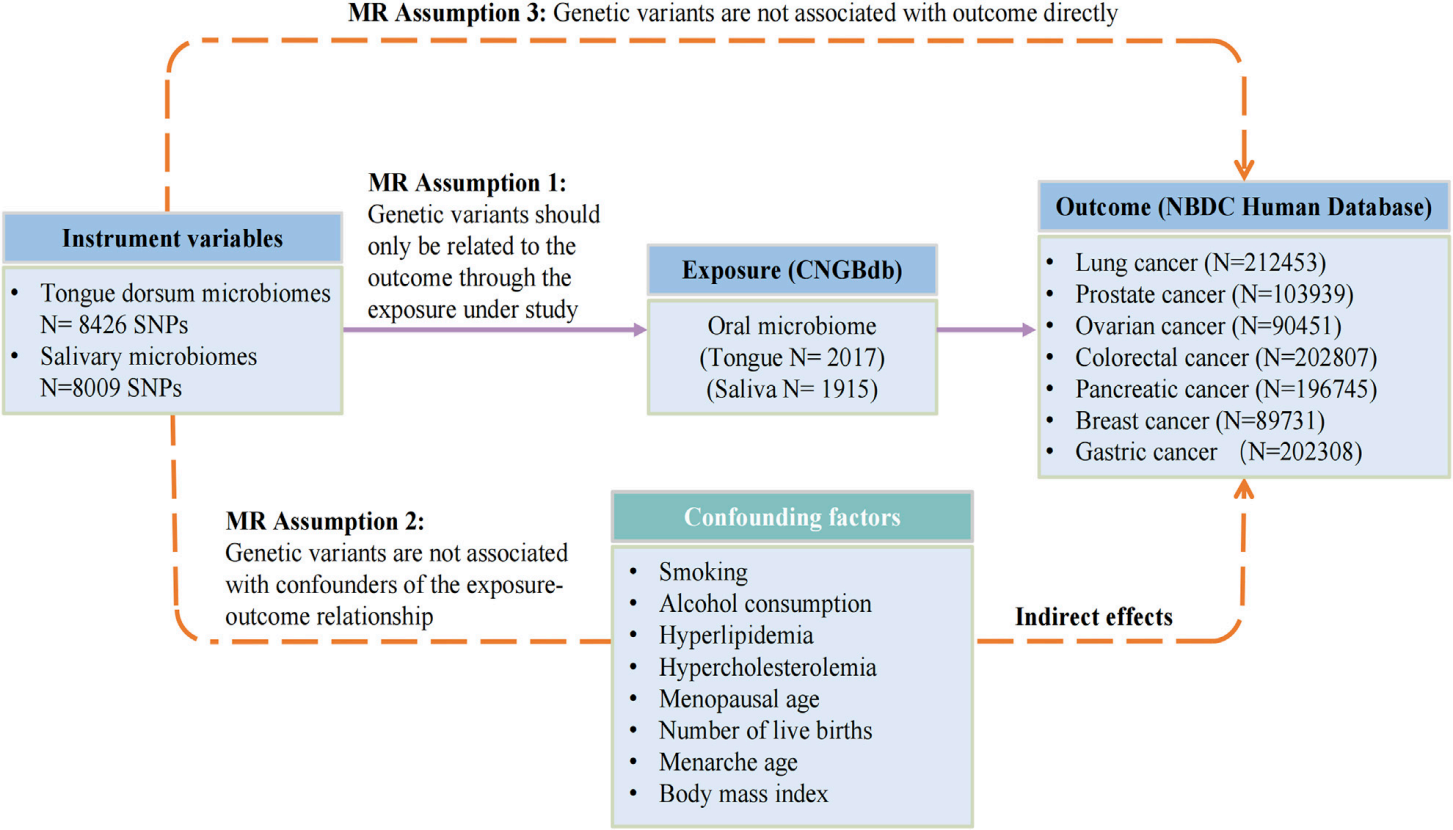
The flowchart of Mendelian randomization analysis. IVW, inverse variance weighted; MR, Mendelian randomization; MVMR, multivariable Mendelian randomization; SNPs, single nucleotide polymorphisms; IVs, instrumental variables; CNGBdb, China National GeneBank DataBase; BBJ, BioBank Japan Project.

The flowchart depicted in Figure 1 provides an overview of the MR analysis procedure. Following the removal of SNPs influenced by linkage disequilibrium and palindrome structure, a combined count of 8009 SNPs linked to salivary microbiomes and 8426 SNPs linked to tongue microbiomes were retained for subsequent analysis, utilizing a suggestive significance threshold of *p* < 5.0 × 10^−6^. The SNPs included in this study represent species. The F-statistics of the instrumental variables varied between 20.01 and 32.44, all of which were statistically significant and exceeded the threshold of 10. These results suggest that there is no indication of weak instrument bias. The statistical analysis conducted by Cochran’s Q test revealed no statistically significant heterogeneity (*p* > 0.05). The P- values of MR-PRESSO and the MR-Egger regression were all over 0.05, indicating no horizontal pleiotropy among the chosen SNPs. The leave-one-out sensitivity analysis revealed that individual SNPs did not exhibit a dominant influence on the overall evaluation. Detailed data regarding the MR results of specific tongue microflora at genus level related to seven cancers can be found in the Supplementary Table S1, and results of saliva microflora were in Supplementary Table S2.

### 3.2 Causal effects of oral microbiota on the development of seven cancer types

#### 3.2.1 Breast cancer

**FIGURE 2:**
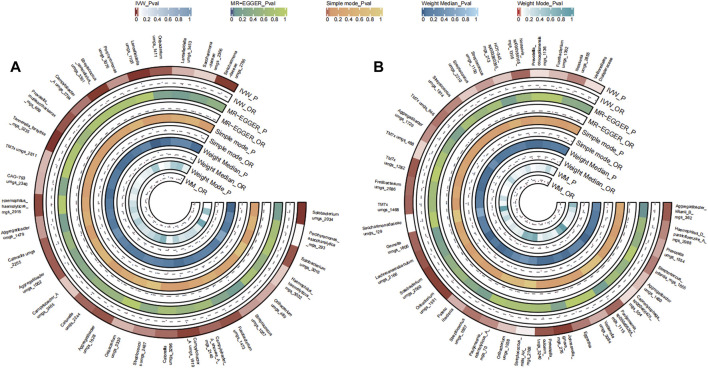
Circular Heatmap of Mendelian randomization results of oral microflora at species level and breast cancer **(A)**, tongue, **(B)** saliva.

A total of 31 bacterial species in the tongue (16 genera and 12 family) and 36 bacterial species in saliva (19 genera and 16 family) had statistically significant relationships with BC. Seven genera were shared by both the tongue and saliva, including Aggregatibacter, *Fusobacterium*, *Streptococcus* and Saccharimonadaceae TM7x (OR >1), Prevotella, Oribacterium, and Solobacterium (OR <1). The causal effects of tongue and saliva bacterial species on breast cancer were shown in Figures 2A, B.

#### 3.2.2 Lung cancer

**FIGURE 3:**
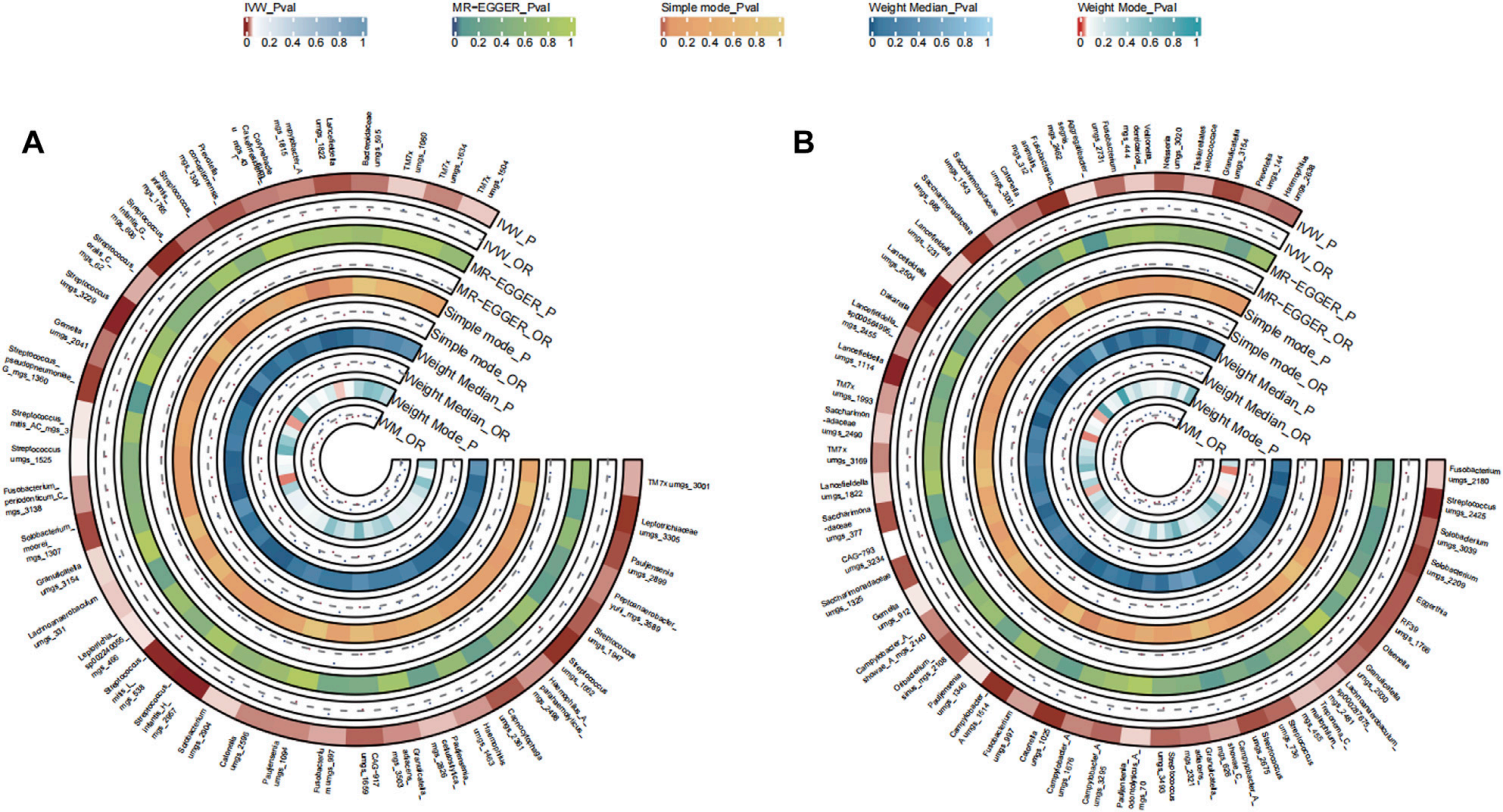
Circular Heatmap of Mendelian randomization results of oral microflora at species level and lung cancer **(A)**, tongue, **(B)** saliva.

A total of 32 bacterial species in the tongue (11 genus and 11 family) and 37 bacterial species in saliva (18 genus and 24 family) had statistically significant relationships with LC. For LC, six genera manifested in both tongue and saliva samples. Among them, Aggregatibacter and Gemella were positively correlated (OR >1), whereas *Fusobacterium*, *Streptococcus*, *Campylobacter* A, and Saccharimonadaceae TM7x were negatively associated with disease progression (OR <1). The causal effects of tongue and saliva bacterial species on lung cancer were shown in Figures 3A, B.

#### 3.2.3 Pancreatic cancer

**FIGURE 4:**
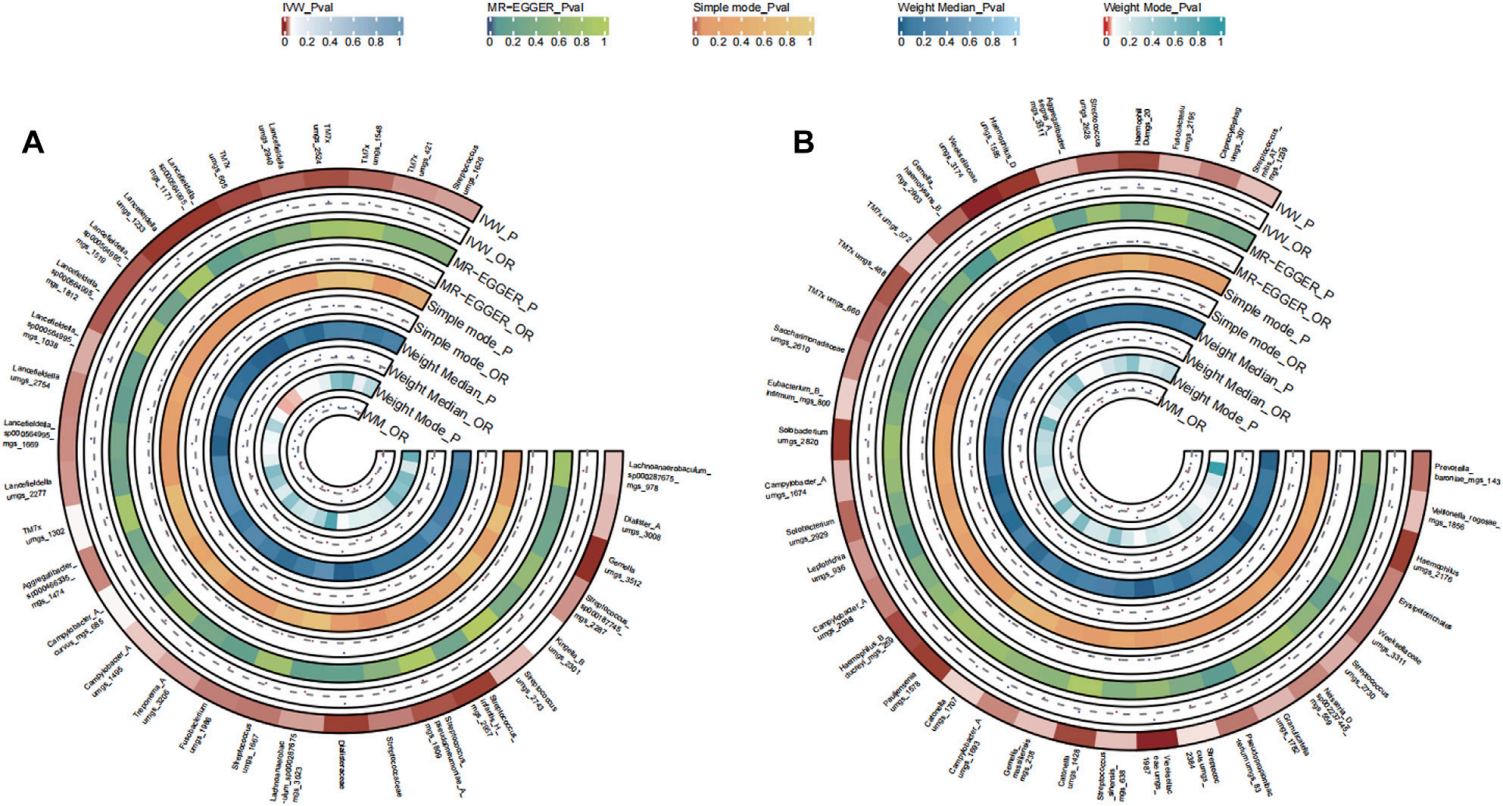
Circular Heatmap of Mendelian randomization results of oral microflora at species level and pancreatic cancer **(A)**, tongue, **(B)** saliva.

A total of 48 bacterial species in the tongue (26 genus and 18 family) and 51 bacterial species in saliva (29 genus and 21 family) had statistically significant relationships with PC. For PC, a total of ten genera were present across both sample sources. *Fusobacterium* and Veillonellaceae F0422 emerged as potential risk factors (OR >1), while genera such as Prevotella, Oribacterium, Aggregatibacter, Solobacterium, Pauljensenia, *Streptococcus*, Gemella, Porphyromonas, Saccharimonadaceae TM7x, and Lancefieldella might confer protective benefits (OR <1). The causal effects of tongue and saliva bacterial species on pancreatic cancer were shown in Figures 4A, B.

#### 3.2.4 Colorectal cancer

**FIGURE 5:**
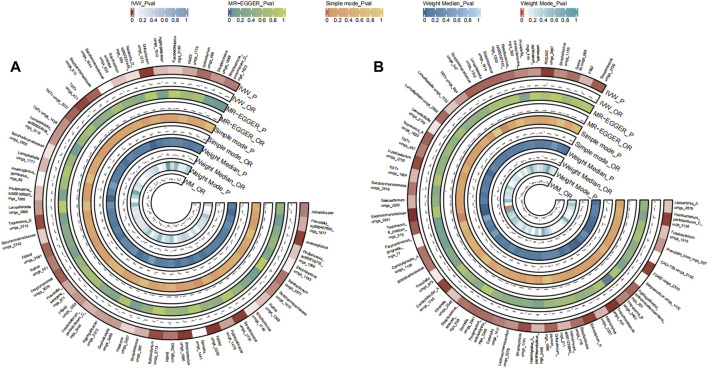
Circular Heatmap of Mendelian randomization results of oral microflora at species level and colorectal cancer **(A)**, tongue, **(B)** saliva.

A total of 39 bacterial species in the tongue (21 genera and 17 family) and 49 bacterial species in saliva (20 genera and 27 family) had statistically significant relationships with CC. Thirteen genera were shared by both the tongue and saliva, including Pauljensenia, *Fusobacterium*, Catonella, *Campylobacter*_A, *Haemophilus*, Granulicatella, Saccharimonadaceae TM7x (OR >1), Prevotella, Solobacterium, *Streptococcus*, Gemella, Lachnoanaerobaculum, Lancefieldella (OR <1). The causal effects of tongue and saliva bacterial species on colorectal cancer were shown in Figures 5A, B.

#### 3.2.5 Gastric cancer

**FIGURE 6:**
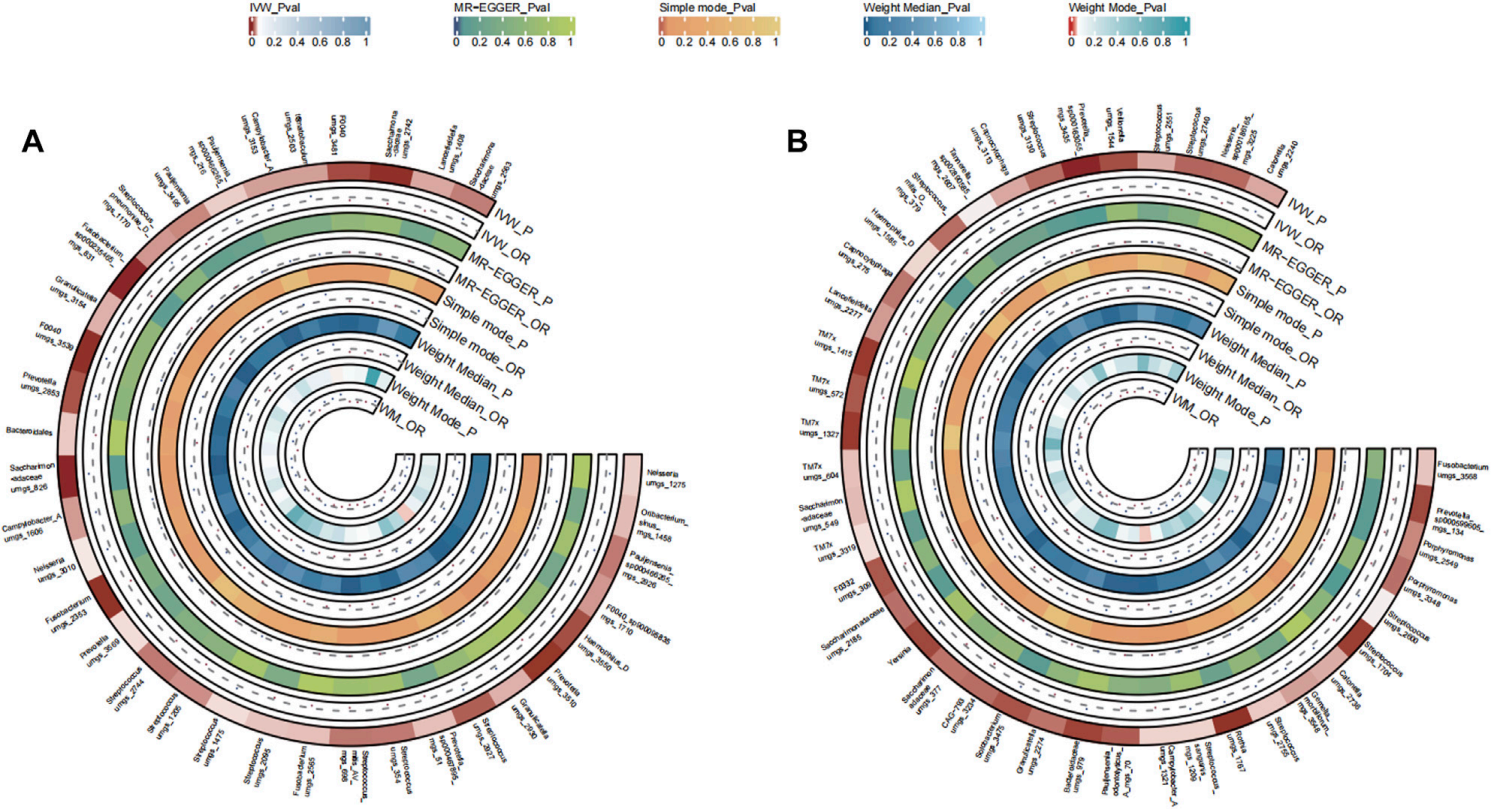
Circular Heatmap of Mendelian randomization results of oral microflora at species level and gastric cancer **(A)**, tongue, **(B)** saliva.

A total of 35 bacterial species in the tongue (12 genera and 15 family) and 40 bacterial species in saliva (25 genera and 20 family) had statistically significant relationships with GC. Seven genera were identified both in the tongue and saliva samples associated with gastric cancer. Notably, *Neisseria*, *Fusobacterium*, *Haemophilus* D, and Granulicatella were observed to potentially promote the disease (OR >1), while Prevotella, Pauljensenia, *Streptococcus*, *Campylobacter* A, and Lancefieldella might exhibit protective roles (OR <1). The causal effects of tongue and saliva bacterial species on gastric cancer were shown in Figures 6A, B.

#### 3.2.6 Prostate cancer

**FIGURE 7:**
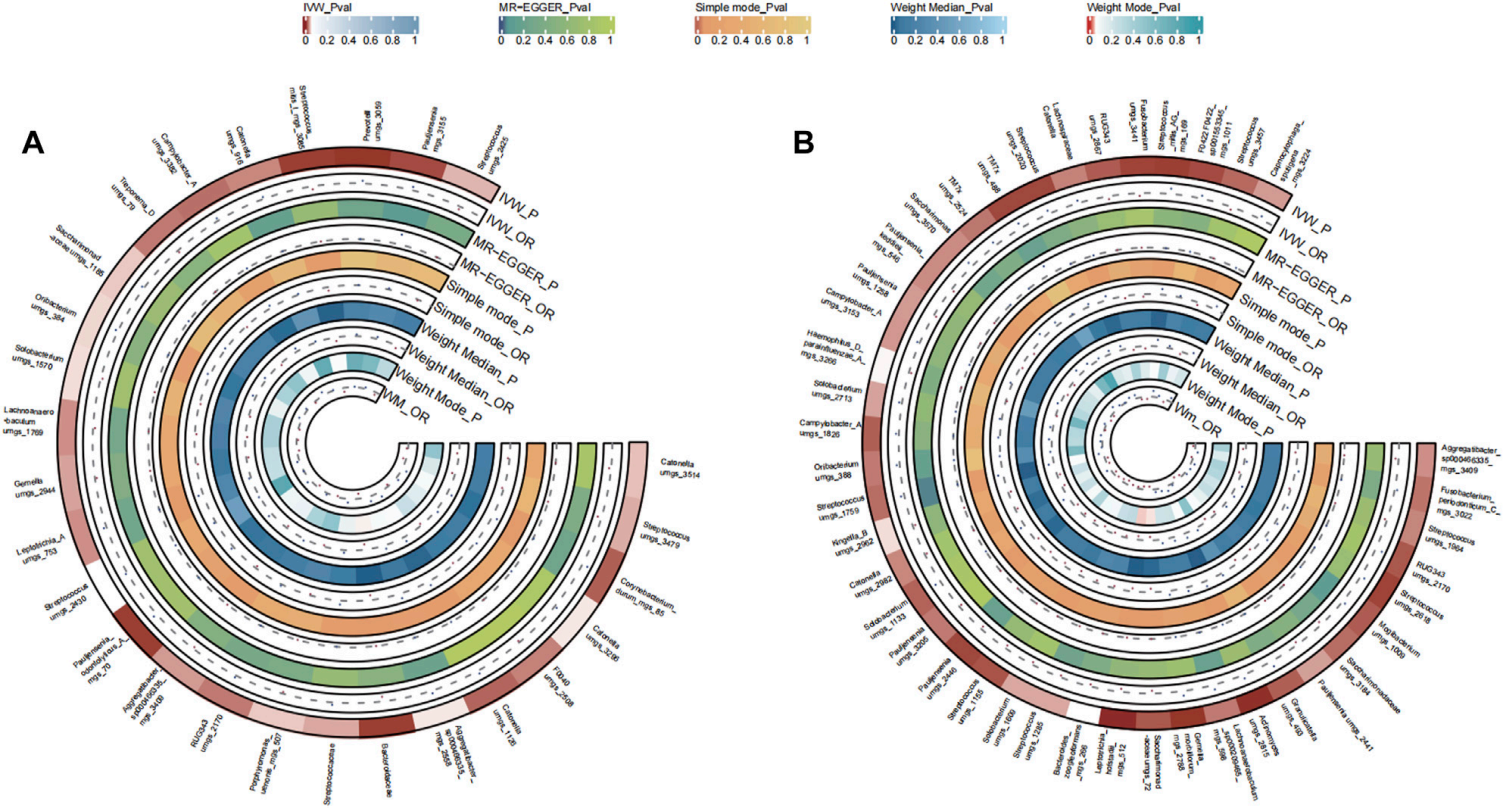
Circular Heatmap of Mendelian randomization results of oral microflora at species level and prostate cancer **(A)**, tongue, **(B)** saliva.

A total of 27 bacterial species in the tongue (17 genus and 14 family) and 42 bacterial species in saliva (24 genus and 17 family) had statistically significant relationships with PCa. In prostate cancer samples, nine genera were identified from both the tongue and saliva. Strikingly, Oribacterium, Pauljensenia, *Campylobacter* A, Catonella, Lachnoanaerobaculum, and RUG343 were implicated in possibly elevating the disease risk (OR >1). In contrast, Aggregatibacter, Solobacterium, *Streptococcus*, and Gemella were potentially inhibitory (OR <1). The causal effects of tongue and saliva bacterial species on prostate cancer were shown in Figures 7A, B.

#### 3.2.7 Ovarian cancer

**FIGURE 8:**
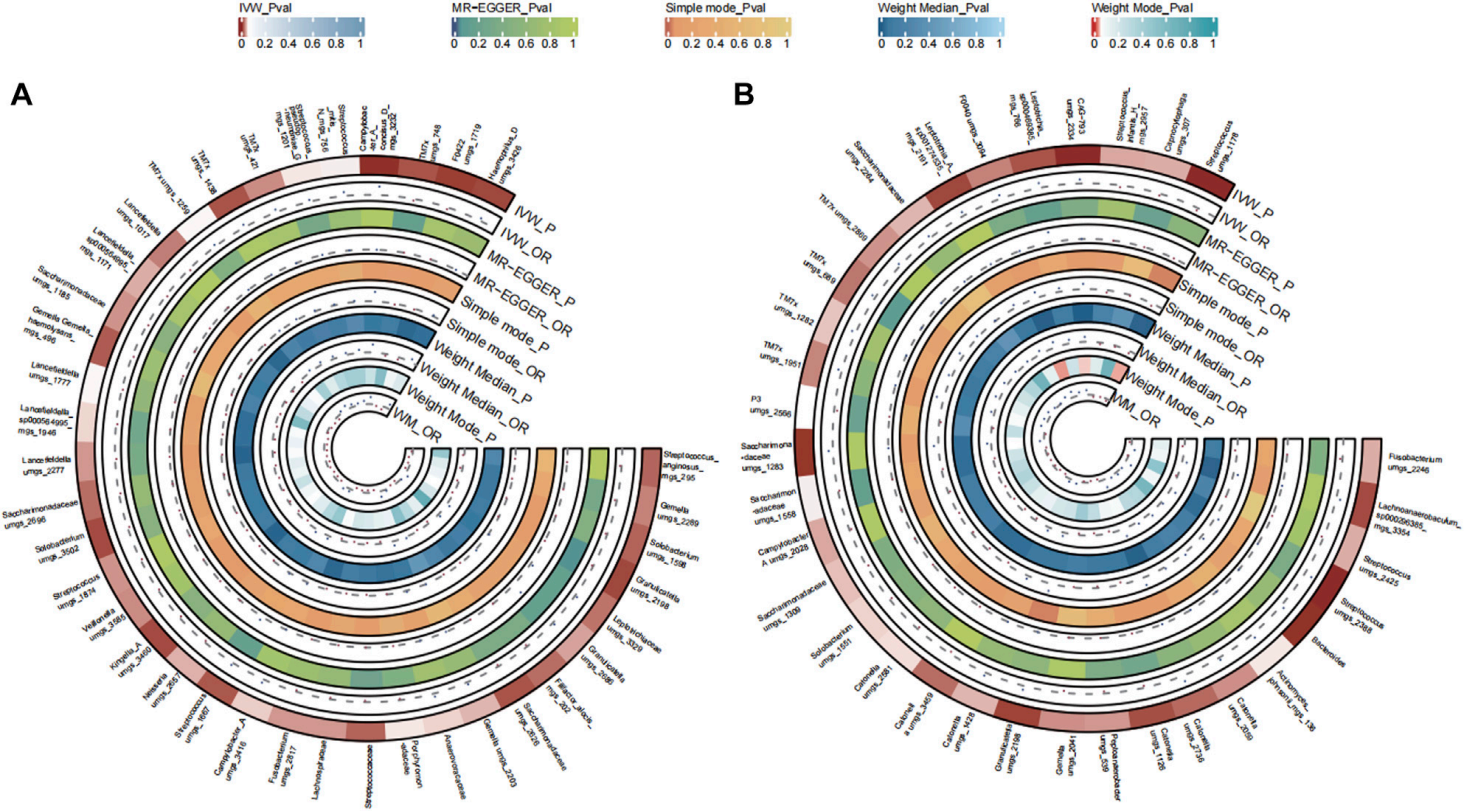
Circular Heatmap of Mendelian randomization results of oral microflora at species level and ovarian cancer **(A)**, tongue, **(B)** saliva.

A total of 39 bacterial species in the tongue (20 genus and 16 family) and 33 bacterial species in saliva (22 genus and 15 family) had statistically significant relationships with OC. In the context of OC, seven genera coexisted in both sample types. Interestingly, *Streptococcus*, *Campylobacter* A, Granulicatella, and Saccharimonadaceae TM7x might enhance disease risk (OR >1), in contrast to Solobacterium, *Fusobacterium*, Gemella, and Saccharimonadaceae umgs 1558 which appeared to be protective (OR <1). The causal effects of tongue and saliva bacterial species on ovarian cancer were shown in Figures 8A, B.

### 3.3 Comprehensive relationship between specific oral microbiota genera and diverse cancers

**FIGURE 9:**
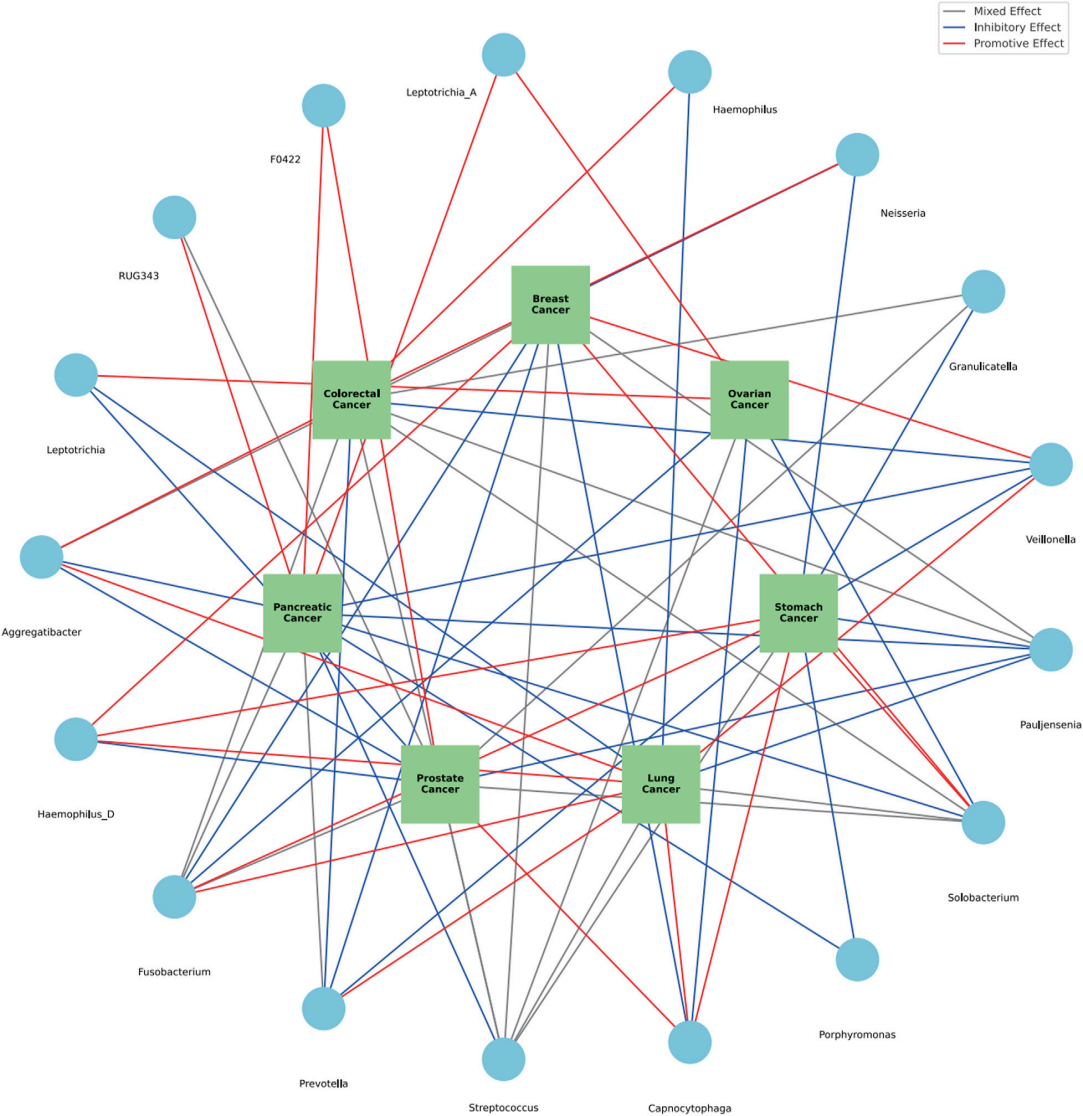
The comprehensive relationships between specific oral microflora at genus level and diverse cancers.

Within the diverse ecosystem of oral microbiota, certain bacterial genera have arisen as potential modulators of various cancer types, as discerned from an integrated analysis of both saliva and tongue datasets. The genus Aggregatibacter show cases a multifaceted influence, with a promotive role in breast and lung cancers, an inhibitory stance towards pancreatic and prostate cancers, a nuanced effect on colorectal, gastric and ovarian cancers. *Haemophilus* D reveals an intriguing profile, acting as a promoter for breast, stomach, and lung cancers, while seemingly offering protection against prostate cancer. *Fusobacterium* stands out with its diverse associations, promoting breast, colorectal and pancreatic cancers, while adopting a more complex mixed stance on prostate cancers. Interestingly, it exhibits inhibitory effects against ovarian and lung cancers. The genus Prevotella leans towards an inhibitory role in breast, stomach, and colorectal cancers, but its association with pancreatic cancer is mixed, and it distinctly promotes lung cancer. *Streptococcus*, a ubiquitous member of the oral microbiome, presents a mosaic of effects. While it demonstrates mixed associations with a suite of cancers, including breast, colorectal, stomach, prostate, and ovarian, its influence on pancreatic cancer is predominantly inhibitory, and for lung cancer, it remains diverse. This intricate dance of associations continues with Capnocytophaga, which suppresses breast and ovarian cancers, but promotes stomach, lung, and notably, prostate cancers. Porphyromonas, on the other hand, consistently inhibits stomach and pancreatic cancers. The canvas of associations broadens with Solobacterium promoting breast and stomach cancers, inhibiting pancreatic and ovarian, while showing mixed tendencies for colorectal, prostate, and lung cancers. Pauljensenia mostly exerts inhibitory effects, especially against stomach, pancreatic, prostate, and lung cancers, but its influence on breast and colorectal cancers remains mixed. Other genera, including Veillonella, Granulicatella, *Neisseria*, *Haemophilus*, Leptotrichia A, Veillonellaceae F0422, RUG343, and Leptotrichia, further enrich this complex tapestry of interactions, each with their distinct patterns of promotion, inhibition, or mixed effects on various cancers. Summary of multiple cancers in relation to oral microbiota genera was presented in Table 2. The comprehensive relationships between specific oral microflora at genus level and diverse cancers were shown in Figure 9.

**TABLE 2:** Summary of multiple cancers in relation to bacteria (genera) present in both tongue and saliva.

Outcomes	Mechanism	Quantity	Bacterial (genus)
Breast cancer	promote	5	Aggregatibacter, *Campylobacter*, *Fusobacterium*, *Streptococcus*, TM7x
	inhibit	1	Prevotella
	mix	7	Aggregatibacter, *Fusobacterium*, Oribacterium, Saccharimonadaceae, *Streptococcus*, Solobacterium, *Haemophilus*
Colorectal cancer	promote	1	*Haemophilus*
	inhibit	1	Gemella
	mix	9	*Campylobacter*, Catonella, *Fusobacterium*, Granulicatella, Lancefieldella, Pauljensenia, Solobacterium, TM7x, *Streptococcus*
Lung cancer	promote	0	None
	inhibit	0	None
	mix	5	Aggregatibacter, *Campylobacter*, *Fusobacterium*, *Streptococcus*, TM7x
Gastric cancer	promote	1	*Haemophilus*
	inhibit	2	Lancefieldella, Prevotella
	mix	6	*Campylobacter*, *Fusobacterium*, Granulicatella, Pauljensenia, Saccharimonadaceae, *Streptococcus*
Pancreatic cancer	promote	0	None
	inhibit	3	Gemella, Pauljensenia, *Treponema*
	mix	8	F0422, *Fusobacterium*, Lancefieldella, Prevotella, Saccharimonadaceae, Solobacterium, *Streptococcus*, TM7x
Ovarian cancer	promote	1	*Fusobacterium*
	inhibit	1	Solobacterium
	mix	6	*Campylobacter*, Gemella, Granulicatella, Saccharimonadaceae, *Streptococcus*, TM7x
Prostate cancer	promote	2	*Campylobacter*, Oribacterium
	inhibit	0	None
	mix	8	Aggregatibacter, Catonella, Leptotrichia, Pauljensenia, RUG343, Saccharimonadaceae, Solobacterium, *Streptococcus*

## 4 Discussion

The oral microbiome has the second highest amount of diversity, with a diverse range of bacterial genera and families, which was second only to that of gut (Caselli et al., 2020). The relationship between the oral microbiota and various forms of cancers has been the focus of numerous studies, but the exact mechanisms and implications remain partially understood. Our study, using MR, provides insights into this relationship (Allen and Sears, 2019).

Numerous studies have delved into the relationship between oral microbiota and breast cancer, uncovering various facets of their interaction. A study conducted in Ghana probed the association between the oral microbiome and breast cancer, identifying a potential linkage akin to that observed between the fecal microbiome and breast cancer risk (Wu et al., 2022). Particularly, a linkage seems to exist between breast cancer and the oral microbiota. Women with periodontal disease, triggered by specific bacteria like the red complex (Porphyromonas, Tannerella, and *Treponema*) and the orange complex (*Fusobacterium*, Prevotella, *Peptostreptococcus*, *Streptococcus*, Eubacterium, and *Campylobacter*), are observed to have a heightened risk of breast cancer (Thompson et al., 2017; Wang et al., 2017; Huang et al., 2018). The presence of oral pathogens has also been discovered in breast cancer tumor tissues, notably *Fusobacterium*. The suggested mechanism involves these bacteria entering breast tissues via the bloodstream, potentially driving inflammation and tumorigenic processes (Parhi et al., 2020; Little et al., 2023). In addition, the ability of *Fusobacterium* to colonize malignant breast tumors by adhering to GalNAc receptors points towards a possible mechanism. Such colonization could alter the tumor microenvironment, modulating host cell signaling, immune responses, or even producing metabolites that promote cancers. *Streptococcus* species are abundant in the oral cavity (Yu et al., 2022). In women without breast tumors, researchers observed elevated levels of Lactococcus and *Streptococcus*, suggesting these bacteria may possess anti-cancer properties. Another study found that in healthy patients, the relative abundance of *Streptococcus* was higher, while there was a negative correlation between the stage of breast cancer and bacterial load in tumor tissue. Through MR analysis, we confirmed the association of Aggregatibacter, *Fusobacterium*, *Streptococcus*, and Saccharimonadaceae, along with Prevotella, Oribacterium, and Solobacterium, with breast cancer.

Lung cancer, another major global health concern, may find its etiological roots intertwined with oral health. In three US cohort studies, Vogtmann et al. found certain types of oral flora, like *Streptococcus*, has a favourable association with the risk of developing lung cancer. *Streptococcus* abundance was associated with 1.14 times the risk of lung cancer (95% confidence interval = 1.06–1.22) (Vogtmann et al., 2022). Sun et al. (2023) and Zhang et al. (2019) also reported the levels of *Streptococcus* in saliva samples collected from patients with LC are considerably elevated compared to those in control samples. A study by Yan et al. demonstrated Capnocytophaga and Veillonella were significantly higher in the saliva from lung cancer patients (Yan et al., 2015). A study sought to prospectively investigate the association of the oral microbiome with lung cancer risk, involving 156 incident lung cancer cases and 156 individually matched controls (Shi et al., 2021). Capnocytophaga was associated with a decreased risk of lung cancer with ORs and 95% CIs of 0.53 (0.31–0.92). These studies suggested that the oral microbiome may present new avenues for lung cancer prevention. In our study, we found a positive correlation between Aggregatibacter and Gemella with LC, while *Fusobacterium*, *Streptococcus*, *Campylobacter* A, and Saccharimonadaceae were negatively associated with LC progression. These microbial communities have also been validated in previous literature.

Pancreatic cancer, a highly aggressive digestive system malignancy, has ascended to the third leading cause of cancer-related deaths with an increasing incidence rate, imposing significant global health and economic burdens. Characterized by late symptom manifestation and limited therapeutic options, its 5-year survival rate is below 9%, earning it the moniker king of cancers. In a landmark study utilizing the Cancer Prevention Study II (CPSII) and Prostate, Lung, Colorectal and Ovarian (PLCO) prospective databases, Fan et al. found Porphyromonas gingivalis and Aggregatibacter, were associated with higher risk of pancreatic cancer (adjusted OR for presence vs. absence = 1.60 and 95% CI 1.15 to 2.22; OR = 2.20 and 95% CI 1.16 to 4.18, respectively) and Phylum Fusobacteria was associated with decreased PC risk (Fan et al., 2018). Herremans et al.’s review summarized the correlation between oral microbiota and pancreatic cancer (Herremans et al., 2022). Oral bacteria associated with an increased risk of pancreatic cancer include Porphyromonas (Michaud et al., 2013), Aggregatibacter, Enterobacteriaceae, Lachnospiraceae G7 (35), Bacteroidaceae (Vogtmann et al., 2020), Staphylococcaceae (Vogtmann et al., 2020), Gemella adiacens (Farrell et al., 2012), and Firmicutes (Al-Zyoud et al., 2019; Wei et al., 2020). Conversely, oral bacteria associated with a decreased risk of pancreatic cancer include *Fusobacterium* (Mitsuhashi et al., 2015; Wei et al., 2020) and Leptotrichia (Torres et al., 2015; Fan et al., 2018), *Haemophilus* (Vogtmann et al., 2020), *Streptococcus* mitis (Wei et al., 2020), *Neisseria* (Wei et al., 2020), and Proteobacteria (Al-Zyoud et al., 2019). In our study, by MR analysis, the association of *Fusobacterium*, Aggregatibacter, Solobacterium, *Streptococcus*, Gemella, Porphyromonas, Saccharimonadaceae, and Lancefieldella with pancreatic cancer was demonstrated, aligning with previous research. We also found Veillonellaceae, Prevotella, Oribacterium were associated with pancreatic cancer. Veillonellaceae F0422 belongs to the phyla Firmicutes, a common oral microbiome that may be related to gastrointestinal cancers (Asili et al., 2023). Research has indicated a correlation between Prevotella bacteria presence and symptoms of pancreatic cancer. Notably, a higher abundance of Prevotella (*p* = 0.008) was observed in patients reporting jaundice (Wei et al., 2020).

Colorectal cancer, with its burgeoning global incidence, shown associations with gut microbiome. Disruptions in the intestinal flora can stimulate an excessive immune response, leading to the release of inflammatory cytokines such as TNF-α, IL-16, and IL-1β (Chen F. et al., 2022; Kong et al., 2023). Specific periodontopathogens, like *Fusobacterium* nucleatum, have been linked to the development of CC and hold potential as biomarkers for cancer diagnosis (Negrut et al., 2023). Alterations in the oral microbiome, including varying levels of specific taxa like *Streptococcus* and Prevotella, have been noted in individuals with CC. Furthermore, a higher abundance of certain oral-like bacterial networks in colonic tissue correlated with CC, whereas an increased abundance of Lachnospiraceae was inversely associated with the colonization of colonic tissue by these oral-like bacterial networks, hinting at a potential protective effect against CC (Negrut et al., 2023). Evidence indicates that the oral microbiome may colonize the gut, resulting in the dysregulation of gut microbes. This colonization fosters an intestinal inflammatory and immunosuppressive microenvironment, which could potentially facilitate tumorigenesis and progression of CC (Mo et al., 2022).

The oral microbiota is a crucial factor in the onset and progression of gastric cancer in humans, associated with inflammation of the gastric mucosa, interactions in the upper gastrointestinal tract, and the infection and transmission of *Helicobacter pylori* (Stasiewicz and Karpiński, 2022). The oral microbiota of gastric cancer patients exhibits significant differences compared to healthy individuals, with an increased abundance of certain oral bacteria such as Porphyromonas gingivalis, *Fusobacterium*, and Streptococci, potentially linked to the onset and advancement of gastric cancer (Șurlin et al., 2020). Like CC, the abundance of certain oral bacteria can migrate to the stomach, altering the gastric microbial milieuoral, producing harmful metabolites or carcinogens in the stomach, and elevating cancer risks. In study of Shu et al., the abundance of *Fusobacterium*, Prevotella, *Neisseria* were significantly changed, compared to healthy controls (Zhang et al., 2022). Chronic prostatitis (CP) and benign prostatic hyperplasia (BPH) are chronic inflammation in the prostate and the etiology is linked to the disorders of oral microbiome (Boland et al., 2013).

The migration of bacteria and chronic inflammation also apply for the correlation between oral microbiota and prostate cancer. Research suggests a potential association between oral microbiota and the development of prostate diseases, notably prostate cancer (Fang et al., 2021). A common chronic inflammatory condition, periodontal disease, shares risk factors with prostate diseases. Inflammation is deemed a significant factor in the progression of prostate diseases. Periodontal disease could potentially lead to increased expression of pro-inflammatory cytokines, thereby impacting prostate health. Additionally, periodontal treatment can significantly alleviate symptoms of prostatic inflammation. Furthermore, oral pathogens, such as Porphyromonas gingivalis and *Fusobacterium* nucleatum, might migrate to the prostate via the bloodstream, inducing analogous pathogenic effects (Wu et al., 2019). These bacterial aggregates may lead to the development of chronic inflammation and may cause damage and abnormal proliferation of prostate cells, or even cancer, thereby increasing the risk of prostate cancer (Fang et al., 2021).

OC is leading oncological cause of death among women. Unlike the above cancers, there is no sufficient evidence substantiating causal relationships between oral bacteria and OC. While the literature on associations of ovarian cancers with oral microbiota is nascent, emerging evidence suggests potential risk elevations tied to alterations in bacterial profiles in numerous compartments, including vaginal, cervicovaginal (Nené et al., 2019), upper genital tract (Zhou et al., 2019), peritoneal (Miao et al., 2020), serum (Kim et al., 2020), and fecal (Mori et al., 2019) compartments in patients with OC.

In the present study, we obtained several important findings. First of all, *Streptococcus* is the sole bacterial genus associated with all seven cancer types. *Streptococcus* is predominantly found in the oral cavity, pharynx, and nasal passages. Its association with multiple cancers is established, underscored by studies indicating shifts in its abundance at respective cancerous sites. Specifically, increased levels were discerned in the mammary tissues of breast cancer patients, bronchoalveolar lavage fluids of lung cancer patients, gastric mucosa of those with stomach cancer, ovarian tissues in ovarian cancer patients, prostate tissues in prostate cancer subjects, and within the microbial communities of the pancreas (Xie et al., 2022). Then, excluding prostate cancer, *Fusobacterium* exhibits associations with six other cancer types. *Fusobacterium* is an oral bacterium, recent investigations have revealed the its intricate involvement in colorectal, breast and pancreatic cancers pathogenesis (Bullman et al., 2017; Parhi et al., 2020; Alon-Maimon et al., 2022; Ou et al., 2022; Udayasuryan et al., 2022). In our MR analysis, *Fusobacterium* is a favorable factor for breast, colorectal and pancreatic cancers, consistent with published studies. This bacterium’s capability to adhere to both healthy and neoplastic cells hinge on specific molecular recognition, subsequently activating β-catenin-centric transcriptional pathways, potentiating carcinogenesis. *Fusobacterium* can migrate to the intestines, reshaping the microbial landscape (Yu et al., 2017; Chen S. et al., 2022). This translocation plays a pivotal role in establishing a tumor-immunosuppressive environment, augmenting cancer cell spread by triggering the host’s innate immune mechanisms (Dai et al., 2018; Zhao et al., 2022). Noteworthy is the concurrent identification of *Fusobacterium* in oral and colorectal cancer specimens from patients, highlighting the profound interplay between oral microbiota and colorectal malignancy. Furthermore, Prevotella, recognized as the second most abundant genus in the human oral microbiome, has inhibitory role in breast, stomach, and colorectal cancers in our MR analysis. In the published literature (Niccolai et al., 2020; Huh et al., 2022), the influence of Prevotella on cancer progression appears to be multifaceted, contingent upon the cancer type, its stage, the host’s immunological profile, and different body sites. Emerging evidence delineates its potential anticancer properties, manifested through mechanisms such as the amplification of immune responses, attenuation of inflammation, or induction of cellular apoptosis. Conversely, other studies implicate Prevotella in exacerbating cancer progression, possibly by bolstering cellular proliferation, invasiveness, or metastatic activities. Such dichotomies emphasize the need for nuanced interpretations of Prevotella’s role in oncogenesis and progression. Such disparities underscore the premise that identical bacterial taxa might manifest contrasting effects, contingent upon the environmental conditions they inhabit. Finally, in both colorectal and gastric cancers, *Haemophilus* stands out as the sole bacterial genus exerting a purely promotive effect. *Haemophilus* emerges as a commonly found inhabitant, predominantly residing within the oral cavity. Intriguingly, a heightened abundance of this bacterial genus has been observed in individuals diagnosed with colorectal cancer. This proliferation might be attributed to its potential role in inducing inflammatory responses and aberrations in the immune system, thereby fostering the progression of colorectal cancer. Complementing this, certain studies have illuminated the significant distinction in the absolute abundance of *Haemophilus* between gastric cancer patients and their healthy counterparts (*p* ≤ 0.05) (Liu D. et al., 2021; Zhang et al., 2022). Such findings dovetail with our research, underscoring the contributory role of *Haemophilus* in the advancement of both gastric and colorectal cancers.

In essence, a singular bacterium may manifest dual roles in oncogenesis, utilizing distinct mechanisms to alternately inhibit or advance cancer progression. The association between the oral microbiome and cancer has significant implications for public health and clinical practice. Screening for alterations in the oral microbiome may become part of routine cancer risk assessments. Patients with a dysbiotic oral microbiome may benefit from early intervention strategies, such as microbiome modulation through diet, prebiotics, probiotics, or even microbiota transplants. Furthermore, our findings suggest that manipulation of the oral microbiome may serve as a novel therapeutic avenue. If a certain bacterial genus is found to promote carcinogenesis, strategies to suppress this microbial population may be pursued. Conversely, if certain bacteria are found to exert protective effects, these may be encouraged through targeted therapies.

This study possesses several notable strengths. Firstly, it utilizes the most up-to-date GWAS data pertaining to the oral microbiome and employs MR as a methodological approach to establish causal connections. Furthermore, our comprehensive analysis reveals the unexplored and multifaceted causal relationship between oral microbiota and diverse cancers. The complex network of relationships between oral microbiota and cancer development highlights the importance of a comprehensive understanding of this intricate system. As we continue to explore this area, these findings provide new insights for future research and potential treatment approaches. This is the first Mendelian Randomization analysis on the relationship between oral microbiota and various cancers in an East Asian population. Additionally, it was precisely identified down to the species level.

However, it is important to note that this study has several limitations that need to be addressed. The potential for horizontal pleiotropy may impact the selection of instrumental variables in MR studies. The oral microbiome can be influenced by a variety of factors, including genetic inheritance, lifestyle choices, dietary changes, and environmental factors. Instrumental variables may only account for a small portion of the observed variability, and further research is needed to fully understand the complex changes in the oral microbiota. Additionally, our MR analysis focused on populations of Asian ancestry, and our findings may not be generalizable to populations of European ancestry.

## 5 Conclusion

Utilizing recent GWAS datasets and Mendelian randomization, the findings of this study support the plausibility of a causal relationship between oral microbiota and seven cancers. These links suggest diverse roles in influencing cancer evolution, utilizing distinct mechanisms to alternately inhibit or advance cancer progression. Particular bacteria, notably *Streptococcus* and *Fusobacterium*, displaying significant correlations with various cancers. The study offers a foundational step towards understanding the profound implications of the oral microbiome in systemic malignancies.

## Data Availability

Publicly available datasets were analyzed in this study. The data of oral microbime can be found here: https://db.cngb.org/search/project/CNP0001664. The data of seven types of cancers can be found here: https://gwas.mrcieu.ac.uk/.

## References

[B1] Abecasis G. R. Altshuler D. Auton A. Brooks L. D. Durbin R. M. Gibbs R. A. (2010). A map of human genome variation from population-scale sequencing. Nature 467 (7319), 1061–1073. Epub 2010/10/29. 10.1038/nature09534 20981092 PMC3042601

[B2] Allen J. Sears C. L. (2019). Impact of the gut microbiome on the genome and epigenome of colon epithelial cells: contributions to colorectal cancer development. Genome Med. 11 (1), 11. Epub 2019/02/26. 10.1186/s13073-019-0621-2 30803449 PMC6388476

[B3] Alon-Maimon T. Mandelboim O. Bachrach G. (2022). Fusobacterium nucleatum and cancer. Periodontol 89 (1), 166–180. Epub 2022/03/05. 10.1111/prd.12426 PMC9315032 35244982

[B4] Al-Zyoud W. Hajjo R. Abu-Siniyeh A. Hajjaj S. (2019). Salivary microbiome and cigarette smoking: a first of its kind investigation in Jordan. Int. J. Environ. Res. Public Health 17 (1), 256. Epub 20191230. 10.3390/ijerph17010256 31905907 PMC6982339

[B5] Asili P. Mirahmad M. Rezaei P. Mahdavi M. Larijani B. Tavangar S. M. (2023). The association of oral microbiome dysbiosis with gastrointestinal cancers and its diagnostic efficacy. J. Gastrointest. Cancer. Epub 2023/01/05. 10.1007/s12029-022-00901-4 36600023

[B6] Boland M. R. Hripcsak G. Albers D. J. Wei Y. Wilcox A. B. Wei J. (2013). Discovering medical conditions associated with periodontitis using linked electronic health records. J. Clin. Periodontol. 40 (5), 474–482. Epub 2013/03/19. 10.1111/jcpe.12086 23495669 PMC3690348

[B7] Bowden J. Davey Smith G. Haycock P. C. Burgess S. (2016). Consistent estimation in mendelian randomization with some invalid instruments using a weighted median estimator. Genet. Epidemiol. 40 (4), 304–314. Epub 2016/04/12. 10.1002/gepi.21965 27061298 PMC4849733

[B8] Bowden J. Holmes M. V. (2019). Meta-analysis and mendelian randomization: a review. Res. Synth. Methods 10 (4), 486–496. Epub 2019/03/13. 10.1002/jrsm.1346 30861319 PMC6973275

[B9] Bullman S. Pedamallu C. S. Sicinska E. Clancy T. E. Zhang X. Cai D. (2017). Analysis of Fusobacterium persistence and antibiotic response in colorectal cancer. Science 358 (6369), 1443–1448. Epub 2017/11/25. 10.1126/science.aal5240 29170280 PMC5823247

[B10] Burgess S. Davey Smith G. Davies N. M. Dudbridge F. Gill D. Glymour M. M. (2019). Guidelines for performing mendelian randomization investigations. Wellcome Open Res. 4, 186. Epub 2020/08/11. 10.12688/wellcomeopenres.15555.2 32760811 PMC7384151

[B11] Burgess S. Foley C. N. Zuber V. (2018). Inferring causal relationships between risk factors and outcomes from genome-wide association study data. Annu. Rev. Genomics Hum. Genet. 19, 303–327. Epub 2018/05/02. 10.1146/annurev-genom-083117-021731 29709202 PMC6481551

[B12] Burgess S. Thompson S. G. CRP CHD Genetics Collaboration (2011). Avoiding bias from weak instruments in mendelian randomization studies. Int. J. Epidemiol. 40 (3), 755–764. Epub 2011/03/19. 10.1093/ije/dyr036 21414999

[B13] Burgess S. Thompson S. G. (2015). Multivariable mendelian randomization: the use of pleiotropic genetic variants to estimate causal effects. Am. J. Epidemiol. 181 (4), 251–260. Epub 20150127. 10.1093/aje/kwu283 25632051 PMC4325677

[B14] Burgess S. Thompson S. G. (2017). Interpreting findings from mendelian randomization using the mr-egger method. Eur. J. Epidemiol. 32 (5), 377–389. Epub 2017/05/21. 10.1007/s10654-017-0255-x 28527048 PMC5506233

[B15] Caselli E. Fabbri C. D'Accolti M. Soffritti I. Bassi C. Mazzacane S. (2020). Defining the oral microbiome by whole-genome sequencing and resistome analysis: the complexity of the healthy picture. BMC Microbiol. 20 (1), 120. Epub 2020/05/20. 10.1186/s12866-020-01801-y 32423437 PMC7236360

[B16] Chen F. Dai X. Zhou C. C. Li K. X. Zhang Y. J. Lou X. Y. (2022a). Integrated analysis of the faecal metagenome and serum metabolome reveals the role of gut microbiome-associated metabolites in the detection of colorectal cancer and adenoma. Gut 71 (7), 1315–1325. Epub 2021/09/01. 10.1136/gutjnl-2020-323476 34462336 PMC9185821

[B17] Chen S. Zhang L. Li M. Zhang Y. Sun M. Wang L. (2022b). Fusobacterium nucleatum reduces mettl3-mediated M(6)a modification and contributes to colorectal cancer metastasis. Nat. Commun. 13 (1), 1248. Epub 2022/03/12. 10.1038/s41467-022-28913-5 35273176 PMC8913623

[B18] Dai Z. Coker O. O. Nakatsu G. Wu W. K. K. Zhao L. Chen Z. (2018). Multi-cohort analysis of colorectal cancer metagenome identified altered bacteria across populations and universal bacterial markers. Microbiome 6 (1), 70. Epub 2018/04/13. 10.1186/s40168-018-0451-2 29642940 PMC5896039

[B19] Fan X. Alekseyenko A. V. Wu J. Peters B. A. Jacobs E. J. Gapstur S. M. (2018). Human oral microbiome and prospective risk for pancreatic cancer: a population-based nested case-control study. Gut 67 (1), 120–127. Epub 2016/11/02. 10.1136/gutjnl-2016-312580 27742762 PMC5607064

[B20] Fang C. Wu L. Zhu C. Xie W. Z. Hu H. Zeng X. T. (2021). A potential therapeutic strategy for prostatic disease by targeting the oral microbiome. Med. Res. Rev. 41 (3), 1812–1834. Epub 20201230. 10.1002/med.21778 33377531 PMC8246803

[B21] Farrell J. J. Zhang L. Zhou H. Chia D. Elashoff D. Akin D. (2012). Variations of oral microbiota are associated with pancreatic diseases including pancreatic cancer. Gut 61 (4), 582–588. Epub 20111012. 10.1136/gutjnl-2011-300784 21994333 PMC3705763

[B22] Hemani G. Zheng J. Elsworth B. Wade K. H. Haberland V. Baird D. (2018). The mr-base platform supports systematic causal inference across the human phenome. Elife 7, e34408. Epub 2018/05/31. 10.7554/eLife.34408 29846171 PMC5976434

[B23] Herremans K. M. Riner A. N. Cameron M. E. McKinley K. L. Triplett E. W. Hughes S. J. (2022). The oral microbiome, pancreatic cancer and human diversity in the age of precision medicine. Microbiome 10 (1), 93. Epub 20220615. 10.1186/s40168-022-01262-7 35701831 PMC9199224

[B24] Huang Y. F. Chen Y. J. Fan T. C. Chang N. C. Chen Y. J. Midha M. K. (2018). Analysis of microbial sequences in plasma cell-free DNA for early-onset breast cancer patients and healthy females. BMC Med. Genomics 11 (Suppl. 1), 16. Epub 20180213. 10.1186/s12920-018-0329-y 29504912 PMC5836824

[B25] Huh J. W. Kim M. J. Kim J. Lee H. G. Ryoo S. B. Ku J. L. (2022). Enterotypical Prevotella and three novel bacterial biomarkers in preoperative stool predict the clinical outcome of colorectal cancer. Microbiome 10 (1), 203. Epub 2022/11/30. 10.1186/s40168-022-01388-8 36443754 PMC9703702

[B26] Ishigaki K. Akiyama M. Kanai M. Takahashi A. Kawakami E. Sugishita H. (2020). Large-scale genome-wide association study in a Japanese population identifies novel susceptibility loci across different diseases. Nat. Genet. 52 (7), 669–679. Epub 20200608. 10.1038/s41588-020-0640-3 32514122 PMC7968075

[B27] Kim S. I. Kang N. Leem S. Yang J. Jo H. Lee M. (2020). Metagenomic analysis of serum microbe-derived extracellular vesicles and diagnostic models to differentiate ovarian cancer and benign ovarian tumor. Cancers (Basel) 12 (5), 1309. Epub 2020/05/28. 10.3390/cancers12051309 32455705 PMC7281409

[B28] Kong C. Liang L. Liu G. Du L. Yang Y. Liu J. (2023). Integrated metagenomic and metabolomic analysis reveals distinct gut-microbiome-derived phenotypes in early-onset colorectal cancer. Gut 72 (6), 1129–1142. Epub 2022/08/12. 10.1136/gutjnl-2022-327156 35953094

[B29] Little A. Tangney M. Tunney M. M. Buckley N. E. (2023). Fusobacterium nucleatum: a novel immune modulator in breast cancer? Expert Rev. Mol. Med. 25, e15. Epub 20230403. 10.1017/erm.2023.9 37009688 PMC10407221

[B30] Liu D. Chen S. Gou Y. Yu W. Zhou H. Zhang R. (2021b). Gastrointestinal microbiota changes in patients with gastric precancerous lesions. Front. Cell Infect. Microbiol. 11, 749207. Epub 2021/12/28. 10.3389/fcimb.2021.749207 34956928 PMC8695999

[B31] Liu X. Tong X. Zhu J. Tian L. Jie Z. Zou Y. (2021a). Metagenome-genome-wide association studies reveal human genetic impact on the oral microbiome. Cell Discov. 7 (1), 117. Epub 2021/12/08. 10.1038/s41421-021-00356-0 34873157 PMC8648780

[B32] Miao R. Badger T. C. Groesch K. Diaz-Sylvester P. L. Wilson T. Ghareeb A. (2020). Assessment of peritoneal microbial features and tumor marker levels as potential diagnostic tools for ovarian cancer. PLoS One 15 (1), e0227707. Epub 2020/01/10. 10.1371/journal.pone.0227707 31917801 PMC6952086

[B33] Michaud D. S. Izard J. Wilhelm-Benartzi C. S. You D. H. Grote V. A. Tjønneland A. (2013). Plasma antibodies to oral bacteria and risk of pancreatic cancer in a large European prospective cohort study. Gut 62 (12), 1764–1770. Epub 20120918. 10.1136/gutjnl-2012-303006 22990306 PMC3815505

[B34] Mitsuhashi K. Nosho K. Sukawa Y. Matsunaga Y. Ito M. Kurihara H. (2015). Association of Fusobacterium species in pancreatic cancer tissues with molecular features and prognosis. Oncotarget 6 (9), 7209–7220. 10.18632/oncotarget.3109 25797243 PMC4466679

[B35] Mo S. Ru H. Huang M. Cheng L. Mo X. Yan L. (2022). Oral-intestinal microbiota in colorectal cancer: inflammation and immunosuppression. J. Inflamm. Res. 15, 747–759. Epub 20220204. 10.2147/jir.S344321 35153499 PMC8824753

[B36] Mori G. Orena B. S. Cultrera I. Barbieri G. Albertini A. M. Ranzani G. N. (2019). Gut microbiota analysis in postoperative lynch syndrome patients. Front. Microbiol. 10, 1746. Epub 2019/08/17. 10.3389/fmicb.2019.01746 31417532 PMC6682596

[B37] Negrut R. L. Cote A. Maghiar A. M. (2023). Exploring the potential of oral microbiome biomarkers for colorectal cancer diagnosis and prognosis: a systematic review. Microorganisms 11 (6), 1586. Epub 20230615. 10.3390/microorganisms11061586 37375087 PMC10305386

[B38] Nené N. R. Reisel D. Leimbach A. Franchi D. Jones A. Evans I. (2019). Association between the cervicovaginal microbiome, Brca1 mutation status, and risk of ovarian cancer: a case-control study. Lancet Oncol. 20 (8), 1171–1182. Epub 2019/07/14. 10.1016/s1470-2045(19)30340-7 31300207

[B39] Niccolai E. Russo E. Baldi S. Ricci F. Nannini G. Pedone M. (2020). Significant and conflicting correlation of il-9 with Prevotella and Bacteroides in human colorectal cancer. Front. Immunol. 11, 573158. Epub 2021/01/26. 10.3389/fimmu.2020.573158 33488574 PMC7820867

[B40] Ou S. Wang H. Tao Y. Luo K. Ye J. Ran S. (2022). Fusobacterium nucleatum and colorectal cancer: from phenomenon to mechanism. Front. Cell Infect. Microbiol. 12, 1020583. Epub 2022/12/17. 10.3389/fcimb.2022.1020583 36523635 PMC9745098

[B41] Parhi L. Alon-Maimon T. Sol A. Nejman D. Shhadeh A. Fainsod-Levi T. (2020). Breast cancer colonization by Fusobacterium nucleatum accelerates tumor growth and metastatic progression. Nat. Commun. 11 (1), 3259. Epub 20200626. 10.1038/s41467-020-16967-2 32591509 PMC7320135

[B42] Purcell S. Neale B. Todd-Brown K. Thomas L. Ferreira M. A. Bender D. (2007). Plink: a tool set for whole-genome association and population-based linkage analyses. Am. J. Hum. Genet. 81 (3), 559–575. Epub 2007/08/19. 10.1086/519795 17701901 PMC1950838

[B43] Shi J. Yang Y. Xie H. Wang X. Wu J. Long J. (2021). Association of oral microbiota with lung cancer risk in a low-income population in the southeastern USA. Cancer Causes Control 32 (12), 1423–1432. Epub 20210825. 10.1007/s10552-021-01490-6 34432217 PMC8541916

[B44] Siegel R. L. Miller K. D. Fuchs H. E. Jemal A. (2022). Cancer statistics, 2022. CA Cancer J. Clin. 72 (1), 7–33. Epub 2022/01/13. 10.3322/caac.21708 35020204

[B45] Stasiewicz M. Karpiński T. M. (2022). The oral microbiota and its role in carcinogenesis. Semin. Cancer Biol. 86 (Pt 3), 633–642. Epub 20211104. 10.1016/j.semcancer.2021.11.002 34743032

[B46] Sun Y. Liu Y. Li J. Tan Y. An T. Zhuo M. (2023). Characterization of lung and oral microbiomes in lung cancer patients using culturomics and 16s rrna gene sequencing. Microbiol. Spectr. 11 (3), e0031423. Epub 2023/04/24. 10.1128/spectrum.00314-23 37092999 PMC10269771

[B47] Sung H. Ferlay J. Siegel R. L. Laversanne M. Soerjomataram I. Jemal A. (2021). Global cancer statistics 2020: globocan estimates of incidence and mortality worldwide for 36 cancers in 185 countries. CA Cancer J. Clin. 71 (3), 209–249. Epub 2021/02/05. 10.3322/caac.21660 33538338

[B48] Șurlin P. Nicolae F. M. Șurlin V. M. Ș P. Ungureanu B. S. Didilescu A. C. (2020). Could periodontal disease through periopathogen Fusobacterium nucleatum Be an aggravating factor for gastric cancer? J. Clin. Med. 9 (12), 3885. Epub 20201129. 10.3390/jcm9123885 33260439 PMC7761398

[B49] Teles F. R. F. Alawi F. Castilho R. M. Wang Y. (2020). Association or causation? Exploring the oral microbiome and cancer links. J. Dent. Res. 99 (13), 1411–1424. Epub 2020/08/20. 10.1177/0022034520945242 32811287 PMC7684840

[B50] Thompson K. J. Ingle J. N. Tang X. Chia N. Jeraldo P. R. Walther-Antonio M. R. (2017). A comprehensive analysis of breast cancer microbiota and host gene expression. PLoS One 12 (11), e0188873. Epub 20171130. 10.1371/journal.pone.0188873 29190829 PMC5708741

[B51] Torres P. J. Fletcher E. M. Gibbons S. M. Bouvet M. Doran K. S. Kelley S. T. (2015). Characterization of the salivary microbiome in patients with pancreatic cancer. PeerJ 3, e1373. Epub 20151105. 10.7717/peerj.1373 26587342 PMC4647550

[B52] Udayasuryan B. Ahmad R. N. Nguyen T. T. D. Umaña A. Monét Roberts L. Sobol P. (2022). Fusobacterium nucleatum induces proliferation and migration in pancreatic cancer cells through host autocrine and paracrine signaling. Sci. Signal 15 (756), eabn4948. Epub 2022/10/19. 10.1126/scisignal.abn4948 36256708 PMC9732933

[B53] Verbanck M. Chen C. Y. Neale B. Do R. (2018). Detection of widespread horizontal pleiotropy in causal relationships inferred from mendelian randomization between complex traits and diseases. Nat. Genet. 50 (5), 693–698. Epub 2018/04/25. 10.1038/s41588-018-0099-7 29686387 PMC6083837

[B54] Vogtmann E. Han Y. Caporaso J. G. Bokulich N. Mohamadkhani A. Moayyedkazemi A. (2020). Oral microbial community composition is associated with pancreatic cancer: a case-control study in Iran. Cancer Med. 9 (2), 797–806. Epub 20191121. 10.1002/cam4.2660 31750624 PMC6970053

[B55] Vogtmann E. Hua X. Yu G. Purandare V. Hullings A. G. Shao D. (2022). The oral microbiome and lung cancer risk: an analysis of 3 prospective cohort studies. J. Natl. Cancer Inst. 114 (11), 1501–1510. 10.1093/jnci/djac149 35929779 PMC9664178

[B56] Wang H. Altemus J. Niazi F. Green H. Calhoun B. C. Sturgis C. (2017). Breast tissue, oral and urinary microbiomes in breast cancer. Oncotarget 8 (50), 88122–88138. Epub 20170814. 10.18632/oncotarget.21490 29152146 PMC5675698

[B57] Warren R. L. Freeman D. J. Pleasance S. Watson P. Moore R. A. Cochrane K. (2013). Co-occurrence of anaerobic bacteria in colorectal carcinomas. Microbiome 1 (1), 16. Epub 2014/01/24. 10.1186/2049-2618-1-16 24450771 PMC3971631

[B58] Wei A. L. Li M. Li G. Q. Wang X. Hu W. M. Li Z. L. (2020). Oral microbiome and pancreatic cancer. World J. Gastroenterol. 26 (48), 7679–7692. 10.3748/wjg.v26.i48.7679 33505144 PMC7789059

[B59] Wu L. Li B. H. Wang Y. Y. Wang C. Y. Zi H. Weng H. (2019). Periodontal disease and risk of benign prostate hyperplasia: a cross-sectional study. Mil. Med. Res. 6 (1), 34. Epub 2019/11/14. 10.1186/s40779-019-0223-8 31718713 PMC6852712

[B60] Wu Z. Byrd D. A. Wan Y. Ansong D. Clegg-Lamptey J. N. Wiafe-Addai B. (2022). The oral microbiome and breast cancer and nonmalignant breast disease, and its relationship with the fecal microbiome in the Ghana breast health study. Int. J. Cancer 151 (8), 1248–1260. Epub 20220630. 10.1002/ijc.34145 35657343 PMC9420782

[B61] Xie Y. Xie F. Zhou X. Zhang L. Yang B. Huang J. (2022). Microbiota in tumors: from understanding to application. Adv. Sci. (Weinh) 9 (21), e2200470. Epub 2022/05/24. 10.1002/advs.202200470 35603968 PMC9313476

[B62] Yan X. Yang M. Liu J. Gao R. Hu J. Li J. (2015). Discovery and validation of potential bacterial biomarkers for lung cancer. Am. J. Cancer Res. 5 (10), 3111–3122. Epub 20150915. 26693063 PMC4656734

[B63] Yu L. Maishi N. Akahori E. Hasebe A. Takeda R. Matsuda A. Y. (2022). The oral bacterium Streptococcus mutans promotes tumor metastasis by inducing vascular inflammation. Cancer Sci. 113 (11), 3980–3994. Epub 20220912. 10.1111/cas.15538 35997541 PMC9633306

[B64] Yu T. Guo F. Yu Y. Sun T. Ma D. Han J. (2017). Fusobacterium nucleatum promotes chemoresistance to colorectal cancer by modulating autophagy. Cell 170 (3), 548–563. Epub 2017/07/29. 10.1016/j.cell.2017.07.008 28753429 PMC5767127

[B65] Zhang C. Hu A. Li J. Zhang F. Zhong P. Li Y. (2022). Combined non-invasive prediction and new biomarkers of oral and fecal microbiota in patients with gastric and colorectal cancer. Front. Cell Infect. Microbiol. 12, 830684. Epub 2022/06/07. 10.3389/fcimb.2022.830684 35663463 PMC9161364

[B66] Zhang W. Luo J. Dong X. Zhao S. Hao Y. Peng C. (2019). Salivary microbial dysbiosis is associated with systemic inflammatory markers and predicted oral metabolites in non-small cell lung cancer patients. J. Cancer 10 (7), 1651–1662. Epub 2019/06/18. 10.7150/jca.28077 31205521 PMC6548009

[B67] Zhao L. Zhang X. Zhou Y. Fu K. Lau H. C. Chun T. W. (2022). Parvimonas micra promotes colorectal tumorigenesis and is associated with prognosis of colorectal cancer patients. Oncogene 41 (36), 4200–4210. Epub 2022/07/27. 10.1038/s41388-022-02395-7 35882981 PMC9439953

[B68] Zhou B. Sun C. Huang J. Xia M. Guo E. Li N. (2019). The biodiversity composition of microbiome in ovarian carcinoma patients. Sci. Rep. 9 (1), 1691. Epub 2019/02/10. 10.1038/s41598-018-38031-2 30737418 PMC6368644

