# Supplementary material for: Association between oral microbiome and seven types of cancers in East Asian population: a two-sample Mendelian randomization analysis
Source: Front Mol Biosci. 2023 Nov 21;10:1327893. doi: 10.3389/fmolb.2023.1327893 (PMC10702768; doi:10.3389/fmolb.2023.1327893)
Supplement: Supplementary file 1 [file DataSheet1.ZIP › Supplementary Materials/MR plots for tongue/tongue═╝/Colorectal cancer/pheno.1821_to_colorectal cancer_leave_one_out.pdf]

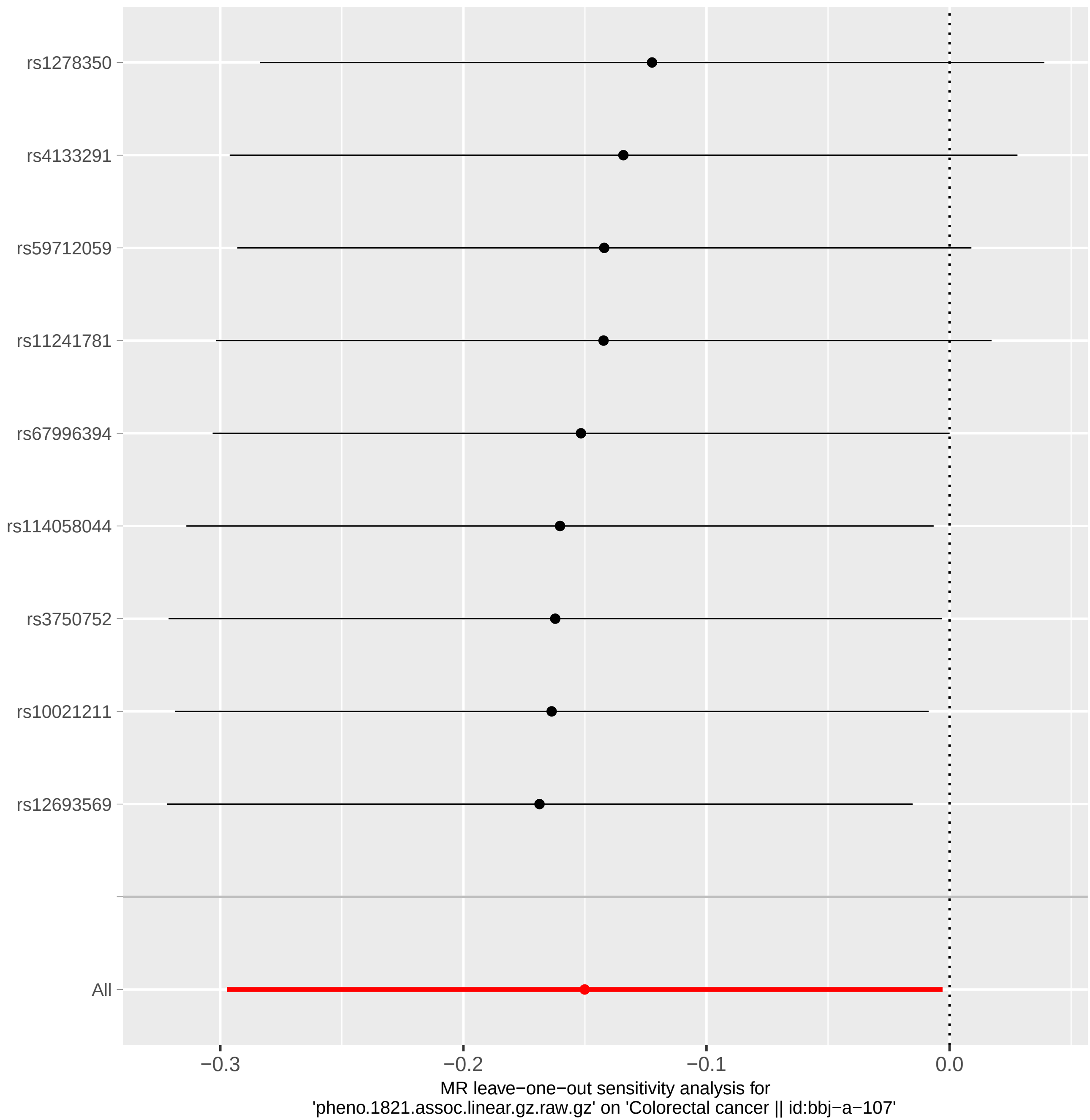

rs1278350
rs4133291
rs59712059
rs11241781
rs67996394
rs114058044
rs3750752
rs10021211
rs12693569
All
−0.3
−0.2
−0.1
0.0
MR leave−one−out sensitivity analysis for
'pheno.1821.assoc.linear.gz.raw.gz' on 'Colorectal cancer || id:bbj−a−107'
