# Supplementary material for: Association between oral microbiome and seven types of cancers in East Asian population: a two-sample Mendelian randomization analysis
Source: Front Mol Biosci. 2023 Nov 21;10:1327893. doi: 10.3389/fmolb.2023.1327893 (PMC10702768; doi:10.3389/fmolb.2023.1327893)
Supplement: Supplementary file 1 [file DataSheet1.ZIP › Supplementary Materials/MR plots for tongue/tongue═╝/Colorectal cancer/pheno.1857_to_colorectal cancer_leave_one_out.pdf]

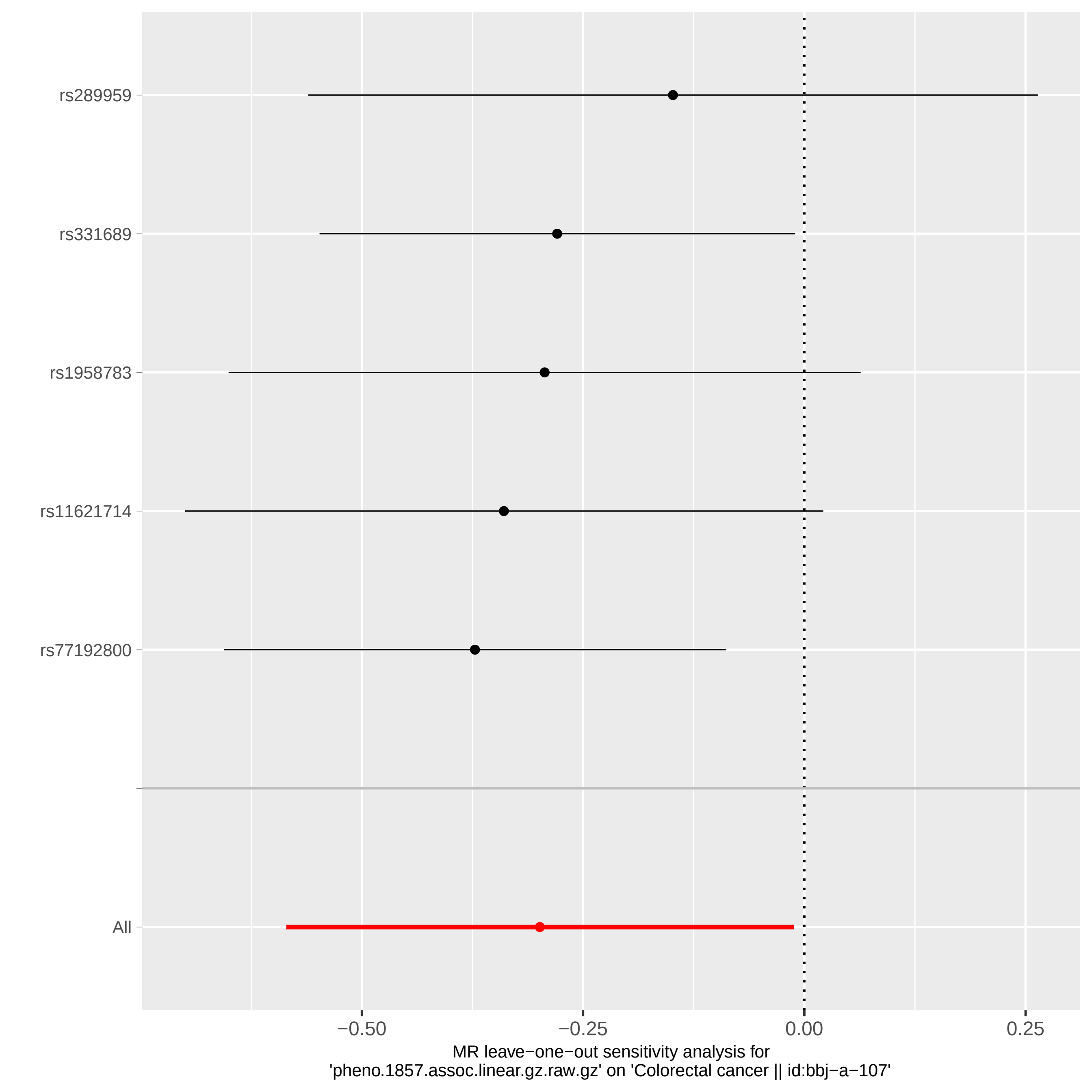
rs289959
rs331689
rs1958783
rs11621714
rs77192800
All
−0.50
−0.25
0.00
0.25
MR leave−one−out sensitivity analysis for
'pheno.1857.assoc.linear.gz.raw.gz' on 'Colorectal cancer || id:bbj−a−107'
