# Supplementary material for: Association between oral microbiome and seven types of cancers in East Asian population: a two-sample Mendelian randomization analysis
Source: Front Mol Biosci. 2023 Nov 21;10:1327893. doi: 10.3389/fmolb.2023.1327893 (PMC10702768; doi:10.3389/fmolb.2023.1327893)
Supplement: Supplementary file 1 [file DataSheet1.ZIP › Supplementary Materials/MR plots for tongue/tongue═╝/Colorectal cancer/pheno.2173_to_colorectal cancer_leave_one_out.pdf]

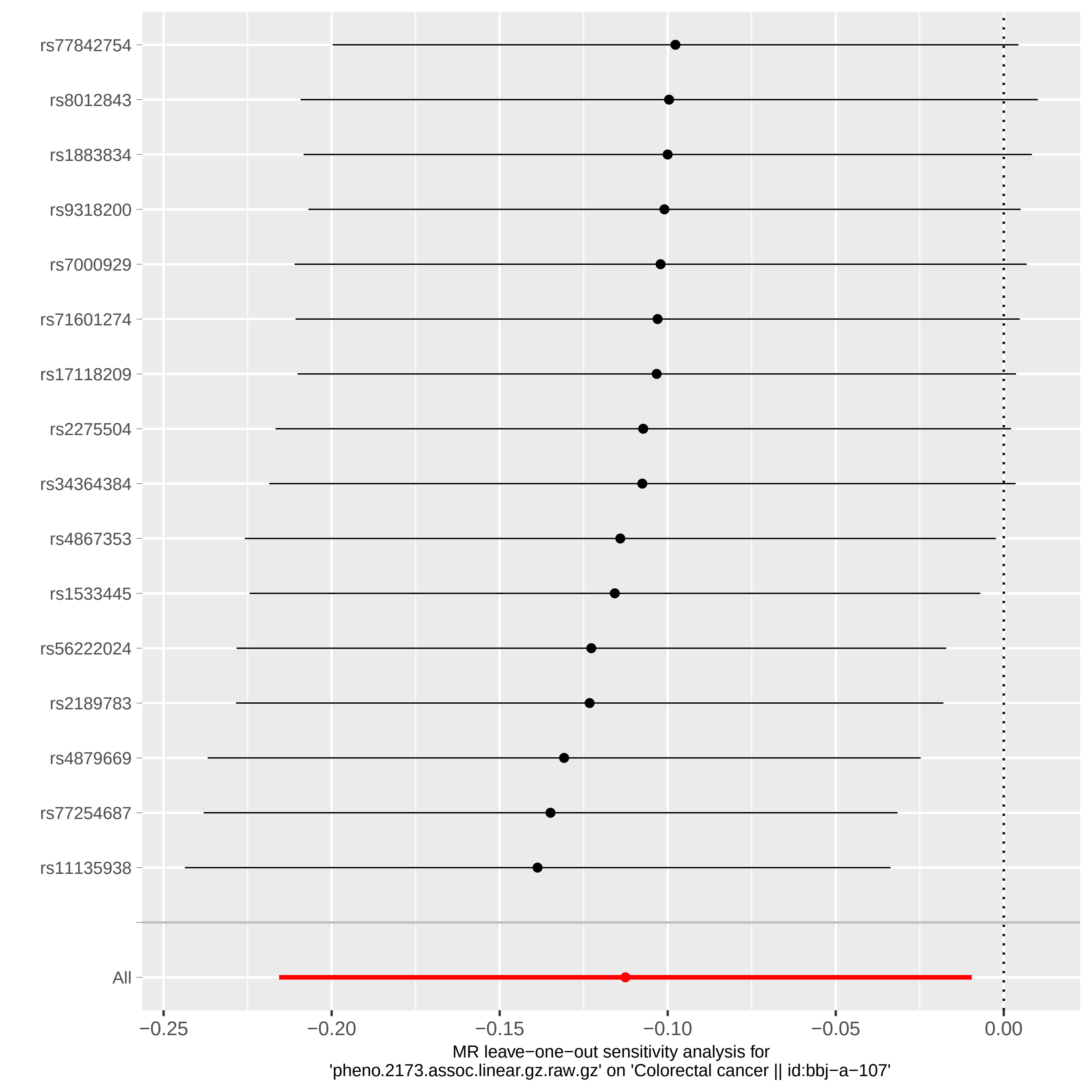
rs77842754
rs8012843
rs1883834
rs9318200
rs7000929
rs71601274
rs17118209
rs2275504
rs34364384
rs4867353
rs1533445
rs56222024
rs2189783
rs4879669
rs77254687
rs11135938
All
−0.25
−0.20
−0.15
−0.10
−0.05
0.00
MR leave−one−out sensitivity analysis for
'pheno.2173.assoc.linear.gz.raw.gz' on 'Colorectal cancer || id:bbj−a−107'
