# Supplementary figures and images for: Association between oral microbiome and seven types of cancers in East Asian population: a two-sample Mendelian randomization analysis (part 2 of 2)
Source: Front Mol Biosci. 2023 Nov 21;10:1327893. doi: 10.3389/fmolb.2023.1327893 (PMC10702768; doi:10.3389/fmolb.2023.1327893)

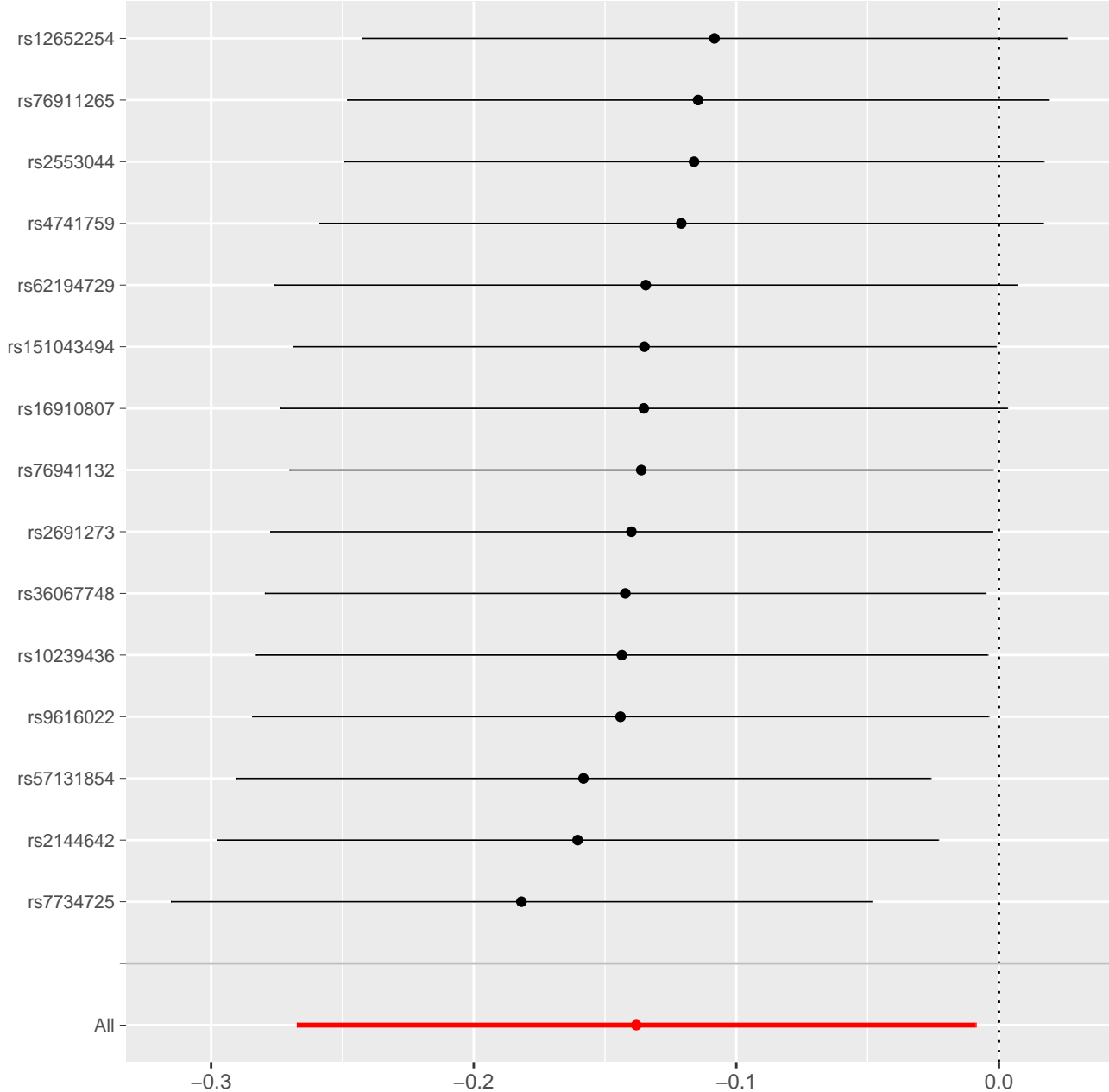

MR leave-one-out sensitivity analysis for  
'pheno.3116.assoc.linear.gz.raw.gz' on 'Breast cancer || id:bbj-a-160'

Supplement: Supplementary file 1 [file DataSheet1.ZIP › Supplementary Materials/MR plots for tongue/tongue═╝/Breast cancer/pheno.3116_to_breast cancer_leave_one_out.pdf]

# MR Test

- Inverse variance weighted
- MR Egger
- Simple mode
- Weighted median
- Weighted mode

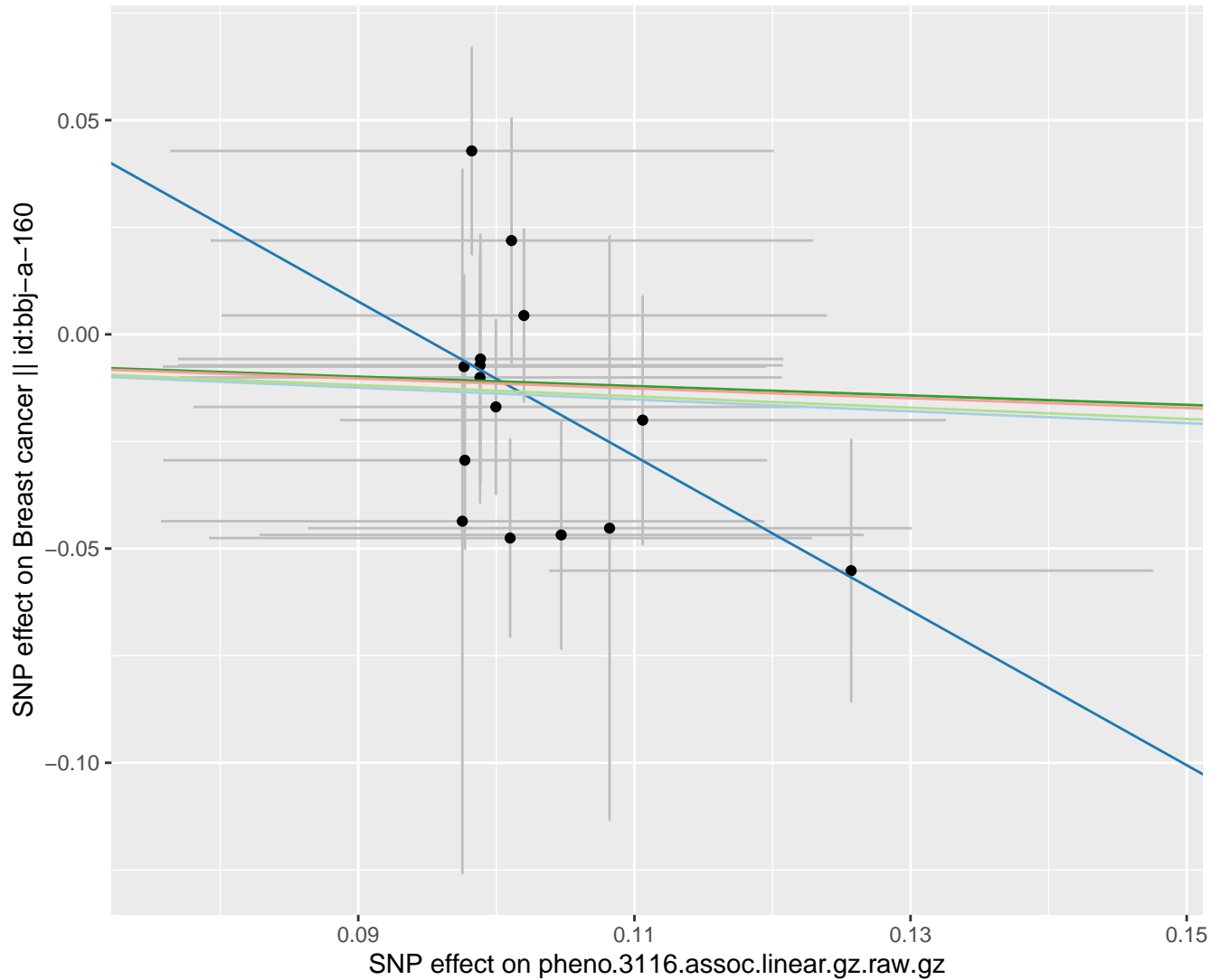

Supplement: Supplementary file 1 [file DataSheet1.ZIP › Supplementary Materials/MR plots for tongue/tongue═╝/Breast cancer/pheno.3116_to_breast cancer_scatter.pdf]

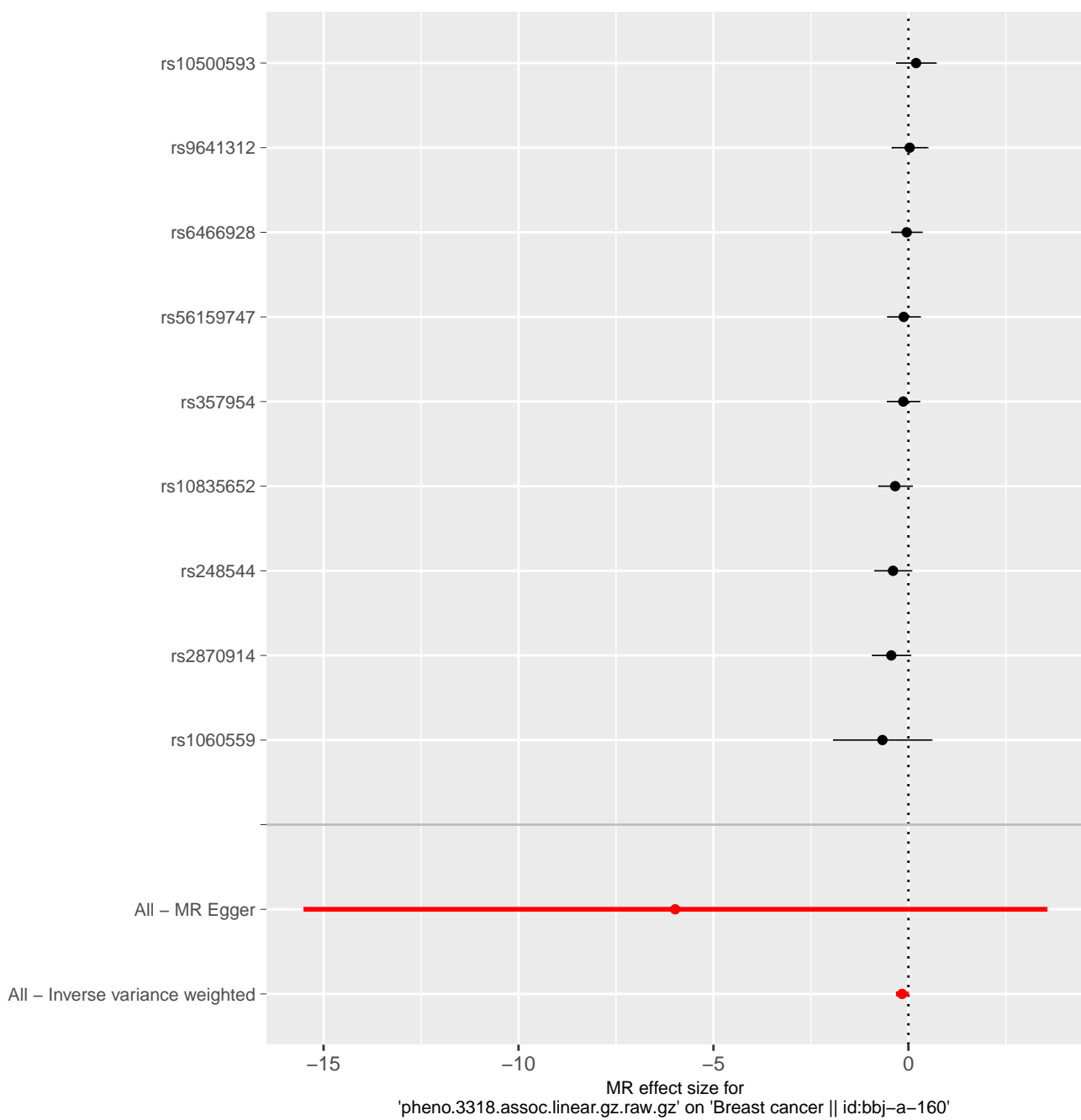

Supplement: Supplementary file 1 [file DataSheet1.ZIP › Supplementary Materials/MR plots for tongue/tongue═╝/Breast cancer/pheno.3318_to_breast cancer_forest.pdf]

# MR Method

- Inverse variance weighted
- MR Egger

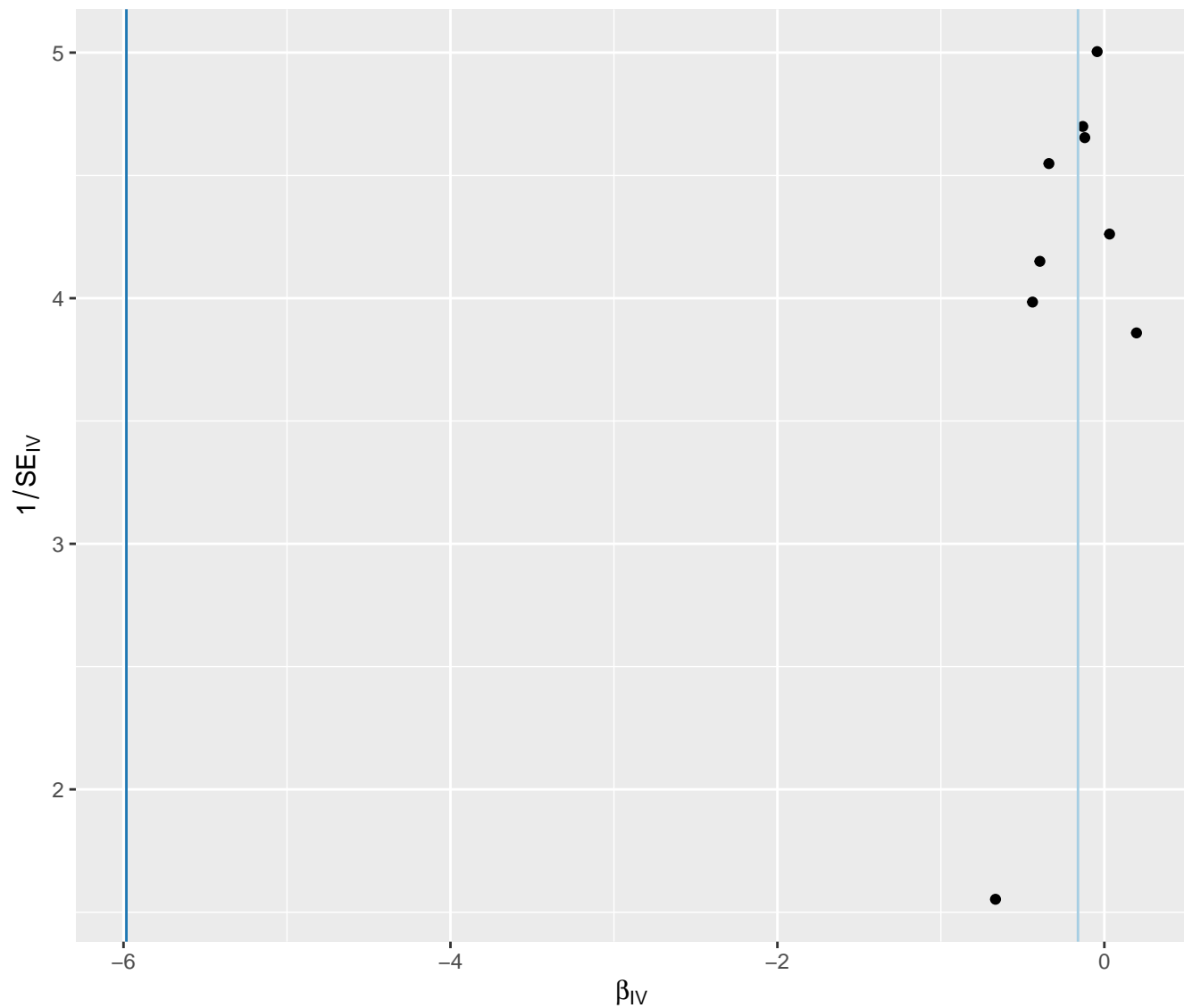

Supplement: Supplementary file 1 [file DataSheet1.ZIP › Supplementary Materials/MR plots for tongue/tongue═╝/Breast cancer/pheno.3318_to_breast cancer_funnel.pdf]

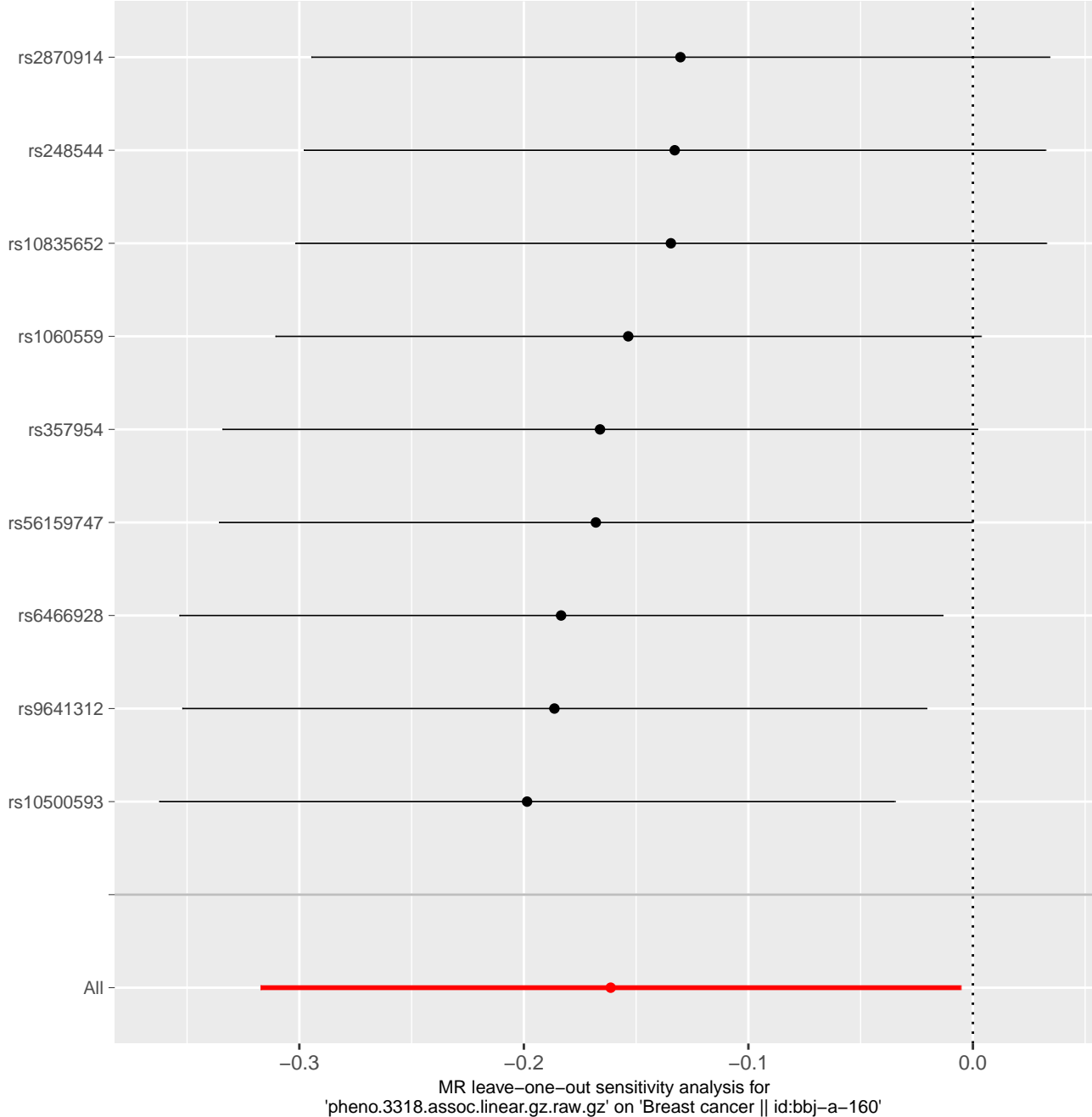

Supplement: Supplementary file 1 [file DataSheet1.ZIP › Supplementary Materials/MR plots for tongue/tongue═╝/Breast cancer/pheno.3318_to_breast cancer_leave_one_out.pdf]

# MR Test

- Inverse variance weighted
- MR Egger
- Simple mode
- Weighted median
- Weighted mode

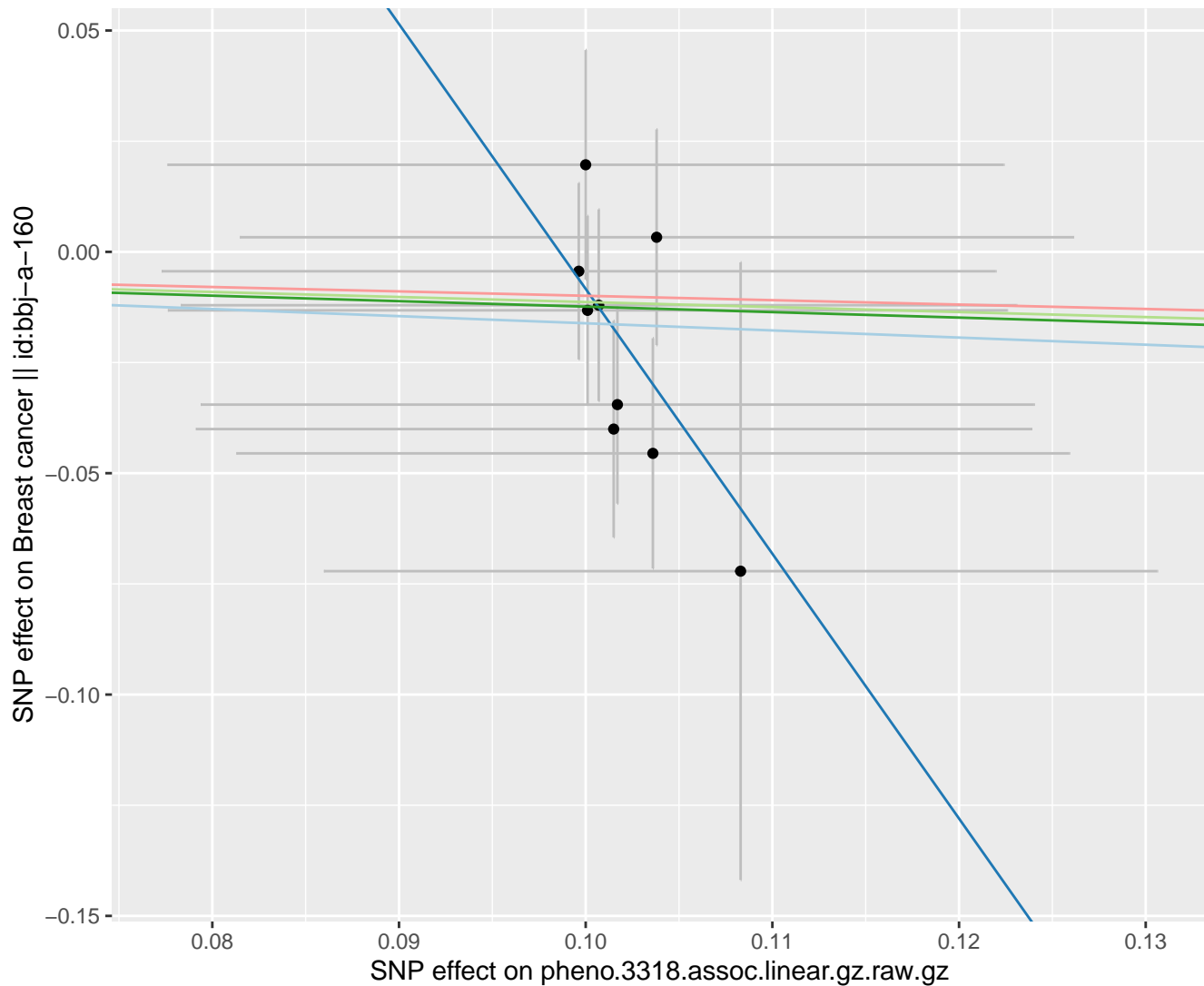

Supplement: Supplementary file 1 [file DataSheet1.ZIP › Supplementary Materials/MR plots for tongue/tongue═╝/Breast cancer/pheno.3318_to_breast cancer_scatter.pdf]

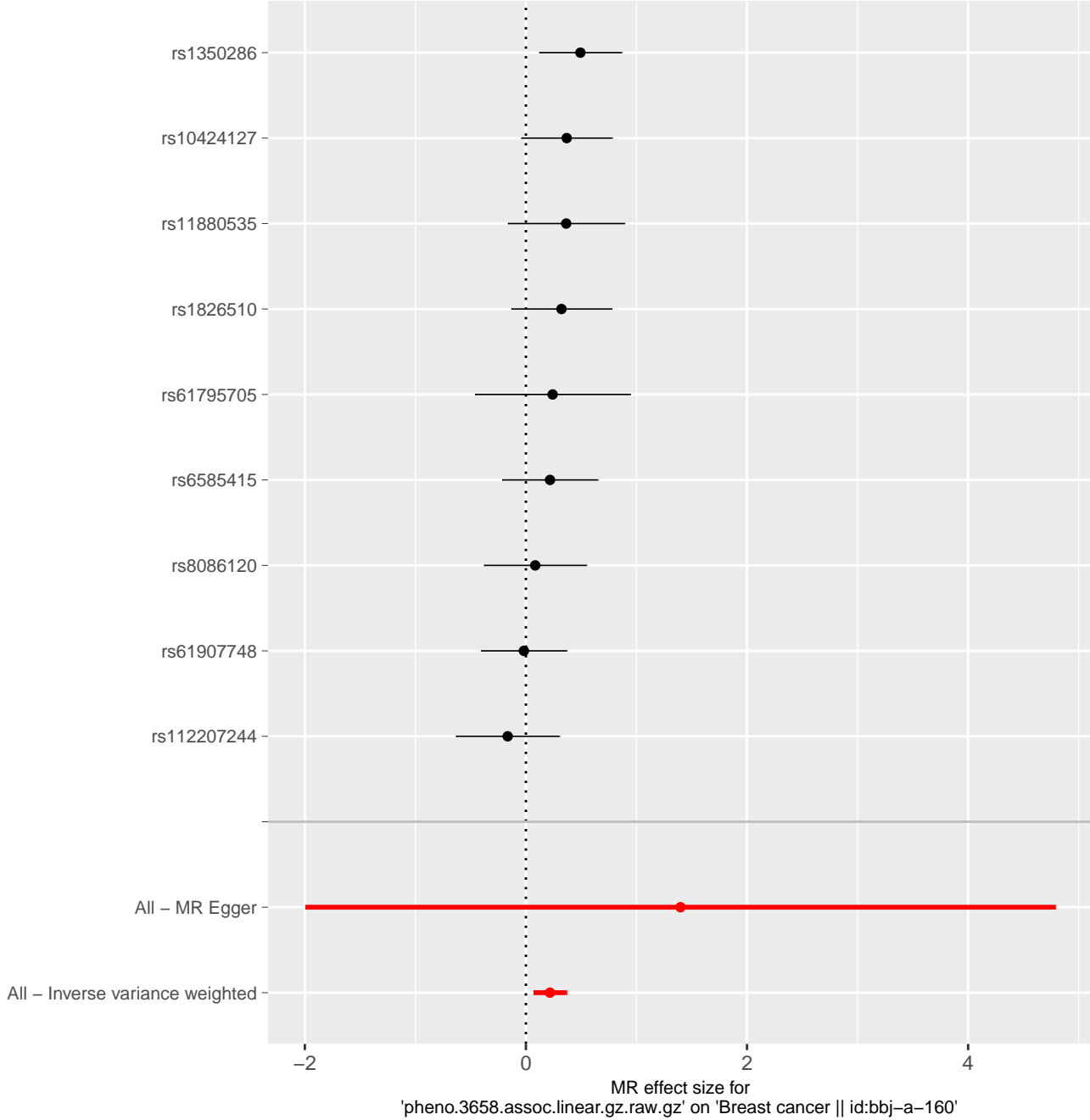

Supplement: Supplementary file 1 [file DataSheet1.ZIP › Supplementary Materials/MR plots for tongue/tongue═╝/Breast cancer/pheno.3658_to_breast cancer_forest.pdf]

# MR Method

- Inverse variance weighted
- MR Egger

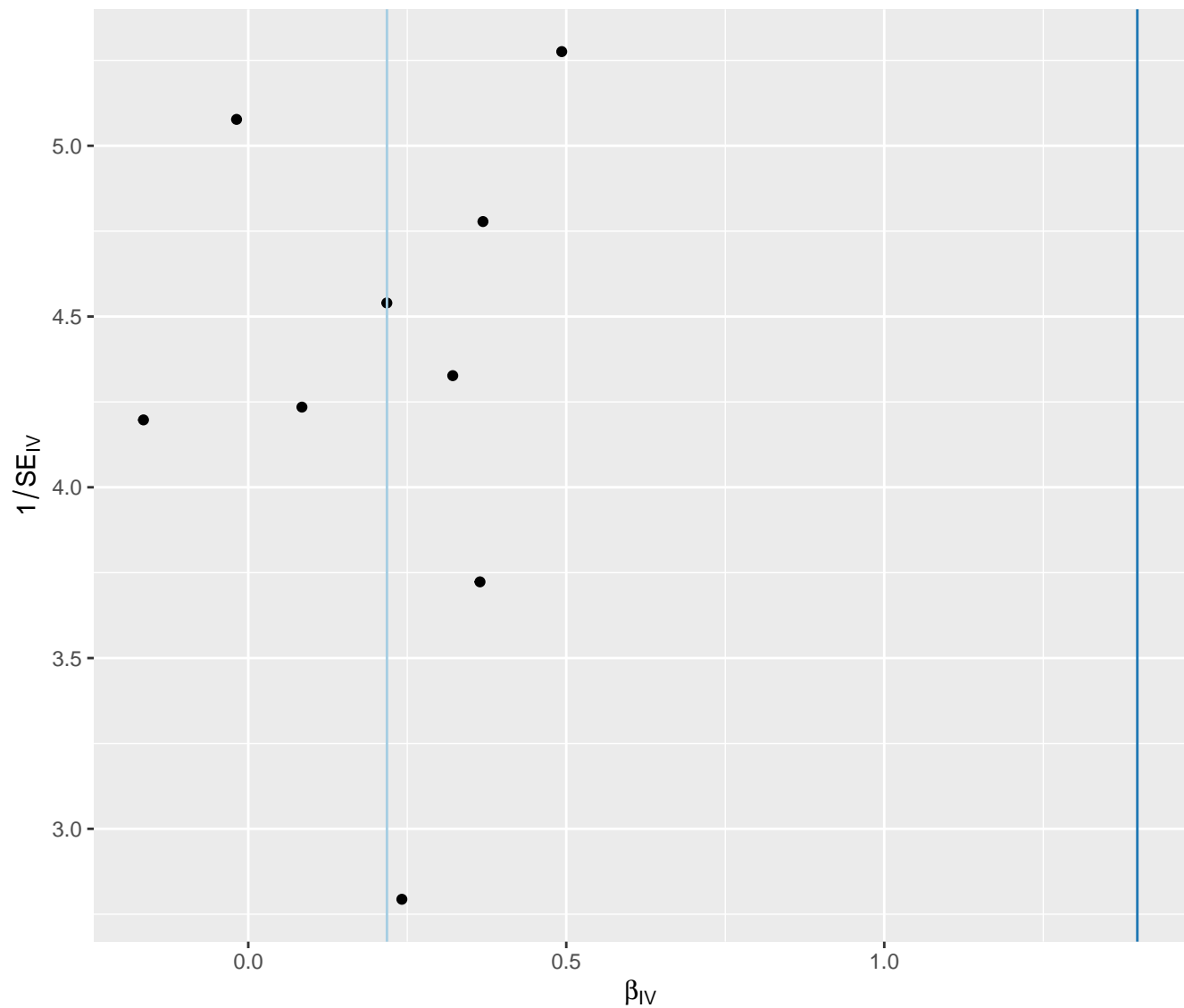

Supplement: Supplementary file 1 [file DataSheet1.ZIP › Supplementary Materials/MR plots for tongue/tongue═╝/Breast cancer/pheno.3658_to_breast cancer_funnel.pdf]

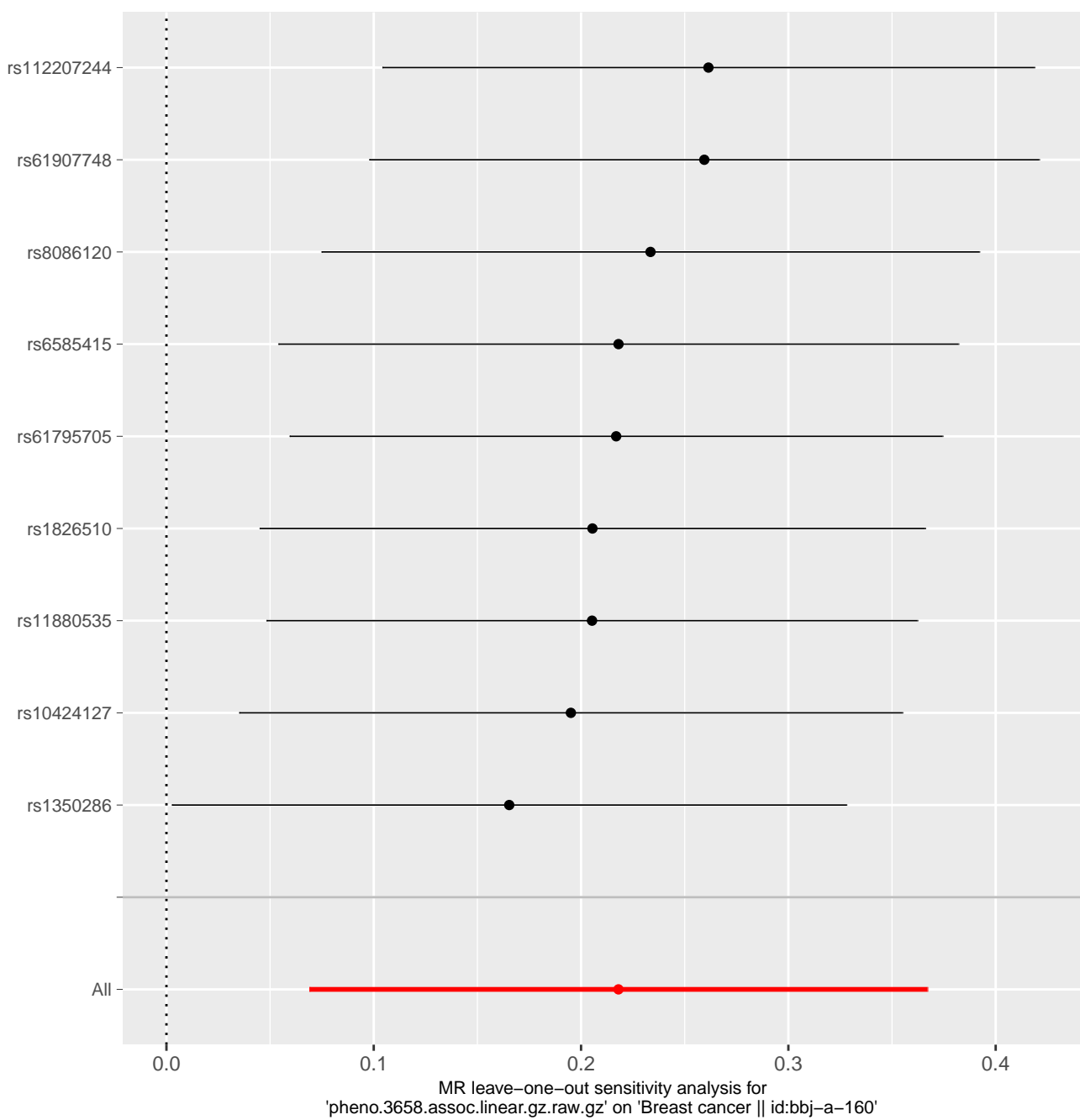

Supplement: Supplementary file 1 [file DataSheet1.ZIP › Supplementary Materials/MR plots for tongue/tongue═╝/Breast cancer/pheno.3658_to_breast cancer_leave_one_out.pdf]

# MR Test

- Inverse variance weighted
- MR Egger
- Simple mode
- Weighted median
- Weighted mode

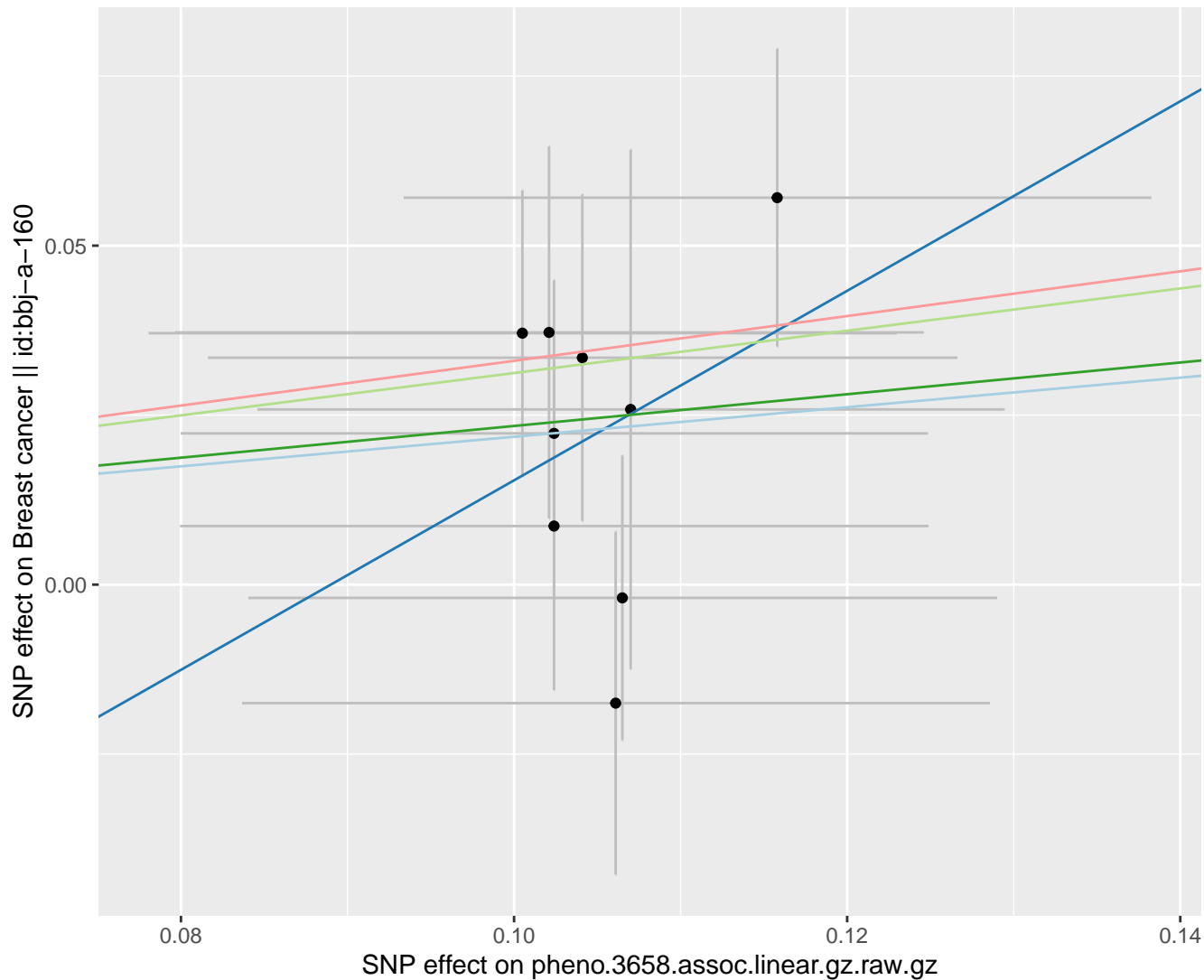

Supplement: Supplementary file 1 [file DataSheet1.ZIP › Supplementary Materials/MR plots for tongue/tongue═╝/Breast cancer/pheno.3658_to_breast cancer_scatter.pdf]

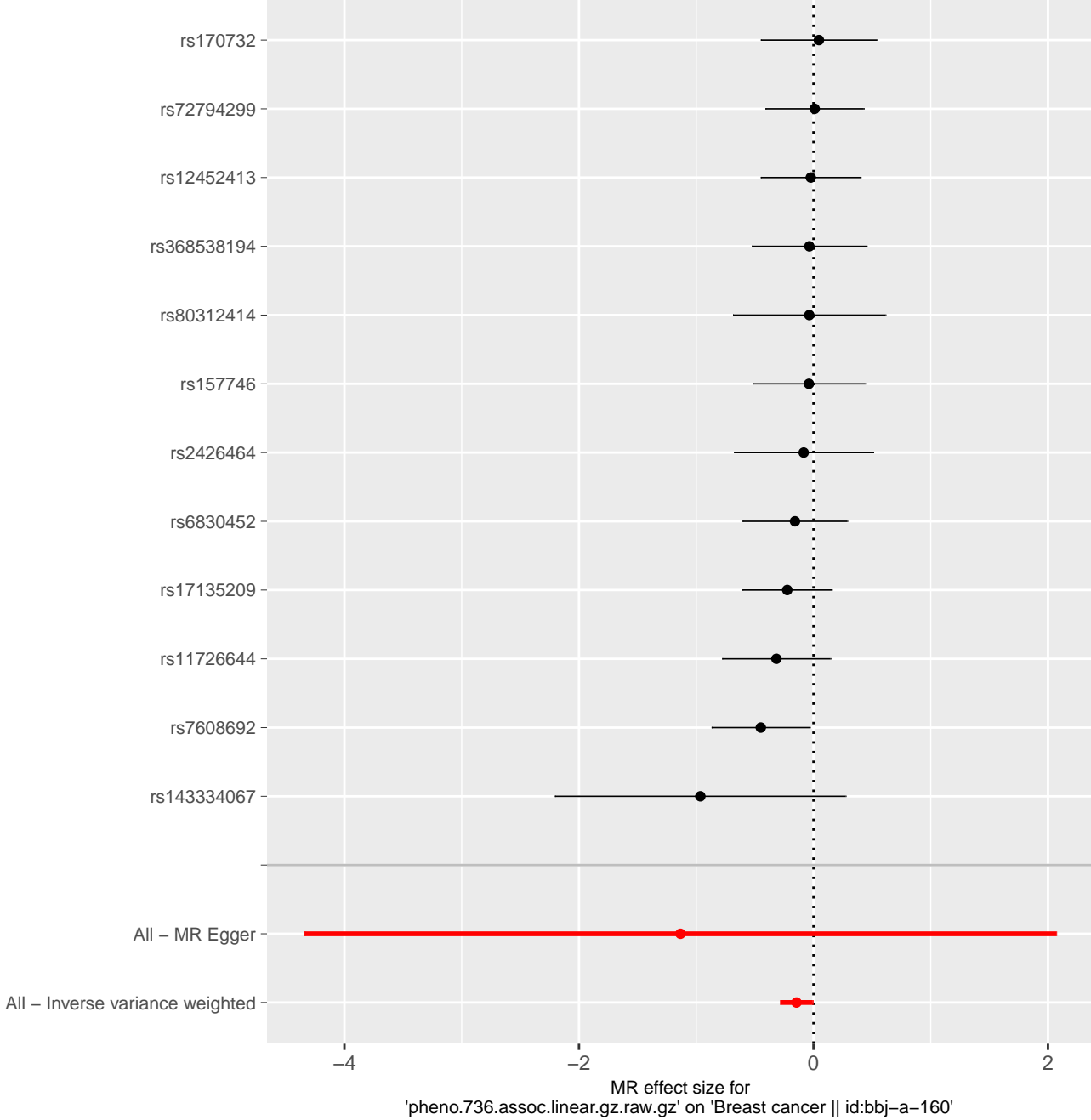

Supplement: Supplementary file 1 [file DataSheet1.ZIP › Supplementary Materials/MR plots for tongue/tongue═╝/Breast cancer/pheno.736_to_breast cancer_forest.pdf]

# MR Method

- Inverse variance weighted
- MR Egger

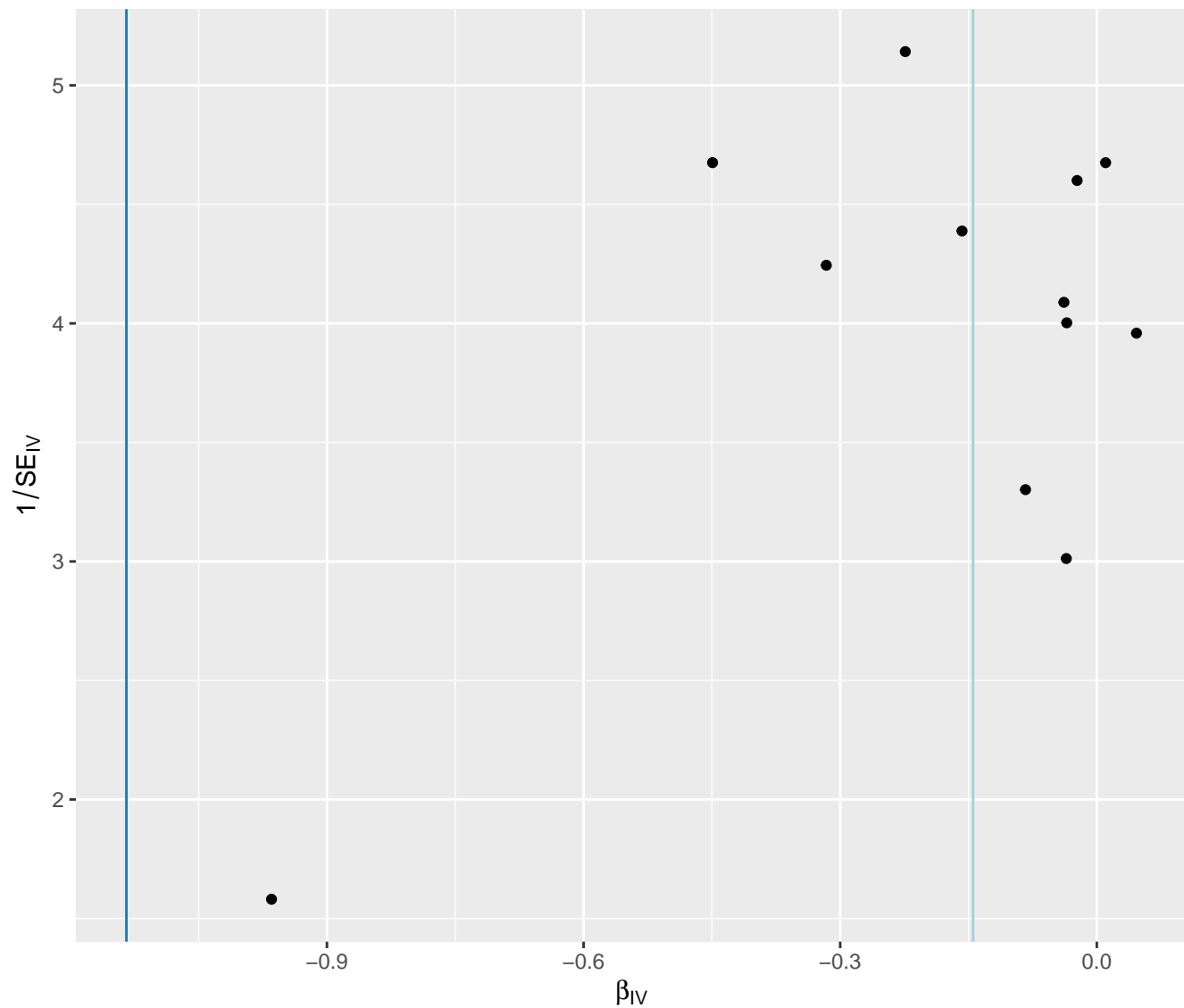

Supplement: Supplementary file 1 [file DataSheet1.ZIP › Supplementary Materials/MR plots for tongue/tongue═╝/Breast cancer/pheno.736_to_breast cancer_funnel.pdf]

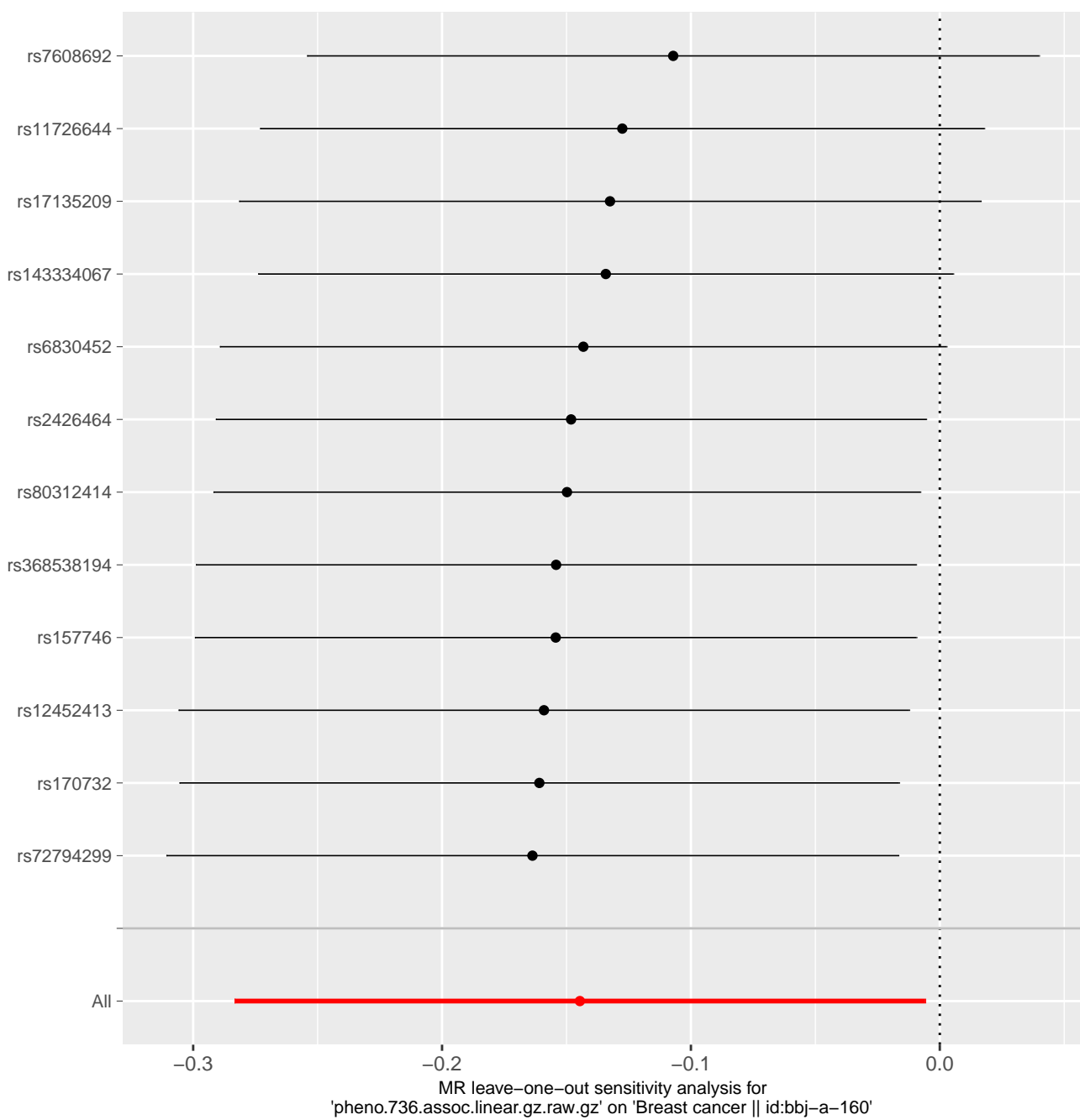

Supplement: Supplementary file 1 [file DataSheet1.ZIP › Supplementary Materials/MR plots for tongue/tongue═╝/Breast cancer/pheno.736_to_breast cancer_leave_one_out.pdf]

# MR Test

- Inverse variance weighted
- MR Egger
- Simple mode
- Weighted median
- Weighted mode

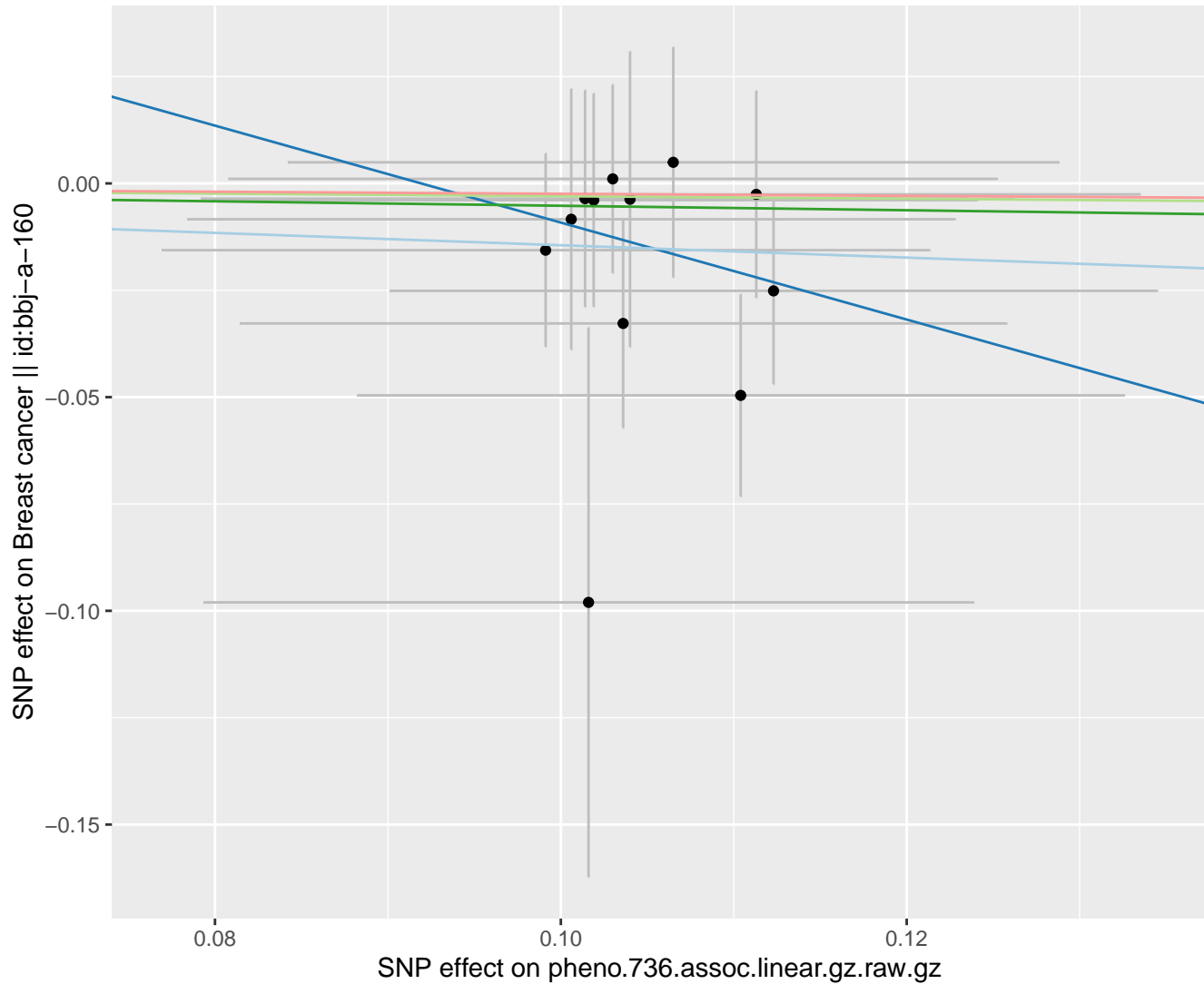

Supplement: Supplementary file 1 [file DataSheet1.ZIP › Supplementary Materials/MR plots for tongue/tongue═╝/Breast cancer/pheno.736_to_breast cancer_scatter.pdf]

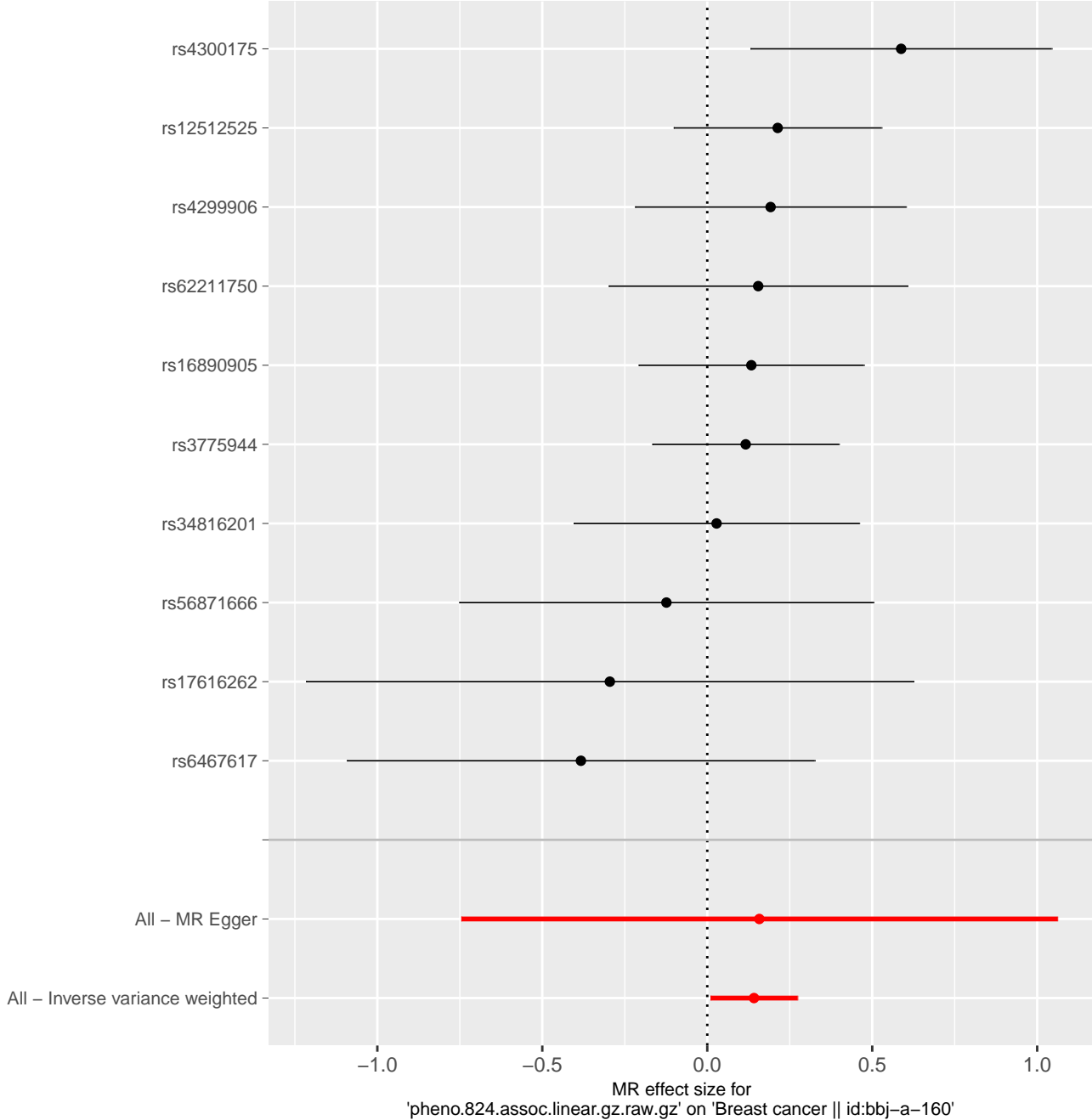

Supplement: Supplementary file 1 [file DataSheet1.ZIP › Supplementary Materials/MR plots for tongue/tongue═╝/Breast cancer/pheno.824_to_breast cancer_forest.pdf]

# MR Method

- Inverse variance weighted
- MR Egger

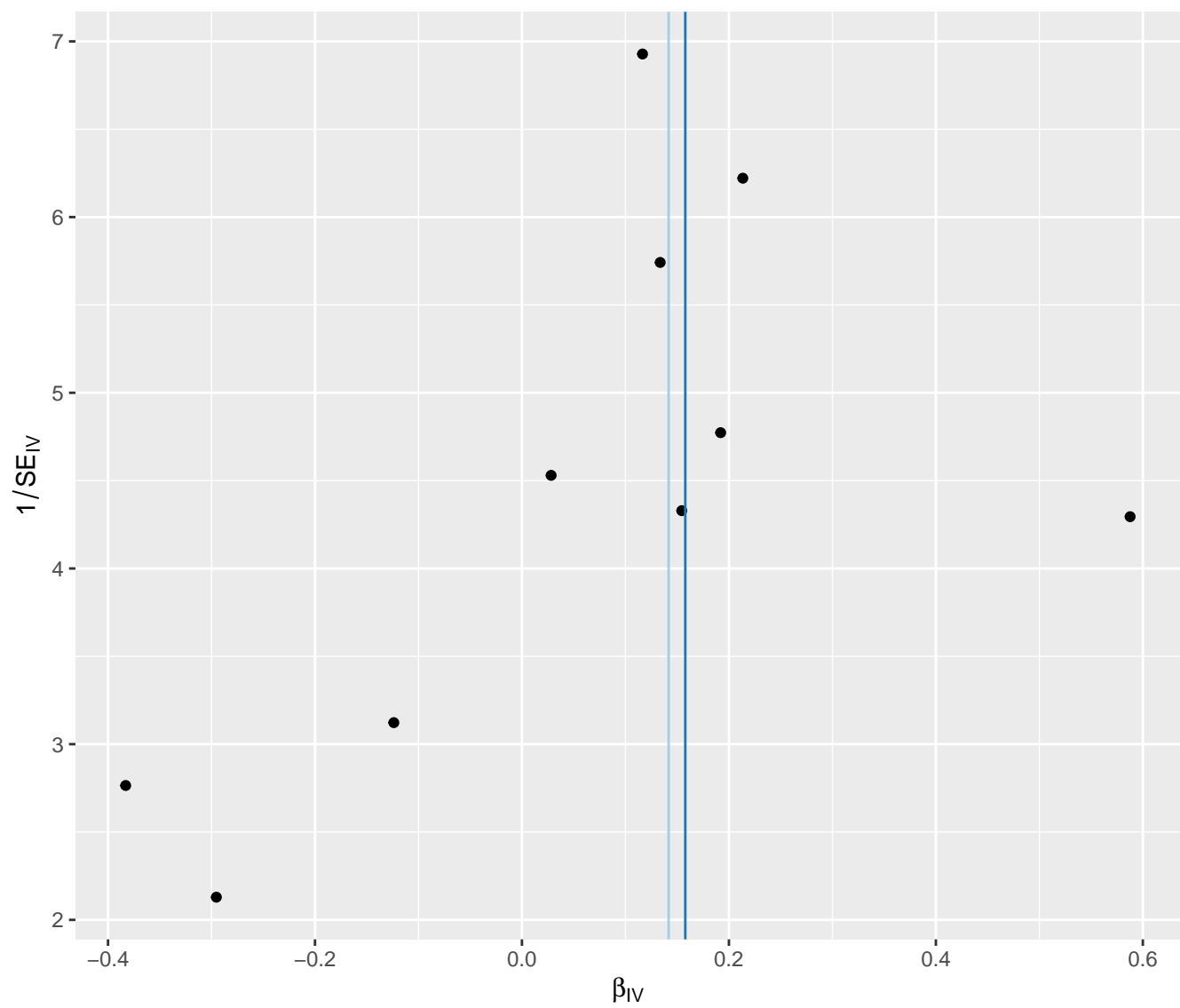

Supplement: Supplementary file 1 [file DataSheet1.ZIP › Supplementary Materials/MR plots for tongue/tongue═╝/Breast cancer/pheno.824_to_breast cancer_funnel.pdf]

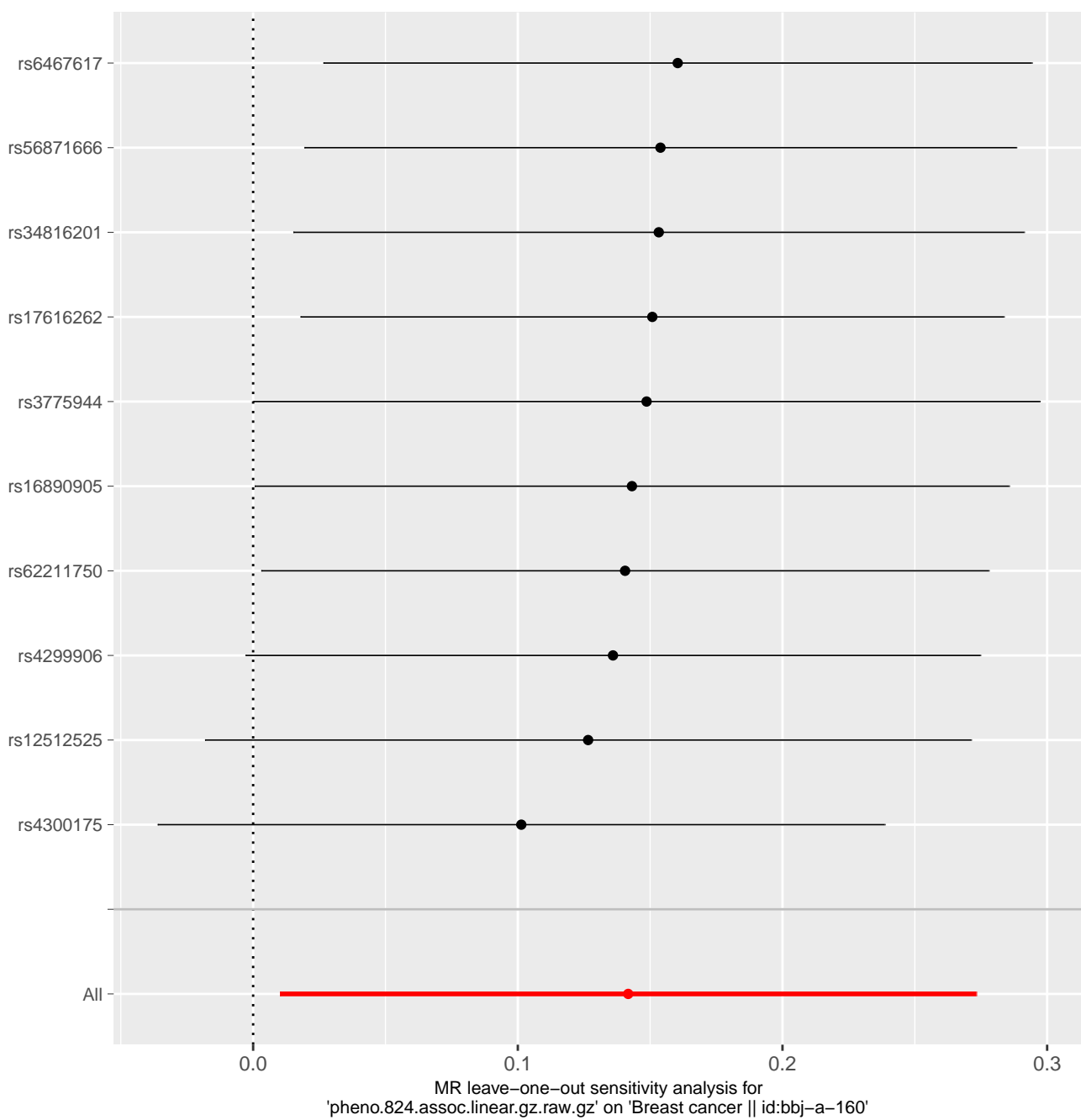

Supplement: Supplementary file 1 [file DataSheet1.ZIP › Supplementary Materials/MR plots for tongue/tongue═╝/Breast cancer/pheno.824_to_breast cancer_leave_one_out.pdf]

# MR Test

- Inverse variance weighted
- MR Egger
- Simple mode
- Weighted median
- Weighted mode

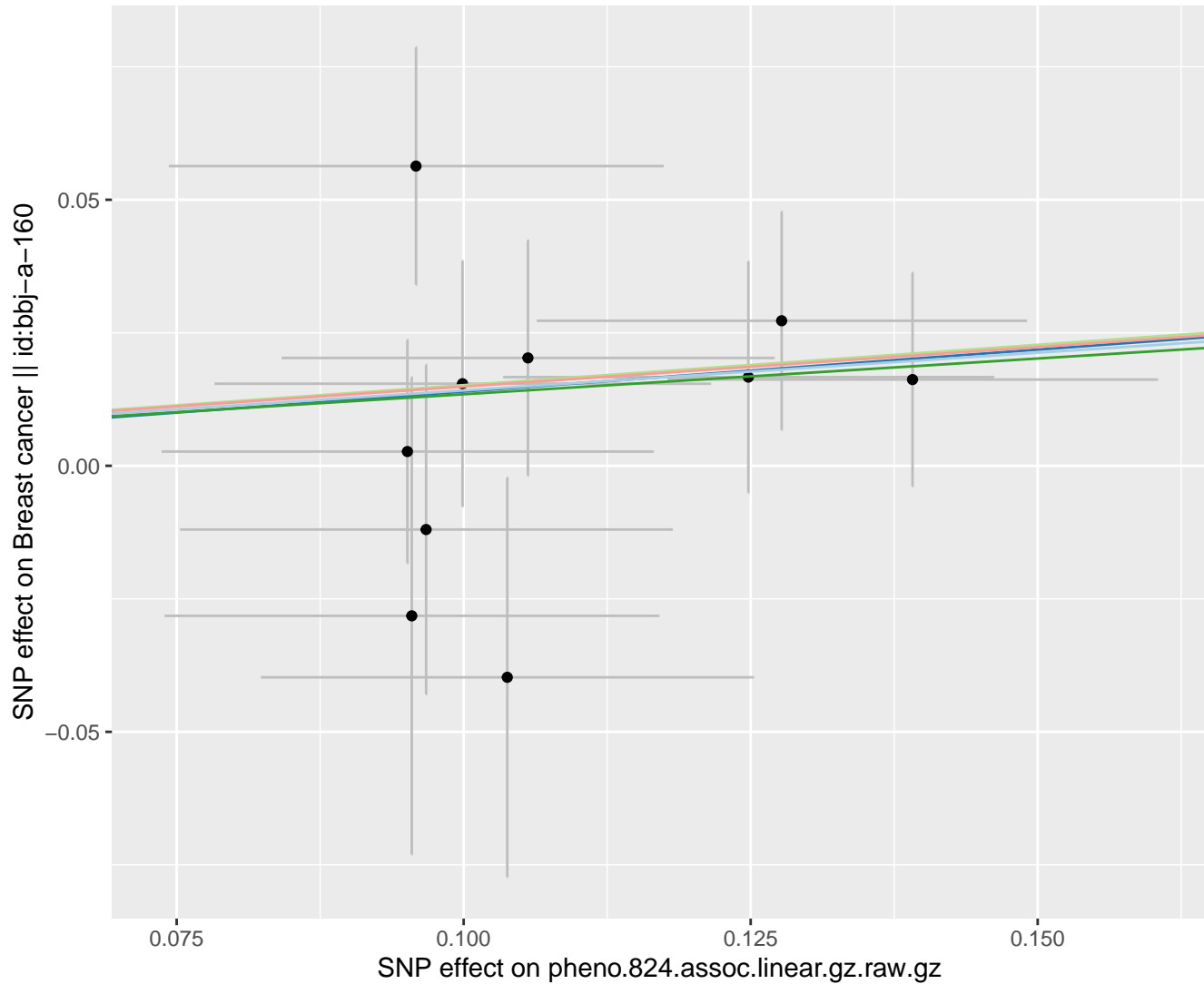

Supplement: Supplementary file 1 [file DataSheet1.ZIP › Supplementary Materials/MR plots for tongue/tongue═╝/Breast cancer/pheno.824_to_breast cancer_scatter.pdf]

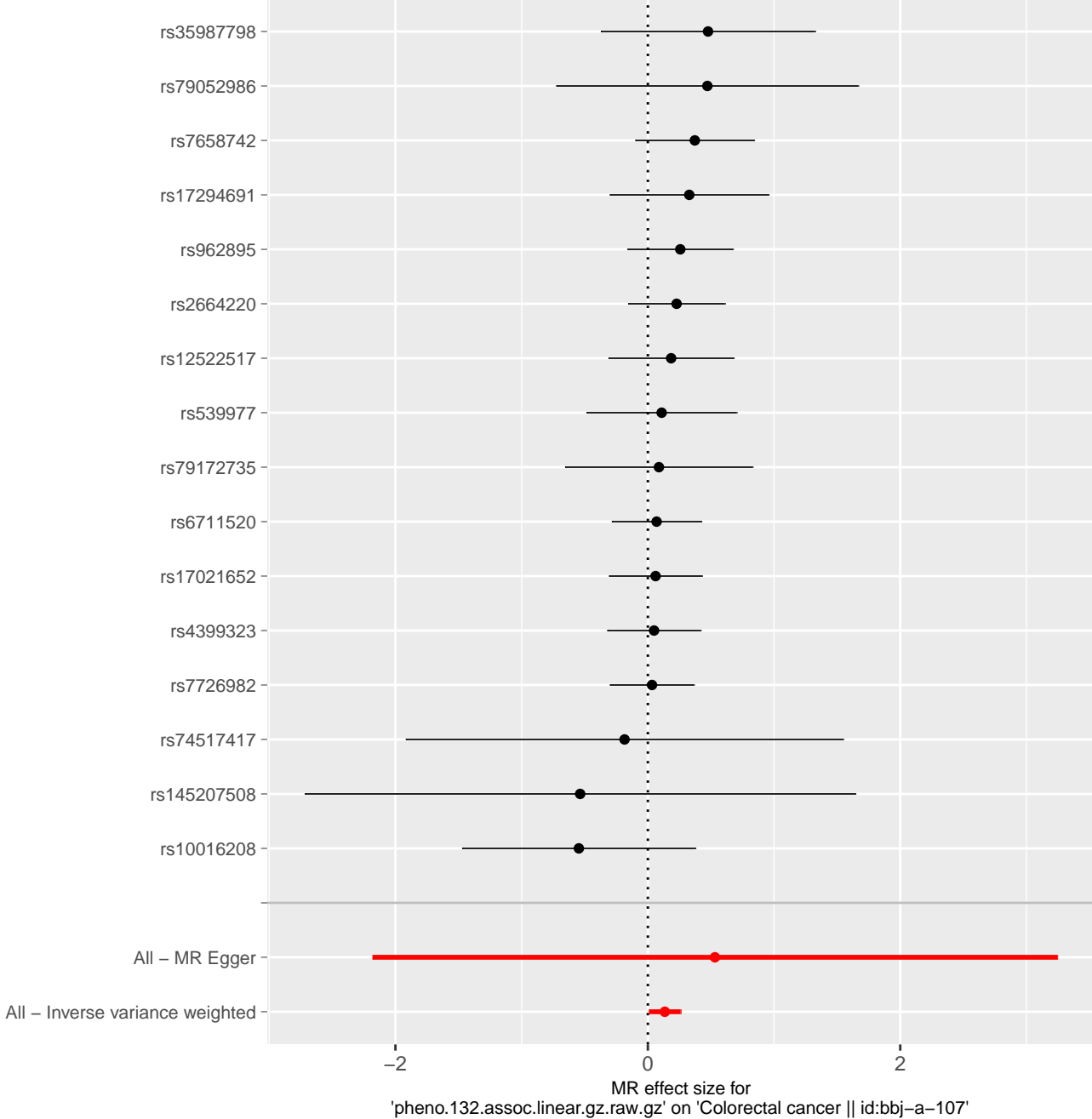

Supplement: Supplementary file 1 [file DataSheet1.ZIP › Supplementary Materials/MR plots for tongue/tongue═╝/Colorectal cancer/pheno.132_to_colorectal cancer_forest.pdf]

# MR Method

- Inverse variance weighted
- MR Egger

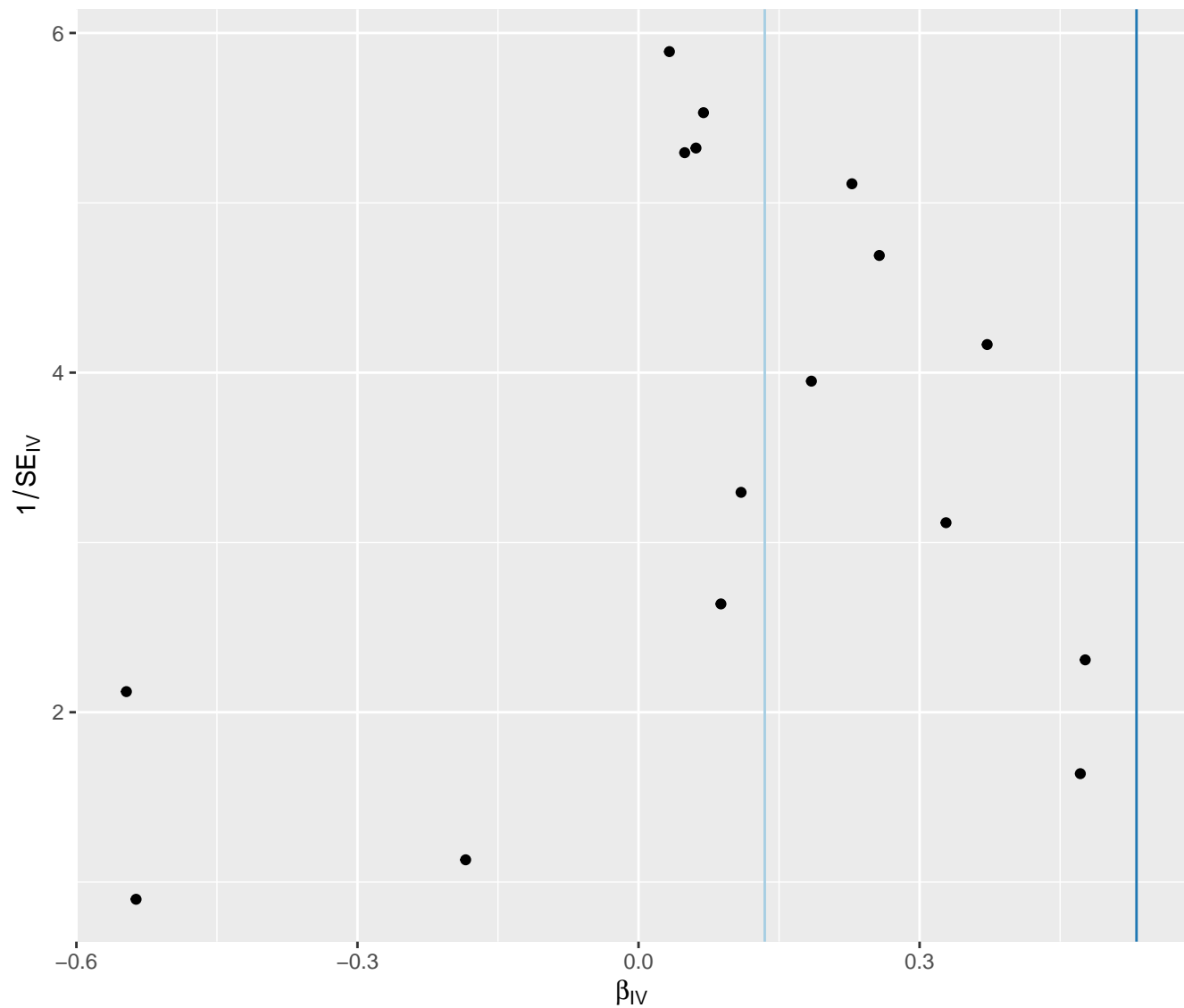

Supplement: Supplementary file 1 [file DataSheet1.ZIP › Supplementary Materials/MR plots for tongue/tongue═╝/Colorectal cancer/pheno.132_to_colorectal cancer_funnel.pdf]

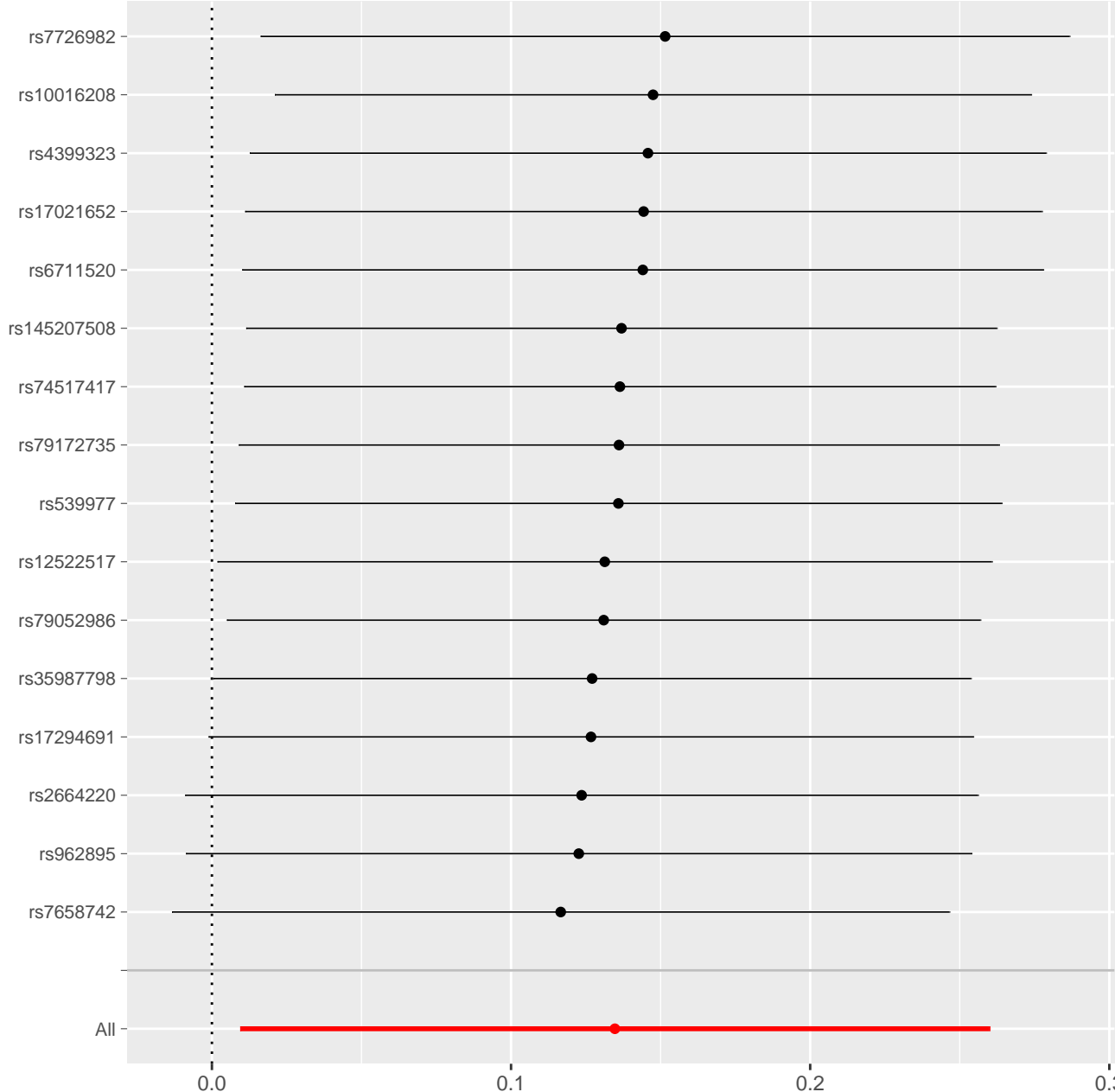

Supplement: Supplementary file 1 [file DataSheet1.ZIP › Supplementary Materials/MR plots for tongue/tongue═╝/Colorectal cancer/pheno.132_to_colorectal cancer_leave_one_out.pdf]

# MR Test

- Inverse variance weighted
- MR Egger
- Simple mode
- Weighted median
- Weighted mode

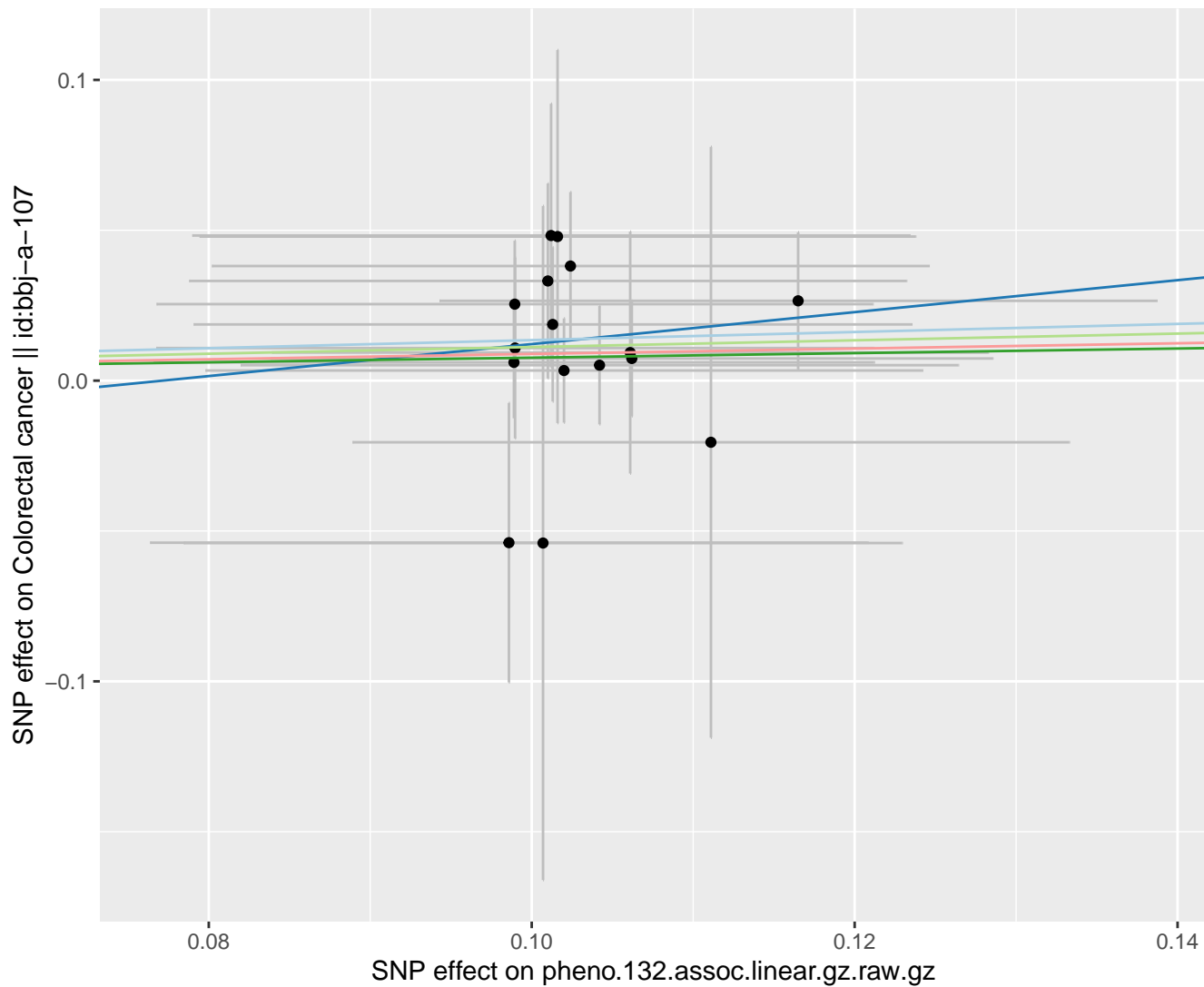

Supplement: Supplementary file 1 [file DataSheet1.ZIP › Supplementary Materials/MR plots for tongue/tongue═╝/Colorectal cancer/pheno.132_to_colorectal cancer_scatter.pdf]

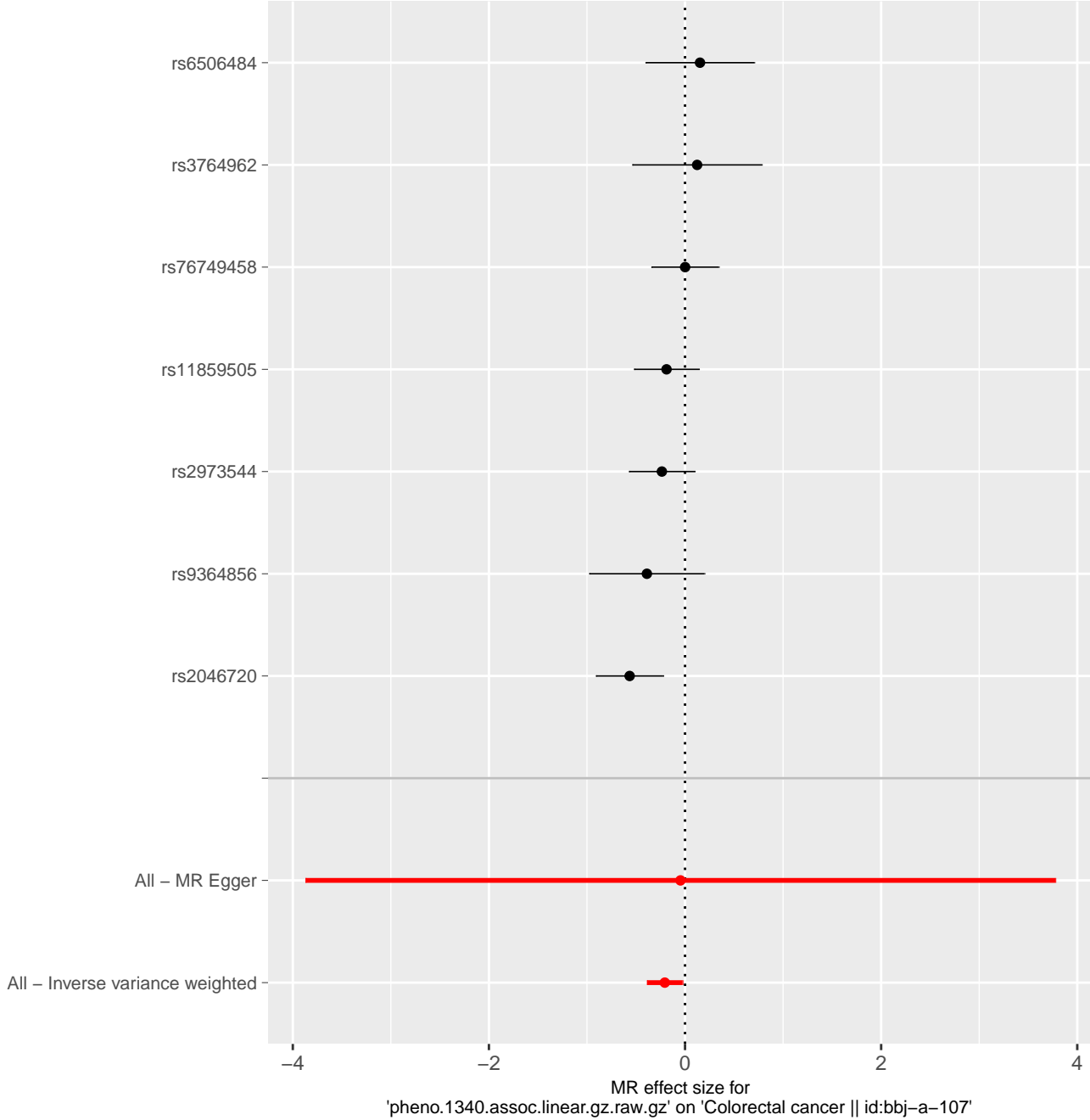

Supplement: Supplementary file 1 [file DataSheet1.ZIP › Supplementary Materials/MR plots for tongue/tongue═╝/Colorectal cancer/pheno.1340_to_colorectal cancer_forest.pdf]

# MR Method

- Inverse variance weighted
- MR Egger

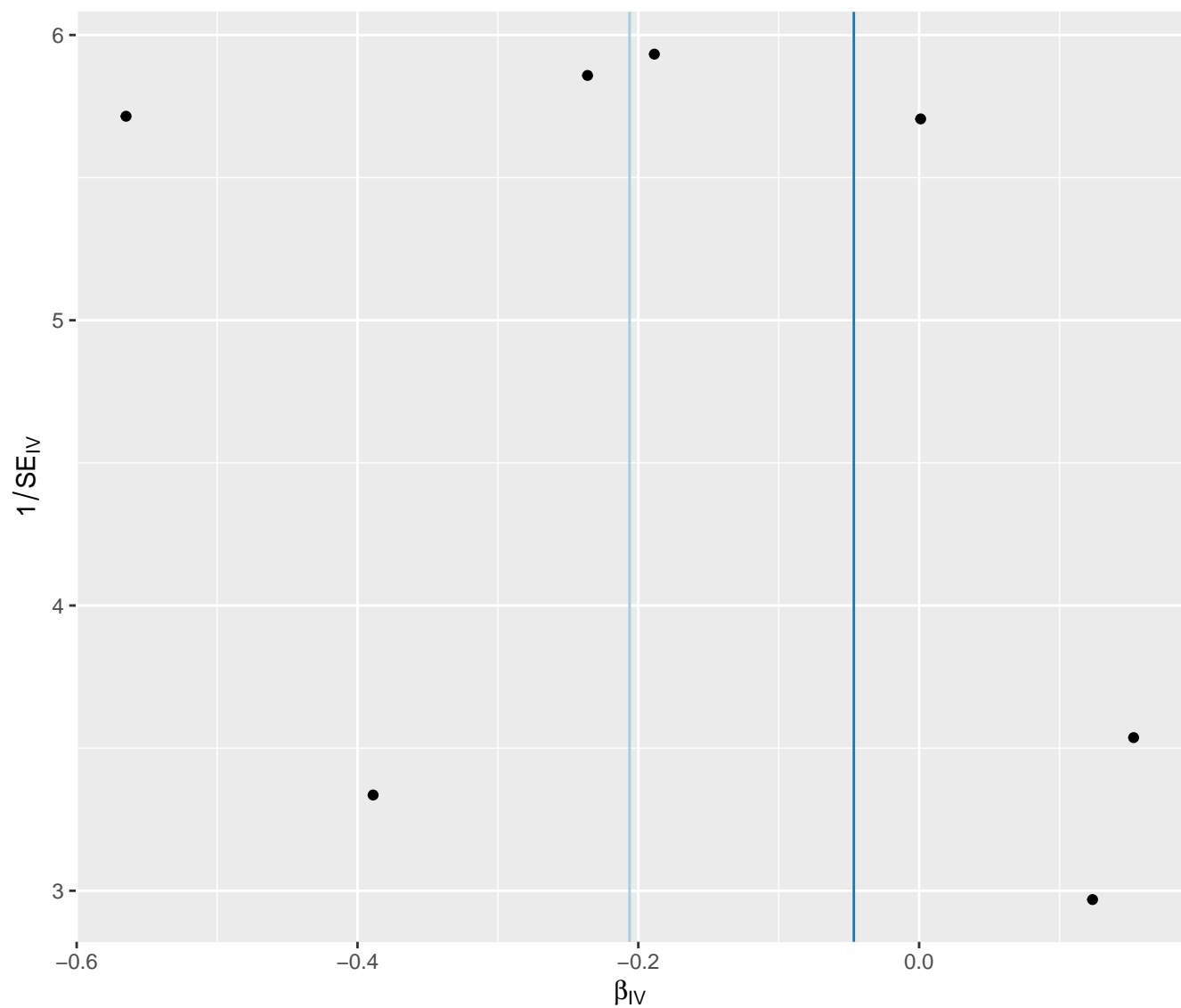

Supplement: Supplementary file 1 [file DataSheet1.ZIP › Supplementary Materials/MR plots for tongue/tongue═╝/Colorectal cancer/pheno.1340_to_colorectal cancer_funnel.pdf]

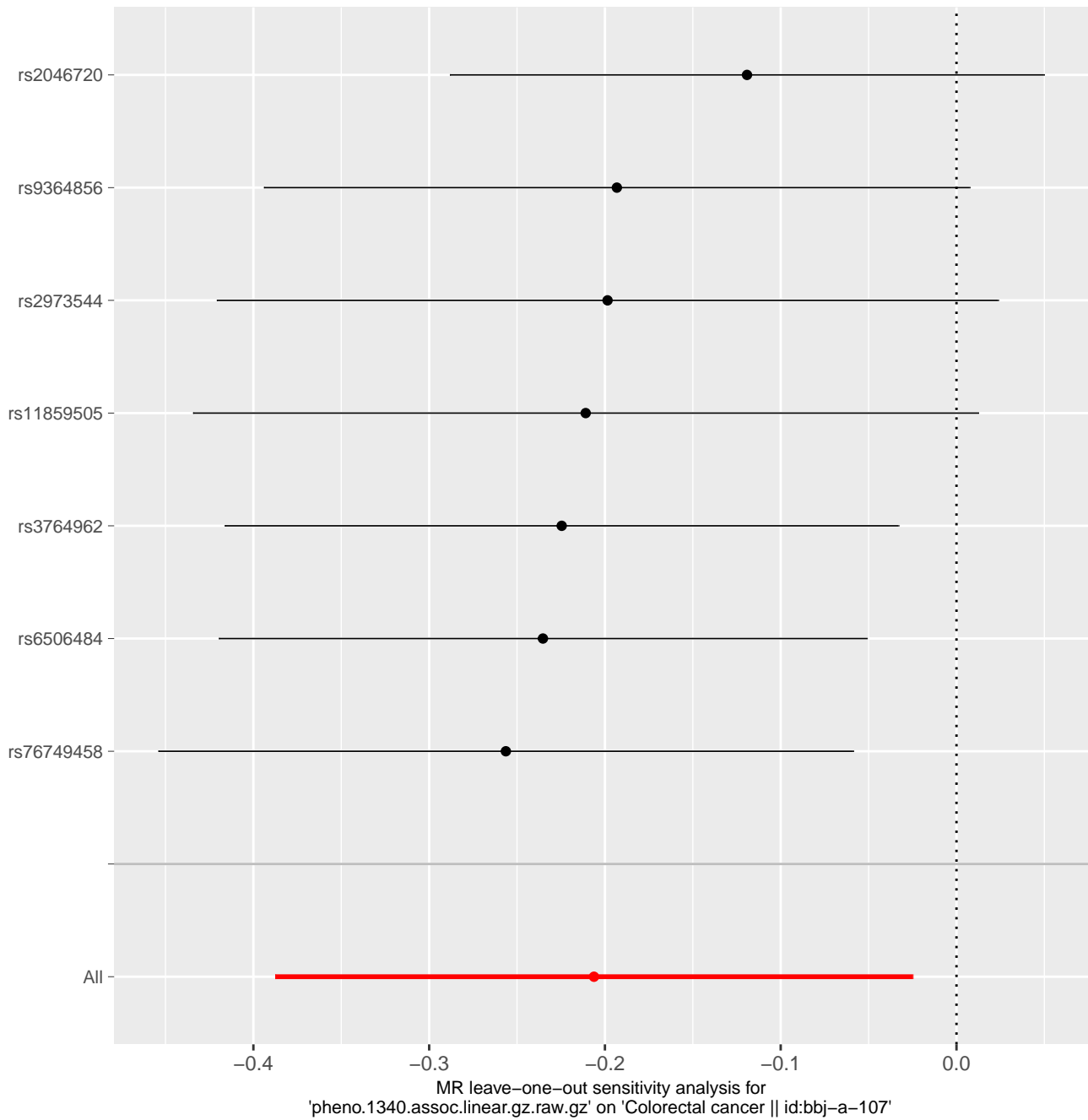

Supplement: Supplementary file 1 [file DataSheet1.ZIP › Supplementary Materials/MR plots for tongue/tongue═╝/Colorectal cancer/pheno.1340_to_colorectal cancer_leave_one_out.pdf]

# MR Test

- Inverse variance weighted
- MR Egger
- Simple mode
- Weighted median
- Weighted mode

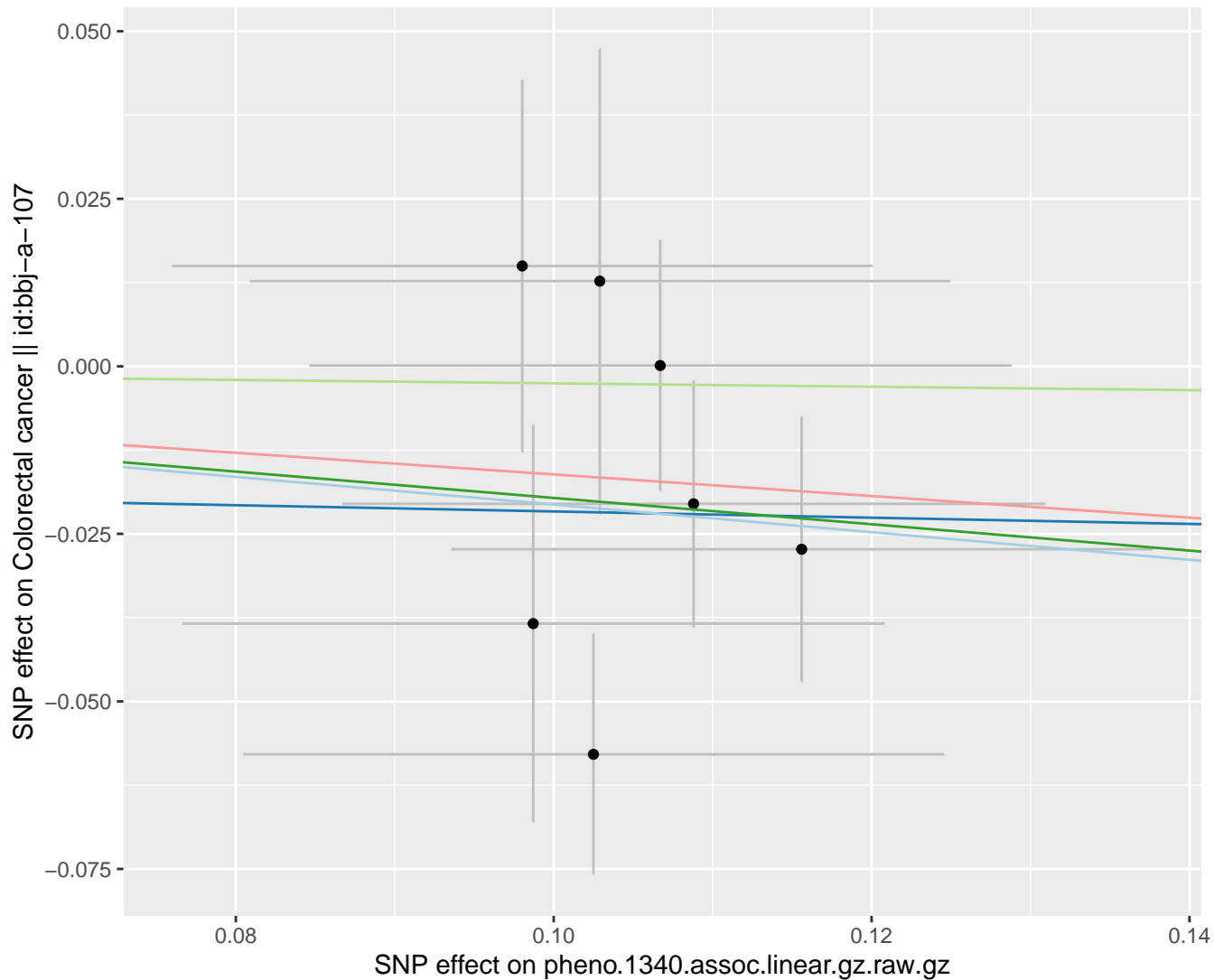

Supplement: Supplementary file 1 [file DataSheet1.ZIP › Supplementary Materials/MR plots for tongue/tongue═╝/Colorectal cancer/pheno.1340_to_colorectal cancer_scatter.pdf]

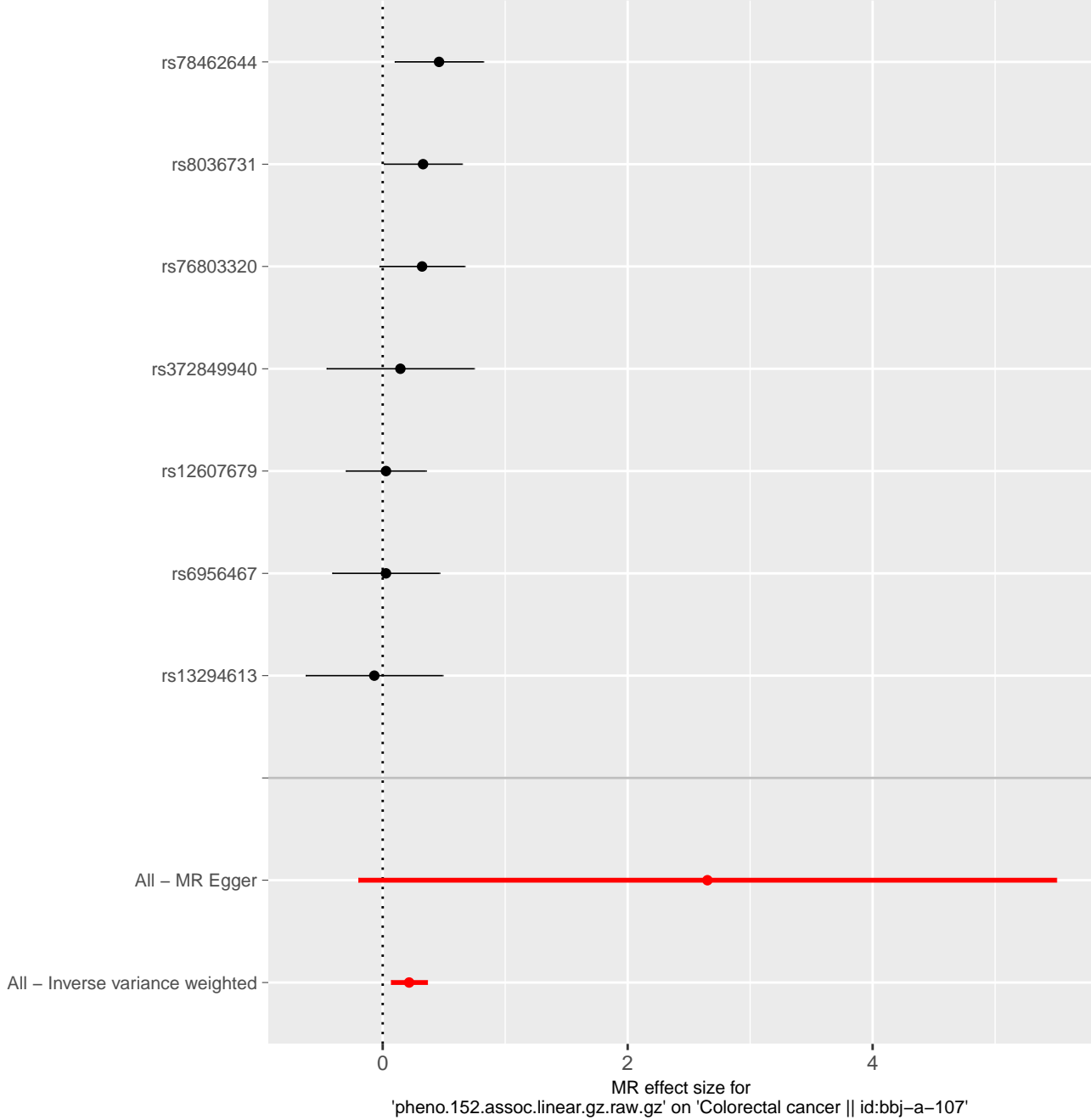

Supplement: Supplementary file 1 [file DataSheet1.ZIP › Supplementary Materials/MR plots for tongue/tongue═╝/Colorectal cancer/pheno.152_to_colorectal cancer_forest.pdf]

# MR Method

- Inverse variance weighted
- MR Egger

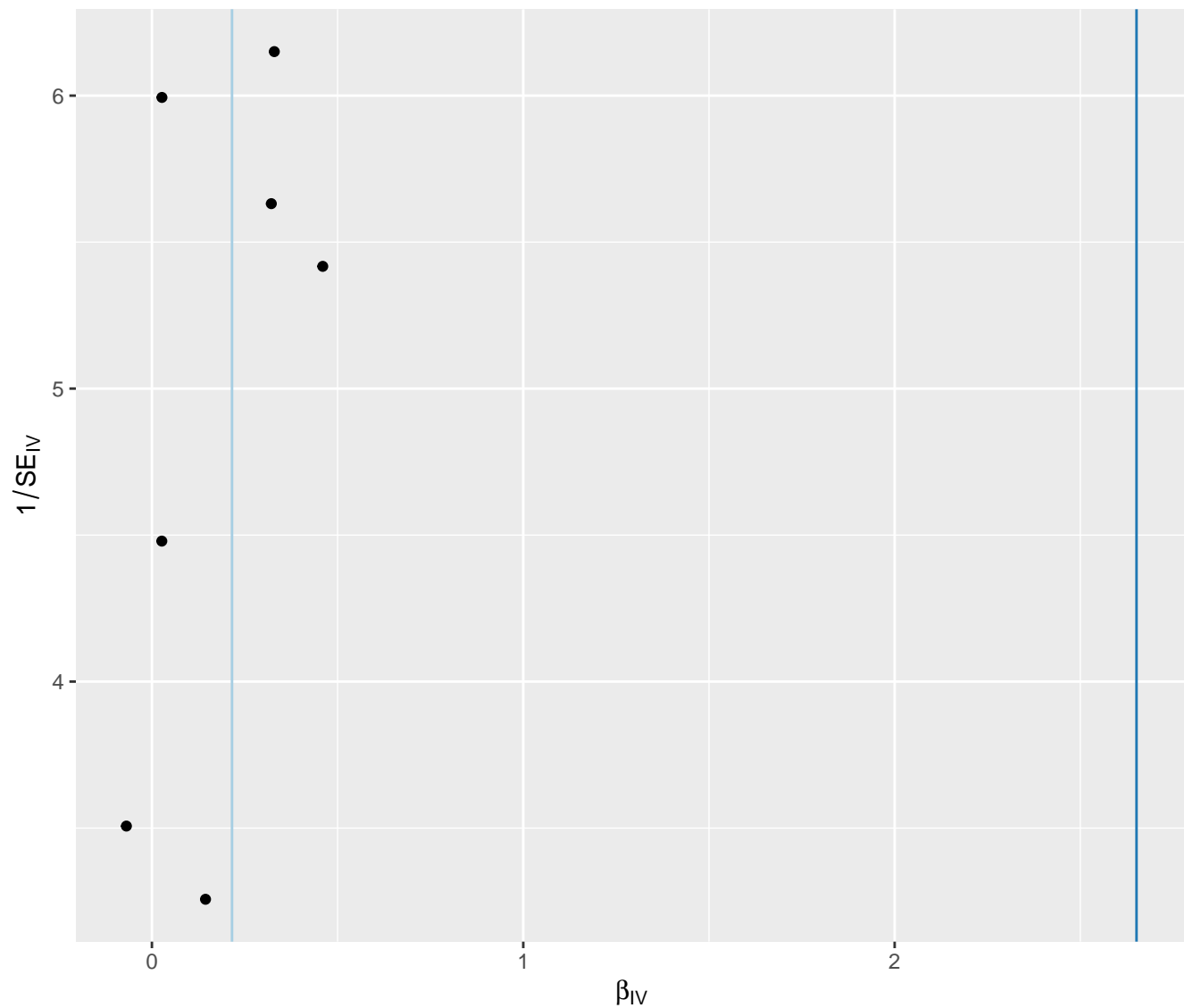

Supplement: Supplementary file 1 [file DataSheet1.ZIP › Supplementary Materials/MR plots for tongue/tongue═╝/Colorectal cancer/pheno.152_to_colorectal cancer_funnel.pdf]

# MR Test

- Inverse variance weighted
- MR Egger
- Simple mode
- Weighted median
- Weighted mode

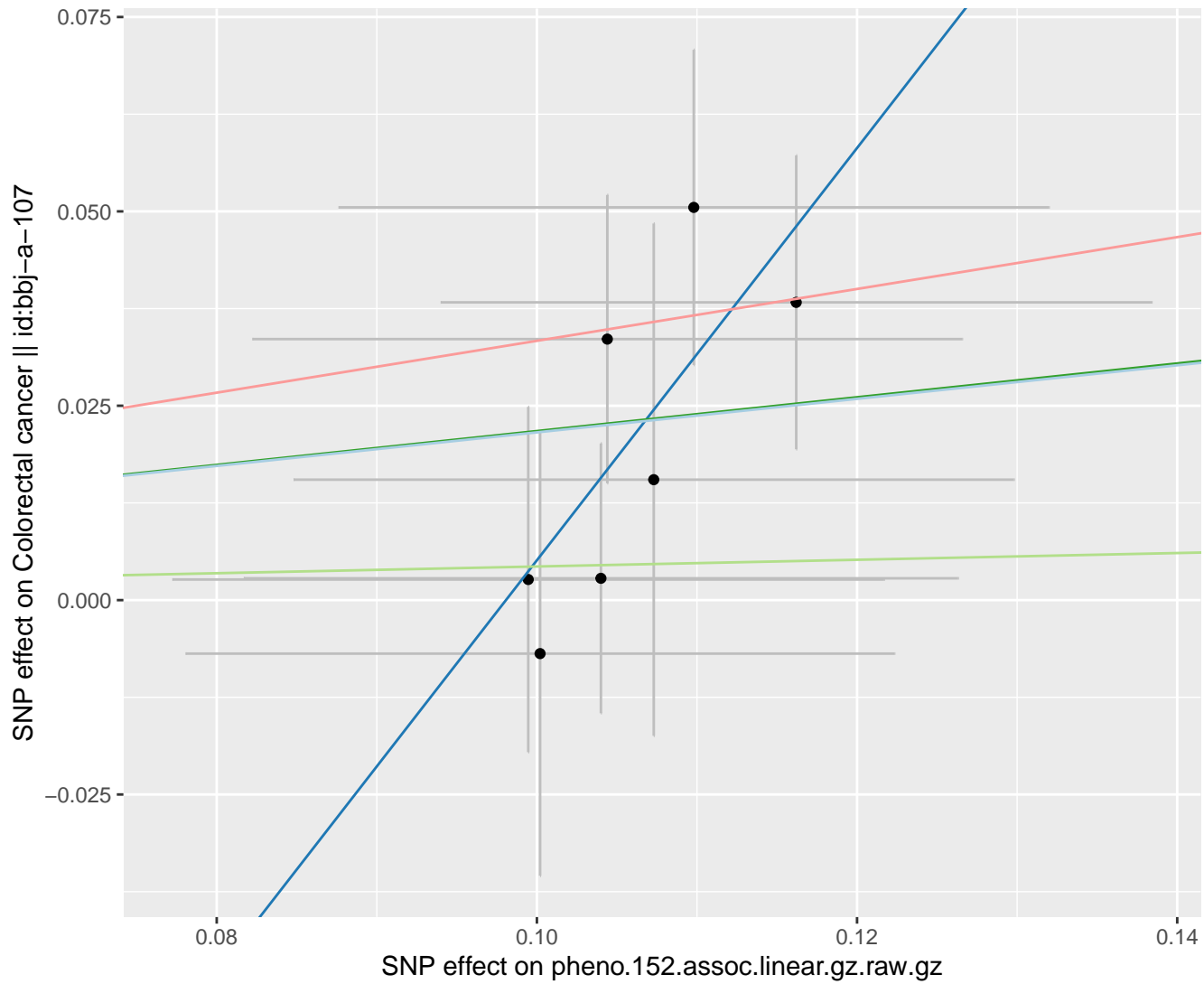

Supplement: Supplementary file 1 [file DataSheet1.ZIP › Supplementary Materials/MR plots for tongue/tongue═╝/Colorectal cancer/pheno.152_to_colorectal cancer_scatter.pdf]

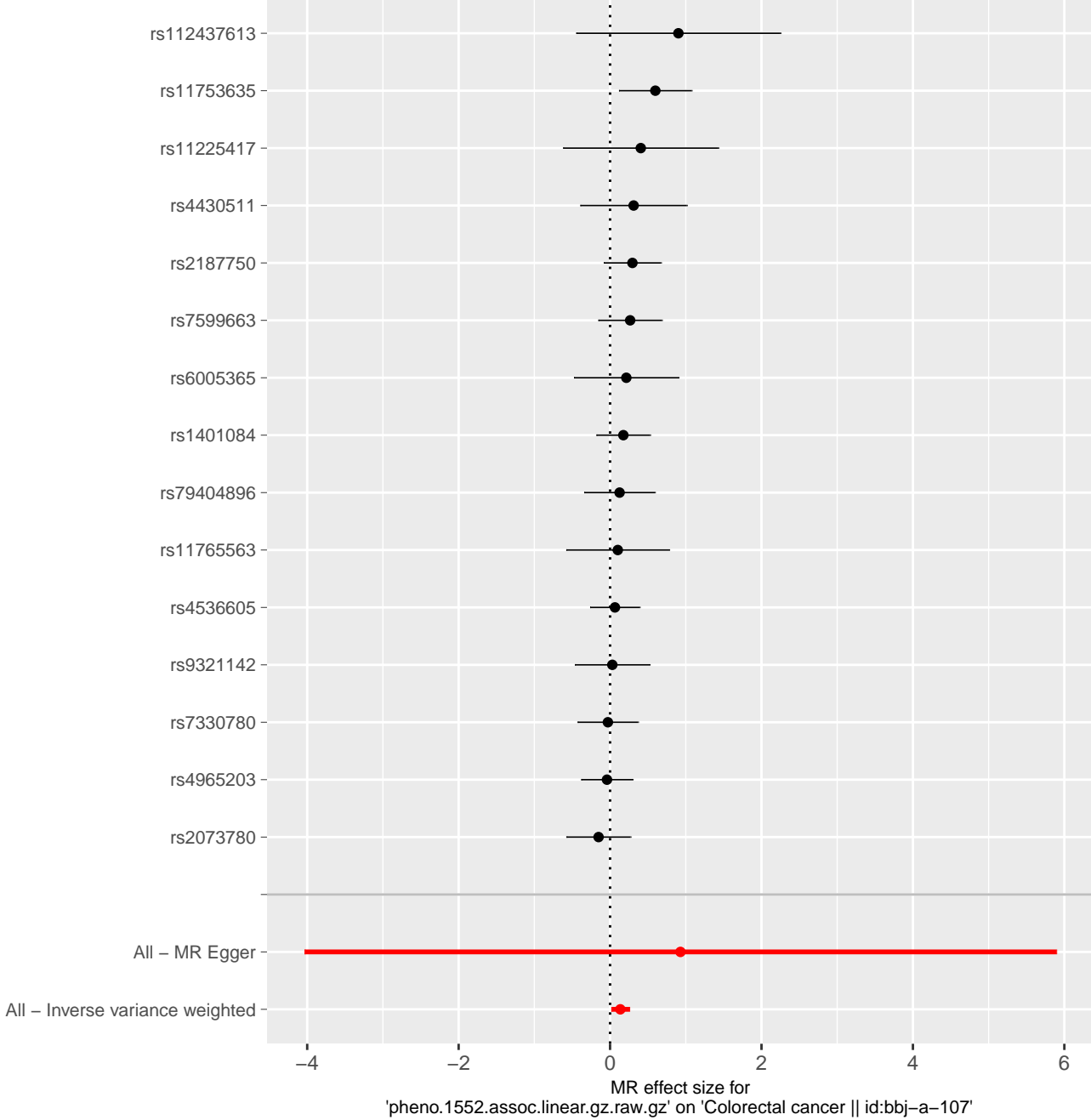

Supplement: Supplementary file 1 [file DataSheet1.ZIP › Supplementary Materials/MR plots for tongue/tongue═╝/Colorectal cancer/pheno.1552_to_colorectal cancer_forest.pdf]

# MR Method

- Inverse variance weighted
- MR Egger

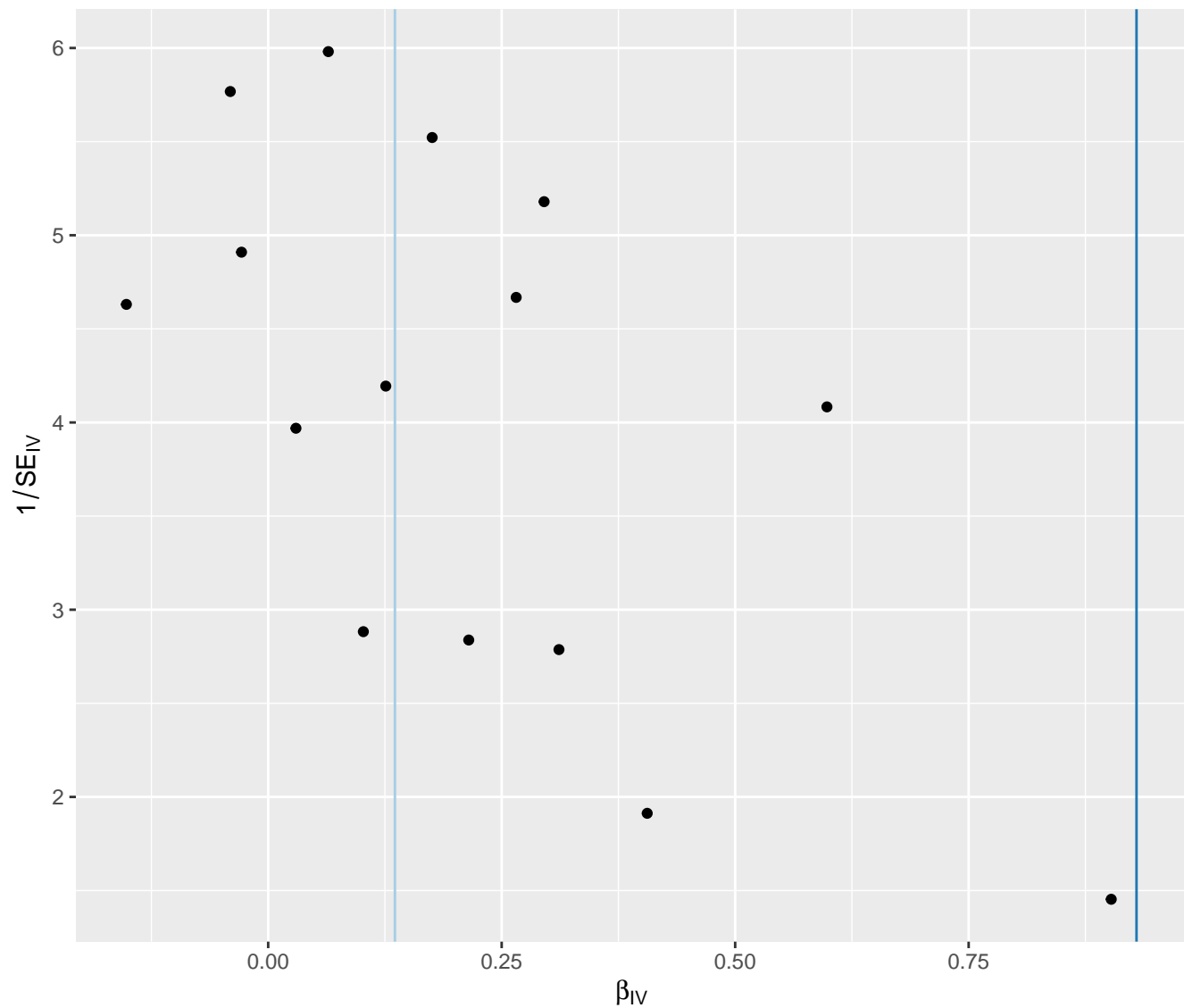

Supplement: Supplementary file 1 [file DataSheet1.ZIP › Supplementary Materials/MR plots for tongue/tongue═╝/Colorectal cancer/pheno.1552_to_colorectal cancer_funnel.pdf]

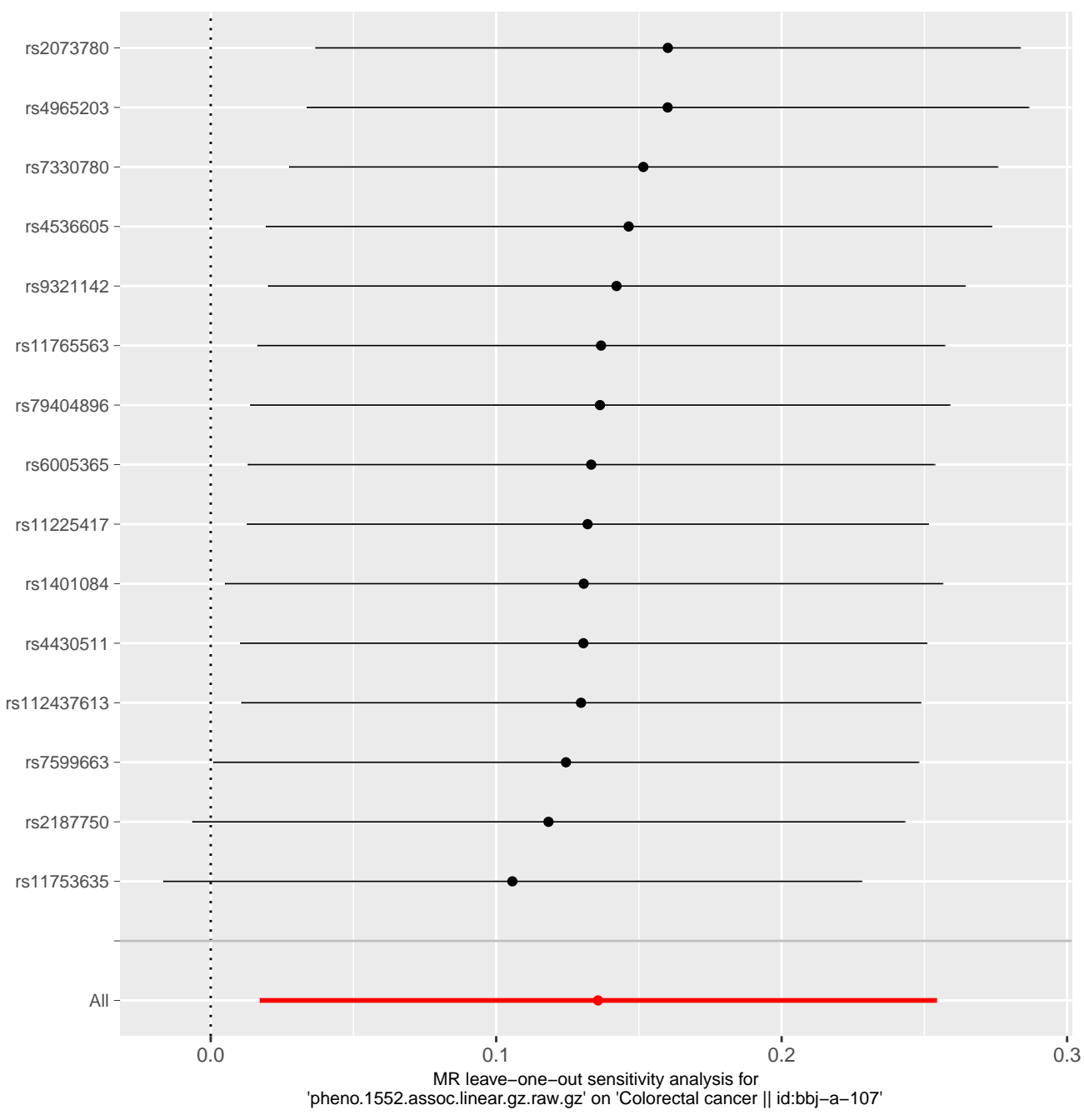

Supplement: Supplementary file 1 [file DataSheet1.ZIP › Supplementary Materials/MR plots for tongue/tongue═╝/Colorectal cancer/pheno.1552_to_colorectal cancer_leave_one_out.pdf]

# MR Test

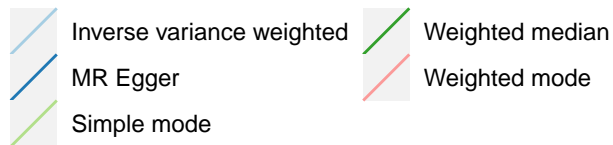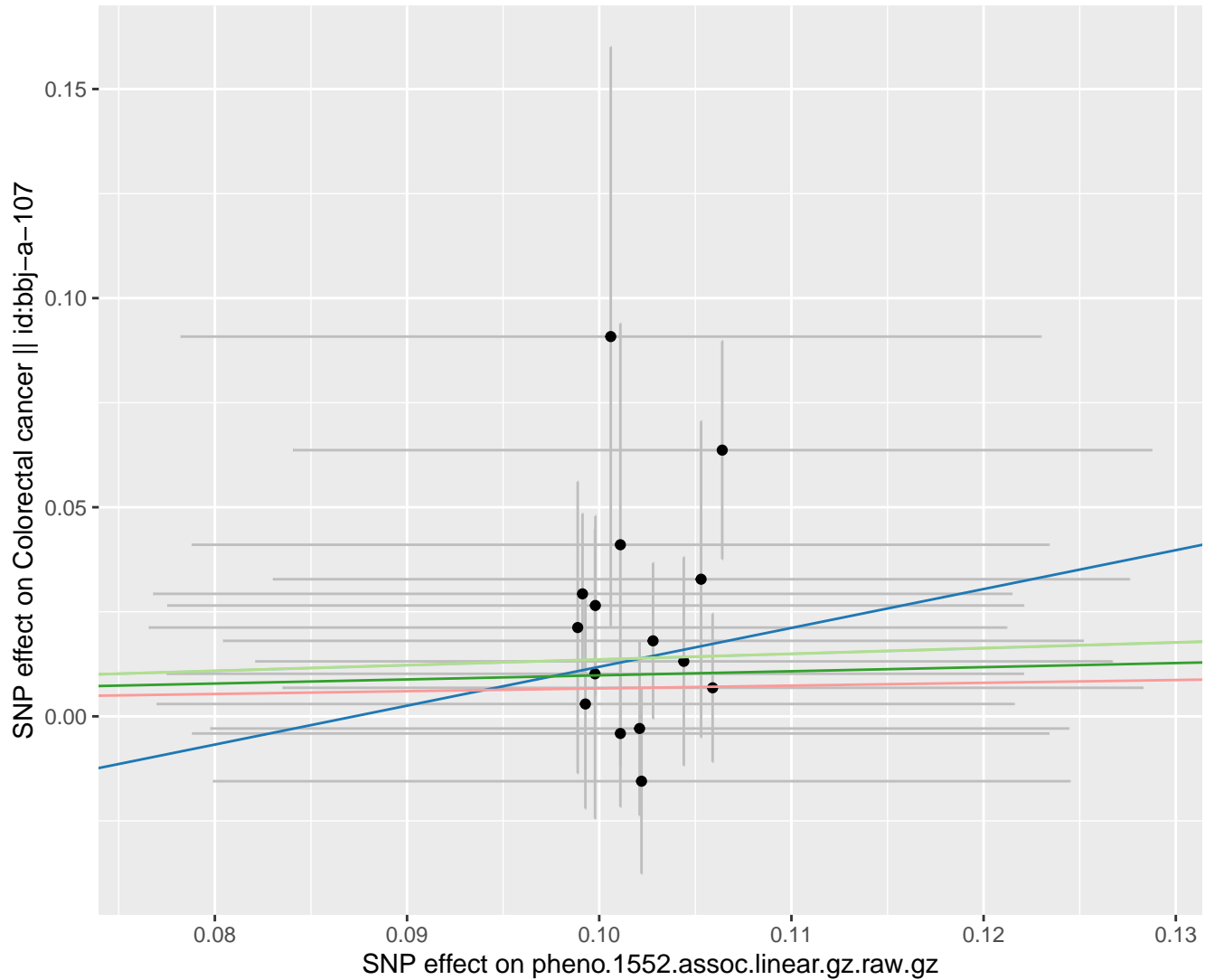

Supplement: Supplementary file 1 [file DataSheet1.ZIP › Supplementary Materials/MR plots for tongue/tongue═╝/Colorectal cancer/pheno.1552_to_colorectal cancer_scatter.pdf]

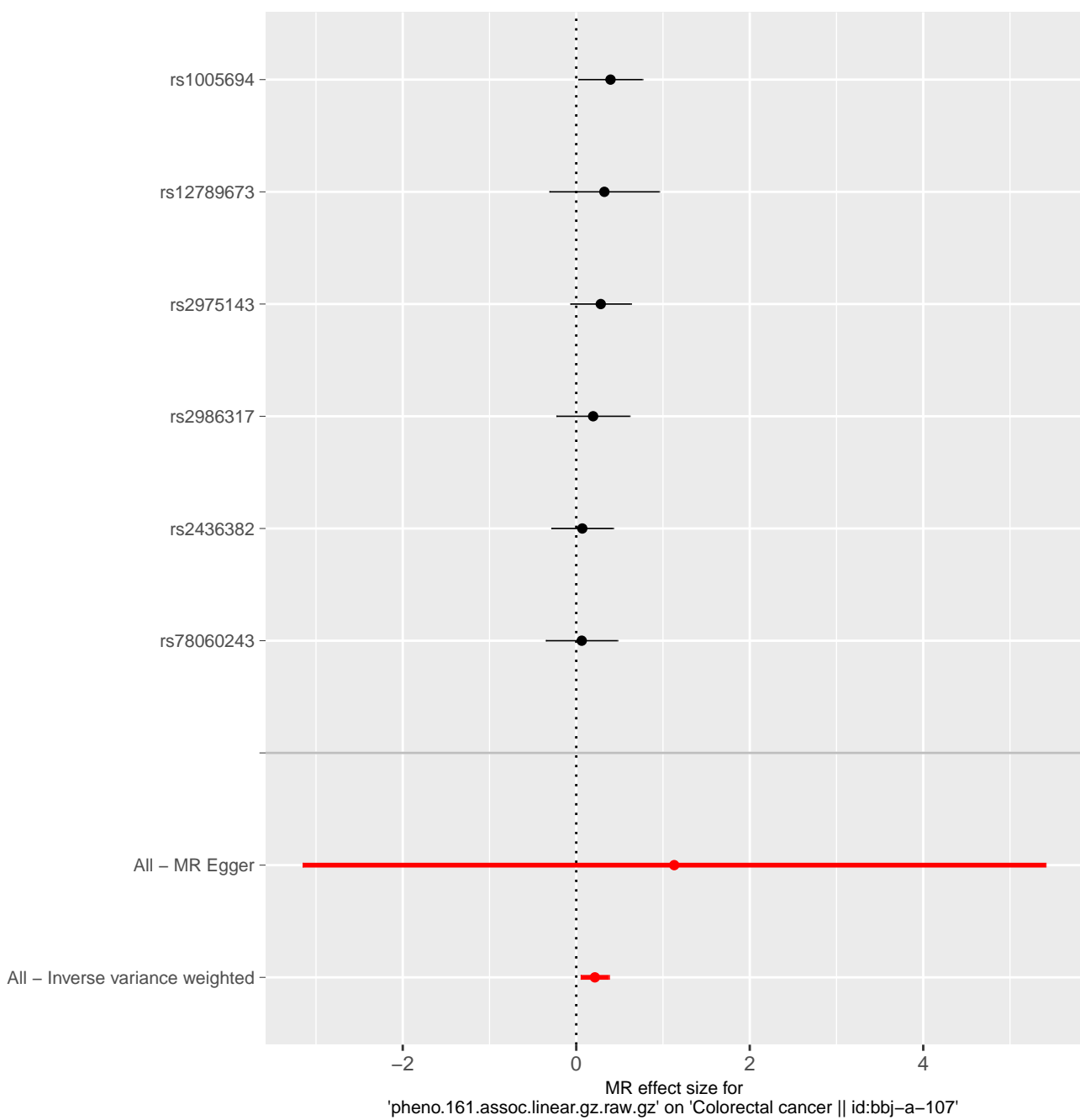

Supplement: Supplementary file 1 [file DataSheet1.ZIP › Supplementary Materials/MR plots for tongue/tongue═╝/Colorectal cancer/pheno.161_to_colorectal cancer_forest.pdf]

# MR Method

- Inverse variance weighted
- MR Egger

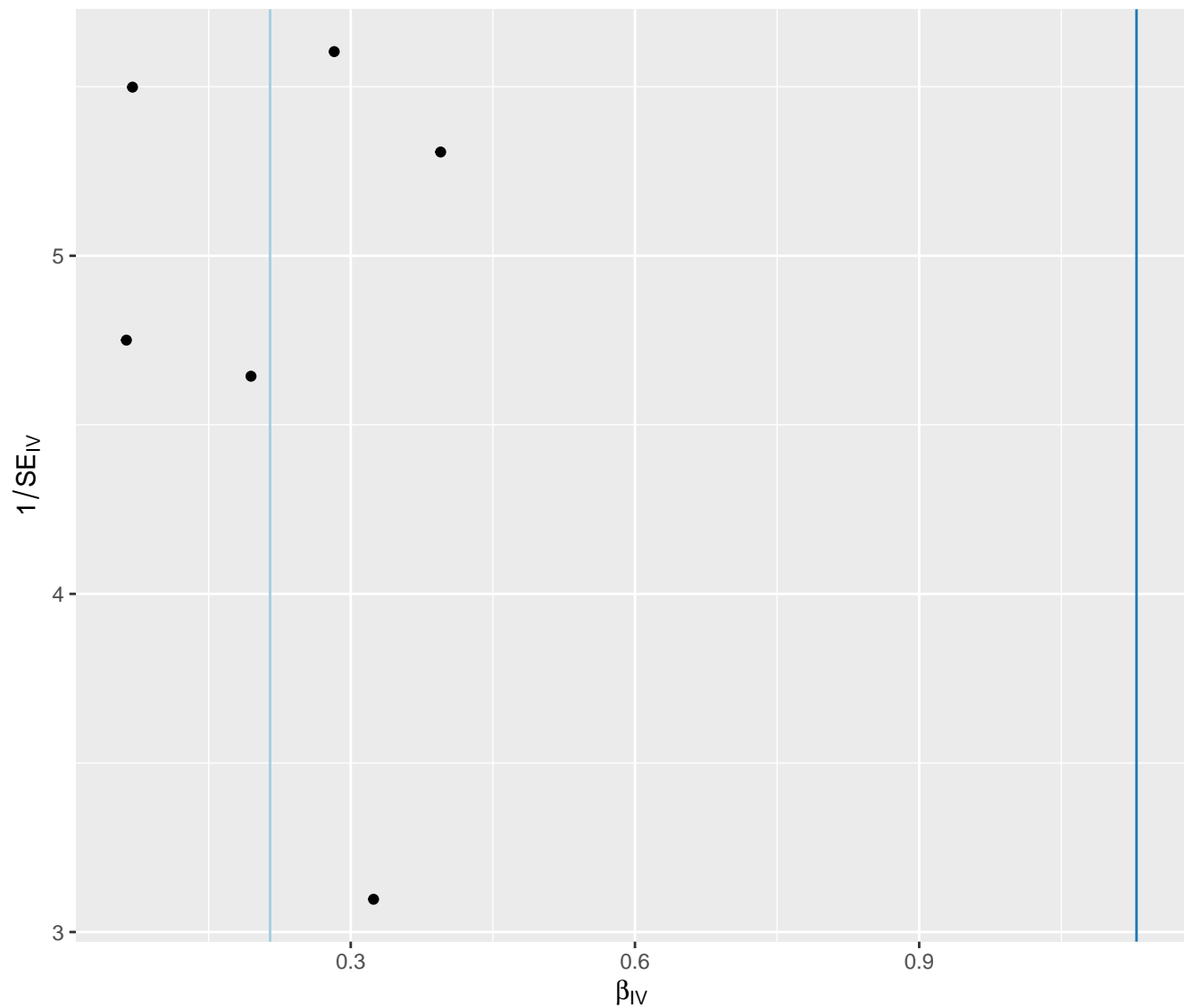

Supplement: Supplementary file 1 [file DataSheet1.ZIP › Supplementary Materials/MR plots for tongue/tongue═╝/Colorectal cancer/pheno.161_to_colorectal cancer_funnel.pdf]

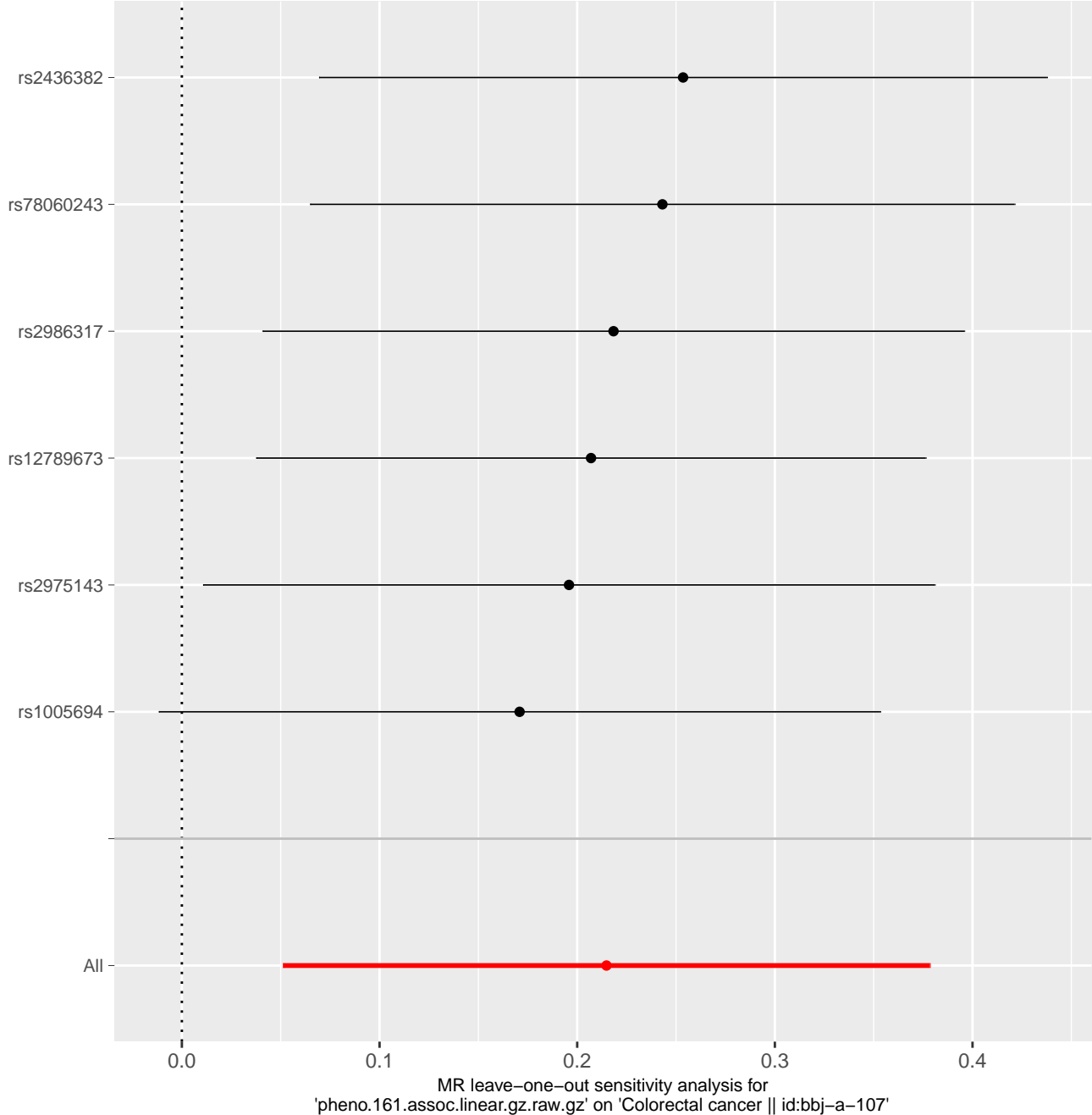

Supplement: Supplementary file 1 [file DataSheet1.ZIP › Supplementary Materials/MR plots for tongue/tongue═╝/Colorectal cancer/pheno.161_to_colorectal cancer_leave_one_out.pdf]

# MR Test

- Inverse variance weighted
- MR Egger
- Simple mode
- Weighted median
- Weighted mode

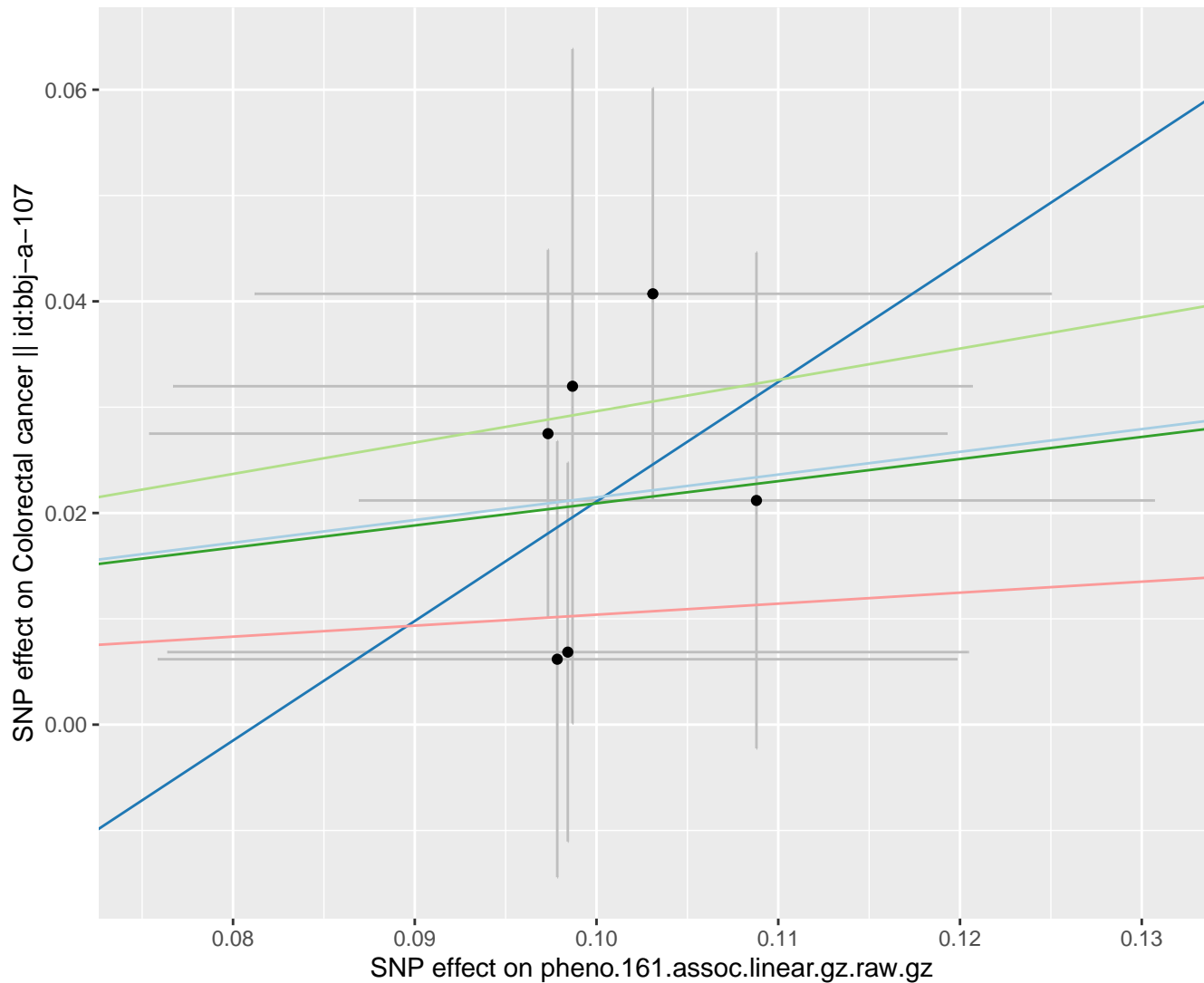

Supplement: Supplementary file 1 [file DataSheet1.ZIP › Supplementary Materials/MR plots for tongue/tongue═╝/Colorectal cancer/pheno.161_to_colorectal cancer_scatter.pdf]

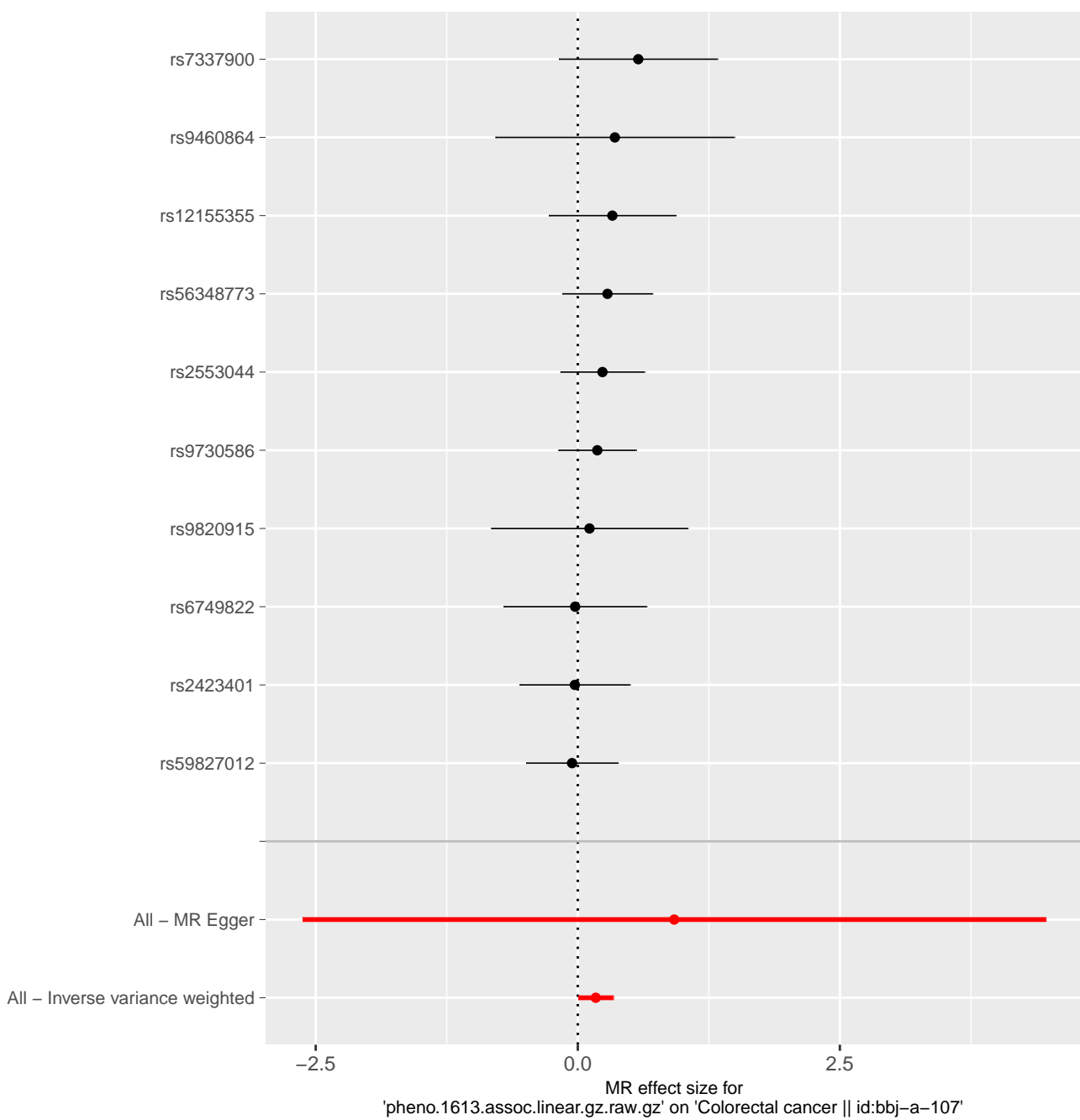

Supplement: Supplementary file 1 [file DataSheet1.ZIP › Supplementary Materials/MR plots for tongue/tongue═╝/Colorectal cancer/pheno.1613_to_colorectal cancer_forest.pdf]

# MR Method

- Inverse variance weighted
- MR Egger

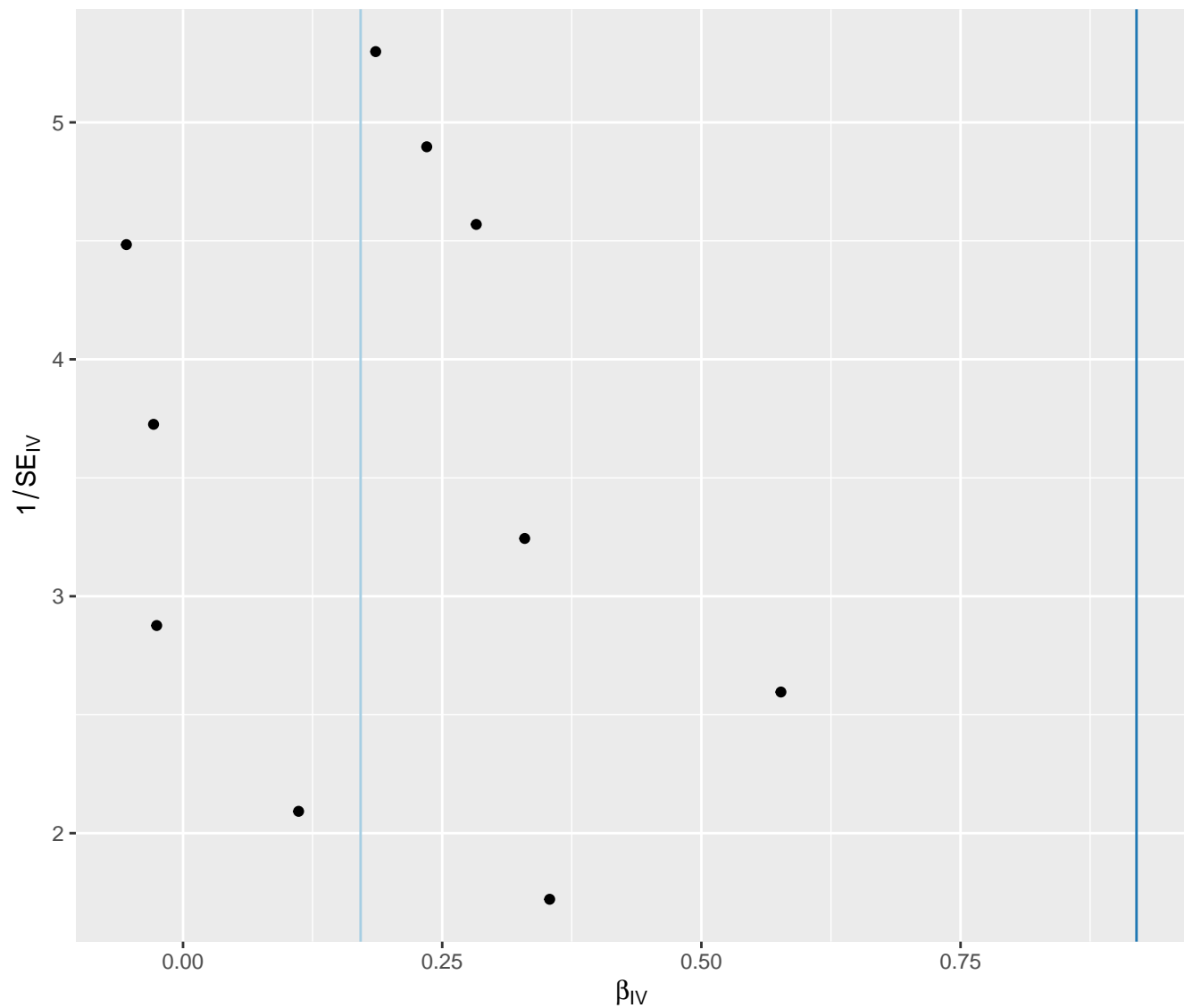

Supplement: Supplementary file 1 [file DataSheet1.ZIP › Supplementary Materials/MR plots for tongue/tongue═╝/Colorectal cancer/pheno.1613_to_colorectal cancer_funnel.pdf]

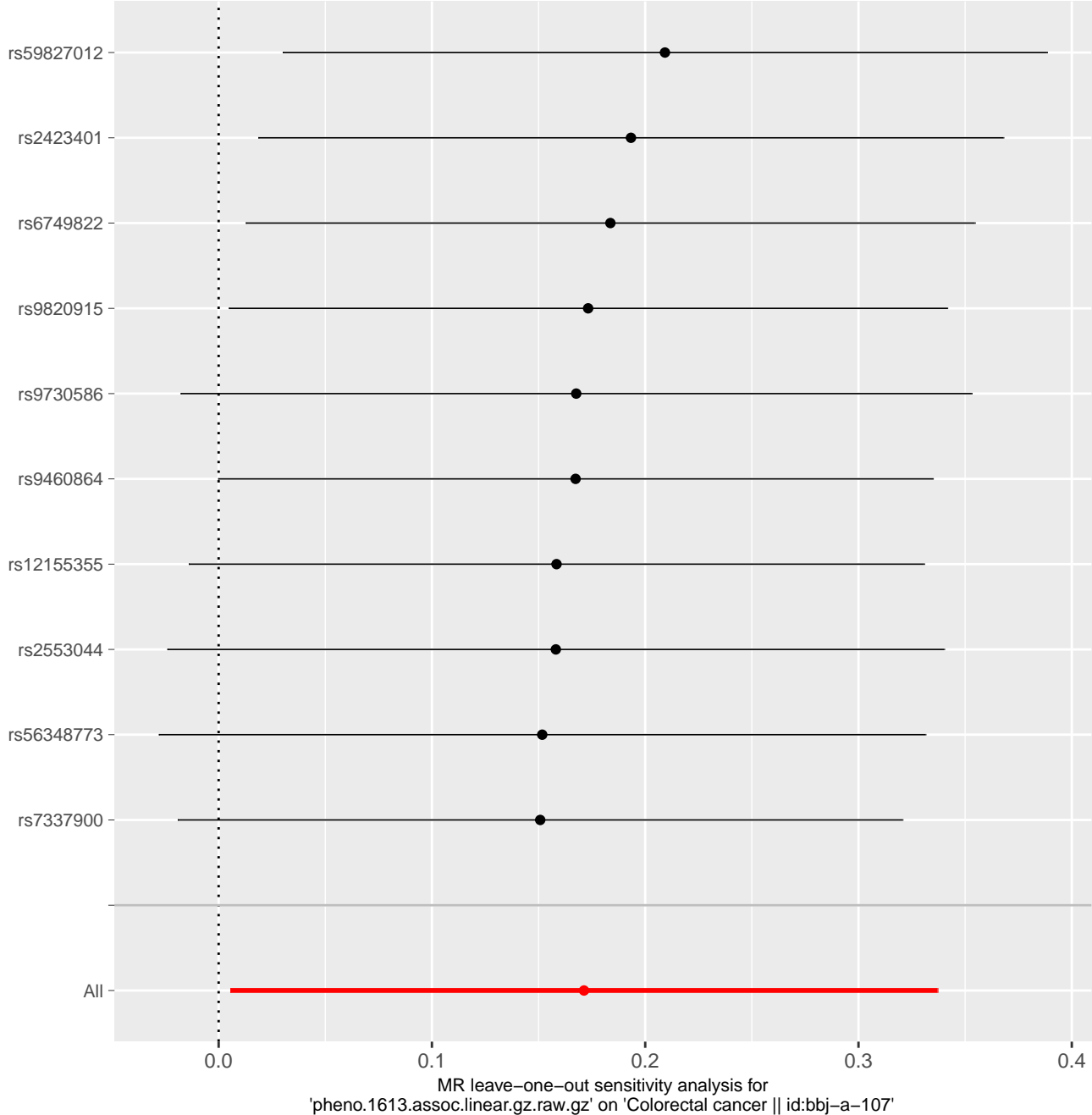

Supplement: Supplementary file 1 [file DataSheet1.ZIP › Supplementary Materials/MR plots for tongue/tongue═╝/Colorectal cancer/pheno.1613_to_colorectal cancer_leave_one_out.pdf]

# MR Test

- Inverse variance weighted
- MR Egger
- Simple mode
- Weighted median
- Weighted mode

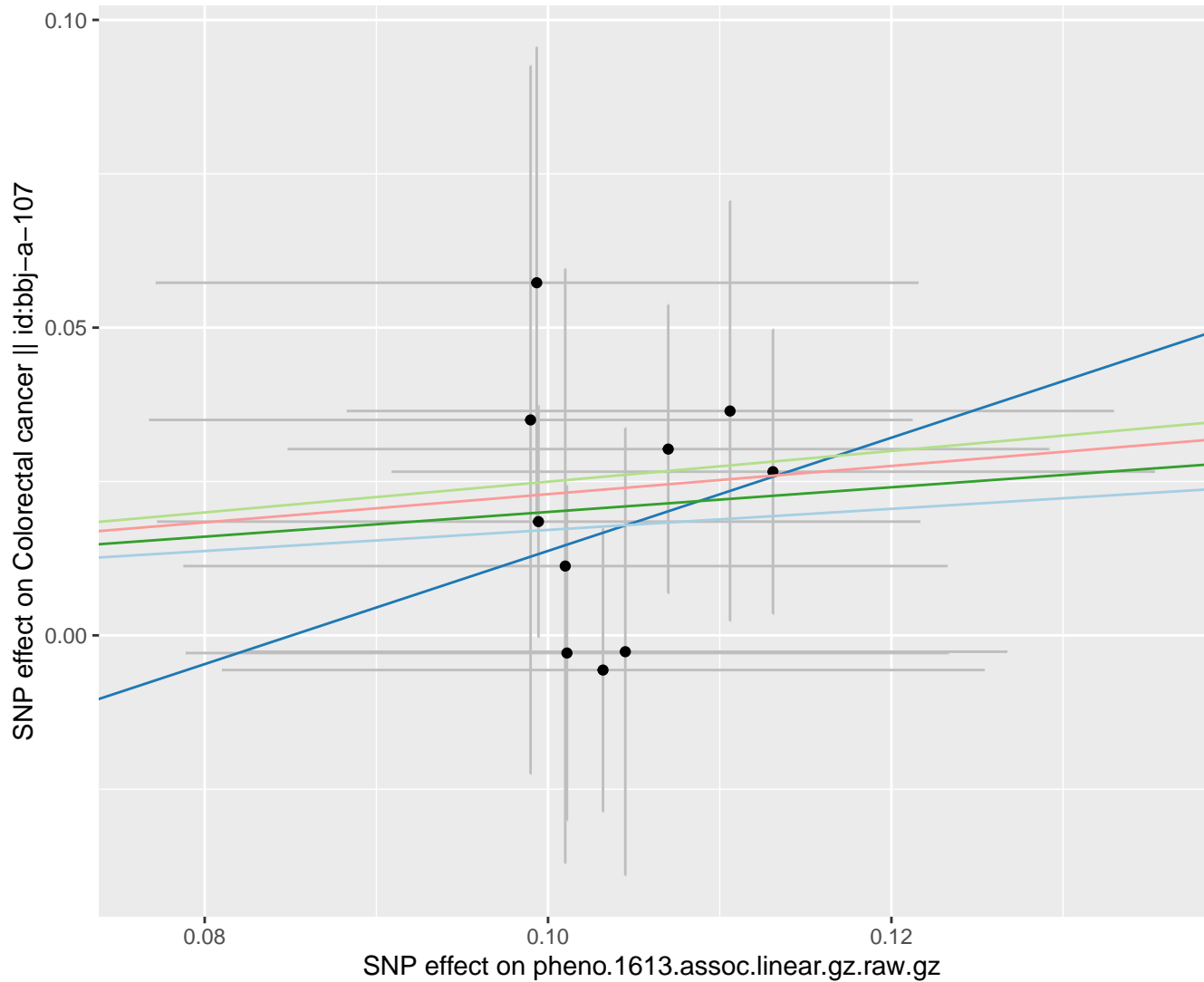

Supplement: Supplementary file 1 [file DataSheet1.ZIP › Supplementary Materials/MR plots for tongue/tongue═╝/Colorectal cancer/pheno.1613_to_colorectal cancer_scatter.pdf]

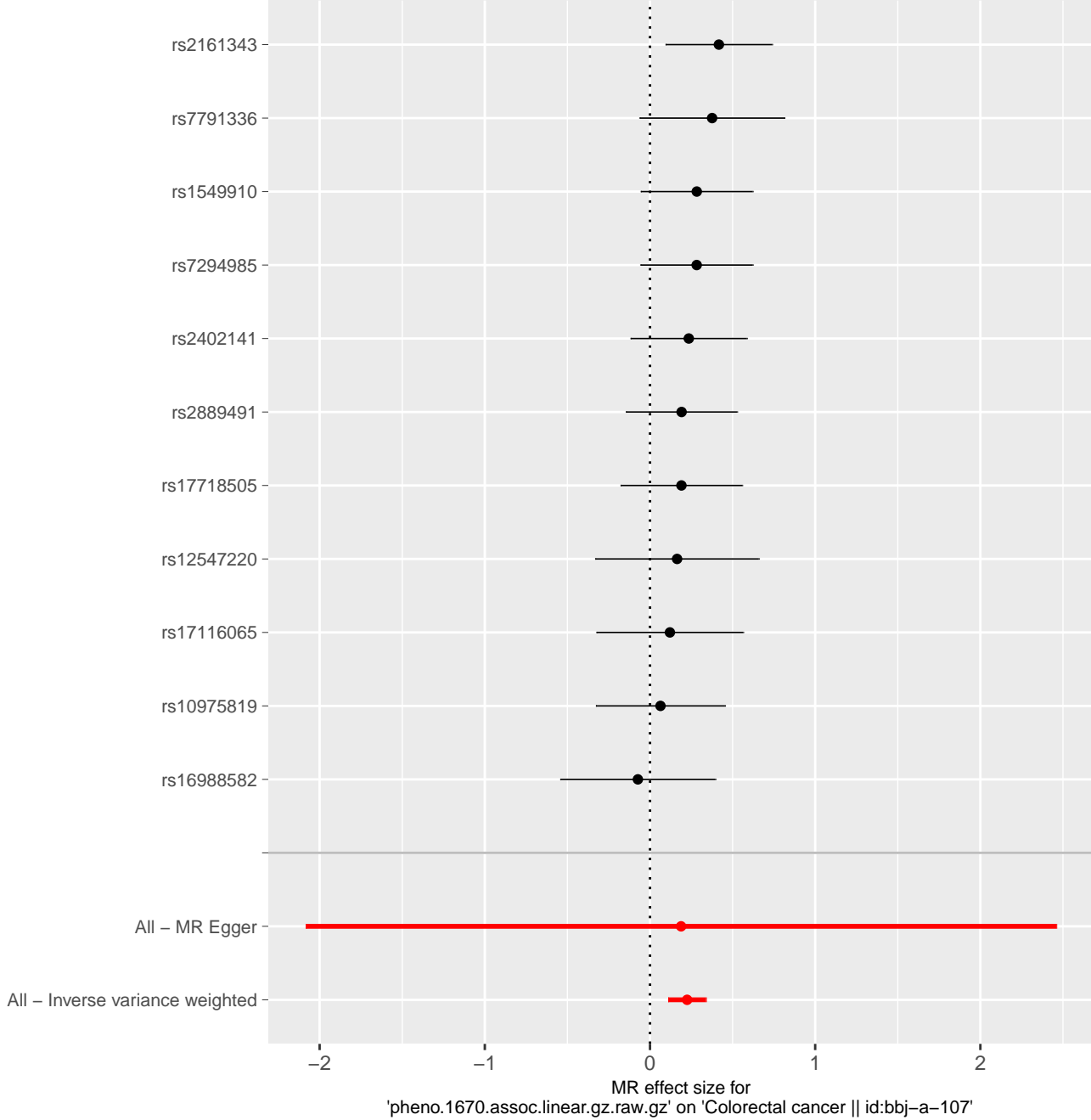

Supplement: Supplementary file 1 [file DataSheet1.ZIP › Supplementary Materials/MR plots for tongue/tongue═╝/Colorectal cancer/pheno.1670_to_colorectal cancer_forest.pdf]

# MR Method

Inverse variance weighted  
MR Egger

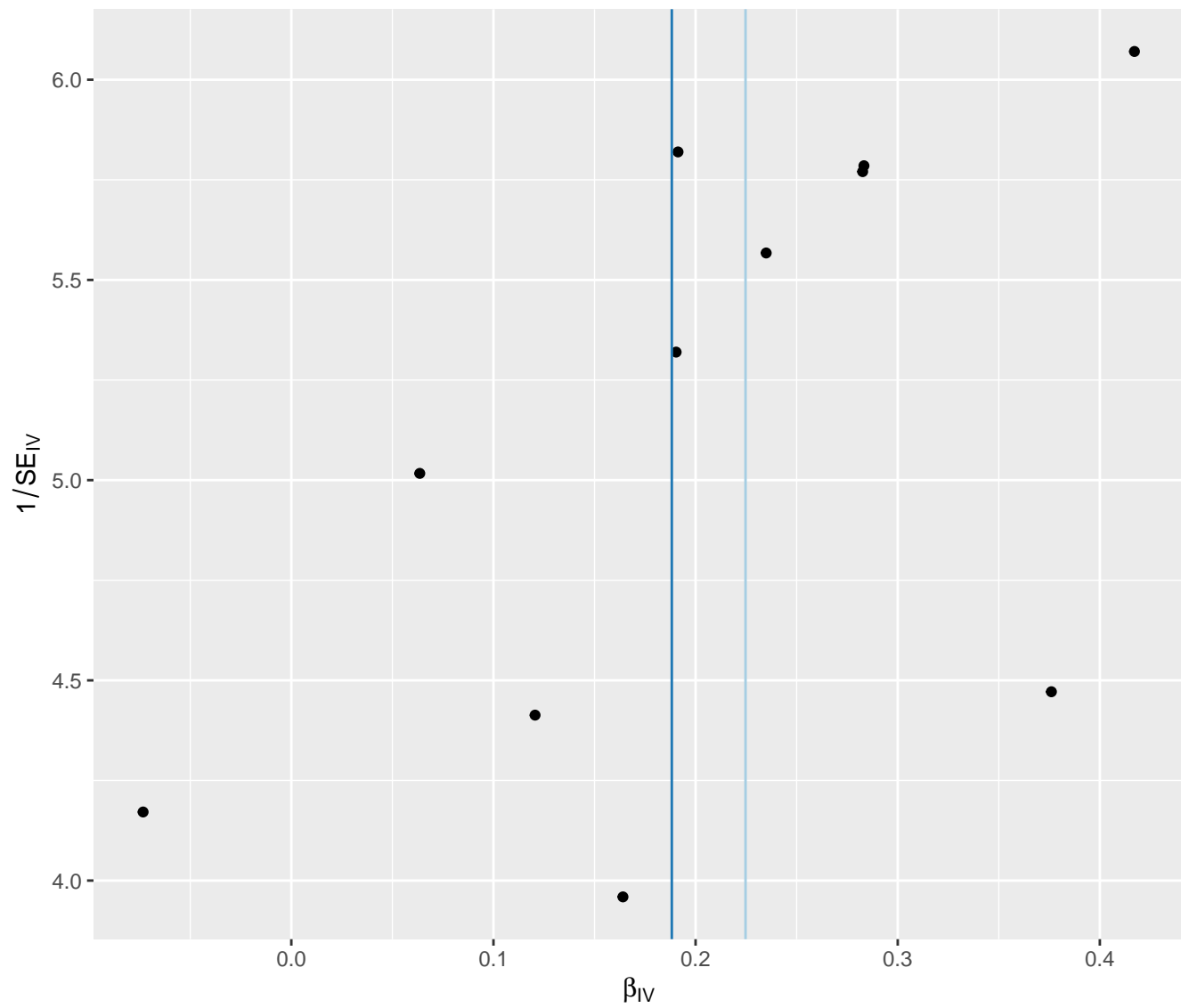

Supplement: Supplementary file 1 [file DataSheet1.ZIP › Supplementary Materials/MR plots for tongue/tongue═╝/Colorectal cancer/pheno.1670_to_colorectal cancer_funnel.pdf]

# MR Test

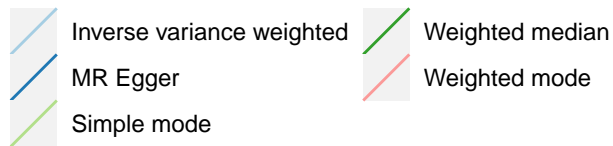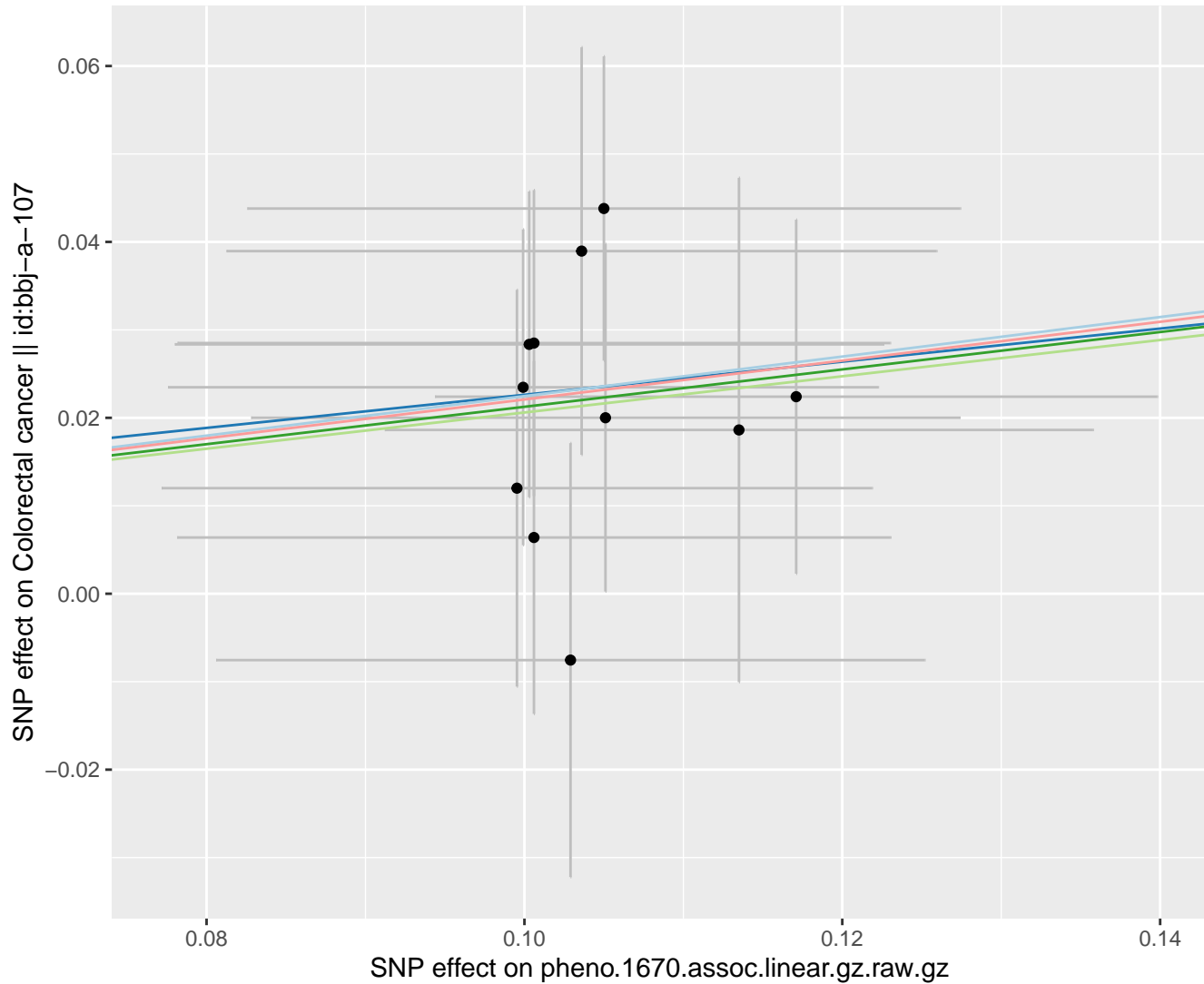

Supplement: Supplementary file 1 [file DataSheet1.ZIP › Supplementary Materials/MR plots for tongue/tongue═╝/Colorectal cancer/pheno.1670_to_colorectal cancer_scatter.pdf]

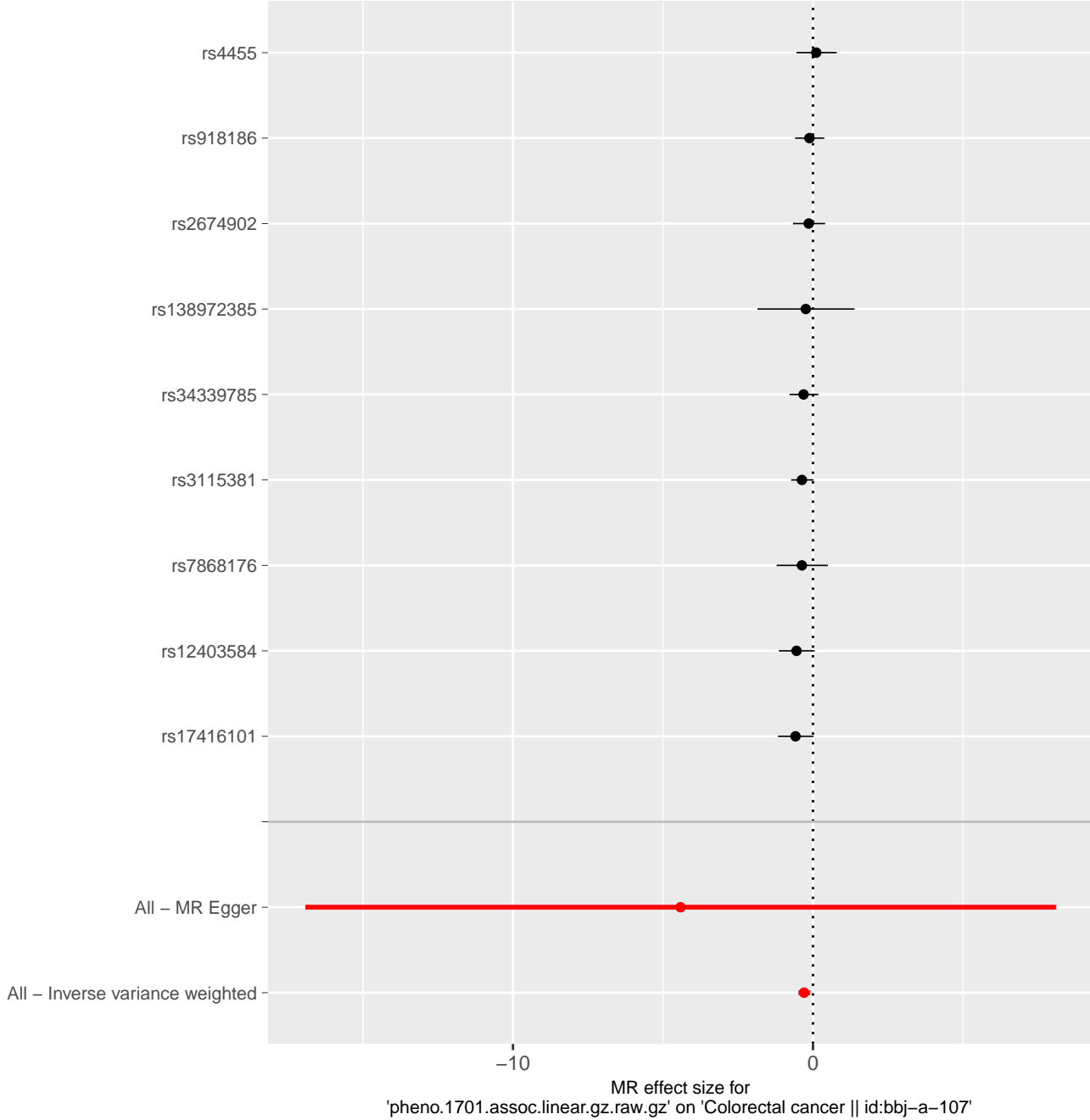

Supplement: Supplementary file 1 [file DataSheet1.ZIP › Supplementary Materials/MR plots for tongue/tongue═╝/Colorectal cancer/pheno.1701_to_colorectal cancer_forest.pdf]

# MR Method

- Inverse variance weighted
- MR Egger

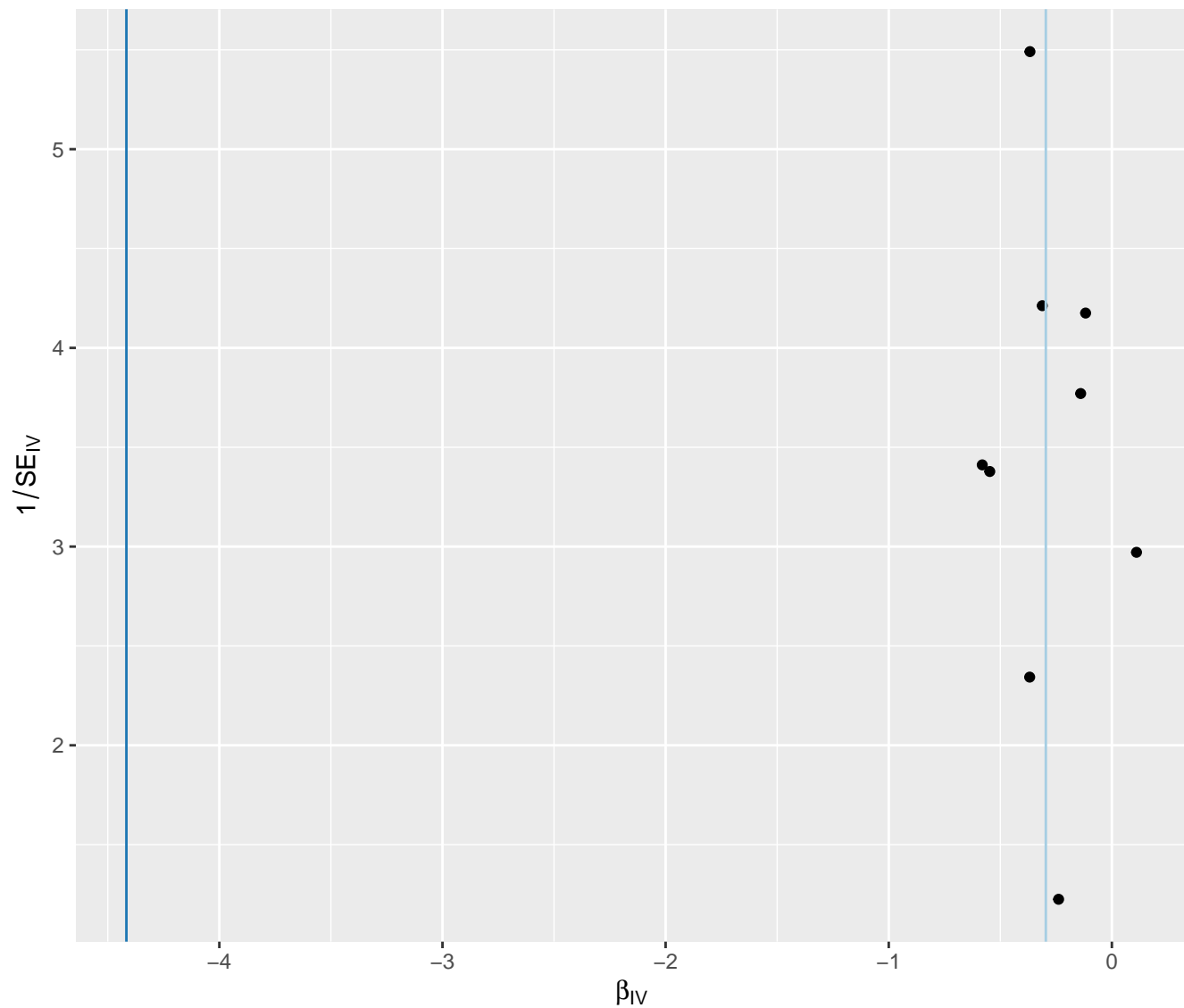

Supplement: Supplementary file 1 [file DataSheet1.ZIP › Supplementary Materials/MR plots for tongue/tongue═╝/Colorectal cancer/pheno.1701_to_colorectal cancer_funnel.pdf]

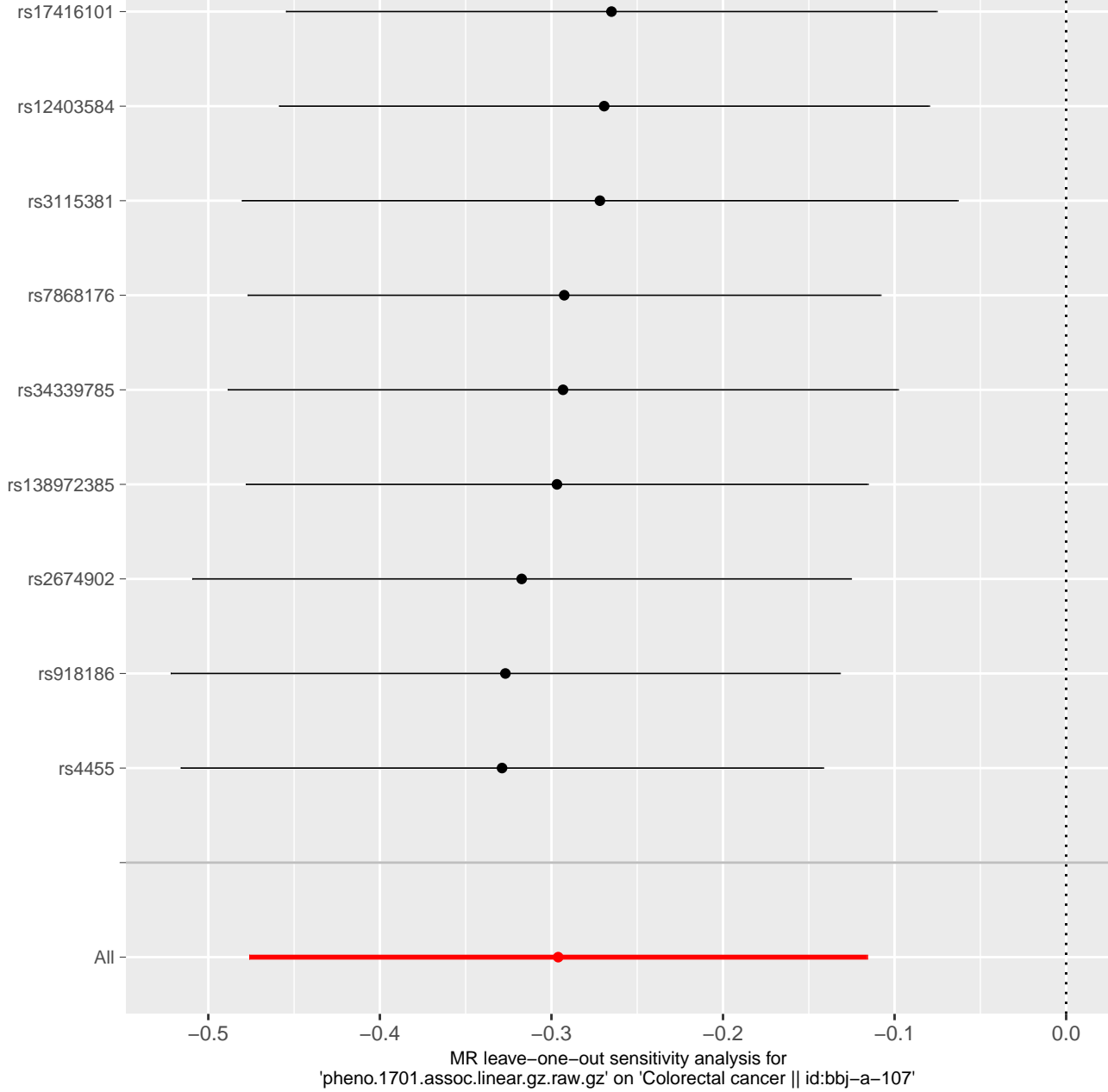

Supplement: Supplementary file 1 [file DataSheet1.ZIP › Supplementary Materials/MR plots for tongue/tongue═╝/Colorectal cancer/pheno.1701_to_colorectal cancer_leave_one_out.pdf]

# MR Test

- Inverse variance weighted
- MR Egger
- Simple mode
- Weighted median
- Weighted mode

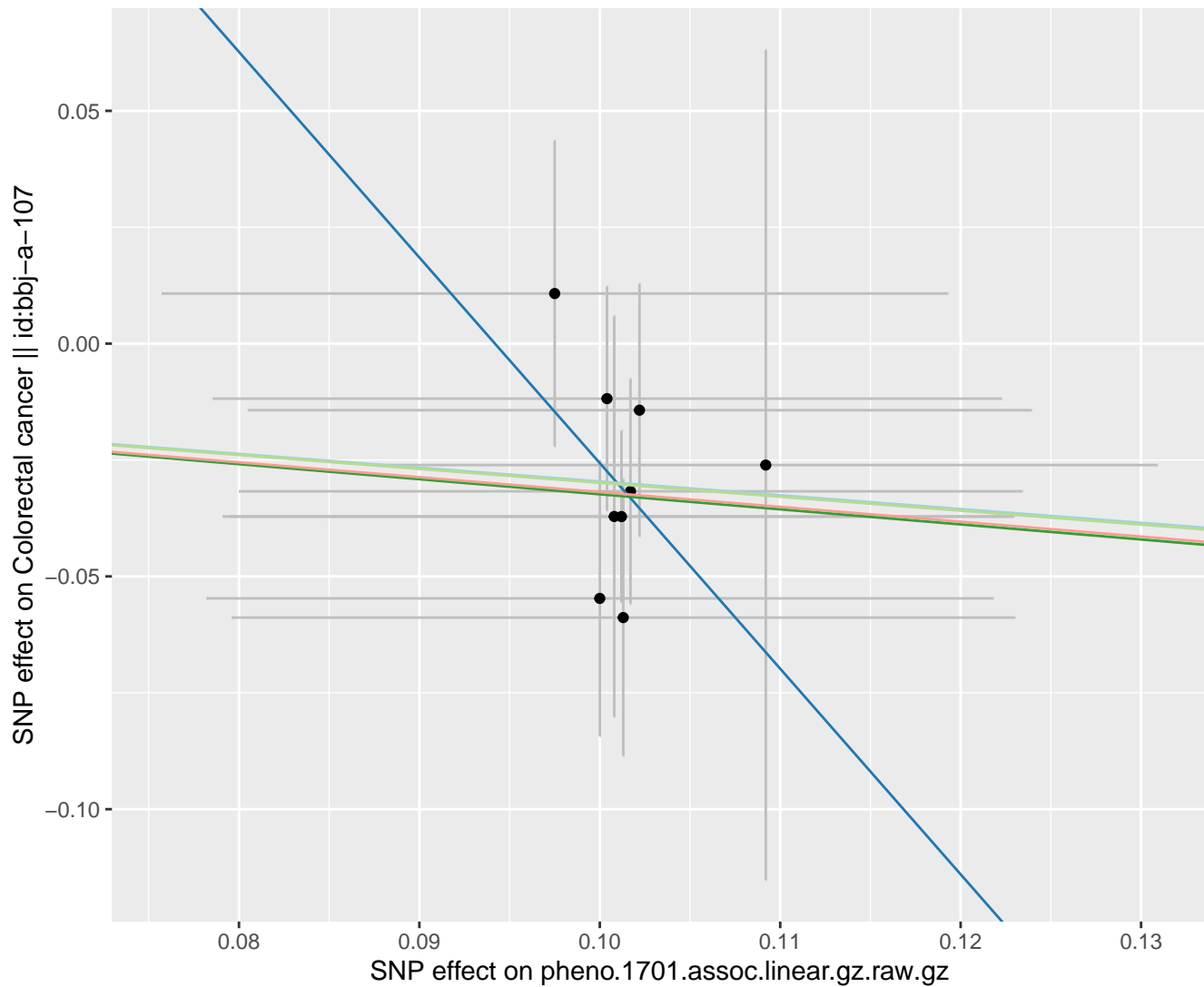

Supplement: Supplementary file 1 [file DataSheet1.ZIP › Supplementary Materials/MR plots for tongue/tongue═╝/Colorectal cancer/pheno.1701_to_colorectal cancer_scatter.pdf]

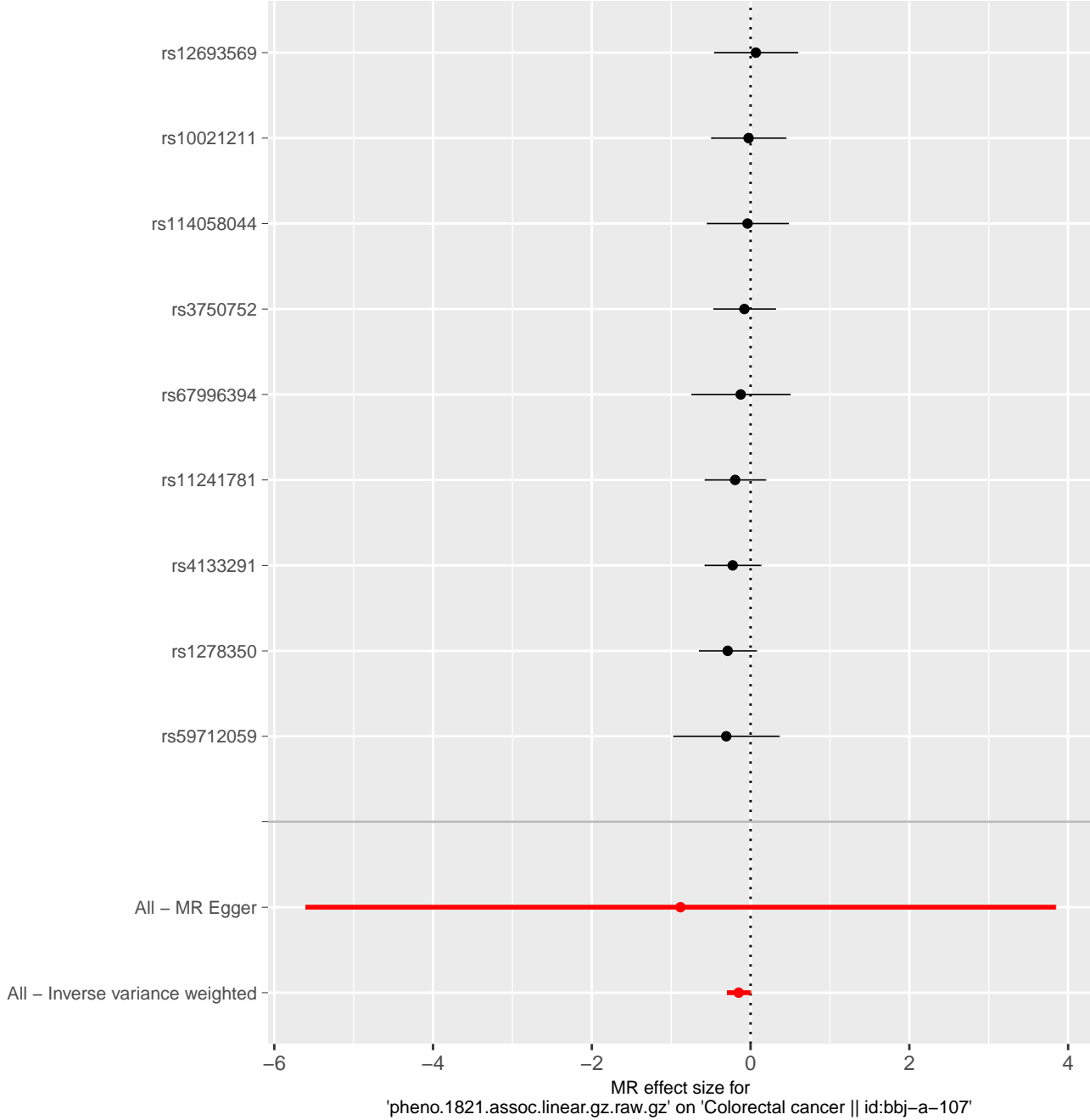

Supplement: Supplementary file 1 [file DataSheet1.ZIP › Supplementary Materials/MR plots for tongue/tongue═╝/Colorectal cancer/pheno.1821_to_colorectal cancer_forest.pdf]

# MR Method

- Inverse variance weighted
- MR Egger

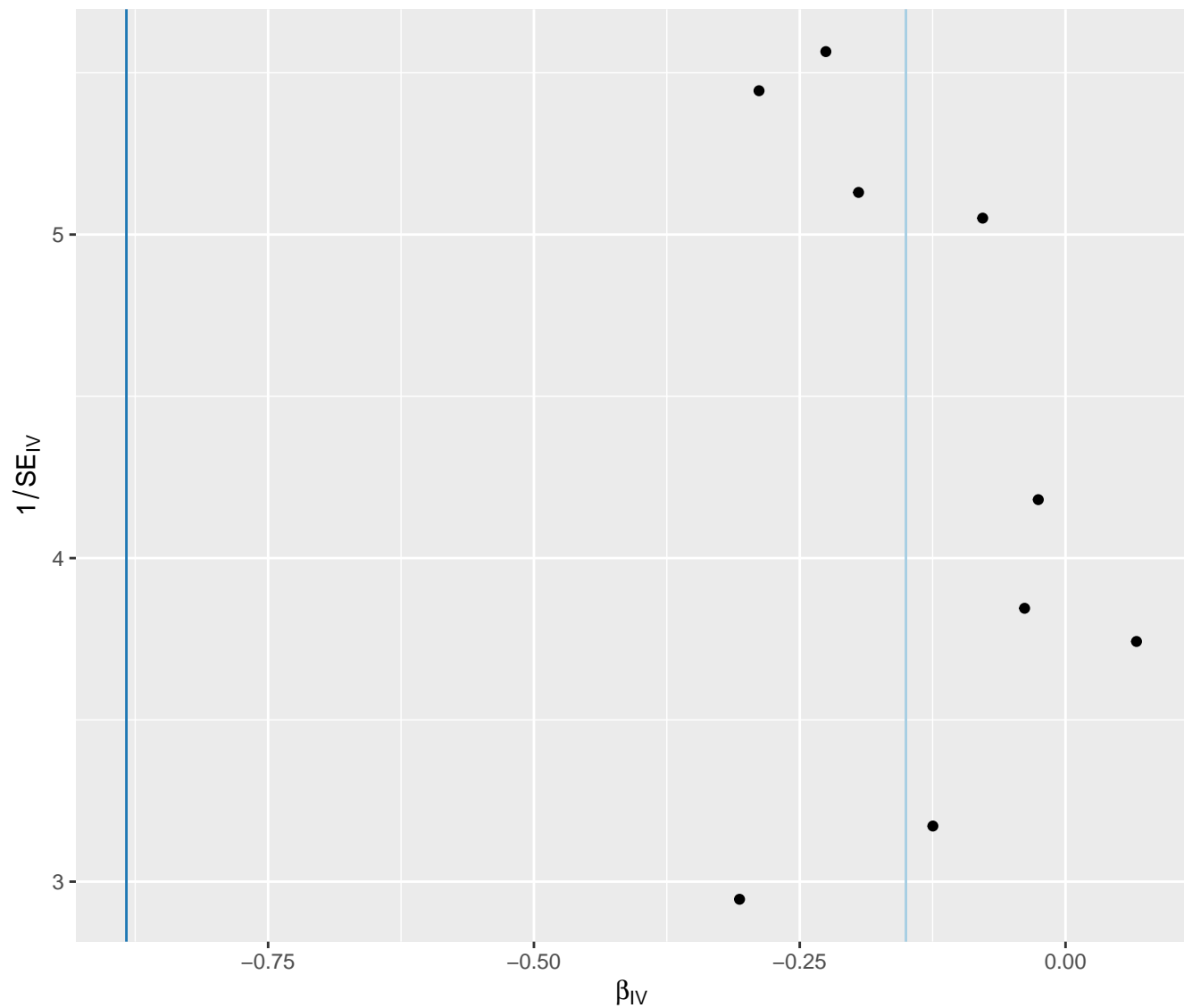

Supplement: Supplementary file 1 [file DataSheet1.ZIP › Supplementary Materials/MR plots for tongue/tongue═╝/Colorectal cancer/pheno.1821_to_colorectal cancer_funnel.pdf]

# MR Test

- Inverse variance weighted
- MR Egger
- Simple mode
- Weighted median
- Weighted mode

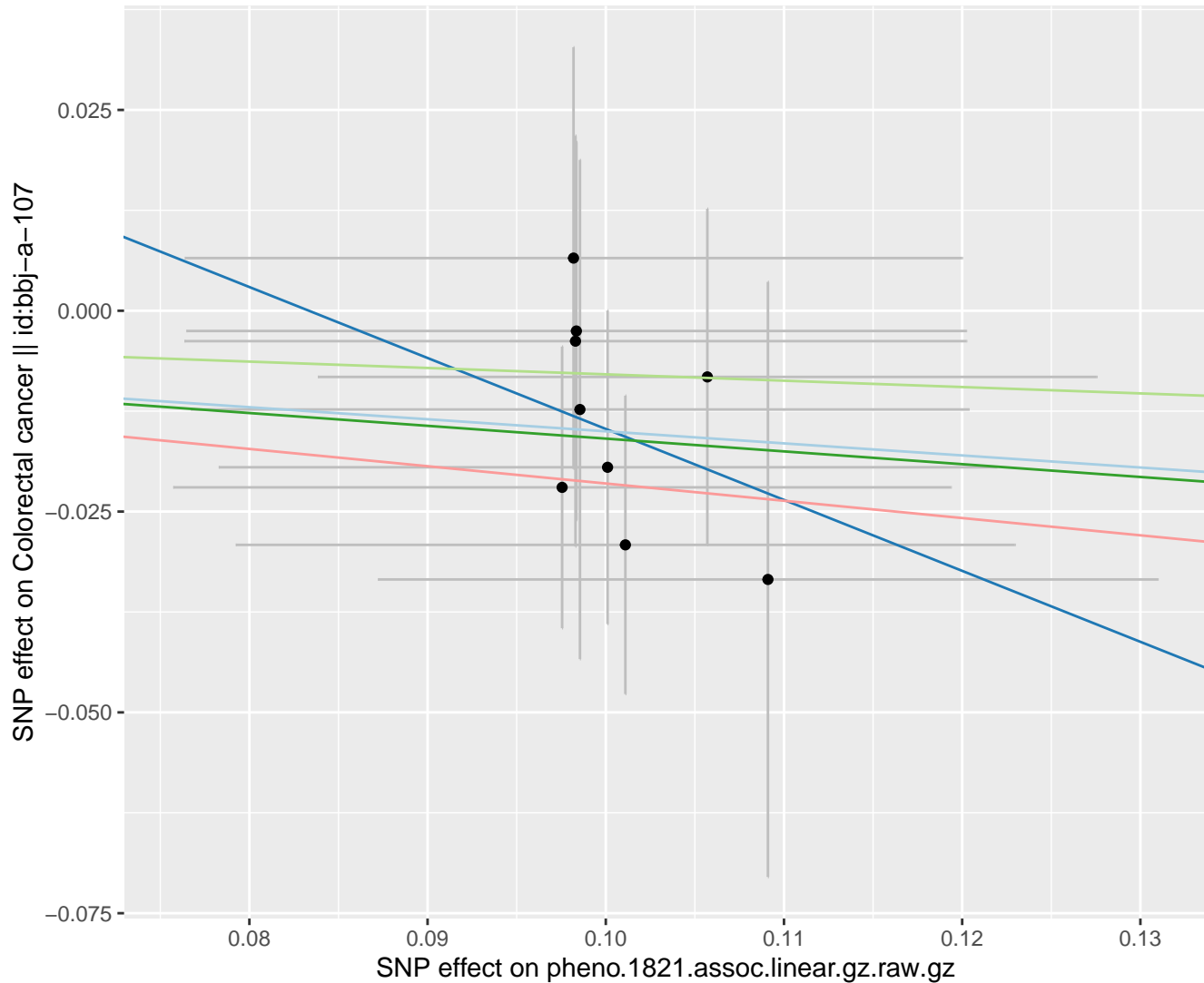

Supplement: Supplementary file 1 [file DataSheet1.ZIP › Supplementary Materials/MR plots for tongue/tongue═╝/Colorectal cancer/pheno.1821_to_colorectal cancer_scatter.pdf]

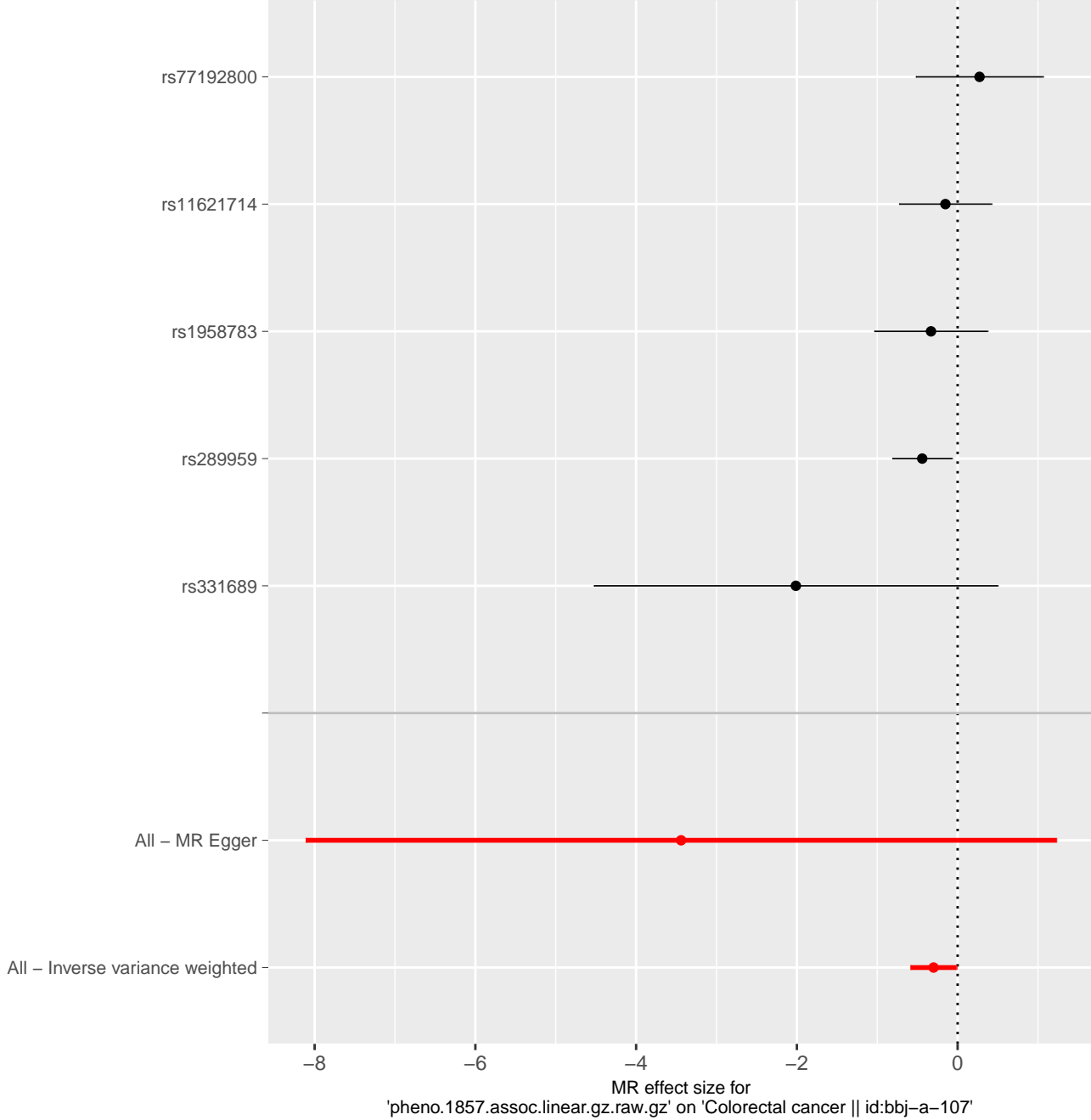

Supplement: Supplementary file 1 [file DataSheet1.ZIP › Supplementary Materials/MR plots for tongue/tongue═╝/Colorectal cancer/pheno.1857_to_colorectal cancer_forest.pdf]

# MR Method

- Inverse variance weighted
- MR Egger

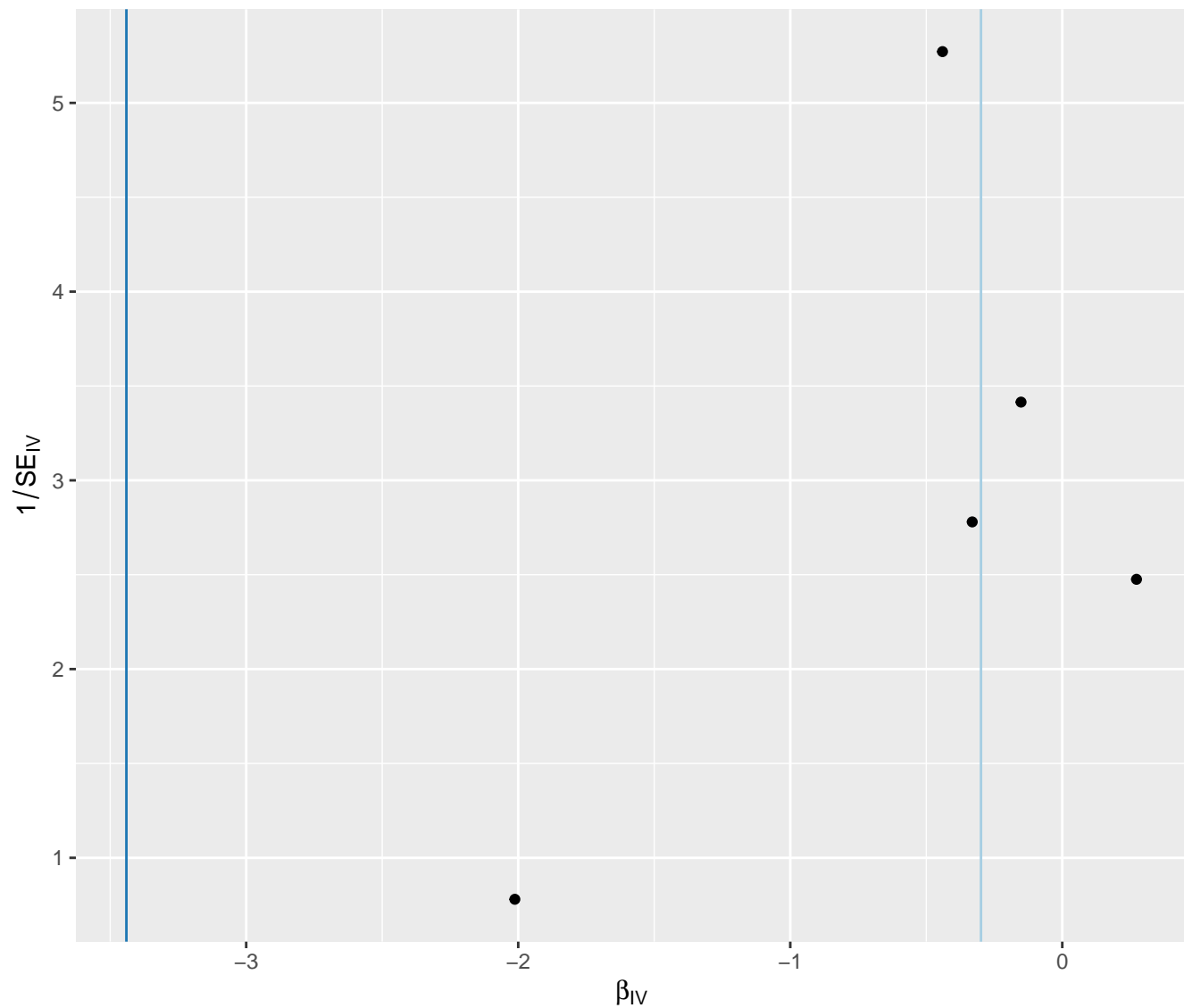

Supplement: Supplementary file 1 [file DataSheet1.ZIP › Supplementary Materials/MR plots for tongue/tongue═╝/Colorectal cancer/pheno.1857_to_colorectal cancer_funnel.pdf]

# MR Test

- Inverse variance weighted
- MR Egger
- Simple mode
- Weighted median
- Weighted mode

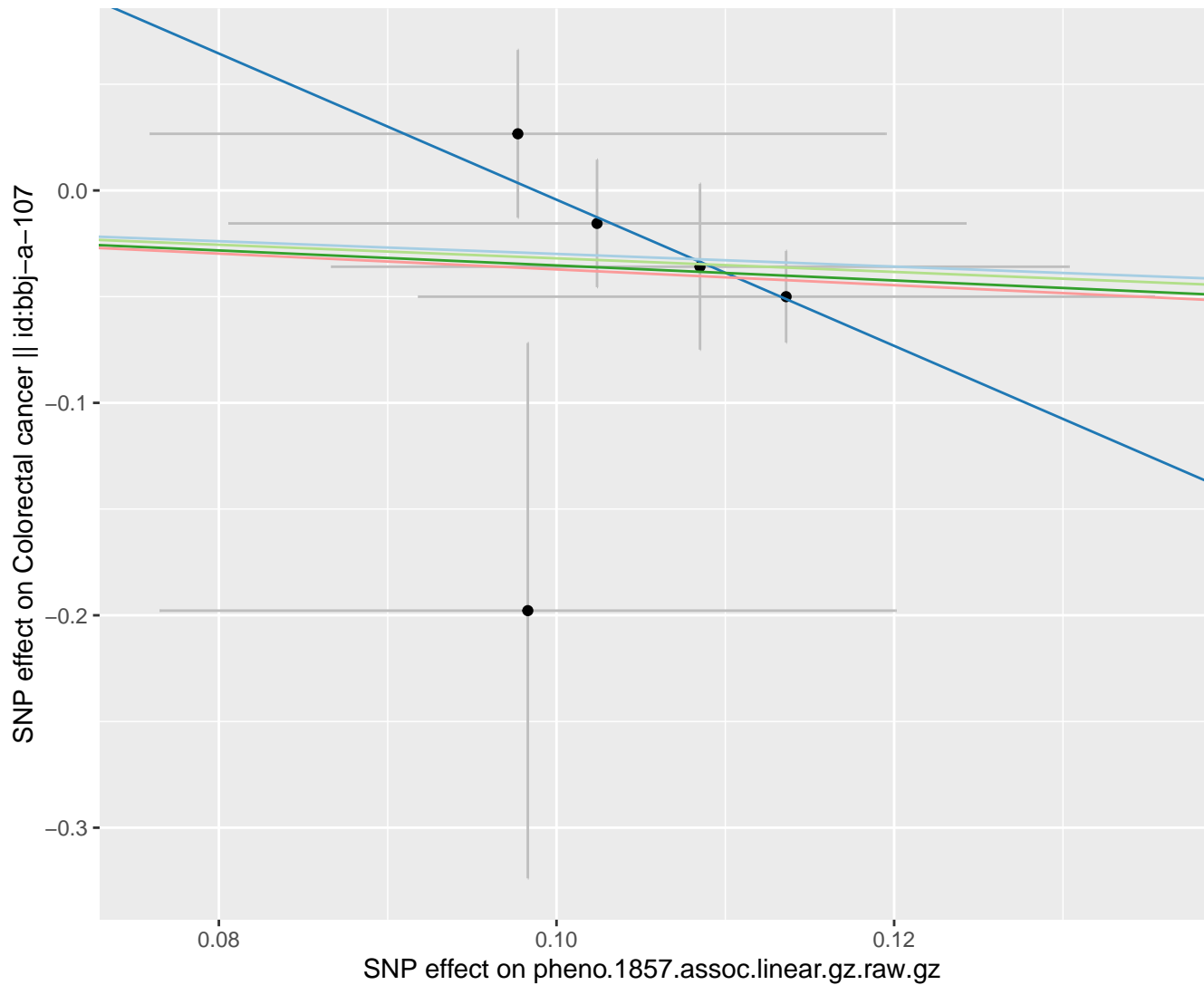

Supplement: Supplementary file 1 [file DataSheet1.ZIP › Supplementary Materials/MR plots for tongue/tongue═╝/Colorectal cancer/pheno.1857_to_colorectal cancer_scatter.pdf]

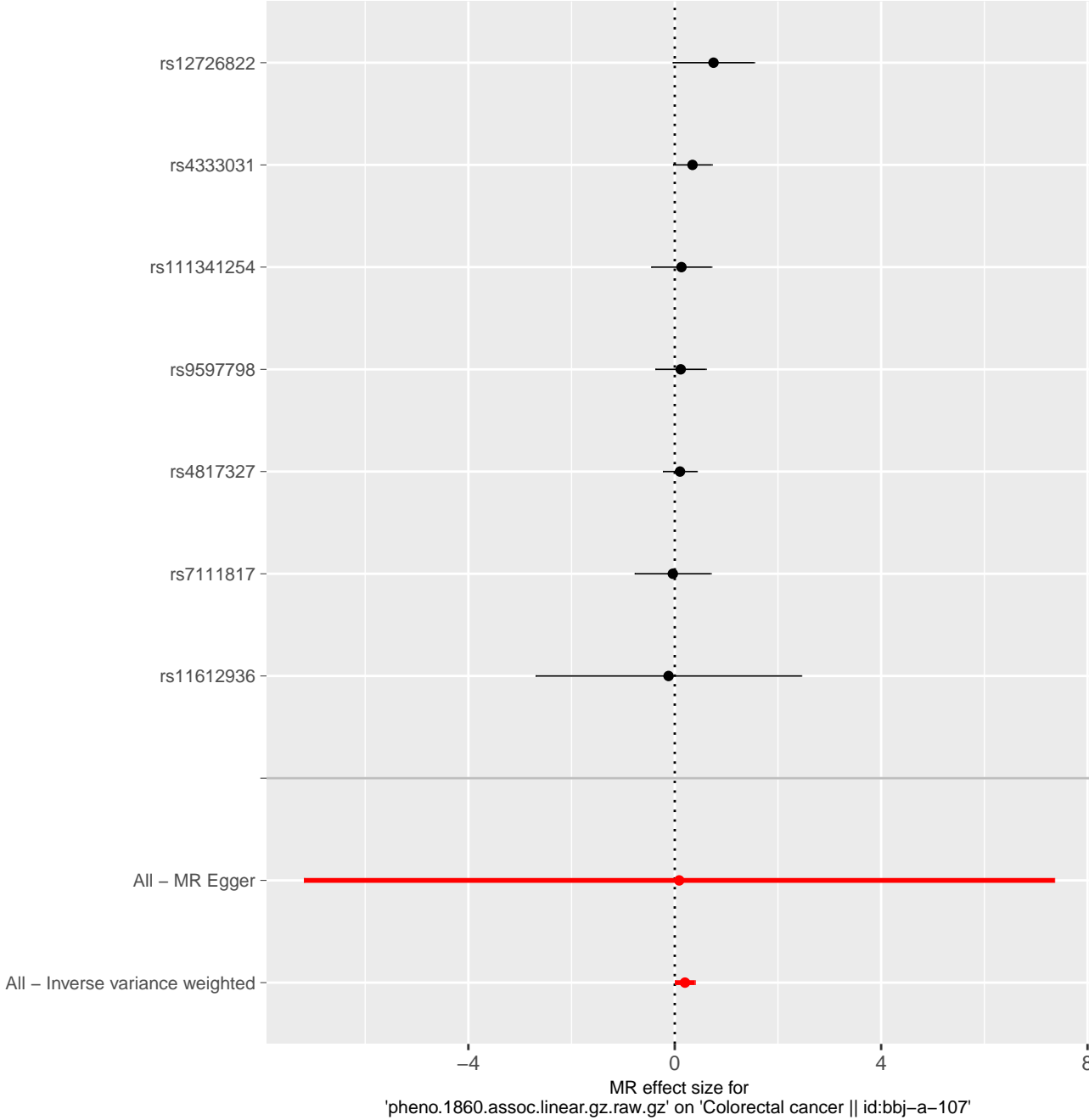

Supplement: Supplementary file 1 [file DataSheet1.ZIP › Supplementary Materials/MR plots for tongue/tongue═╝/Colorectal cancer/pheno.1860_to_colorectal cancer_forest.pdf]

# MR Method

- Inverse variance weighted
- MR Egger

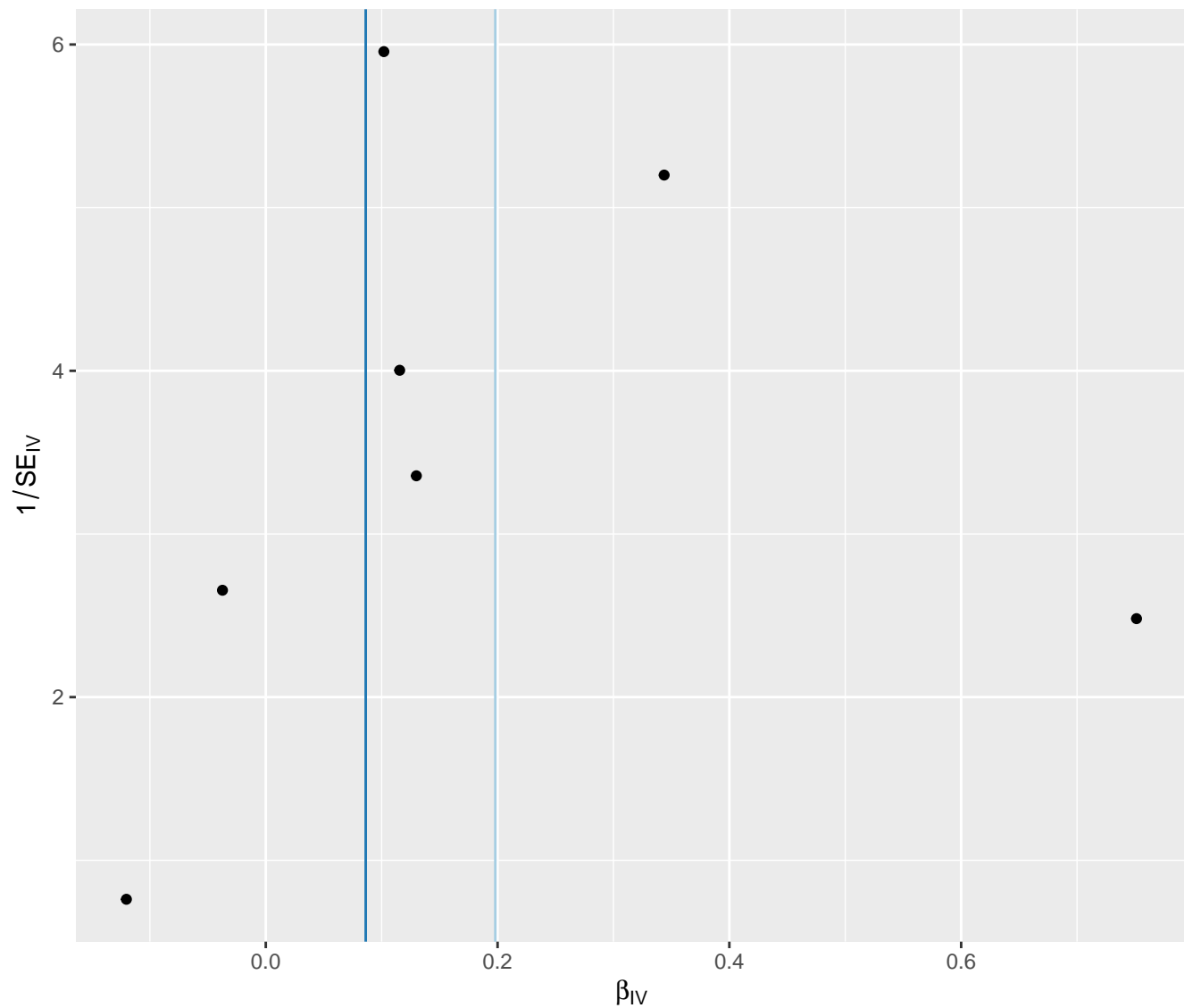

Supplement: Supplementary file 1 [file DataSheet1.ZIP › Supplementary Materials/MR plots for tongue/tongue═╝/Colorectal cancer/pheno.1860_to_colorectal cancer_funnel.pdf]

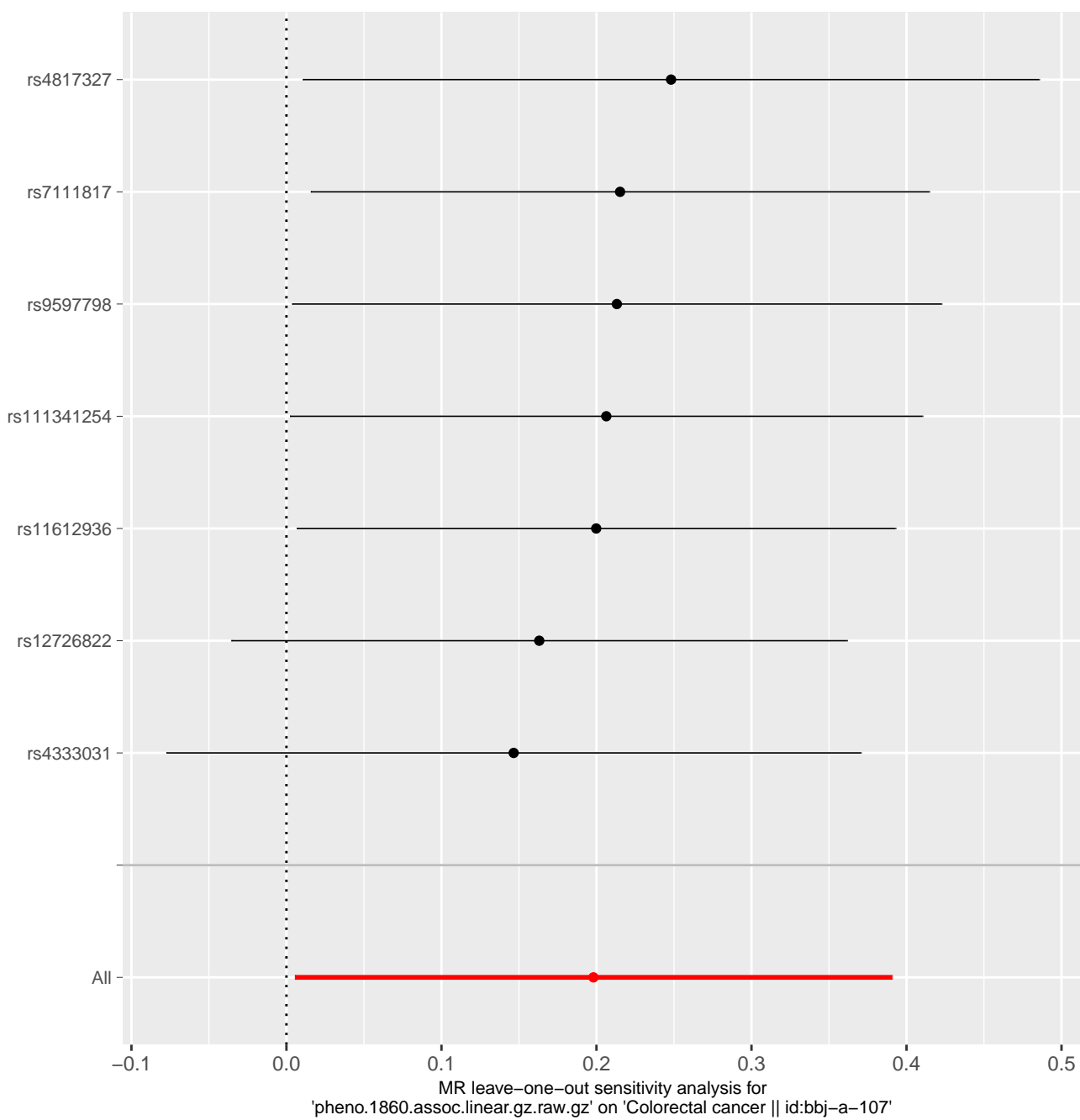

Supplement: Supplementary file 1 [file DataSheet1.ZIP › Supplementary Materials/MR plots for tongue/tongue═╝/Colorectal cancer/pheno.1860_to_colorectal cancer_leave_one_out.pdf]

# MR Test

- Inverse variance weighted
- MR Egger
- Simple mode
- Weighted median
- Weighted mode

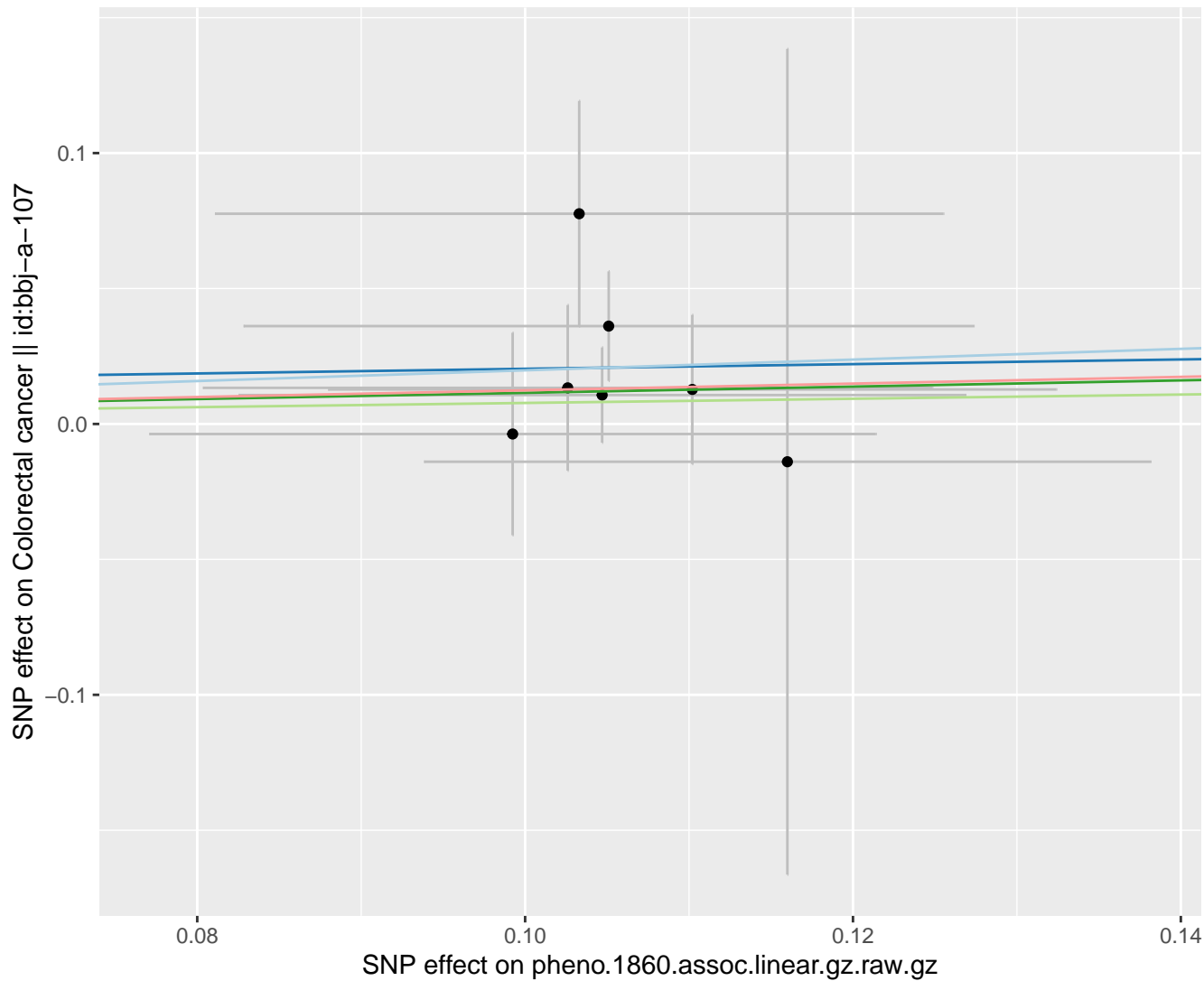

Supplement: Supplementary file 1 [file DataSheet1.ZIP › Supplementary Materials/MR plots for tongue/tongue═╝/Colorectal cancer/pheno.1860_to_colorectal cancer_scatter.pdf]

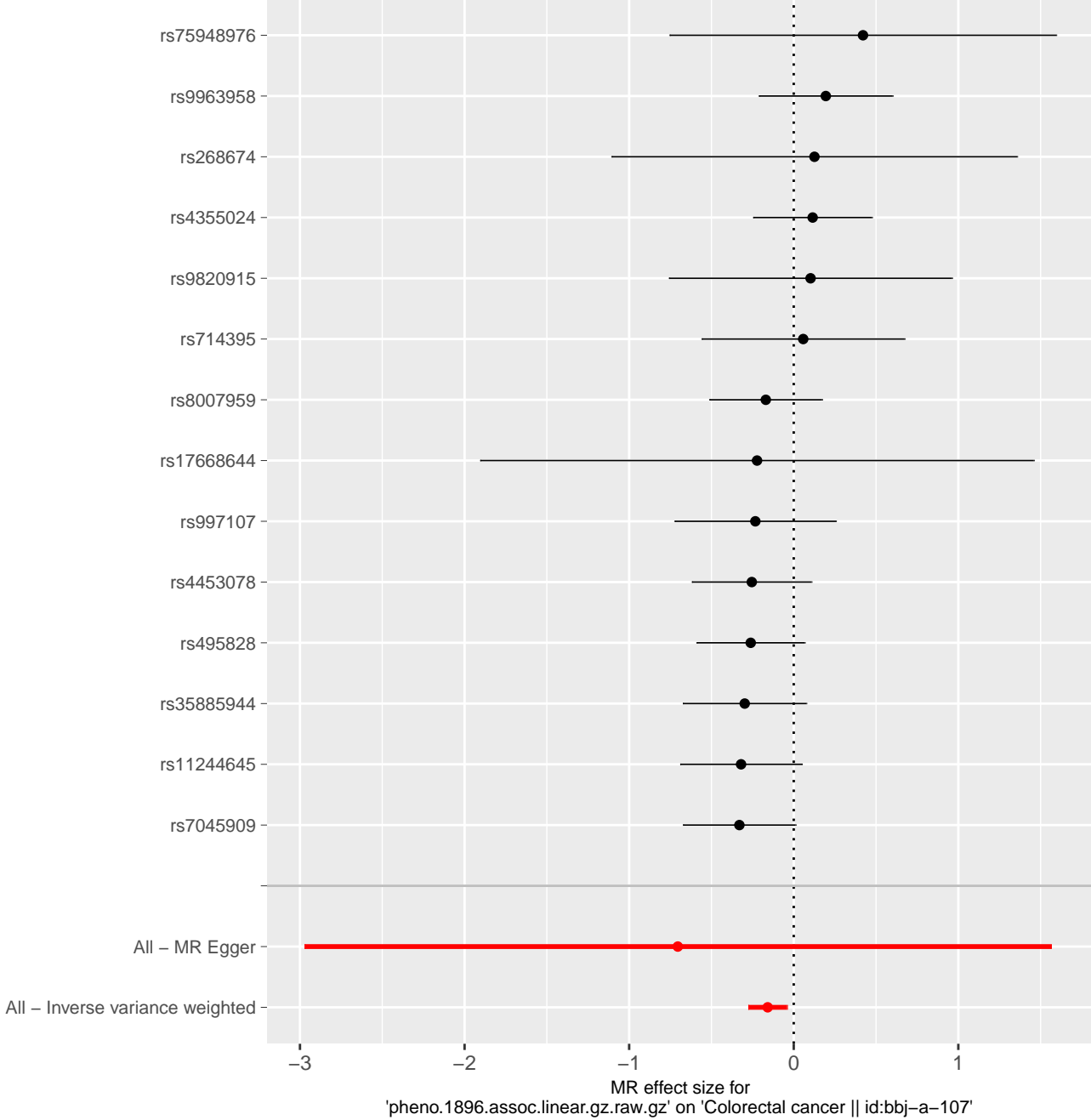

Supplement: Supplementary file 1 [file DataSheet1.ZIP › Supplementary Materials/MR plots for tongue/tongue═╝/Colorectal cancer/pheno.1896_to_colorectal cancer_forest.pdf]

# MR Method

- Inverse variance weighted
- MR Egger

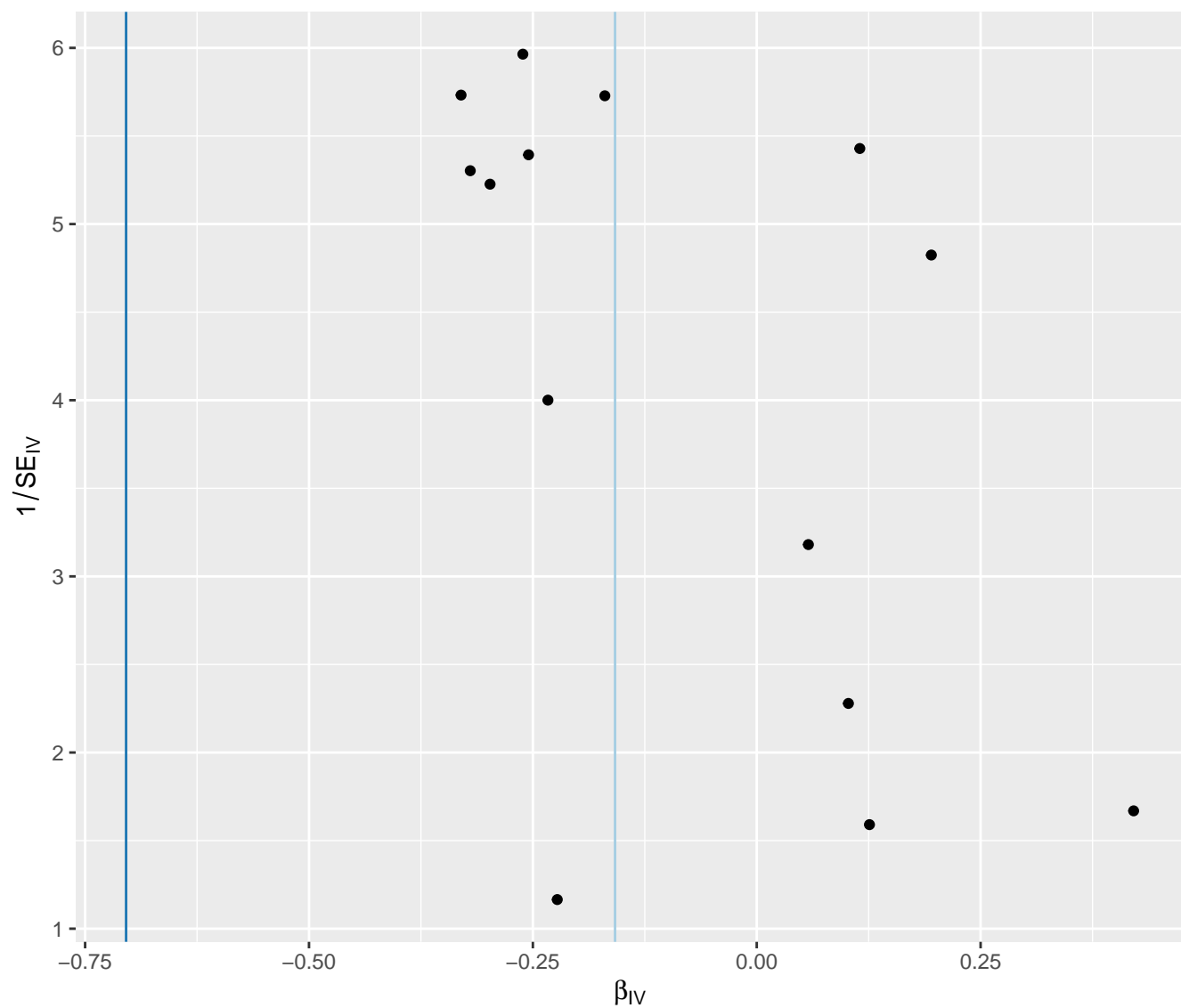

Supplement: Supplementary file 1 [file DataSheet1.ZIP › Supplementary Materials/MR plots for tongue/tongue═╝/Colorectal cancer/pheno.1896_to_colorectal cancer_funnel.pdf]

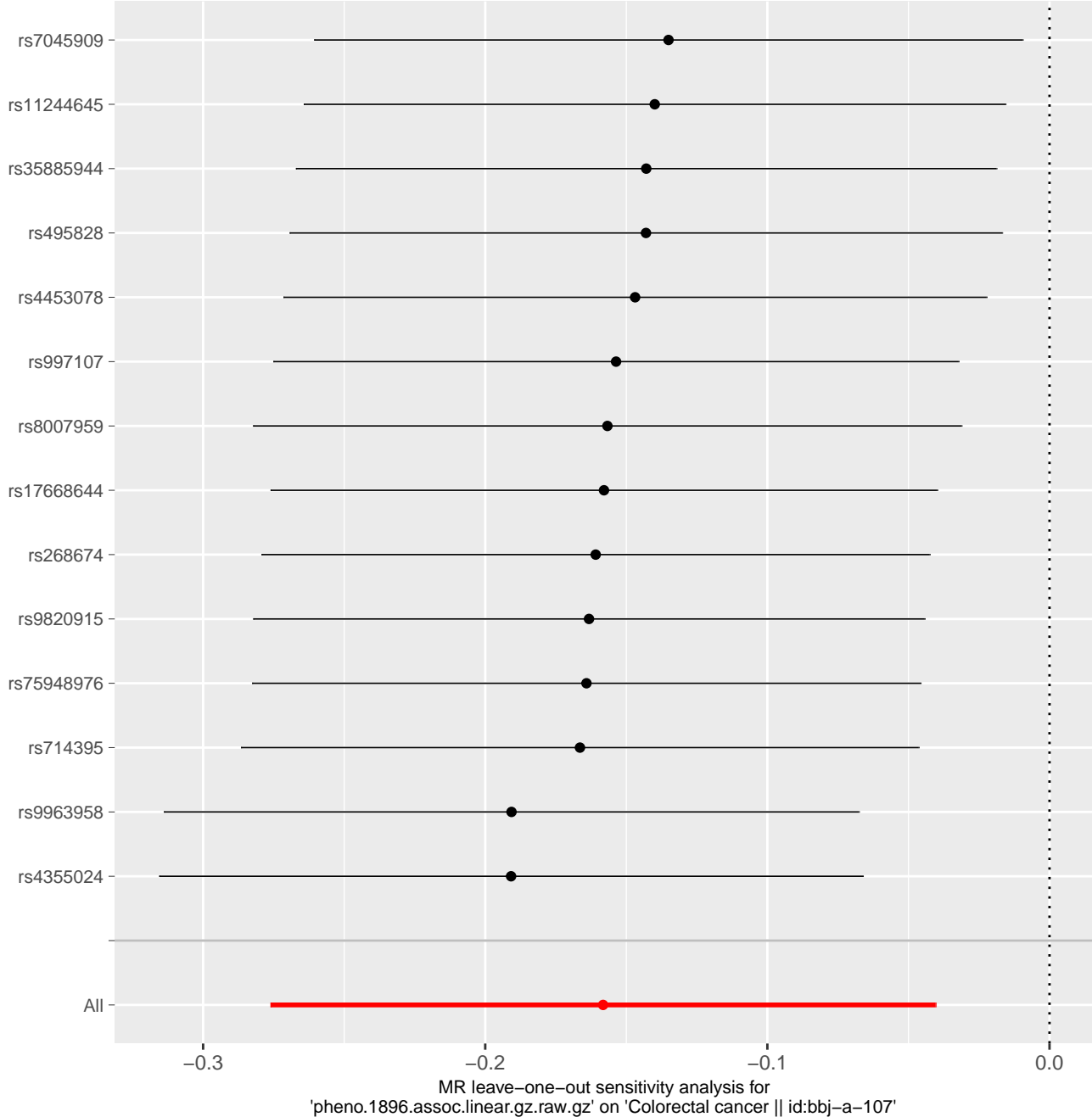

Supplement: Supplementary file 1 [file DataSheet1.ZIP › Supplementary Materials/MR plots for tongue/tongue═╝/Colorectal cancer/pheno.1896_to_colorectal cancer_leave_one_out.pdf]

# MR Test

- Inverse variance weighted
- MR Egger
- Simple mode
- Weighted median
- Weighted mode

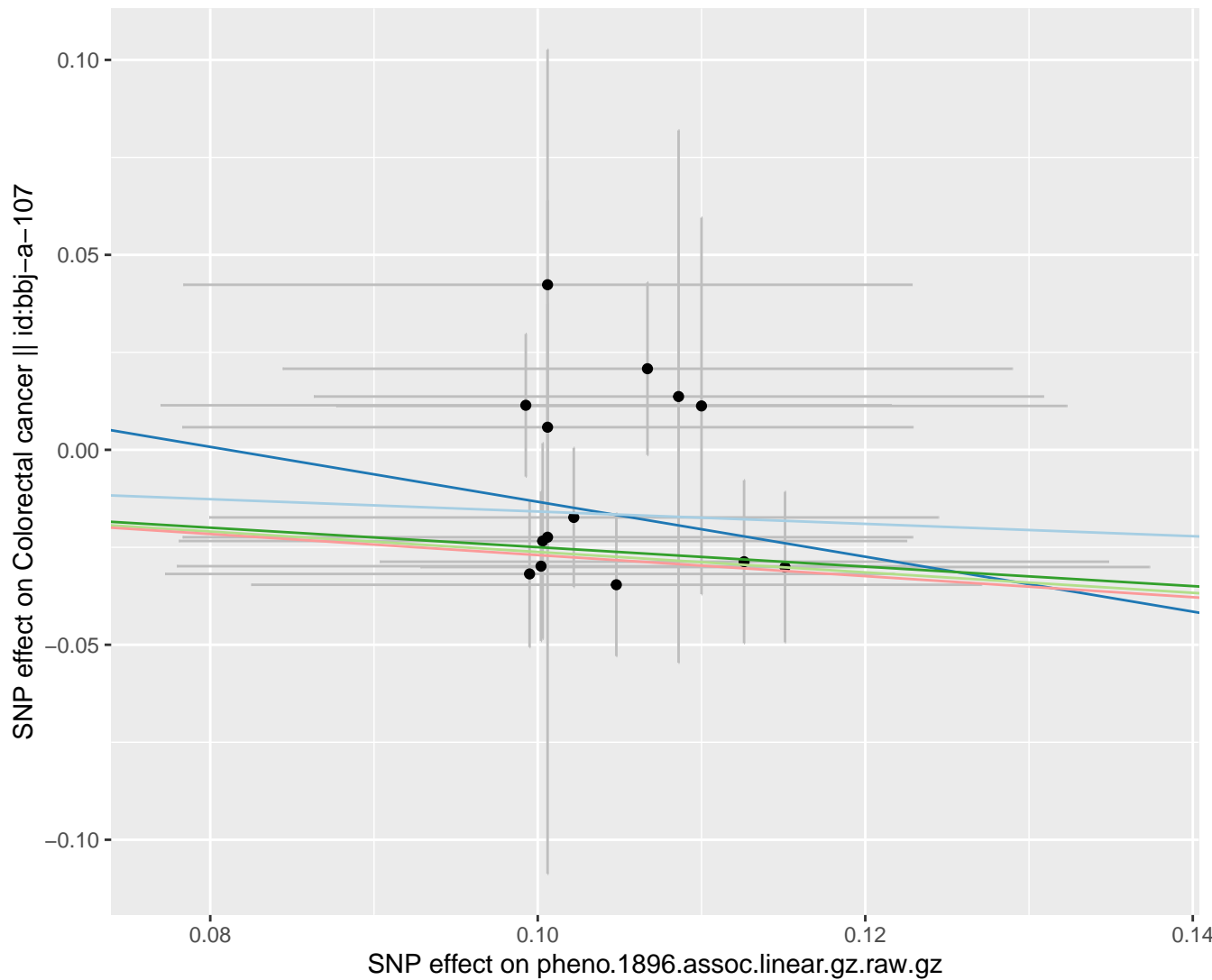

Supplement: Supplementary file 1 [file DataSheet1.ZIP › Supplementary Materials/MR plots for tongue/tongue═╝/Colorectal cancer/pheno.1896_to_colorectal cancer_scatter.pdf]

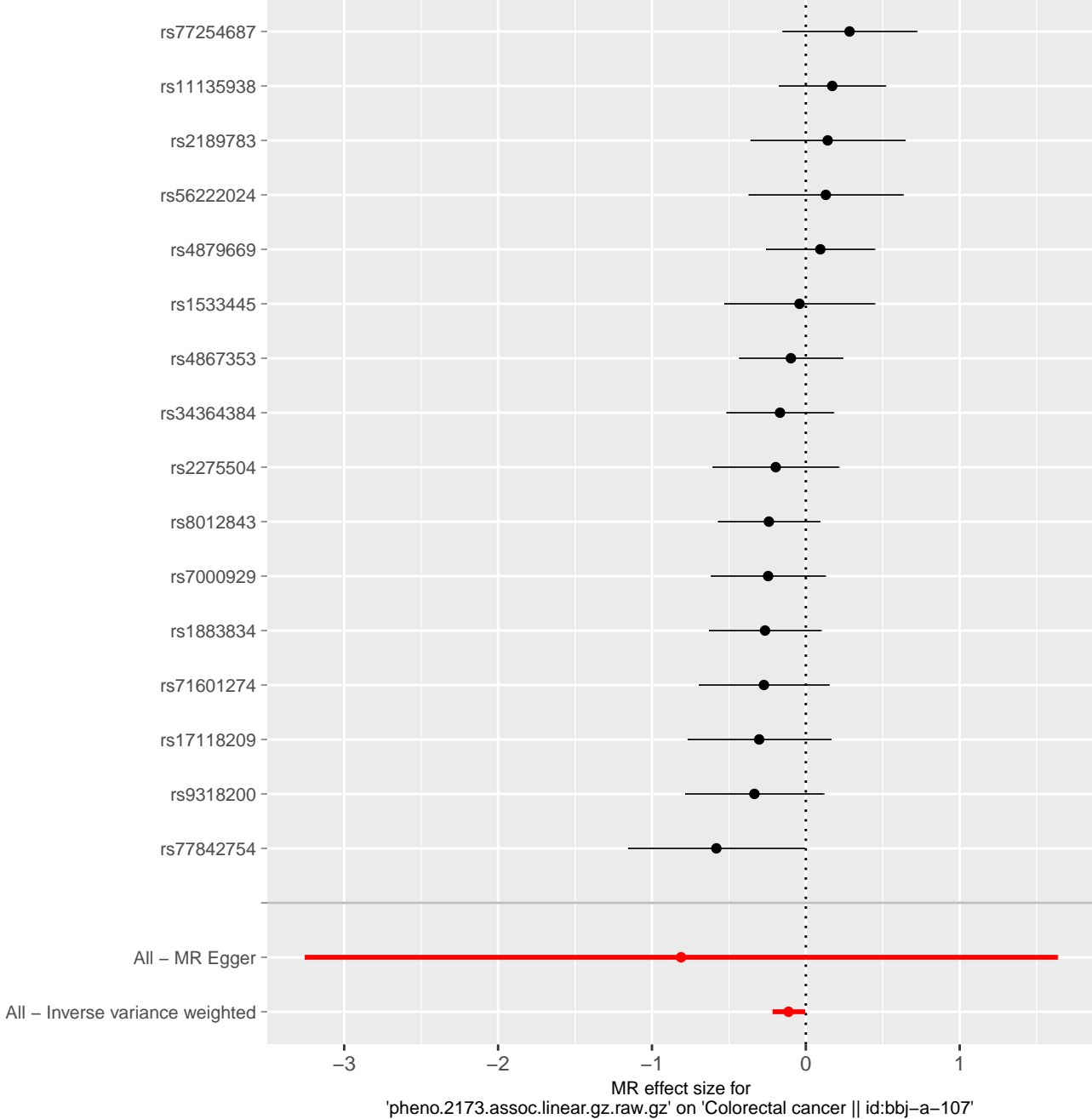

Supplement: Supplementary file 1 [file DataSheet1.ZIP › Supplementary Materials/MR plots for tongue/tongue═╝/Colorectal cancer/pheno.2173_to_colorectal cancer_forest.pdf]

# MR Method

- Inverse variance weighted
- MR Egger

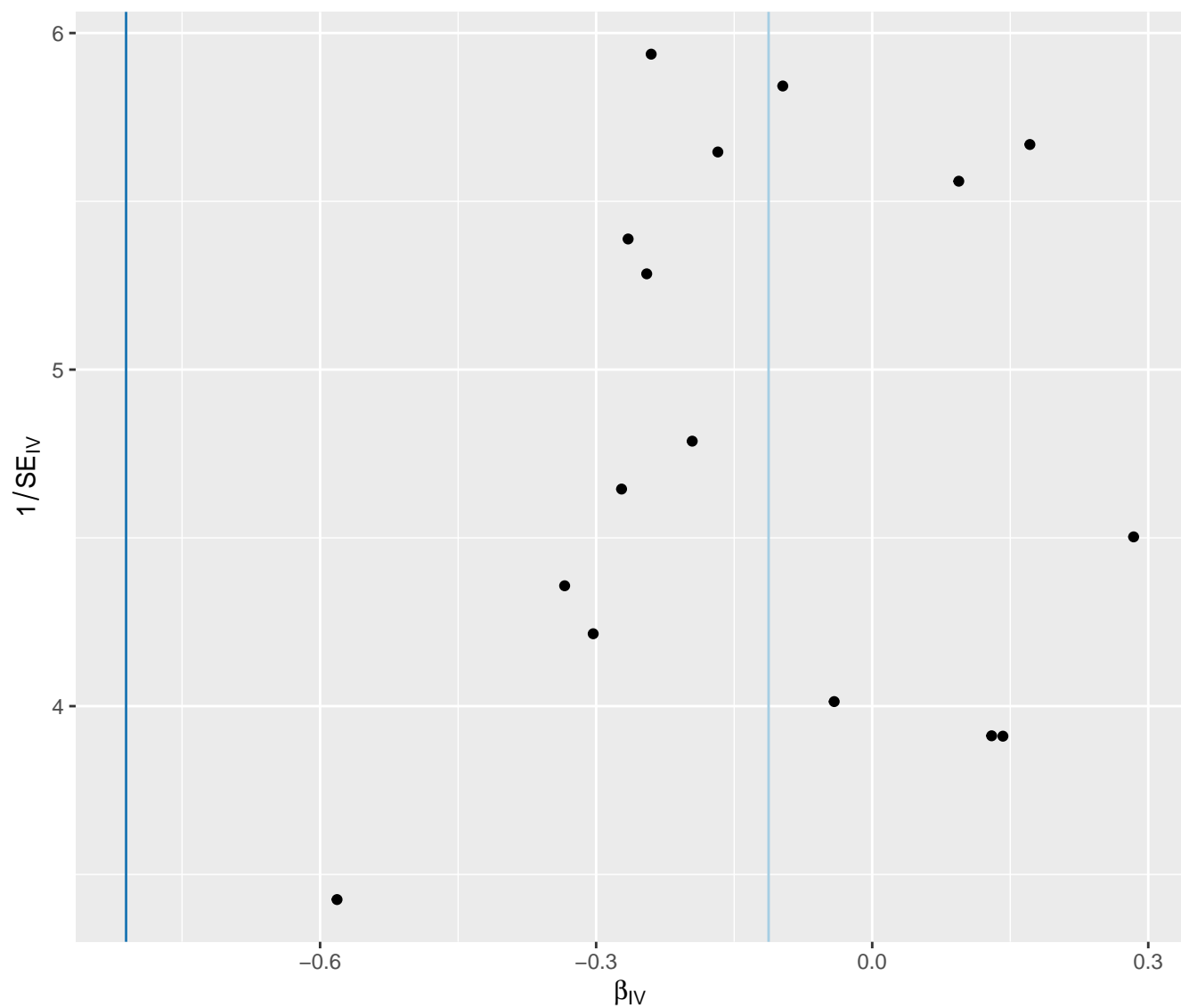

Supplement: Supplementary file 1 [file DataSheet1.ZIP › Supplementary Materials/MR plots for tongue/tongue═╝/Colorectal cancer/pheno.2173_to_colorectal cancer_funnel.pdf]

# MR Test

- Inverse variance weighted
- MR Egger
- Simple mode
- Weighted median
- Weighted mode

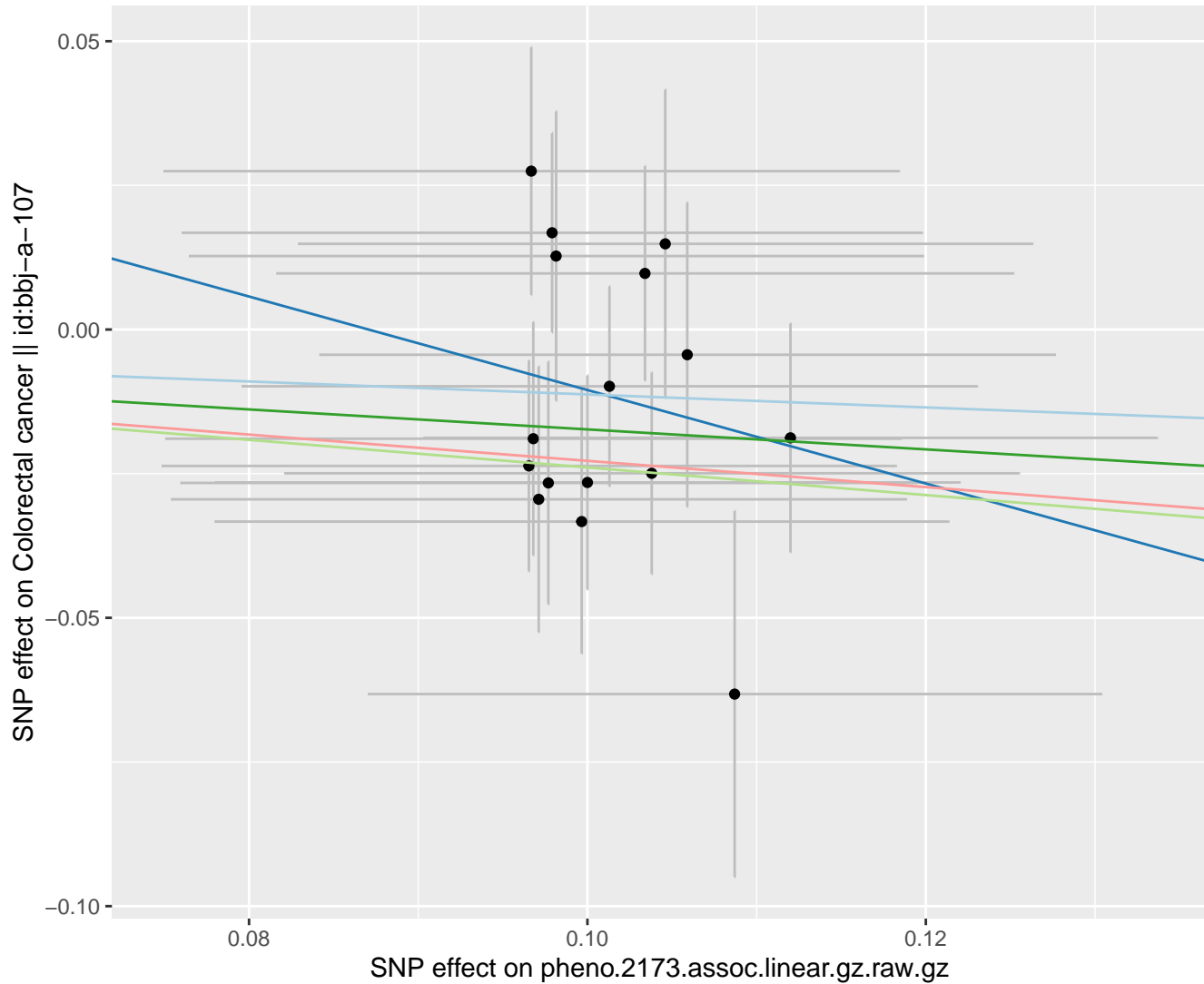

Supplement: Supplementary file 1 [file DataSheet1.ZIP › Supplementary Materials/MR plots for tongue/tongue═╝/Colorectal cancer/pheno.2173_to_colorectal cancer_scatter.pdf]

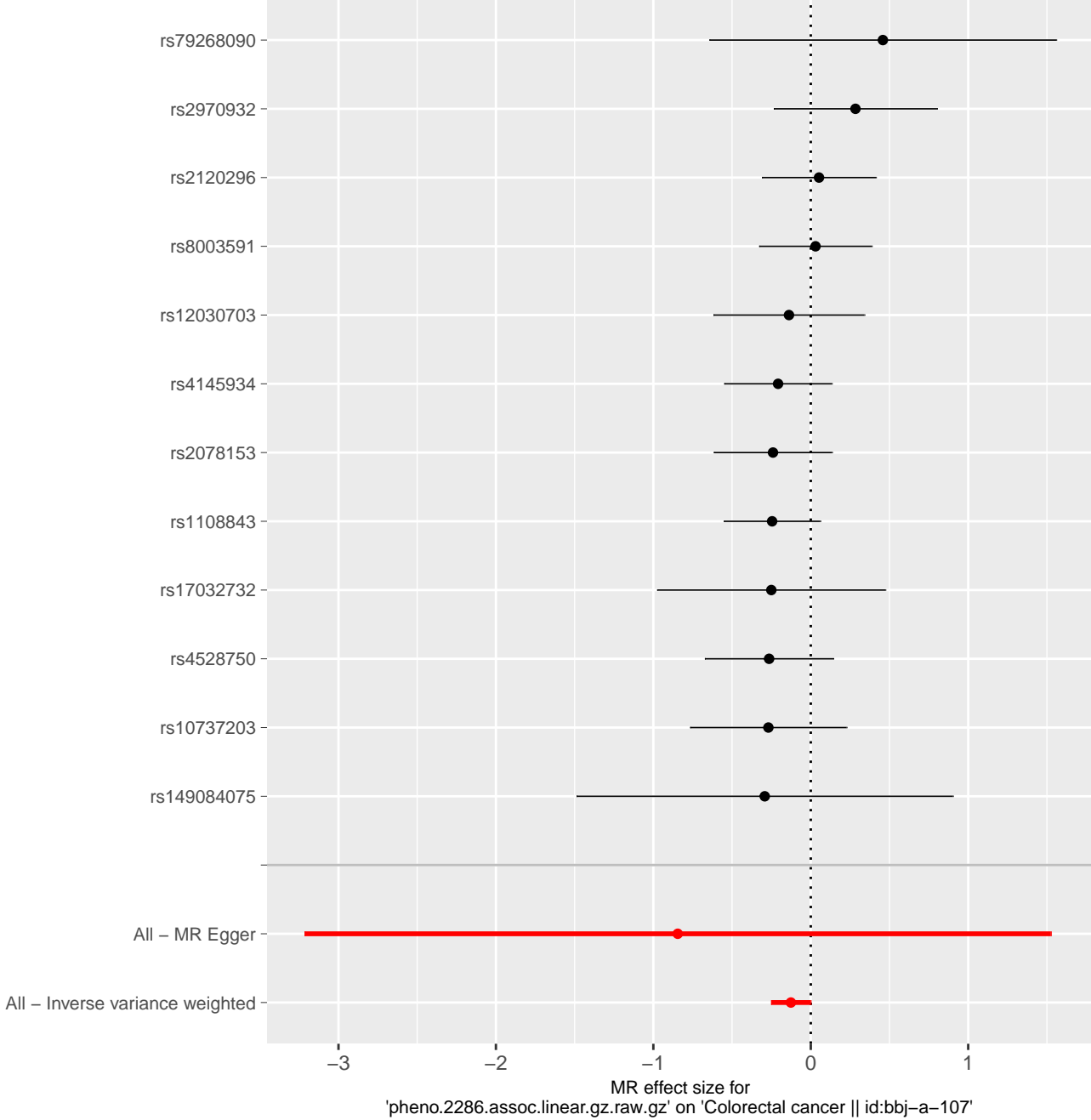

Supplement: Supplementary file 1 [file DataSheet1.ZIP › Supplementary Materials/MR plots for tongue/tongue═╝/Colorectal cancer/pheno.2286_to_colorectal cancer_forest.pdf]

# MR Method

- Inverse variance weighted
- MR Egger

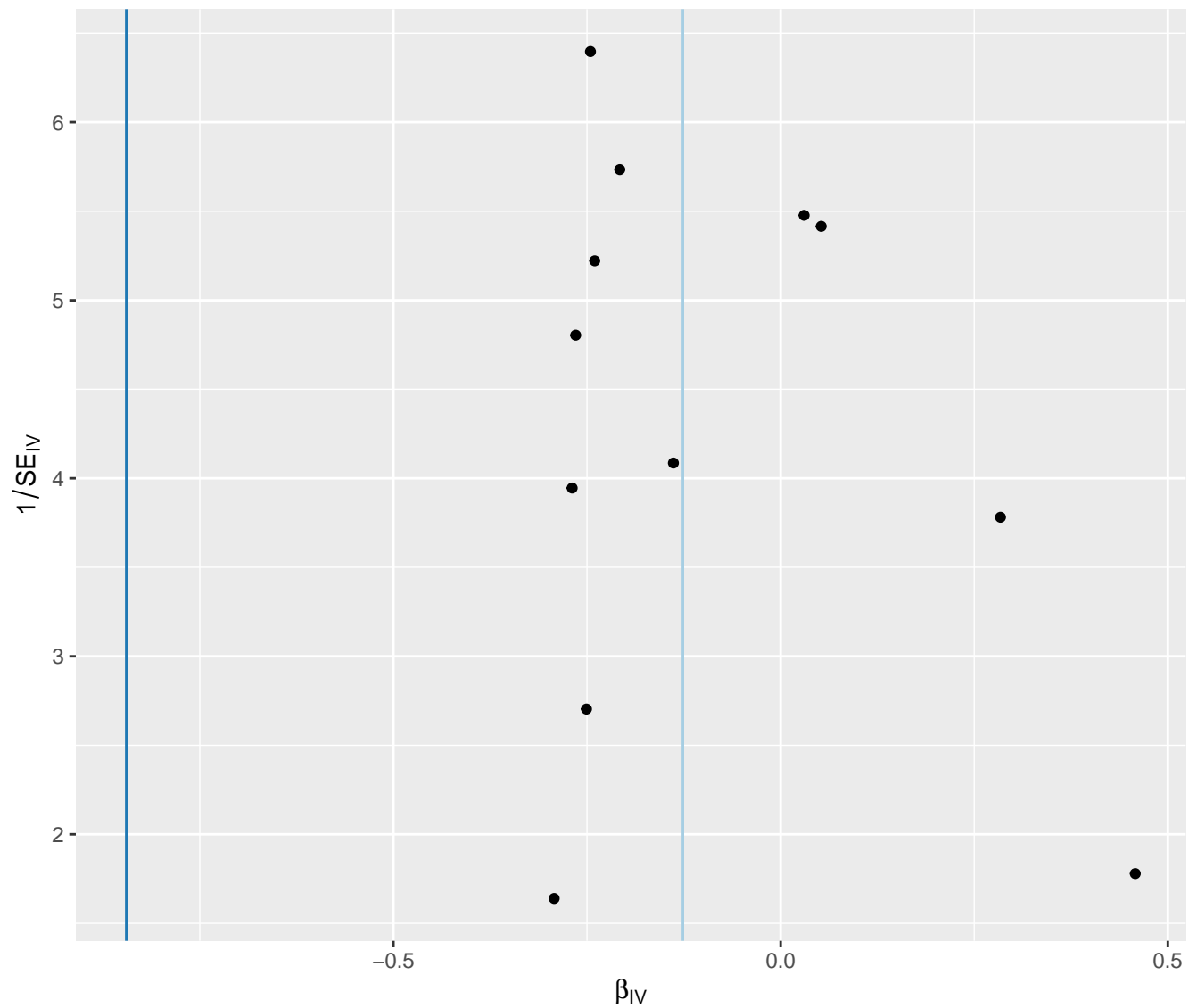

Supplement: Supplementary file 1 [file DataSheet1.ZIP › Supplementary Materials/MR plots for tongue/tongue═╝/Colorectal cancer/pheno.2286_to_colorectal cancer_funnel.pdf]

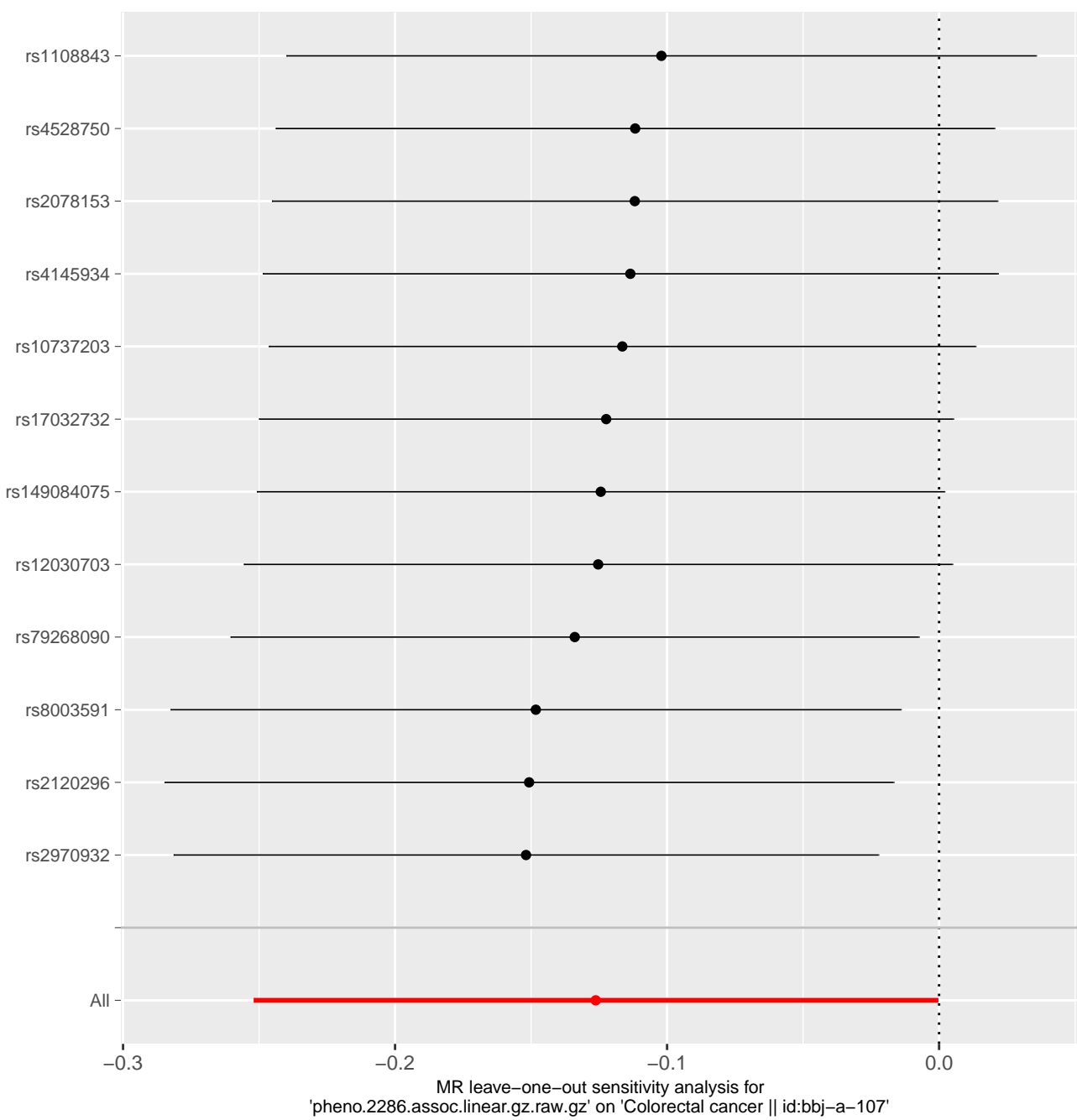

Supplement: Supplementary file 1 [file DataSheet1.ZIP › Supplementary Materials/MR plots for tongue/tongue═╝/Colorectal cancer/pheno.2286_to_colorectal cancer_leave_one_out.pdf]

# MR Test

- Inverse variance weighted
- MR Egger
- Simple mode
- Weighted median
- Weighted mode

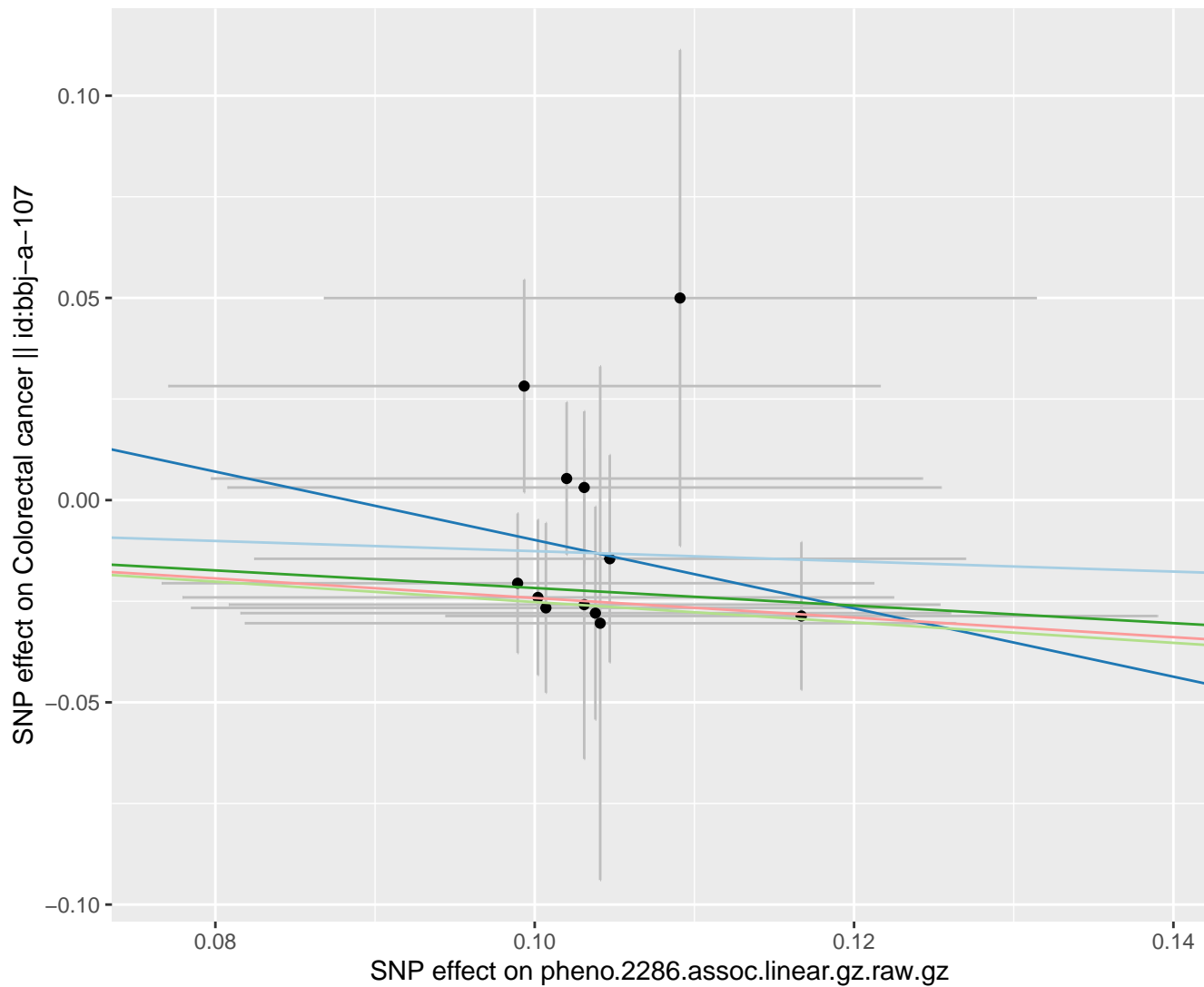

Supplement: Supplementary file 1 [file DataSheet1.ZIP › Supplementary Materials/MR plots for tongue/tongue═╝/Colorectal cancer/pheno.2286_to_colorectal cancer_scatter.pdf]

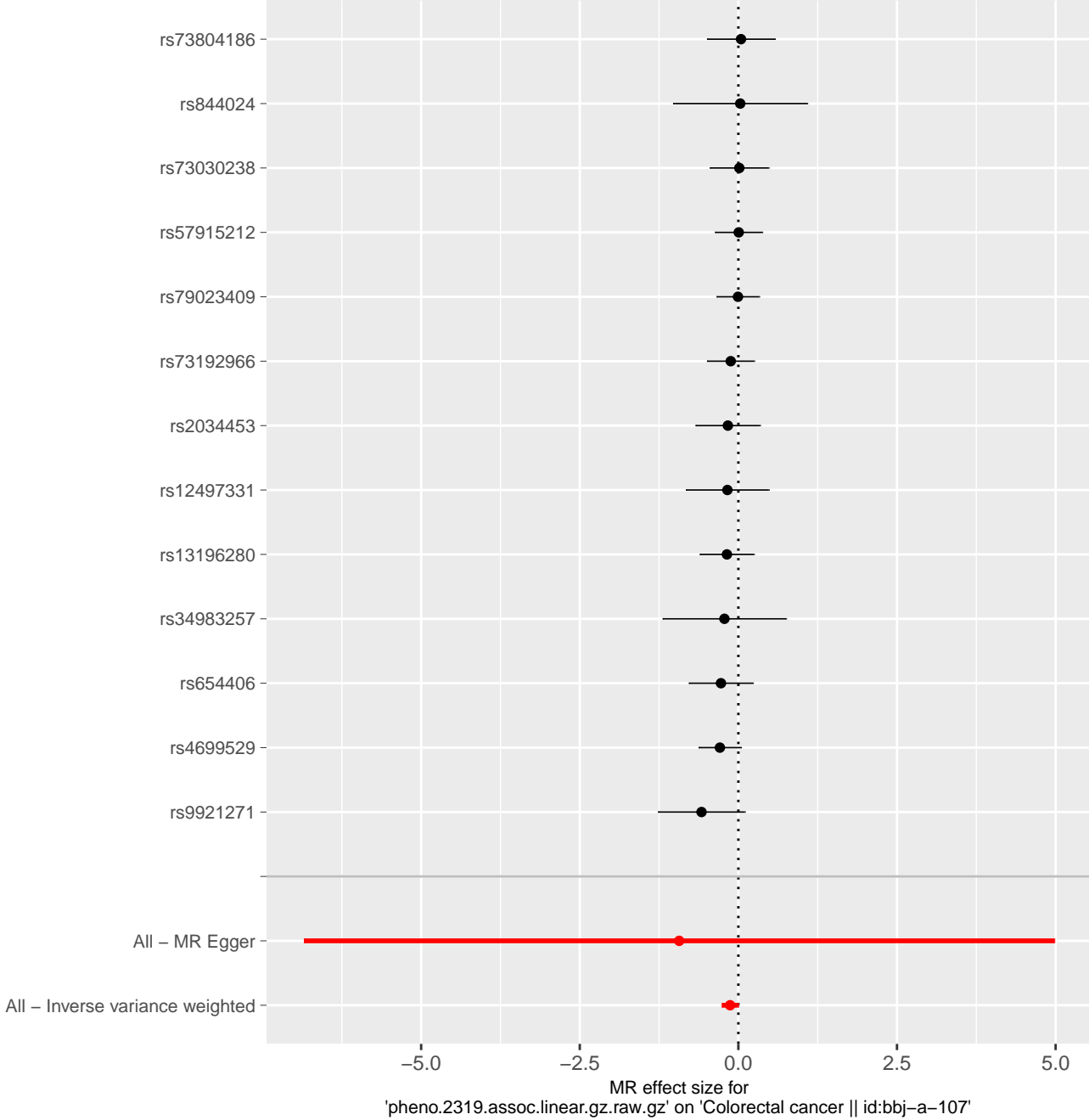

Supplement: Supplementary file 1 [file DataSheet1.ZIP › Supplementary Materials/MR plots for tongue/tongue═╝/Colorectal cancer/pheno.2319_to_colorectal cancer_forest.pdf]

# MR Method

- Inverse variance weighted
- MR Egger

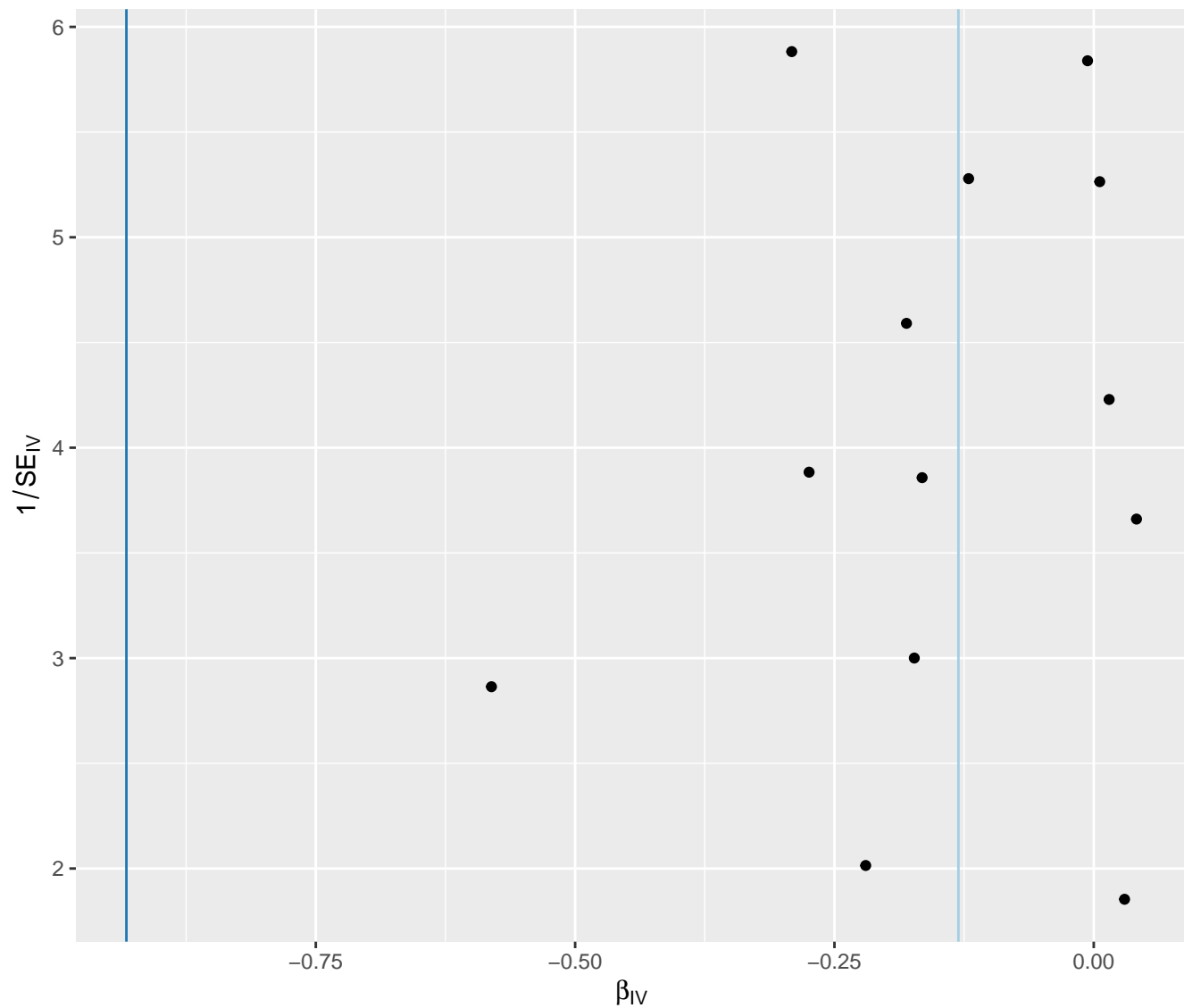

Supplement: Supplementary file 1 [file DataSheet1.ZIP › Supplementary Materials/MR plots for tongue/tongue═╝/Colorectal cancer/pheno.2319_to_colorectal cancer_funnel.pdf]

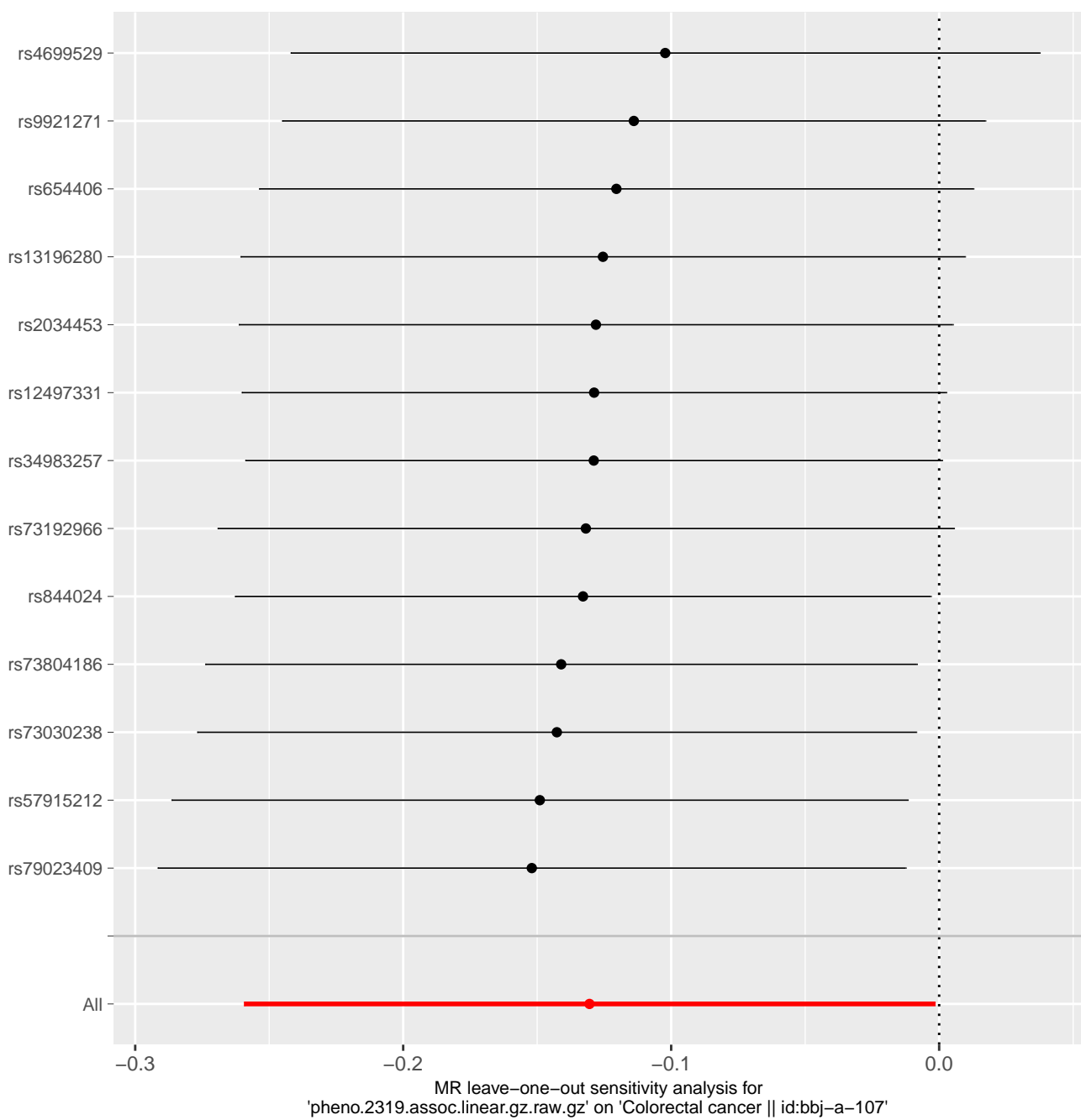

Supplement: Supplementary file 1 [file DataSheet1.ZIP › Supplementary Materials/MR plots for tongue/tongue═╝/Colorectal cancer/pheno.2319_to_colorectal cancer_leave_one_out.pdf]

# MR Test

- Inverse variance weighted
- MR Egger
- Simple mode
- Weighted median
- Weighted mode

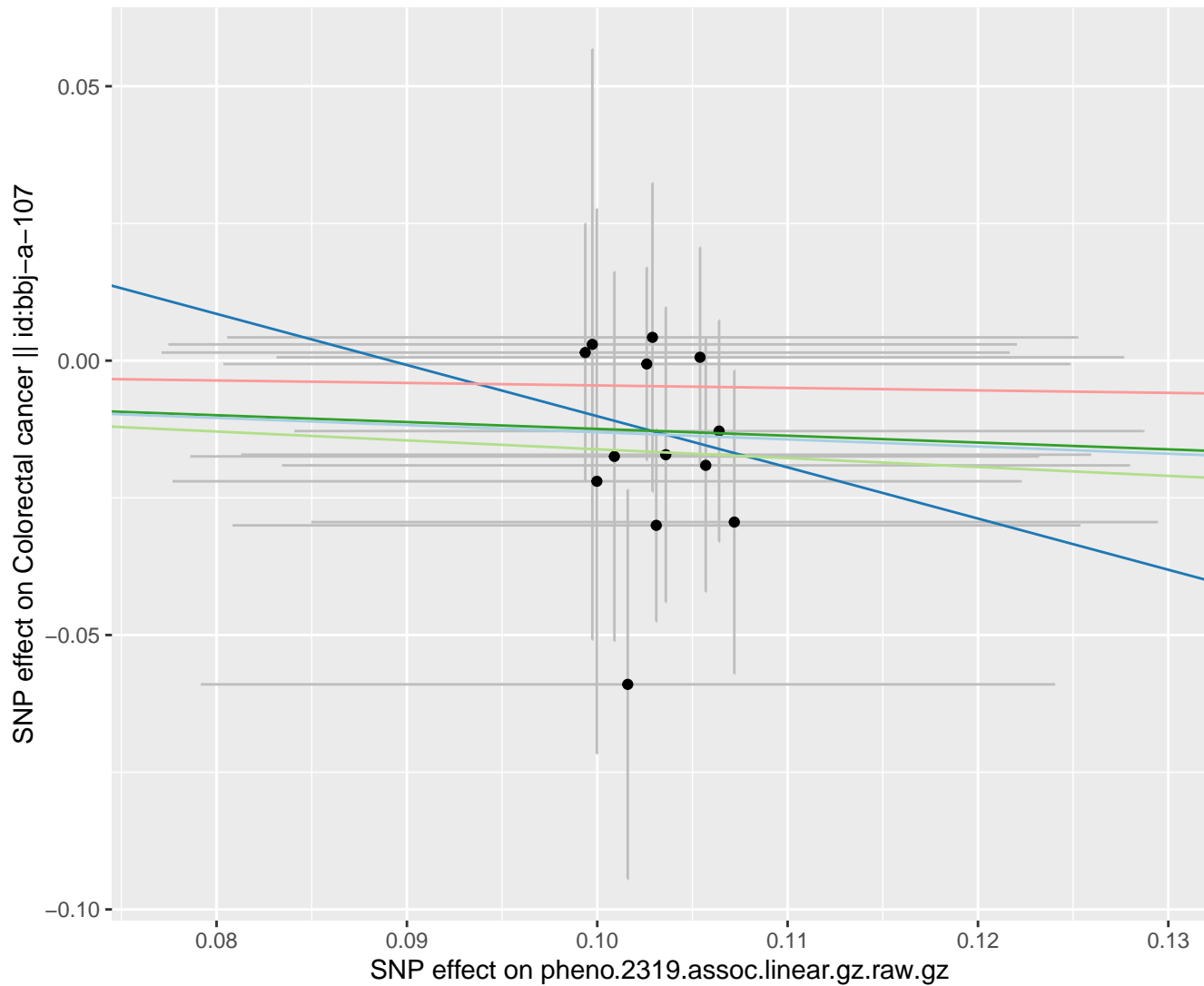

Supplement: Supplementary file 1 [file DataSheet1.ZIP › Supplementary Materials/MR plots for tongue/tongue═╝/Colorectal cancer/pheno.2319_to_colorectal cancer_scatter.pdf]

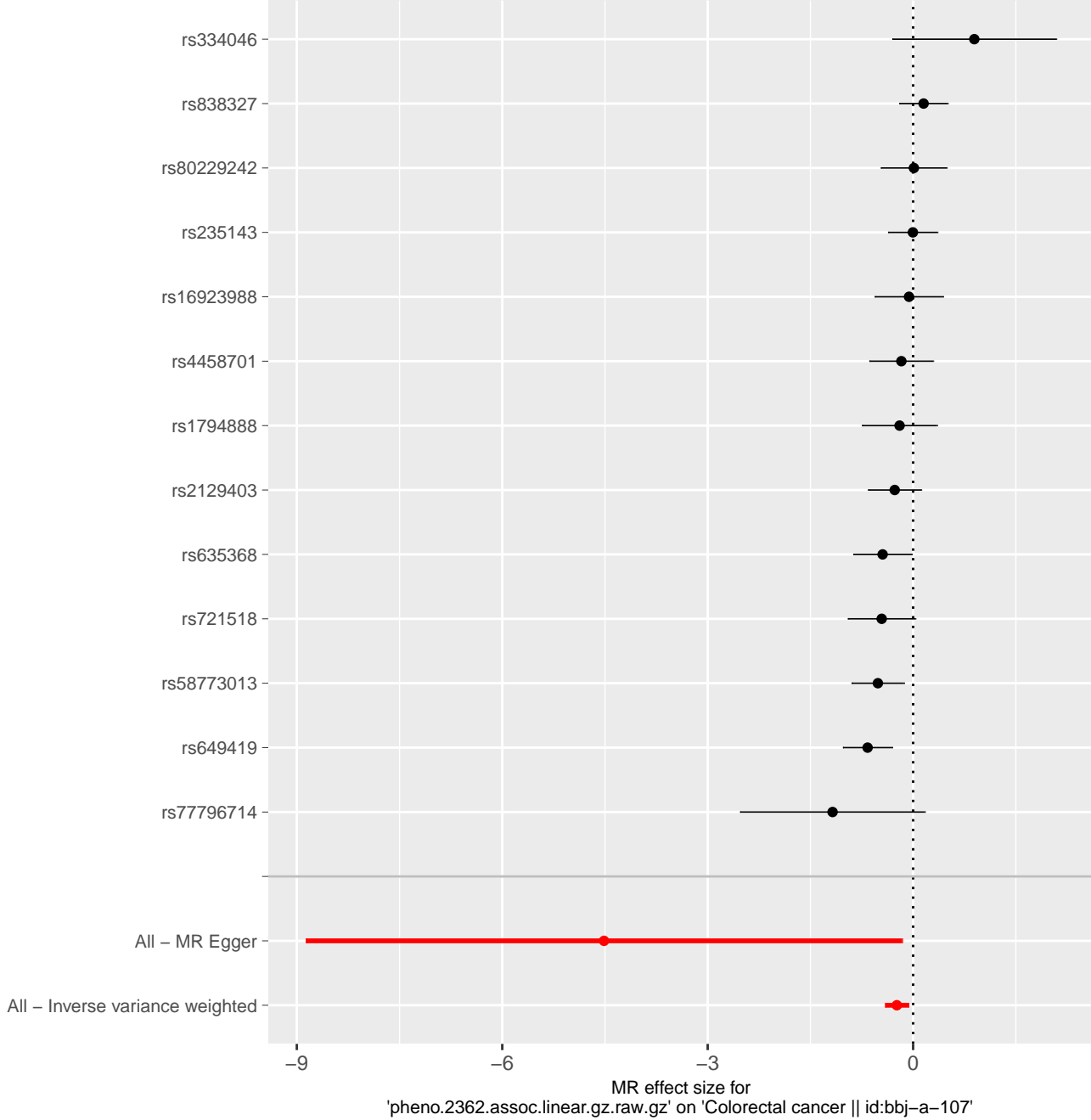

Supplement: Supplementary file 1 [file DataSheet1.ZIP › Supplementary Materials/MR plots for tongue/tongue═╝/Colorectal cancer/pheno.2362_to_colorectal cancer_forest.pdf]

# MR Method

- Inverse variance weighted
- MR Egger

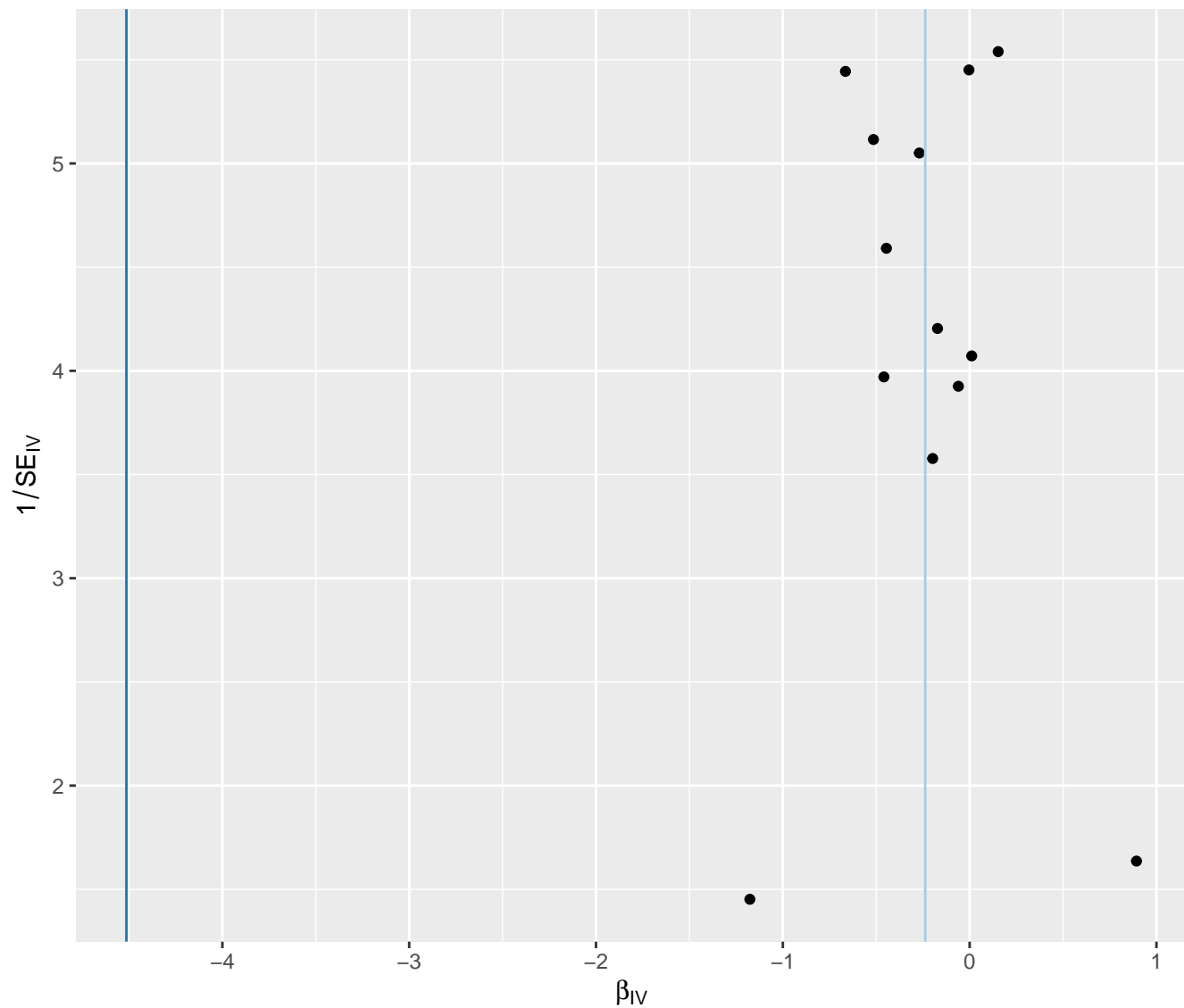

Supplement: Supplementary file 1 [file DataSheet1.ZIP › Supplementary Materials/MR plots for tongue/tongue═╝/Colorectal cancer/pheno.2362_to_colorectal cancer_funnel.pdf]

# MR Test

- Inverse variance weighted
- MR Egger
- Simple mode
- Weighted median
- Weighted mode

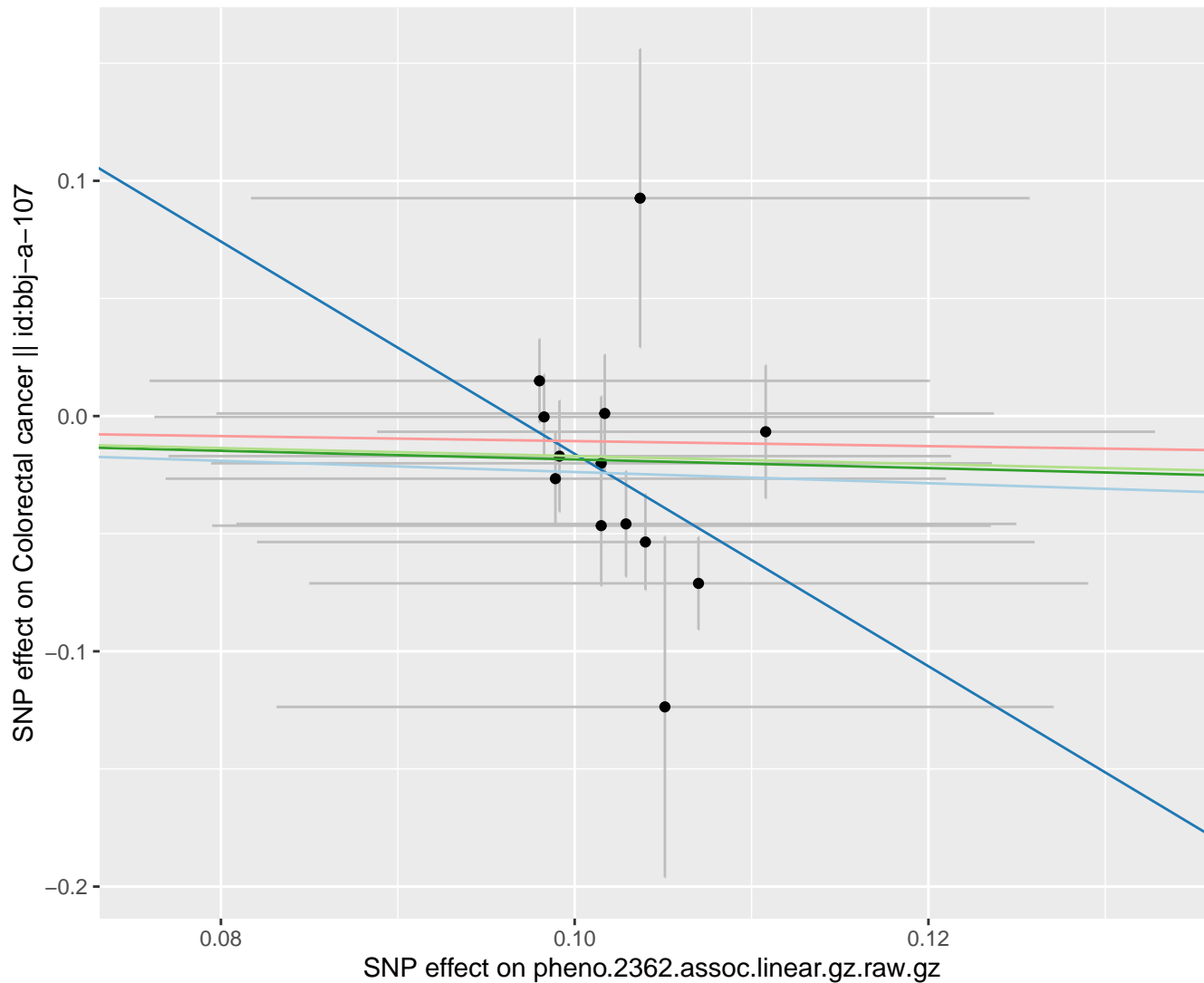

Supplement: Supplementary file 1 [file DataSheet1.ZIP › Supplementary Materials/MR plots for tongue/tongue═╝/Colorectal cancer/pheno.2362_to_colorectal cancer_scatter.pdf]

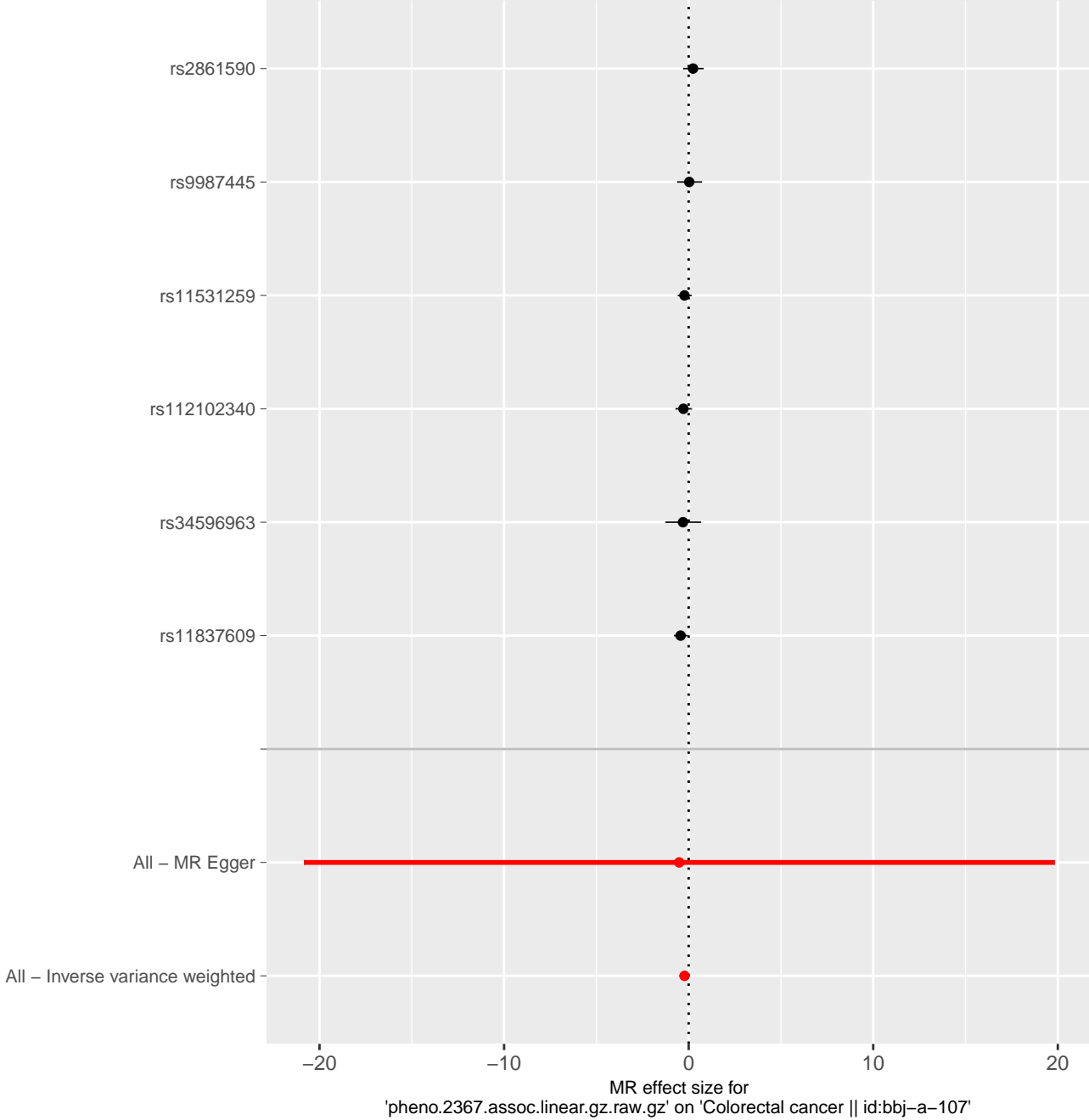

Supplement: Supplementary file 1 [file DataSheet1.ZIP › Supplementary Materials/MR plots for tongue/tongue═╝/Colorectal cancer/pheno.2367_to_colorectal cancer_forest.pdf]

# MR Method

- Inverse variance weighted
- MR Egger

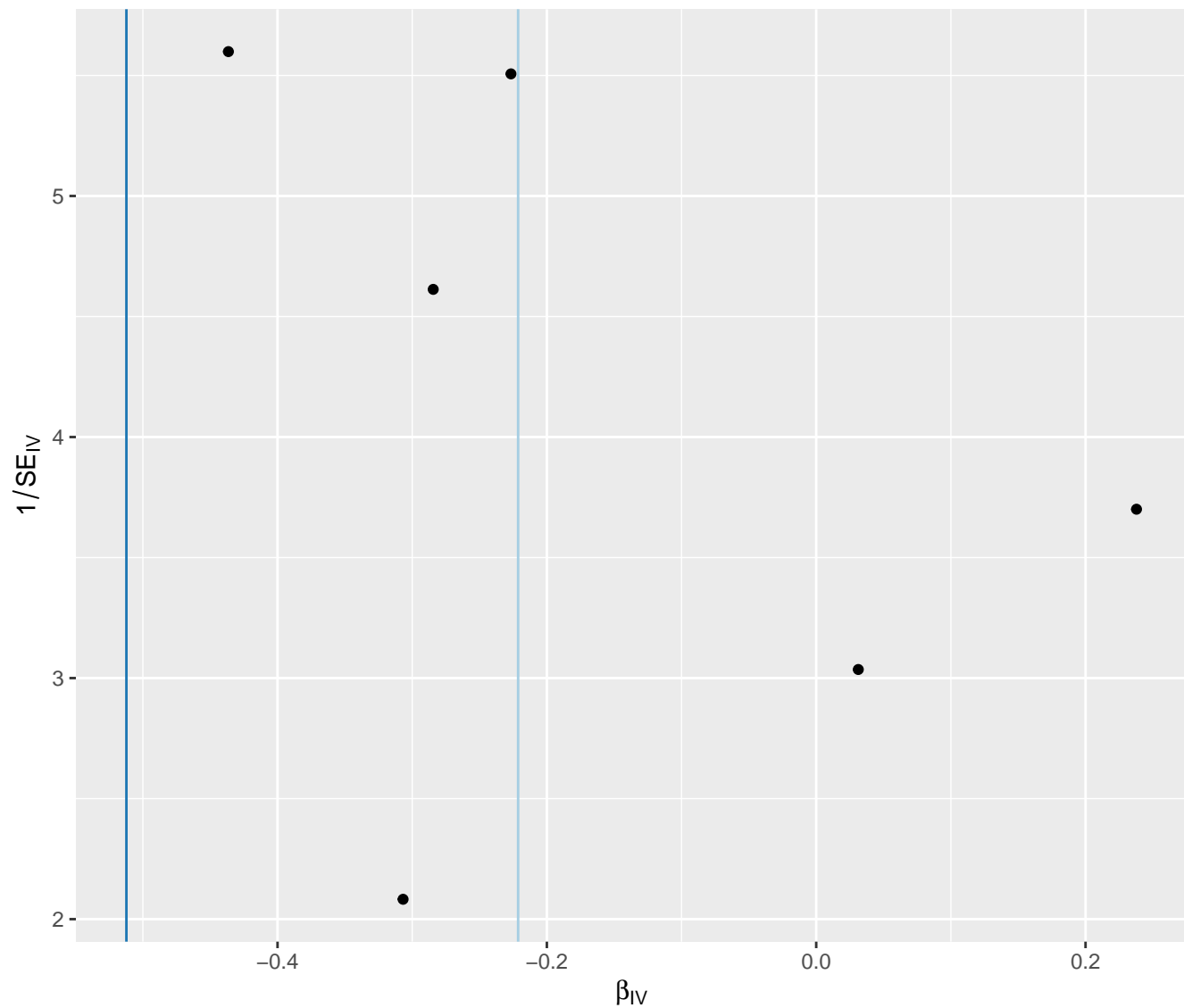

Supplement: Supplementary file 1 [file DataSheet1.ZIP › Supplementary Materials/MR plots for tongue/tongue═╝/Colorectal cancer/pheno.2367_to_colorectal cancer_funnel.pdf]

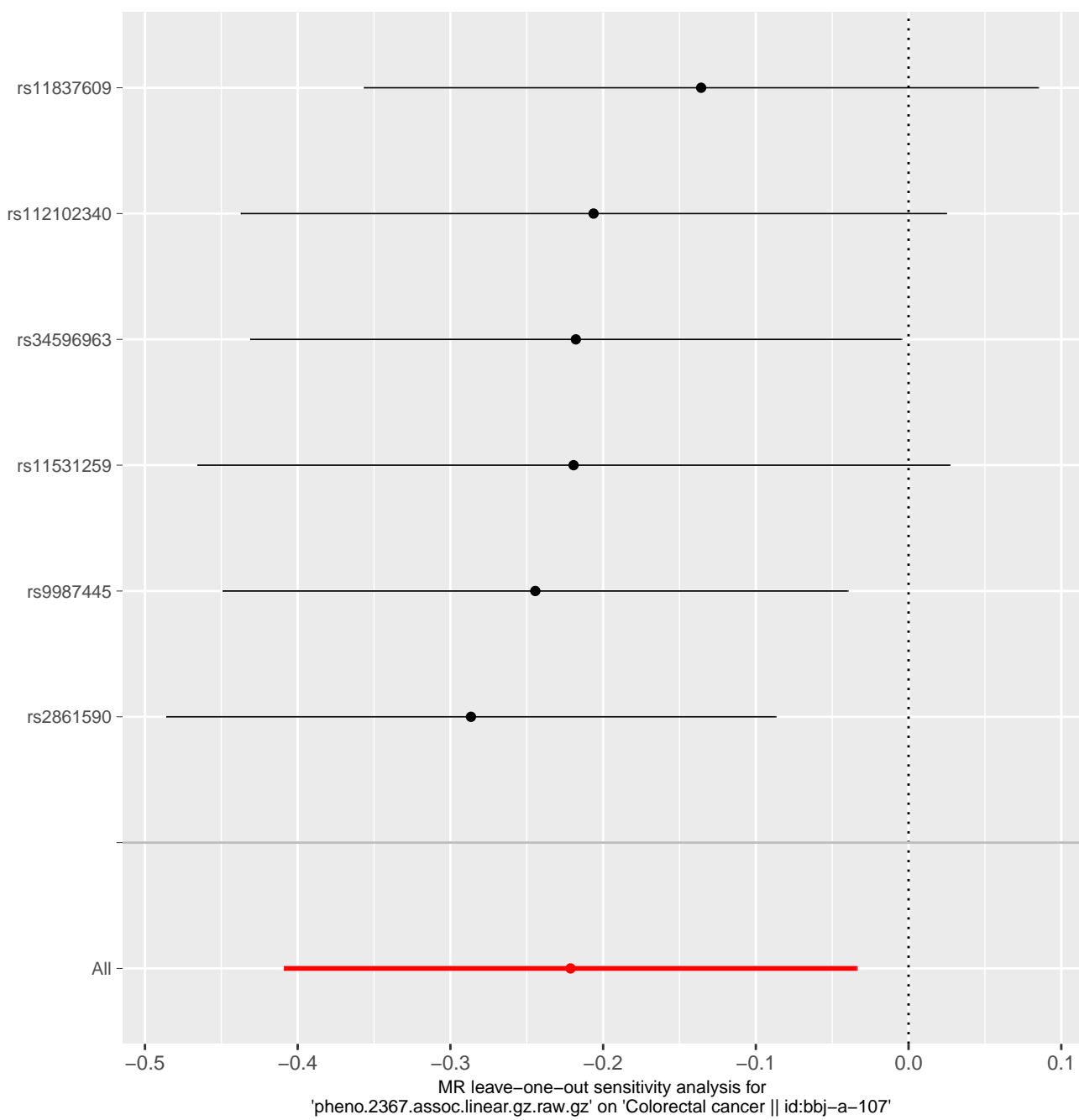

Supplement: Supplementary file 1 [file DataSheet1.ZIP › Supplementary Materials/MR plots for tongue/tongue═╝/Colorectal cancer/pheno.2367_to_colorectal cancer_leave_one_out.pdf]

# MR Test

- Inverse variance weighted
- MR Egger
- Simple mode
- Weighted median
- Weighted mode

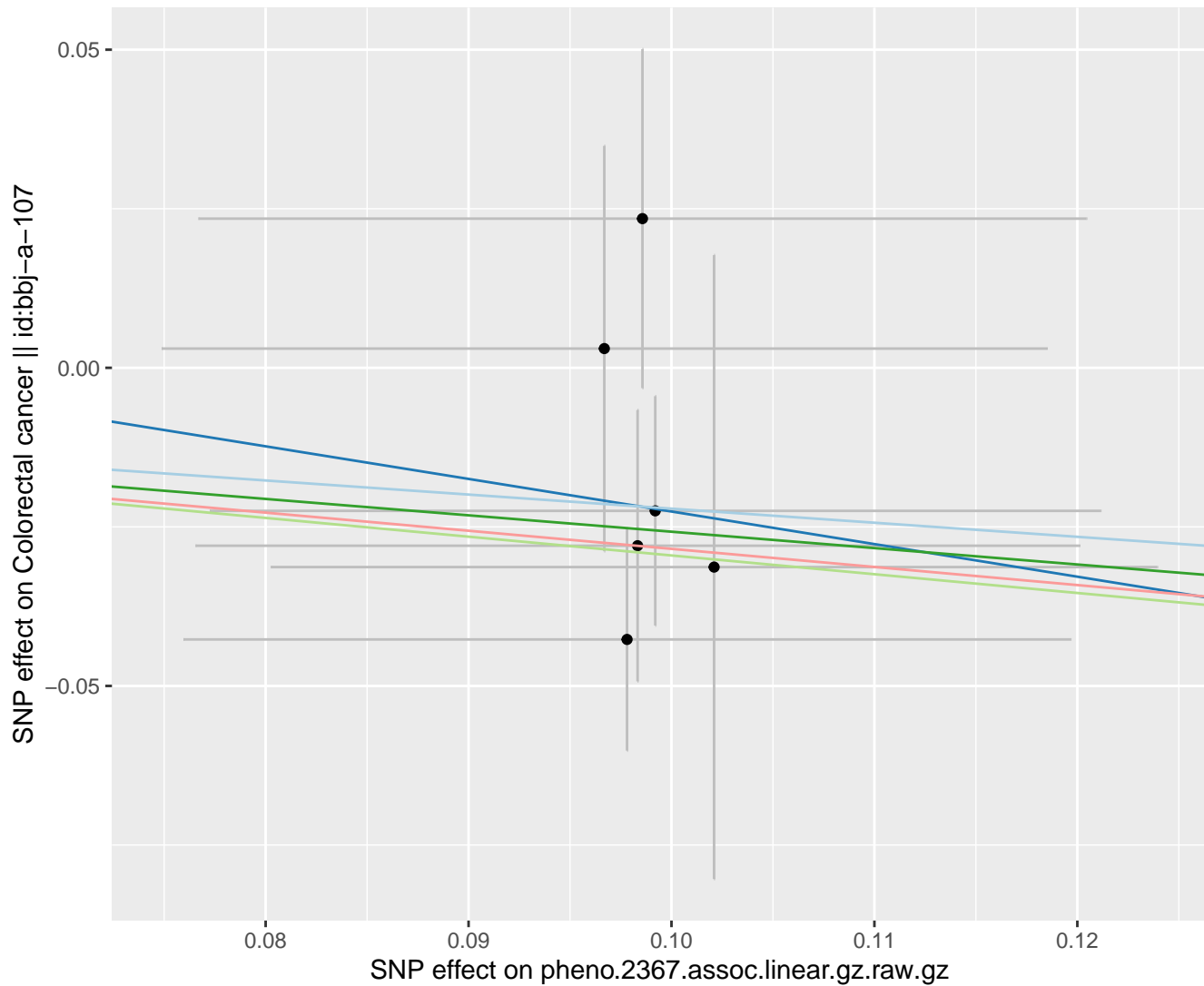

Supplement: Supplementary file 1 [file DataSheet1.ZIP › Supplementary Materials/MR plots for tongue/tongue═╝/Colorectal cancer/pheno.2367_to_colorectal cancer_scatter.pdf]

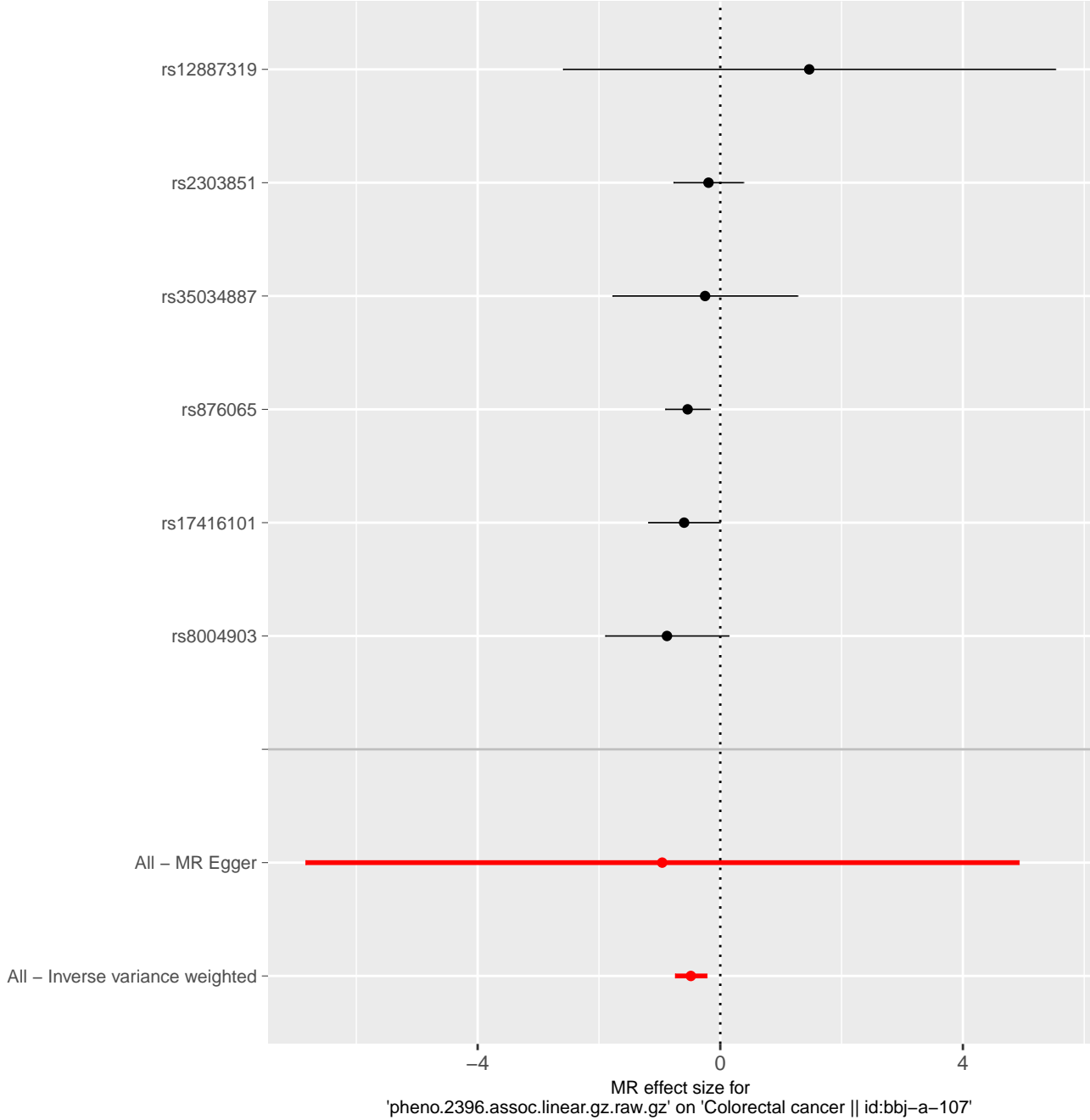

Supplement: Supplementary file 1 [file DataSheet1.ZIP › Supplementary Materials/MR plots for tongue/tongue═╝/Colorectal cancer/pheno.2396_to_colorectal cancer_forest.pdf]

# MR Method

- Inverse variance weighted
- MR Egger

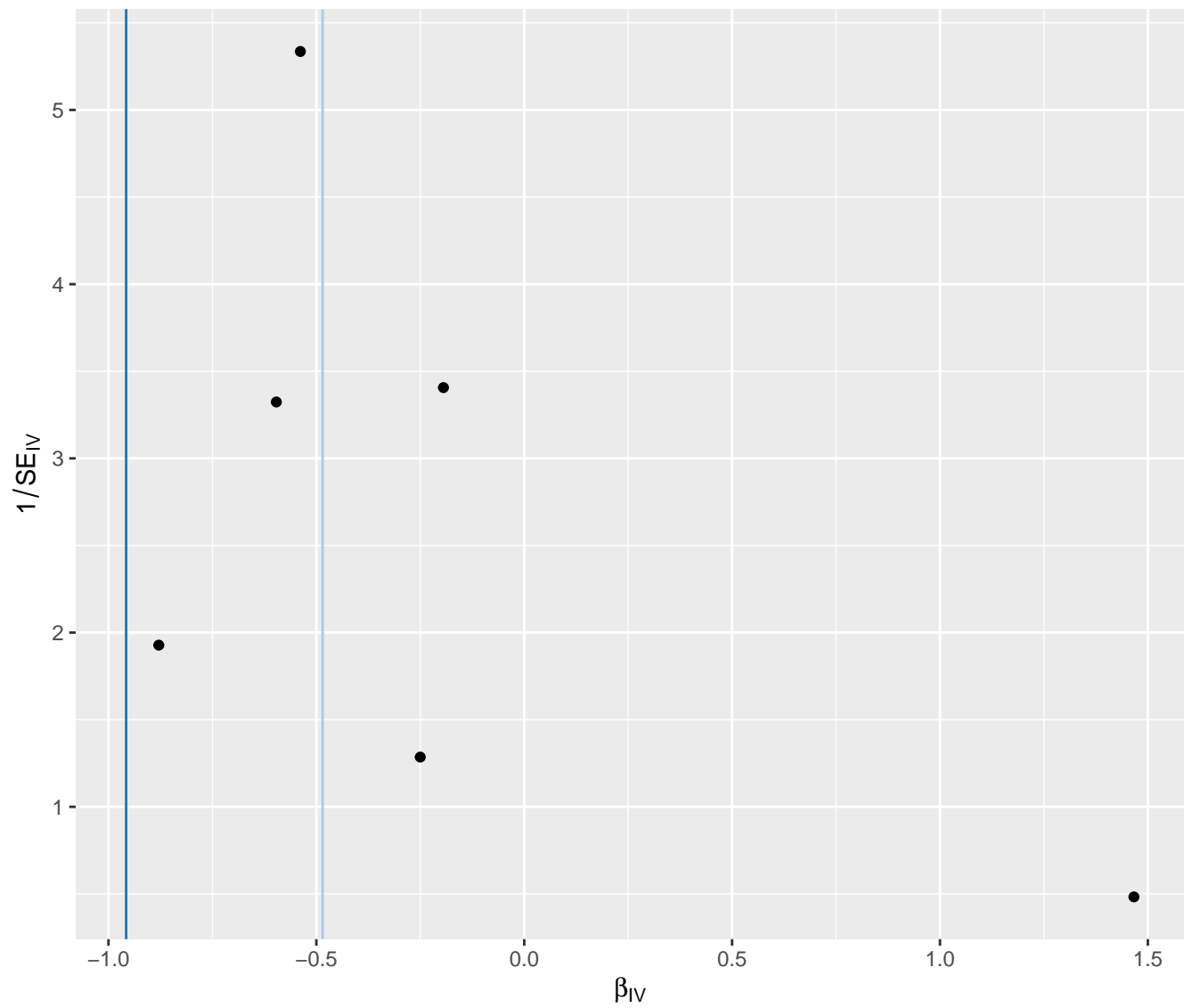

Supplement: Supplementary file 1 [file DataSheet1.ZIP › Supplementary Materials/MR plots for tongue/tongue═╝/Colorectal cancer/pheno.2396_to_colorectal cancer_funnel.pdf]

# MR Test

- Inverse variance weighted
- MR Egger
- Simple mode
- Weighted median
- Weighted mode

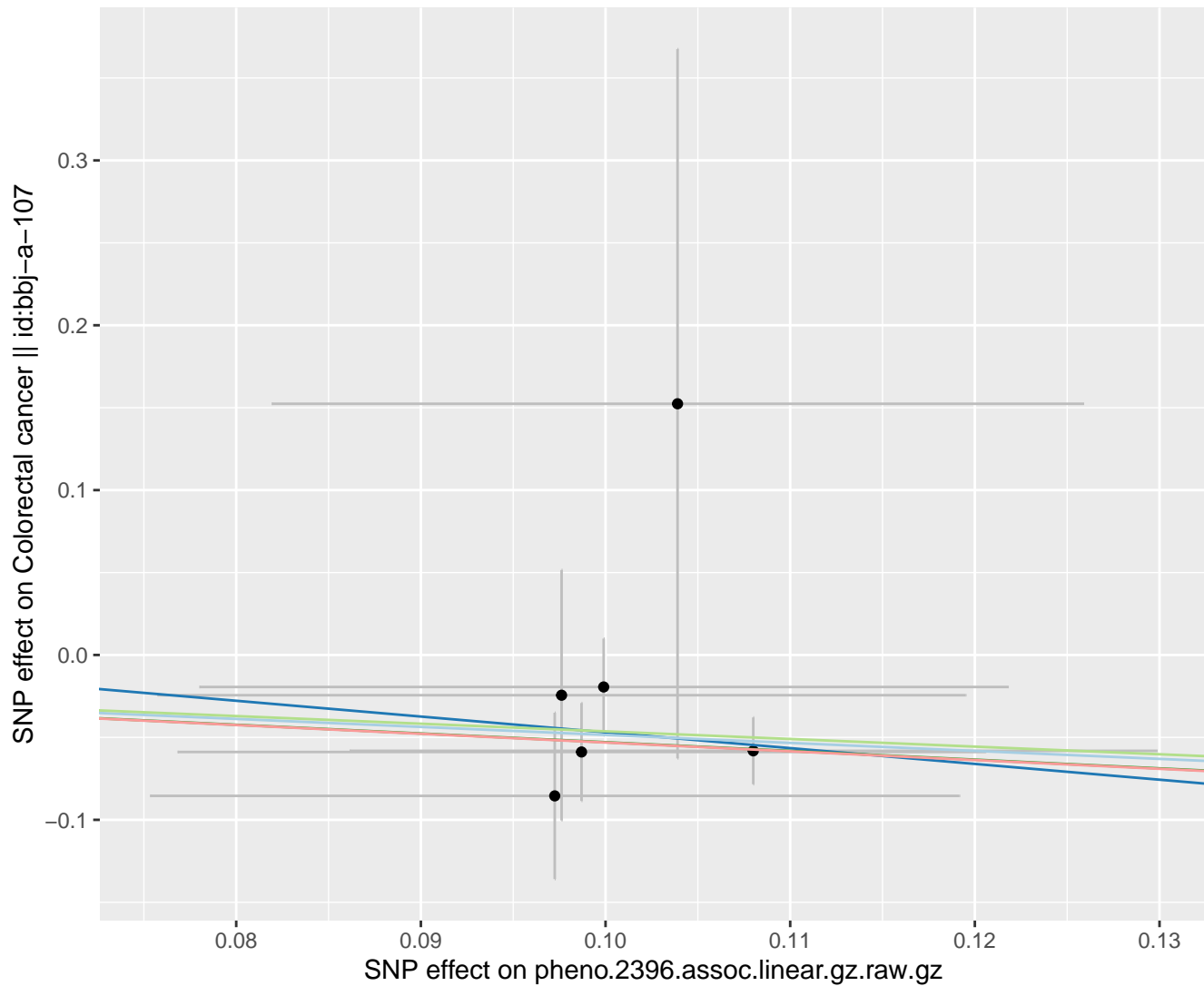

Supplement: Supplementary file 1 [file DataSheet1.ZIP › Supplementary Materials/MR plots for tongue/tongue═╝/Colorectal cancer/pheno.2396_to_colorectal cancer_scatter.pdf]

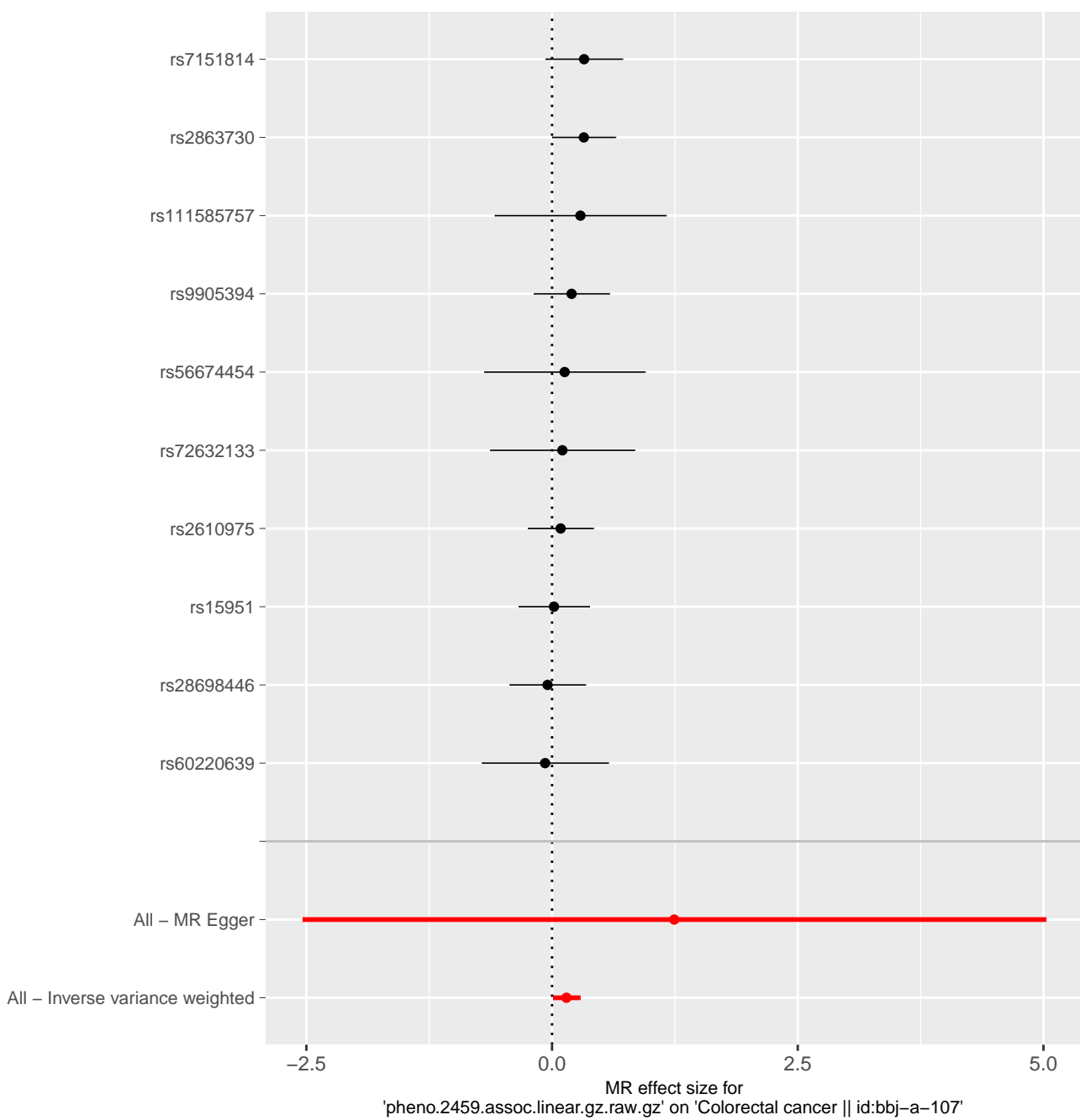

Supplement: Supplementary file 1 [file DataSheet1.ZIP › Supplementary Materials/MR plots for tongue/tongue═╝/Colorectal cancer/pheno.2459_to_colorectal cancer_forest.pdf]

# MR Method

- Inverse variance weighted
- MR Egger

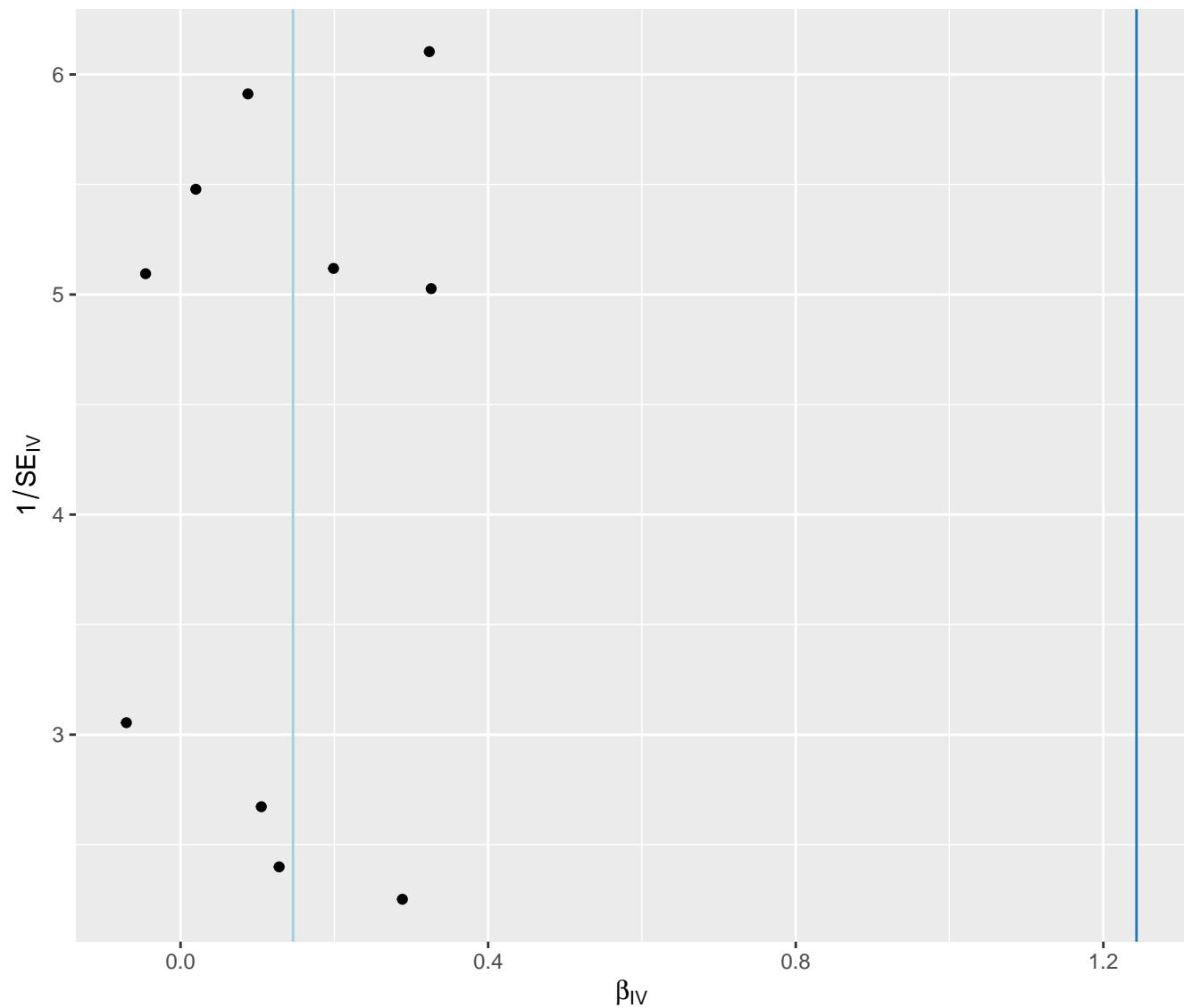

Supplement: Supplementary file 1 [file DataSheet1.ZIP › Supplementary Materials/MR plots for tongue/tongue═╝/Colorectal cancer/pheno.2459_to_colorectal cancer_funnel.pdf]

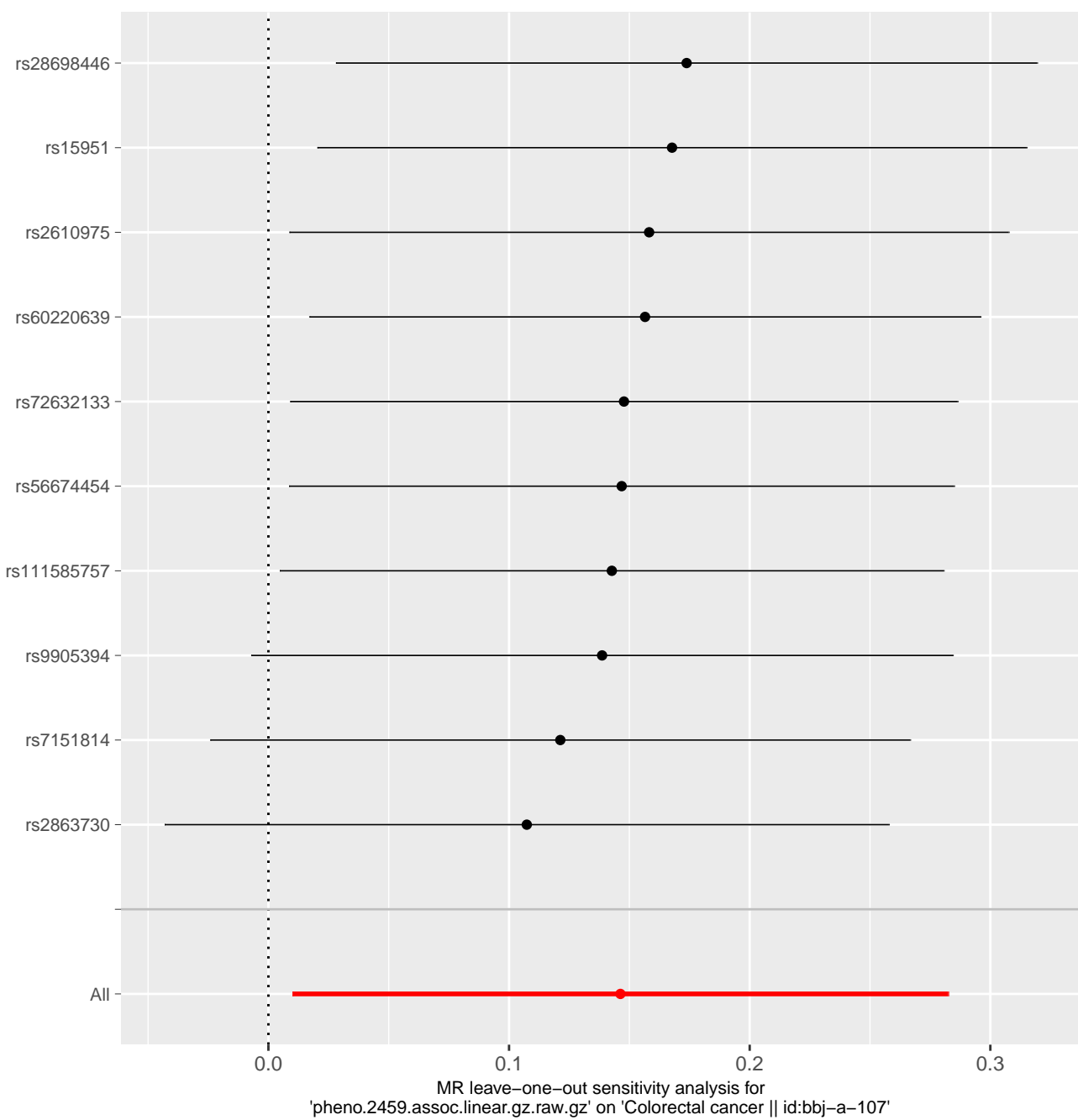

Supplement: Supplementary file 1 [file DataSheet1.ZIP › Supplementary Materials/MR plots for tongue/tongue═╝/Colorectal cancer/pheno.2459_to_colorectal cancer_leave_one_out.pdf]

# MR Test

- Inverse variance weighted
- MR Egger
- Simple mode
- Weighted median
- Weighted mode

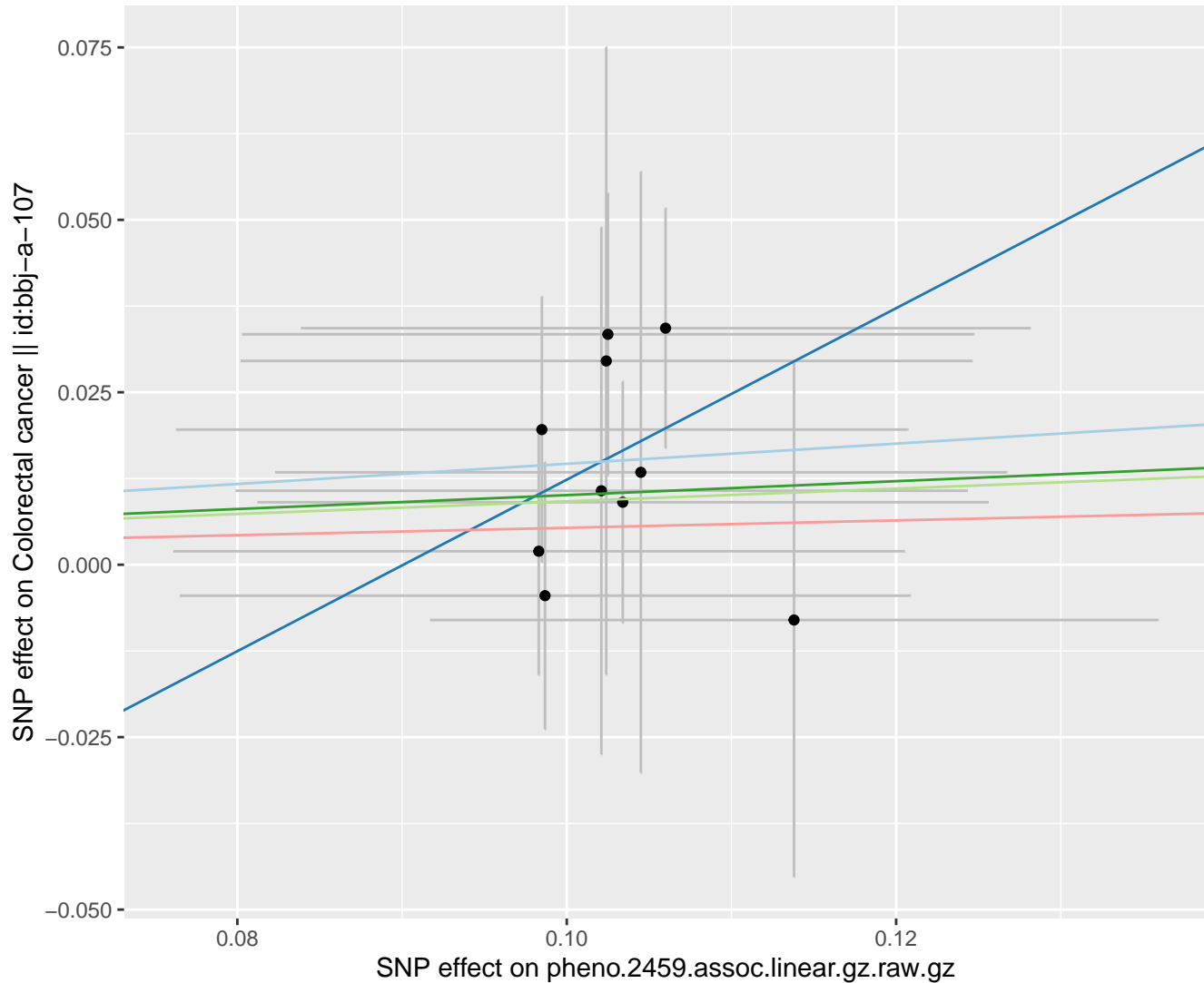

Supplement: Supplementary file 1 [file DataSheet1.ZIP › Supplementary Materials/MR plots for tongue/tongue═╝/Colorectal cancer/pheno.2459_to_colorectal cancer_scatter.pdf]
