# Supplementary figures and images for: Association between oral microbiome and seven types of cancers in East Asian population: a two-sample Mendelian randomization analysis (part 1 of 2)
Source: Front Mol Biosci. 2023 Nov 21;10:1327893. doi: 10.3389/fmolb.2023.1327893 (PMC10702768; doi:10.3389/fmolb.2023.1327893)

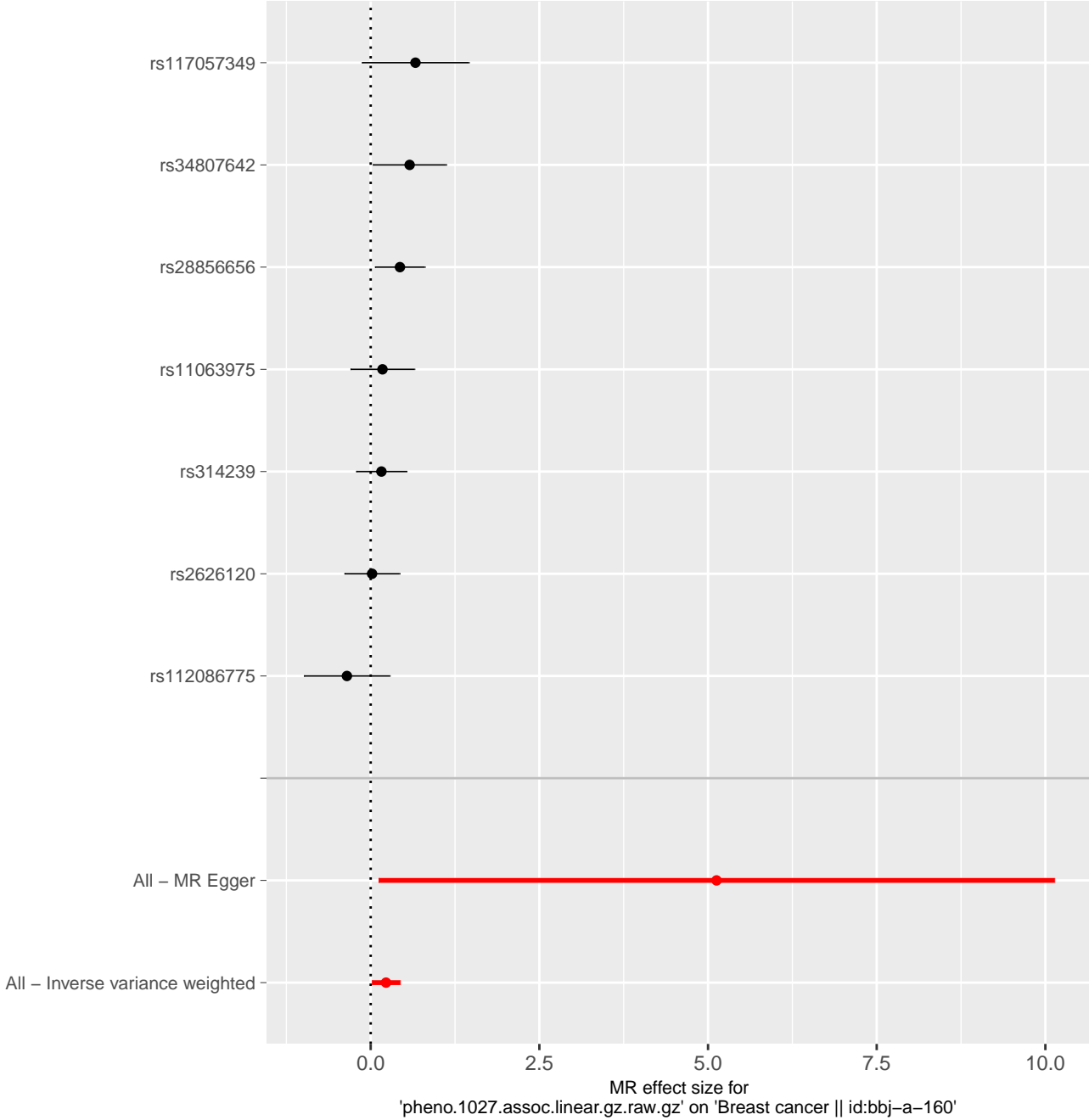

Supplement: Supplementary file 1 [file DataSheet1.ZIP › Supplementary Materials/MR plots for tongue/tongue═╝/Breast cancer/pheno.1027_to_breast cancer_forest.pdf]

# MR Method

- Inverse variance weighted
- MR Egger

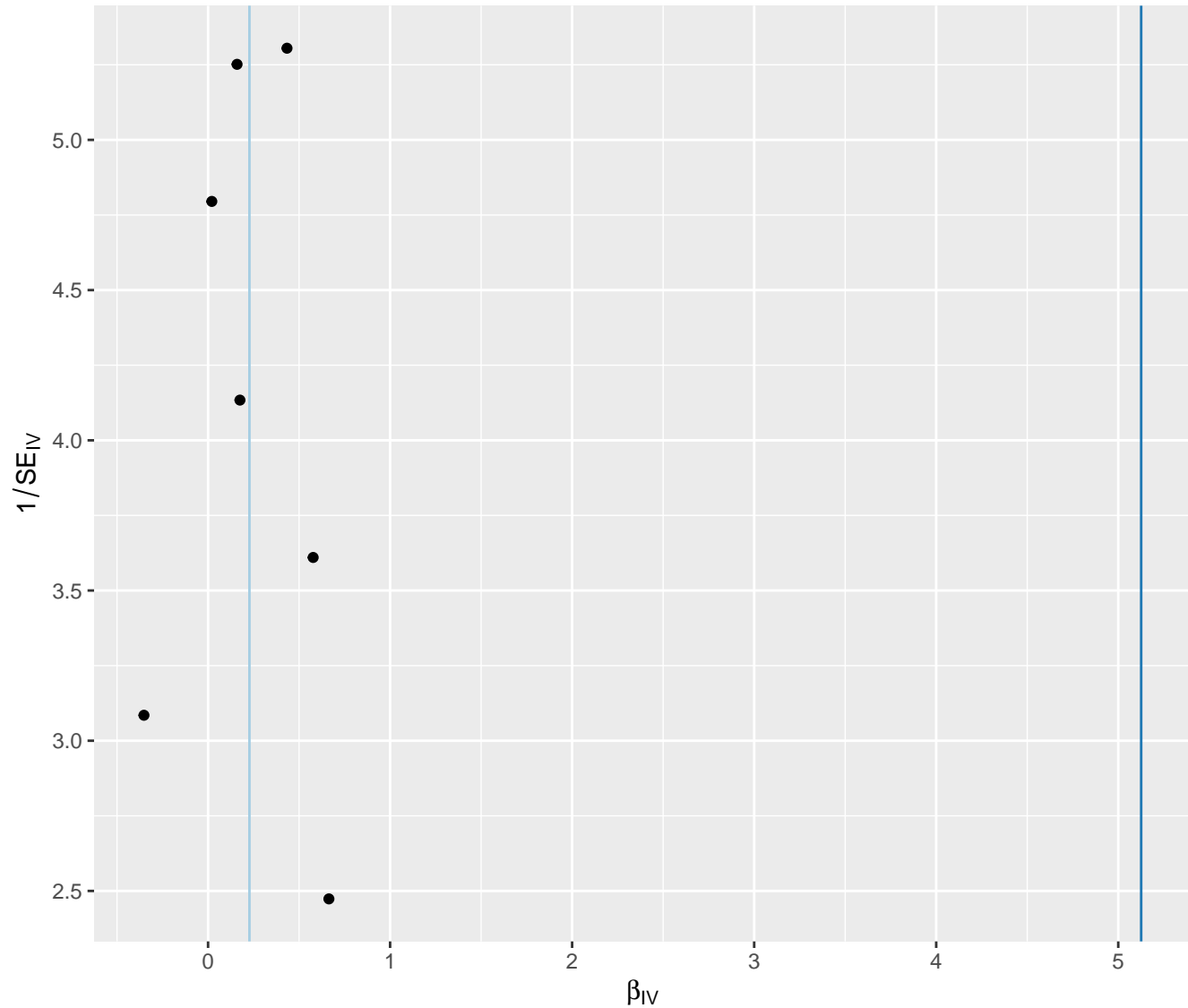

Supplement: Supplementary file 1 [file DataSheet1.ZIP › Supplementary Materials/MR plots for tongue/tongue═╝/Breast cancer/pheno.1027_to_breast cancer_funnel.pdf]

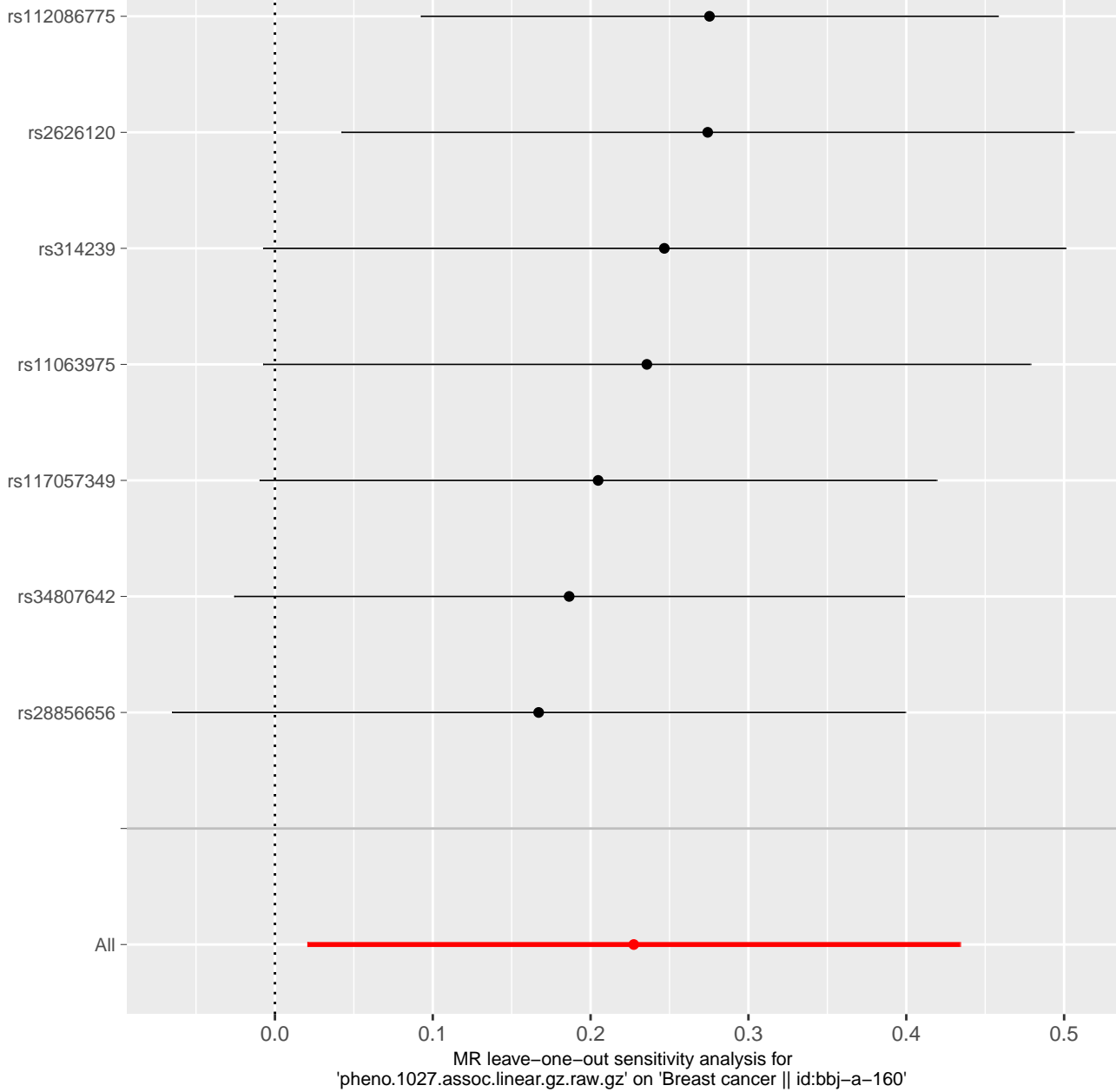

Supplement: Supplementary file 1 [file DataSheet1.ZIP › Supplementary Materials/MR plots for tongue/tongue═╝/Breast cancer/pheno.1027_to_breast cancer_leave_one_out.pdf]

# MR Test

- Inverse variance weighted
- MR Egger
- Simple mode
- Weighted median
- Weighted mode

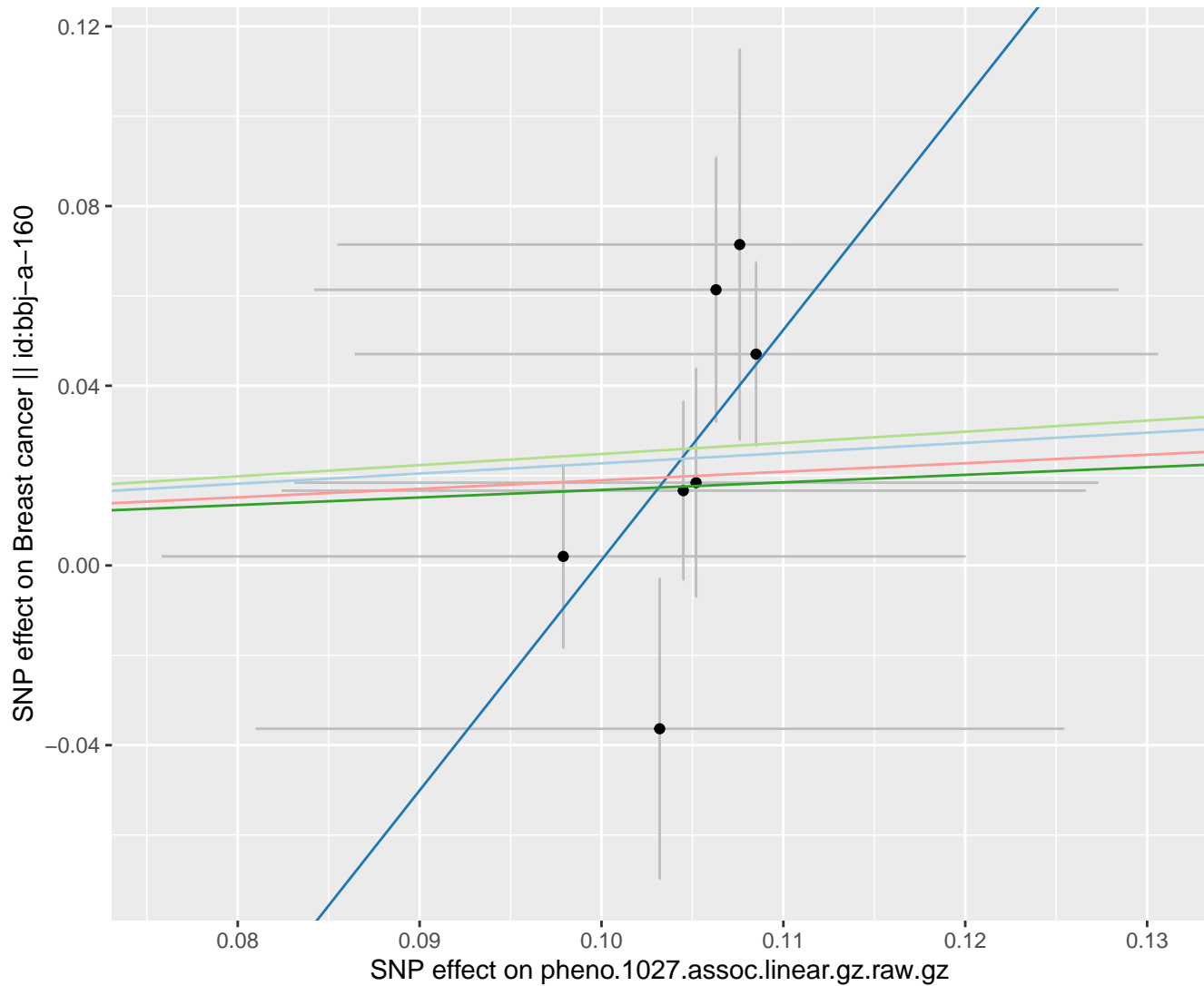

Supplement: Supplementary file 1 [file DataSheet1.ZIP › Supplementary Materials/MR plots for tongue/tongue═╝/Breast cancer/pheno.1027_to_breast cancer_scatter.pdf]

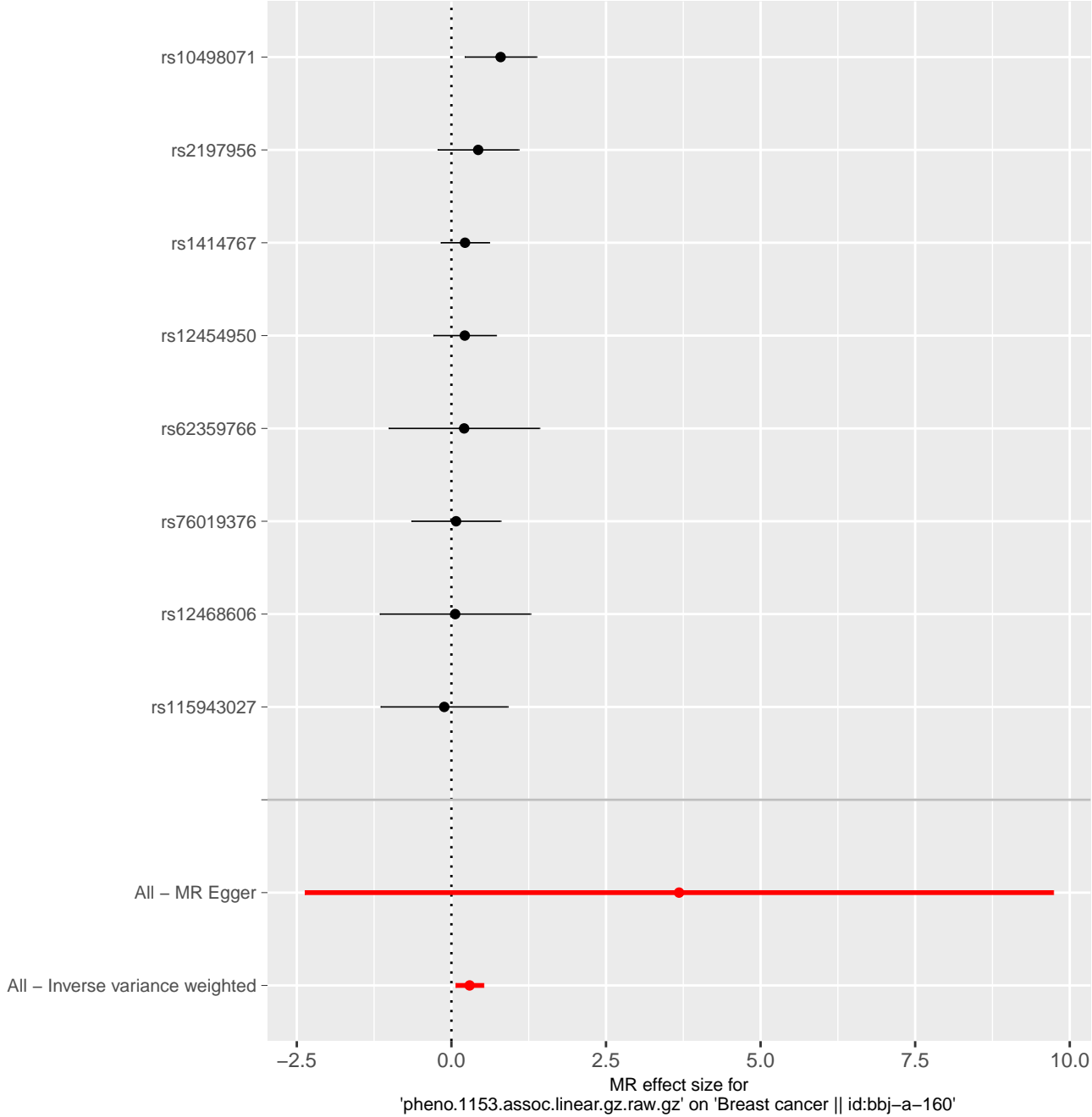

Supplement: Supplementary file 1 [file DataSheet1.ZIP › Supplementary Materials/MR plots for tongue/tongue═╝/Breast cancer/pheno.1153_to_breast cancer_forest.pdf]

# MR Method

- Inverse variance weighted
- MR Egger

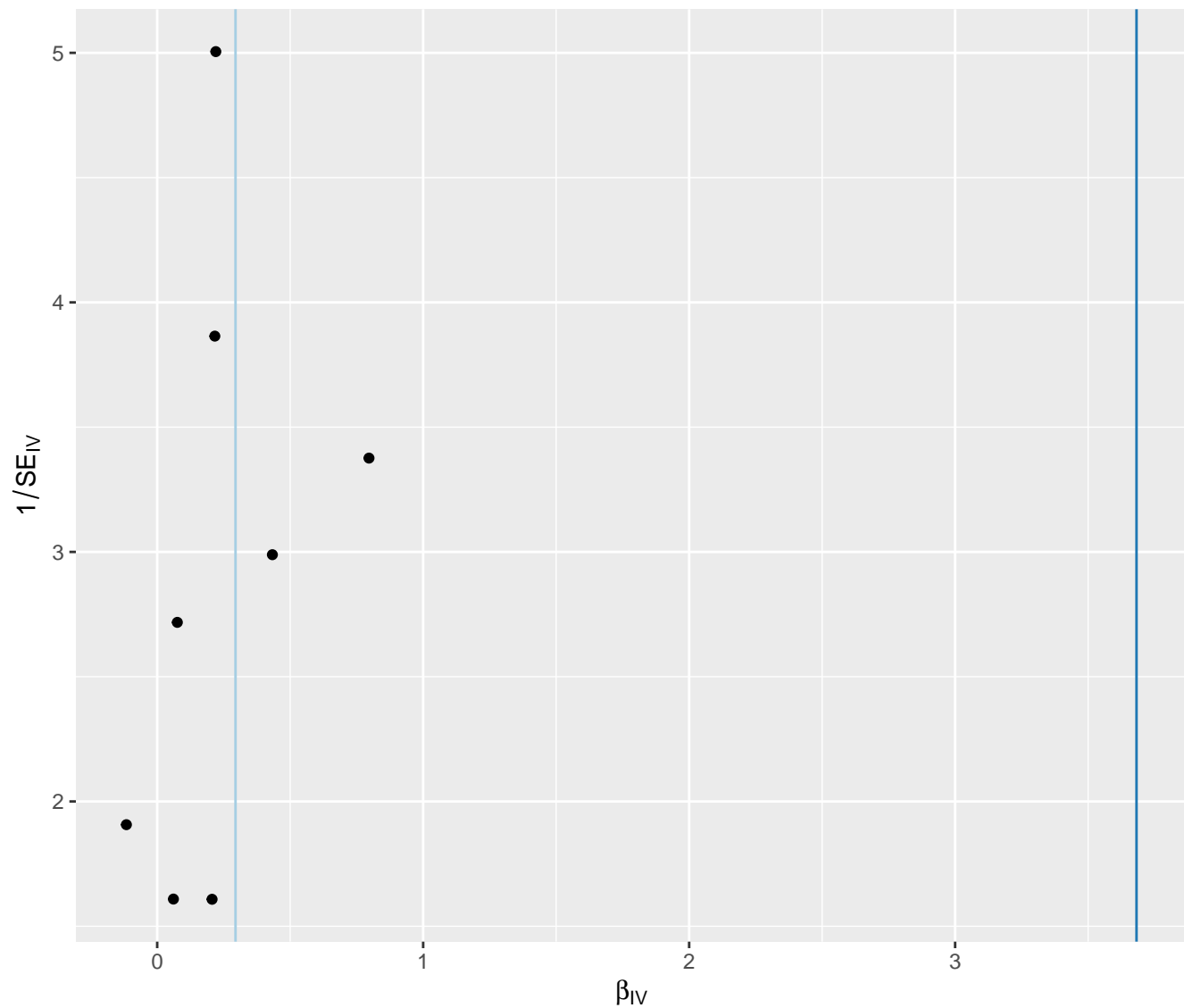

Supplement: Supplementary file 1 [file DataSheet1.ZIP › Supplementary Materials/MR plots for tongue/tongue═╝/Breast cancer/pheno.1153_to_breast cancer_funnel.pdf]

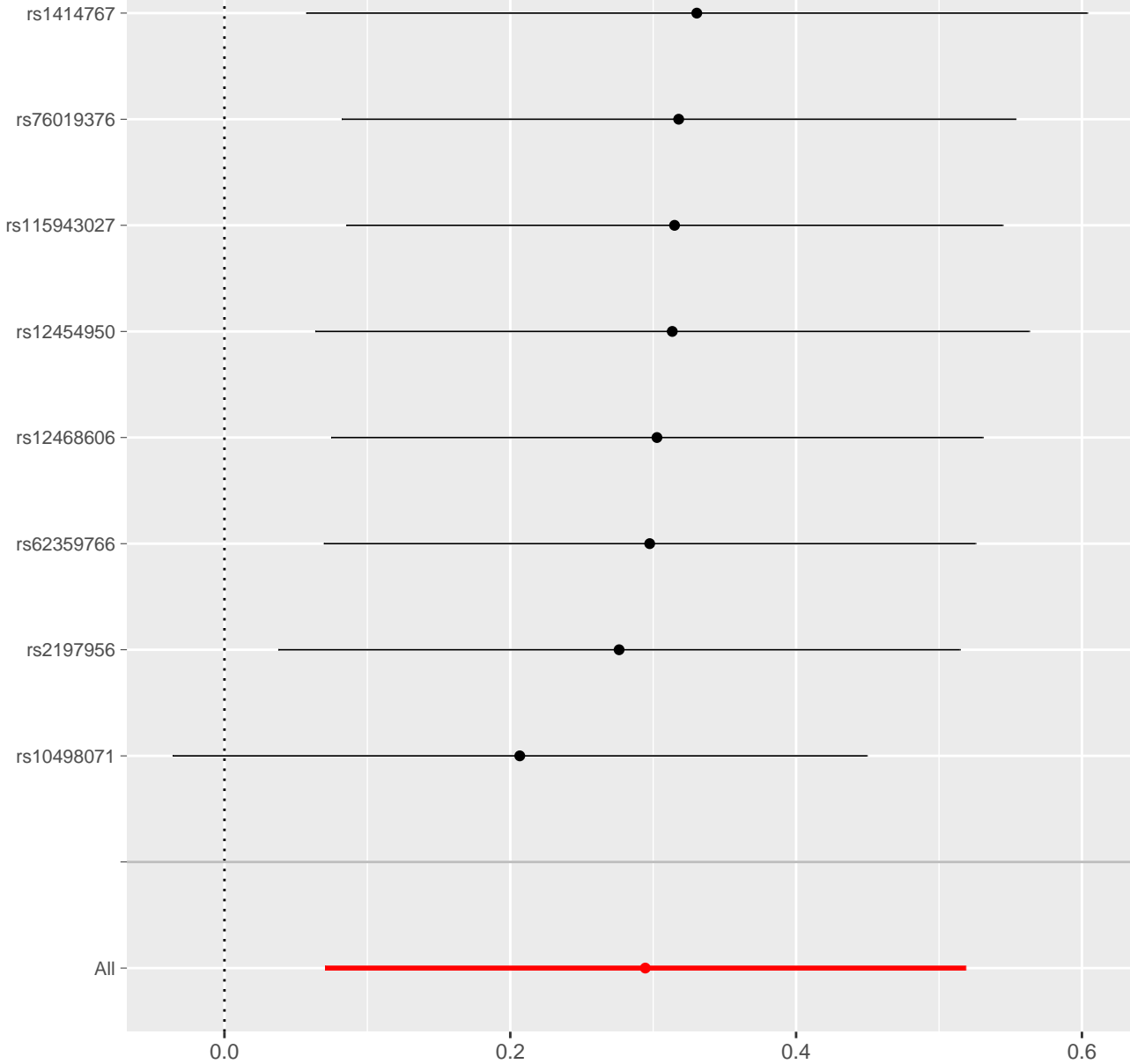

MR leave-one-out sensitivity analysis for  
'pheno.1153.assoc.linear.gz.raw.gz' on 'Breast cancer || id:bbj-a-160'

Supplement: Supplementary file 1 [file DataSheet1.ZIP › Supplementary Materials/MR plots for tongue/tongue═╝/Breast cancer/pheno.1153_to_breast cancer_leave_one_out.pdf]

# MR Test

- Inverse variance weighted
- MR Egger
- Simple mode
- Weighted median
- Weighted mode

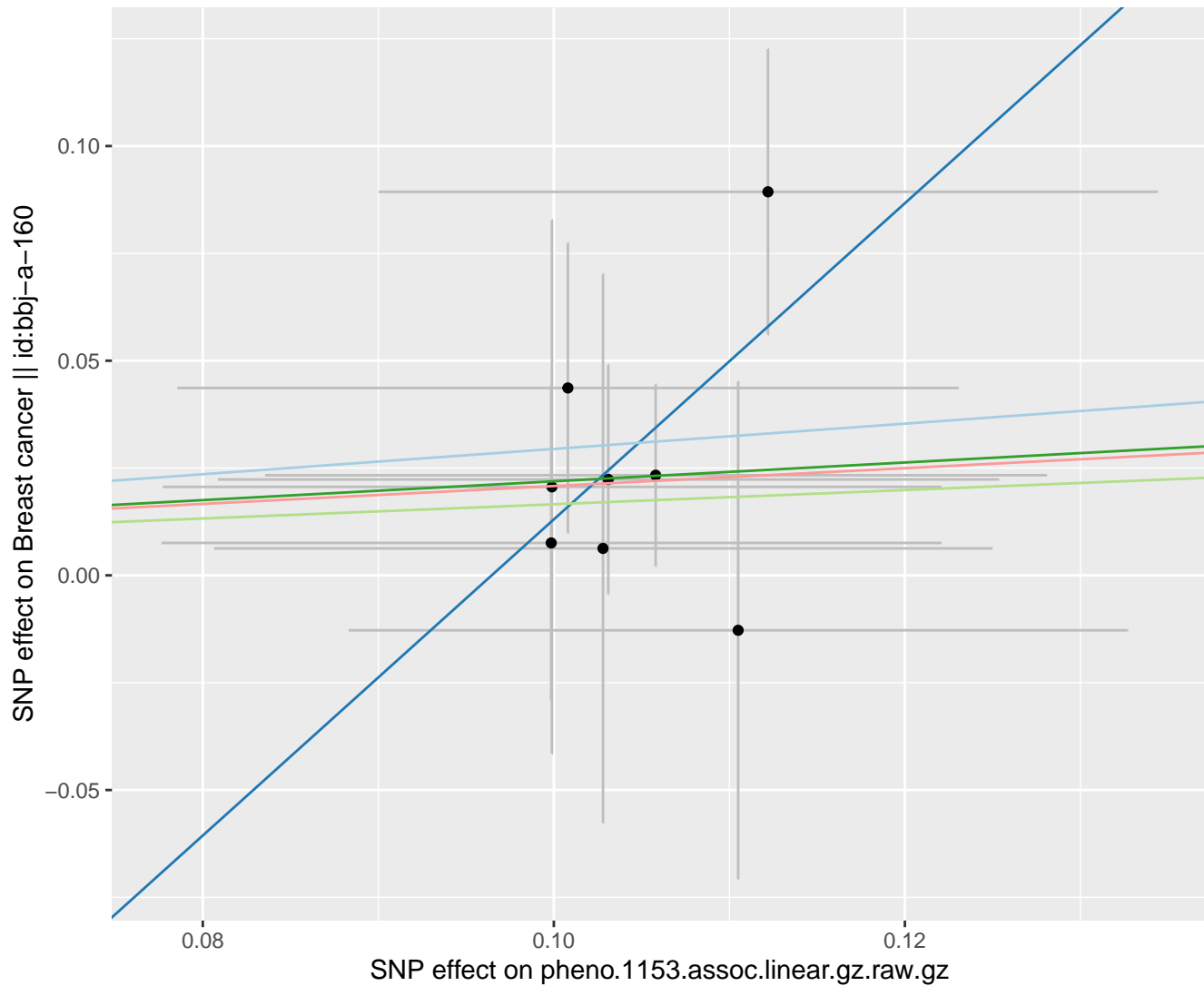

Supplement: Supplementary file 1 [file DataSheet1.ZIP › Supplementary Materials/MR plots for tongue/tongue═╝/Breast cancer/pheno.1153_to_breast cancer_scatter.pdf]

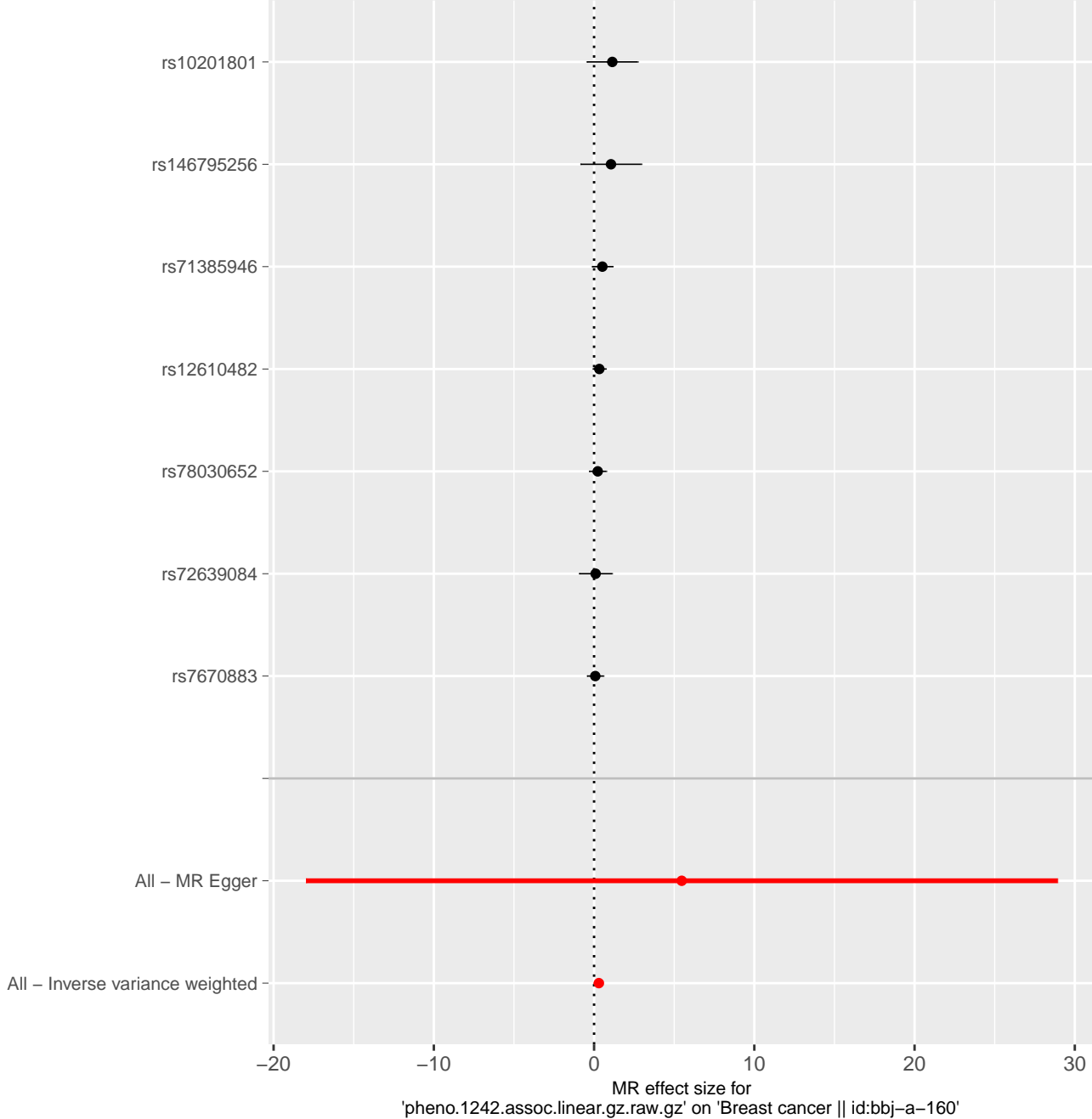

Supplement: Supplementary file 1 [file DataSheet1.ZIP › Supplementary Materials/MR plots for tongue/tongue═╝/Breast cancer/pheno.1242_to_breast cancer_forest.pdf]

# MR Method

- Inverse variance weighted
- MR Egger

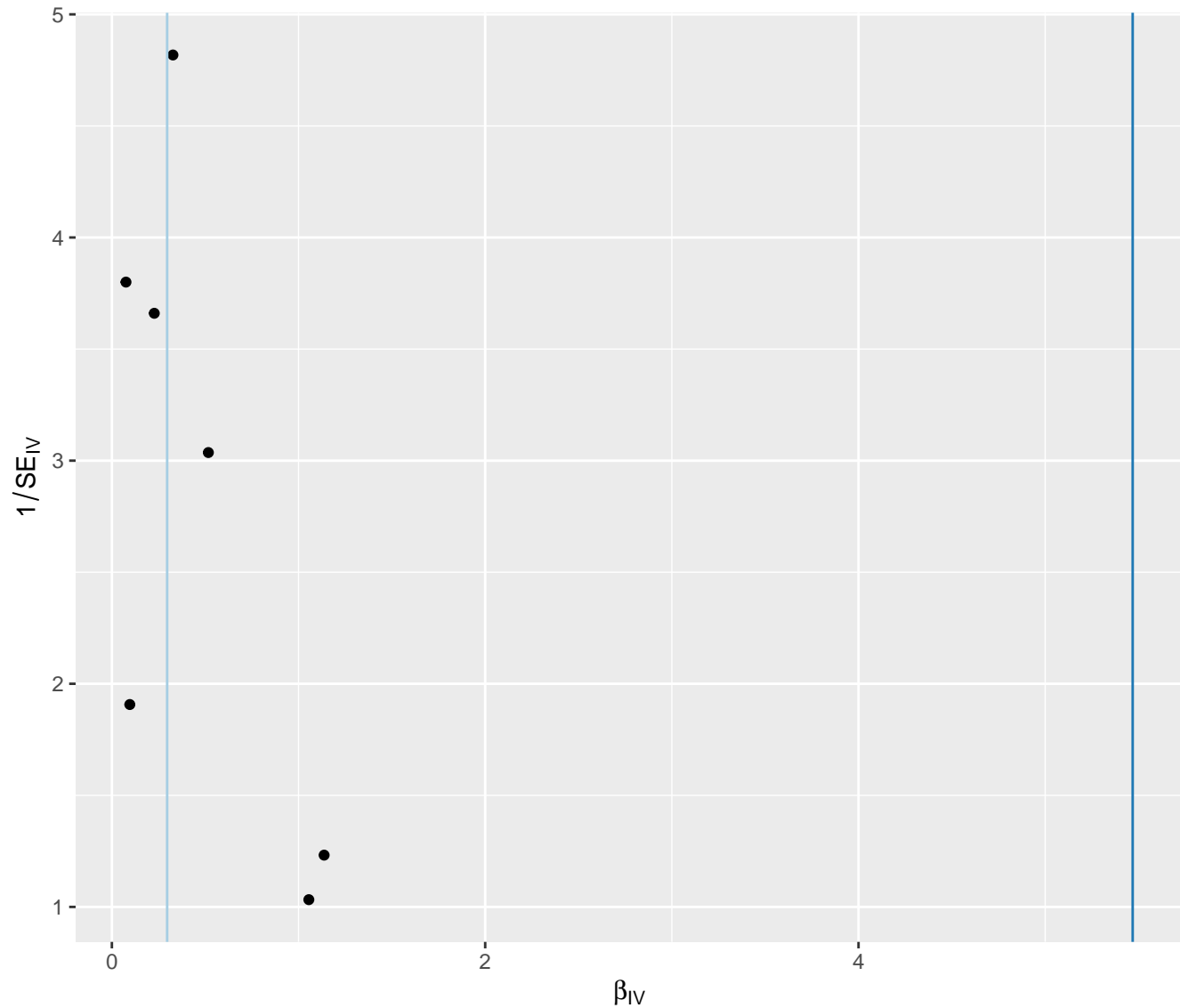

Supplement: Supplementary file 1 [file DataSheet1.ZIP › Supplementary Materials/MR plots for tongue/tongue═╝/Breast cancer/pheno.1242_to_breast cancer_funnel.pdf]

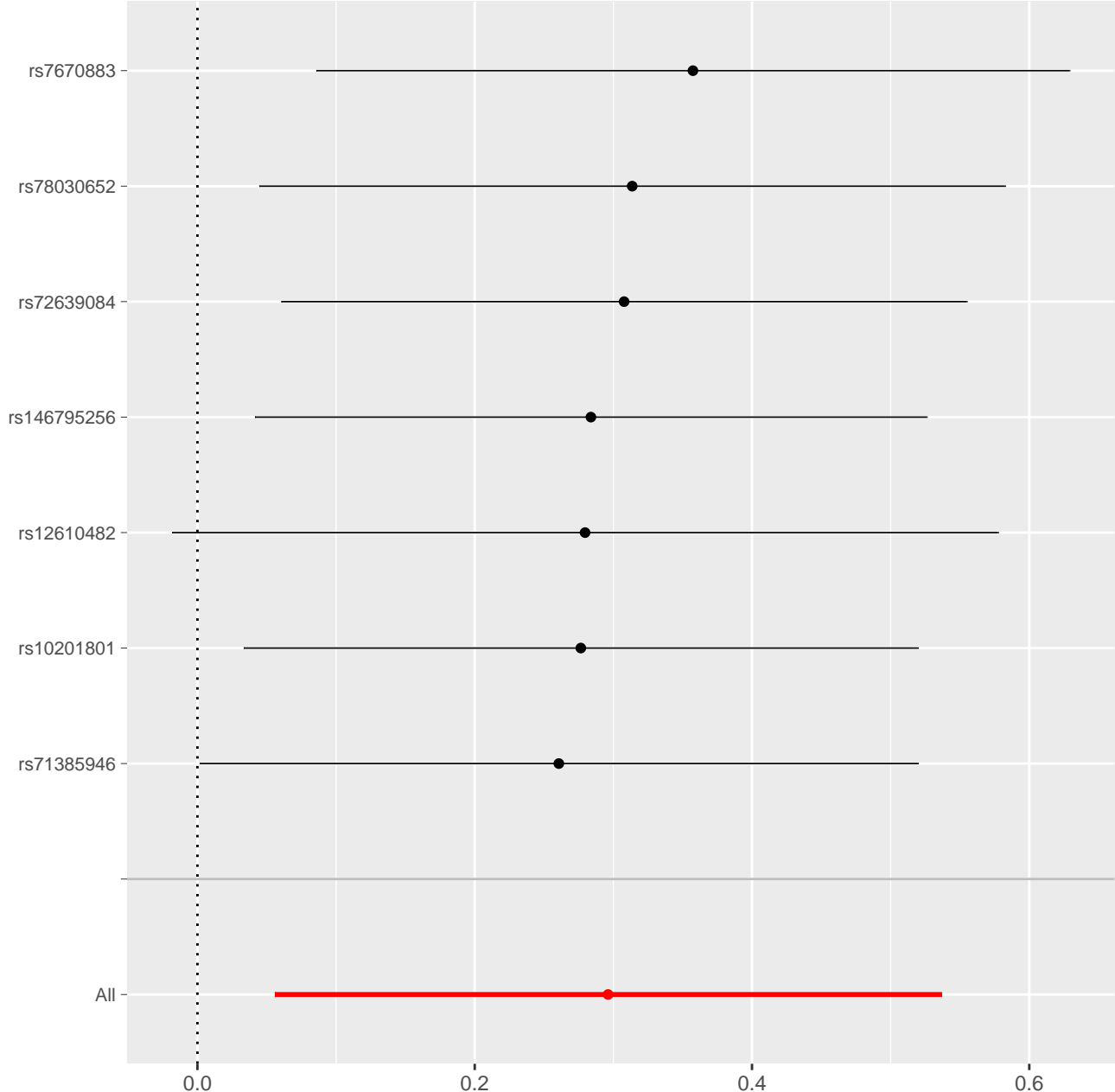

MR leave-one-out sensitivity analysis for  
'pheno.1242.assoc.linear.gz.raw.gz' on 'Breast cancer || id:bbj-a-160'

Supplement: Supplementary file 1 [file DataSheet1.ZIP › Supplementary Materials/MR plots for tongue/tongue═╝/Breast cancer/pheno.1242_to_breast cancer_leave_one_out.pdf]

# MR Test

- Inverse variance weighted
- MR Egger
- Simple mode
- Weighted median
- Weighted mode

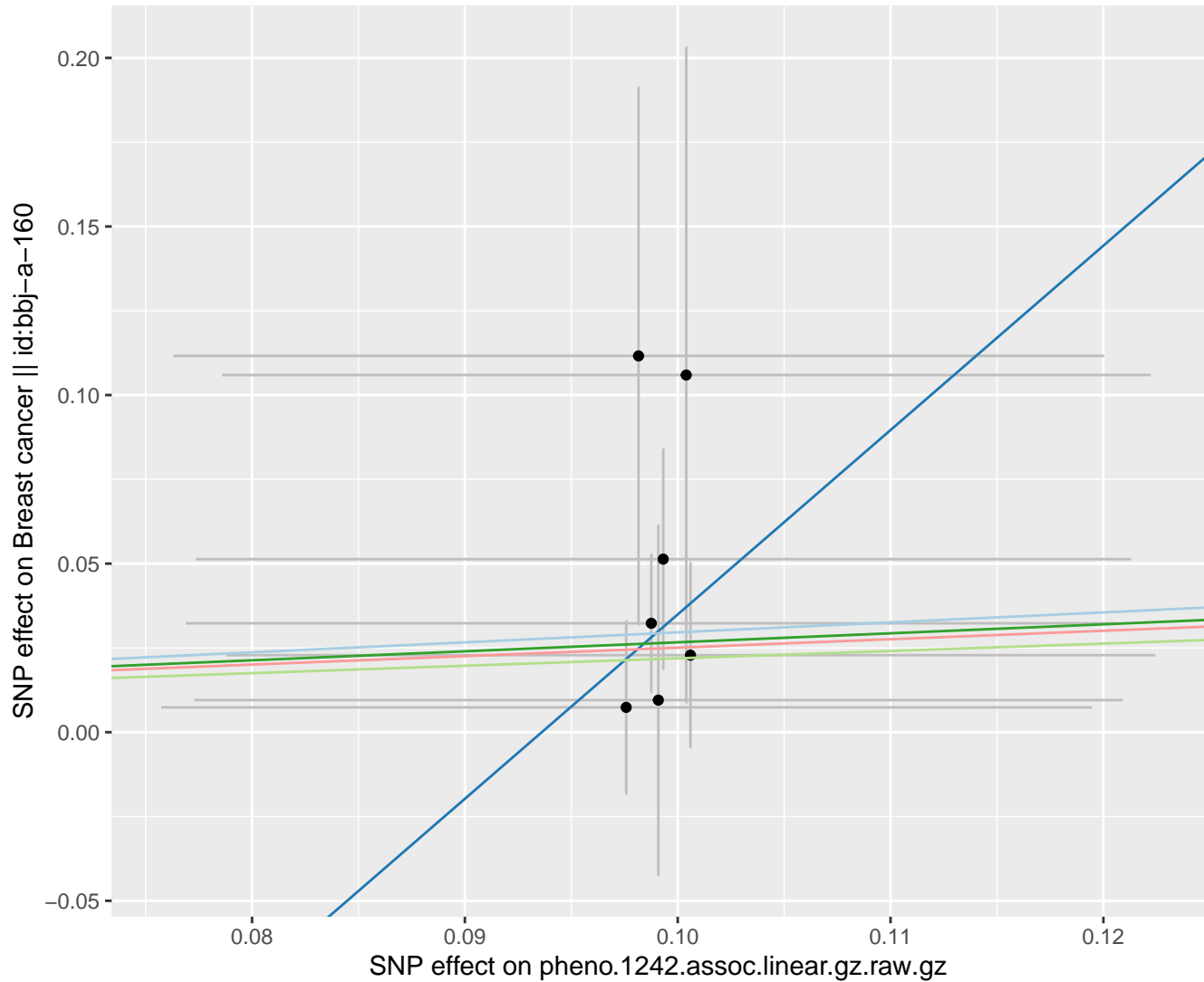

Supplement: Supplementary file 1 [file DataSheet1.ZIP › Supplementary Materials/MR plots for tongue/tongue═╝/Breast cancer/pheno.1242_to_breast cancer_scatter.pdf]

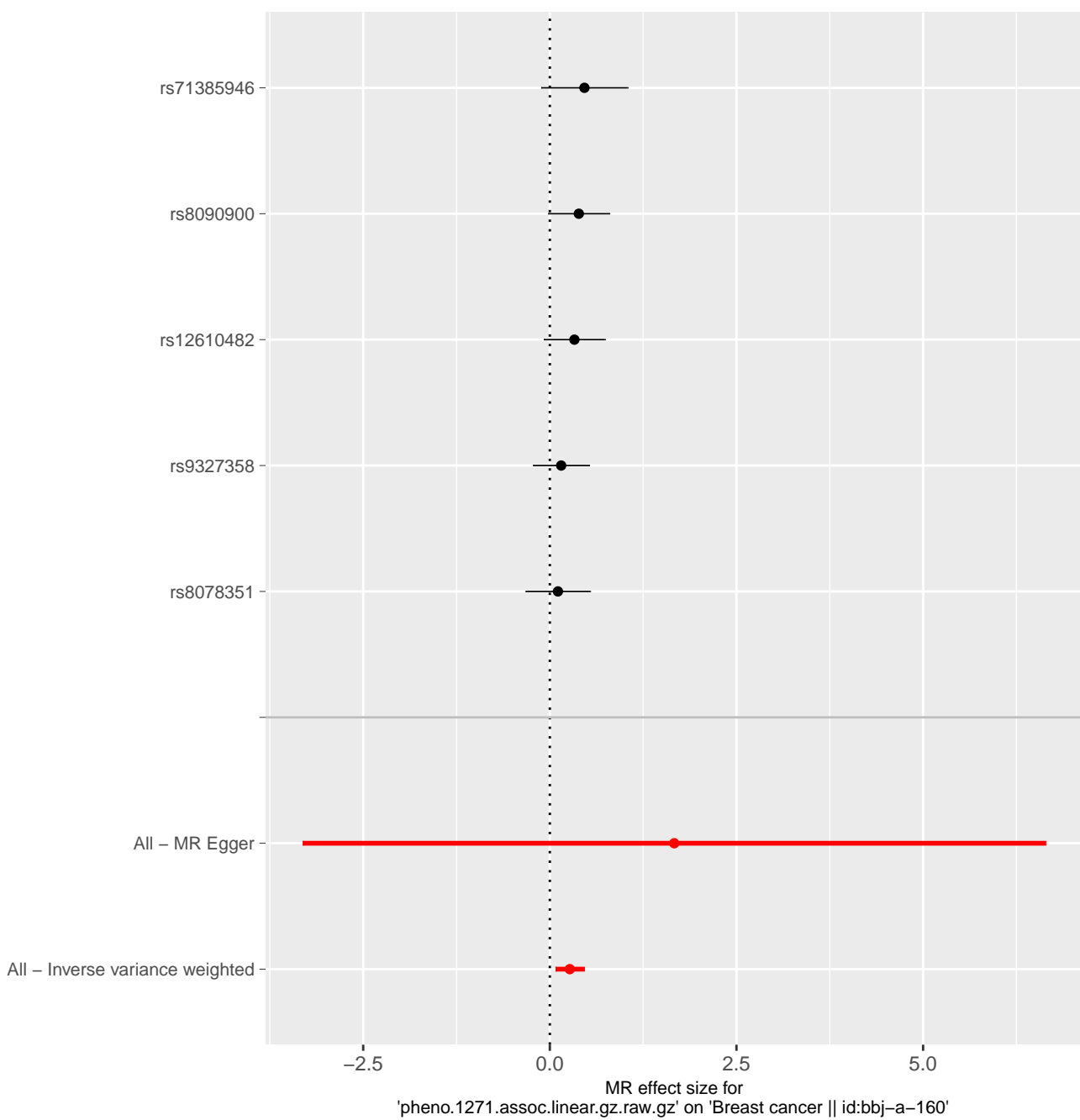

Supplement: Supplementary file 1 [file DataSheet1.ZIP › Supplementary Materials/MR plots for tongue/tongue═╝/Breast cancer/pheno.1271_to_breast cancer_forest.pdf]

# MR Method

Inverse variance weighted  
MR Egger

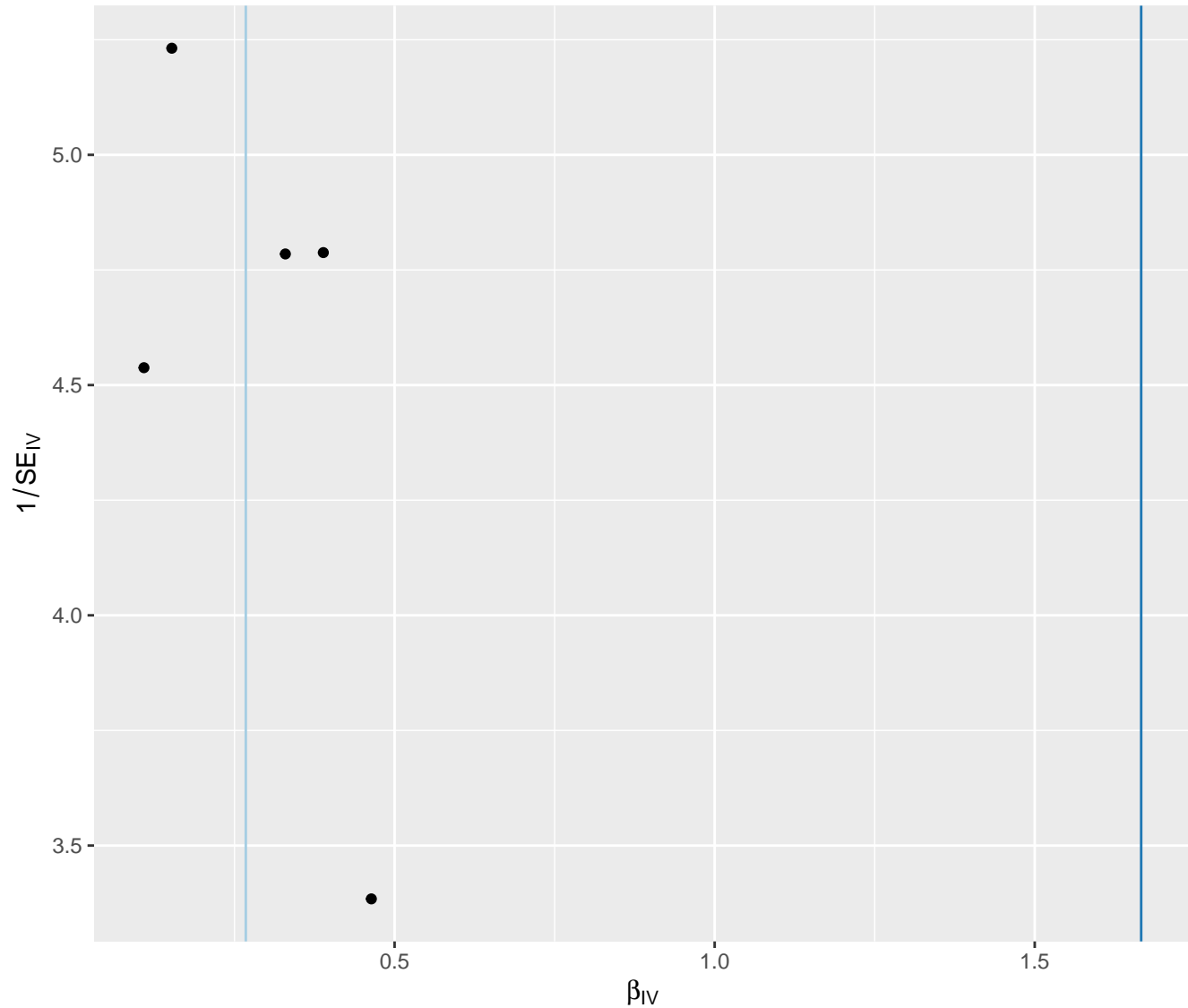

Supplement: Supplementary file 1 [file DataSheet1.ZIP › Supplementary Materials/MR plots for tongue/tongue═╝/Breast cancer/pheno.1271_to_breast cancer_funnel.pdf]

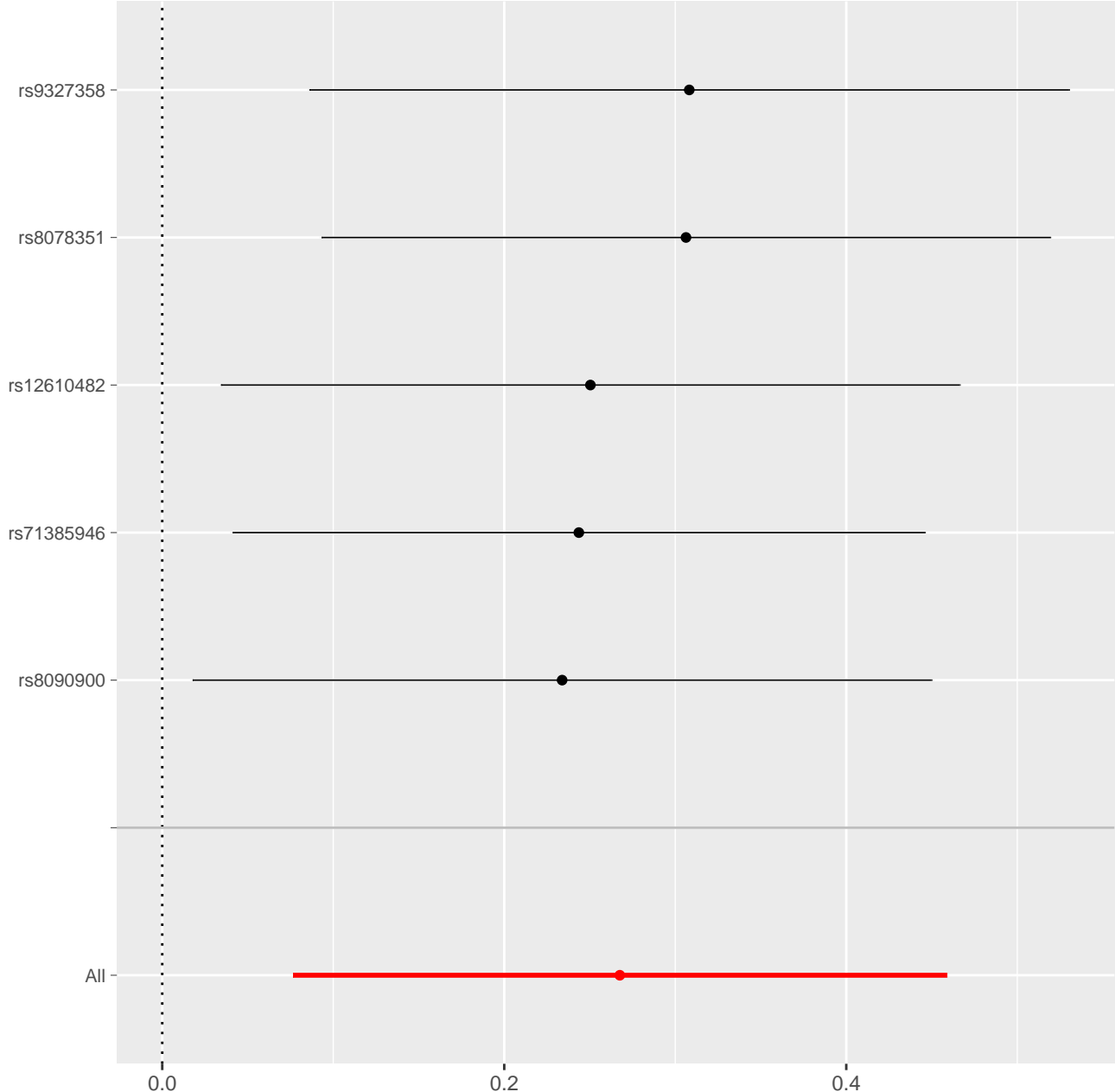

MR leave-one-out sensitivity analysis for  
'pheno.1271.assoc.linear.gz.raw.gz' on 'Breast cancer || id:bbj-a-160'

Supplement: Supplementary file 1 [file DataSheet1.ZIP › Supplementary Materials/MR plots for tongue/tongue═╝/Breast cancer/pheno.1271_to_breast cancer_leave_one_out.pdf]

# MR Test

- Inverse variance weighted
- MR Egger
- Simple mode
- Weighted median
- Weighted mode

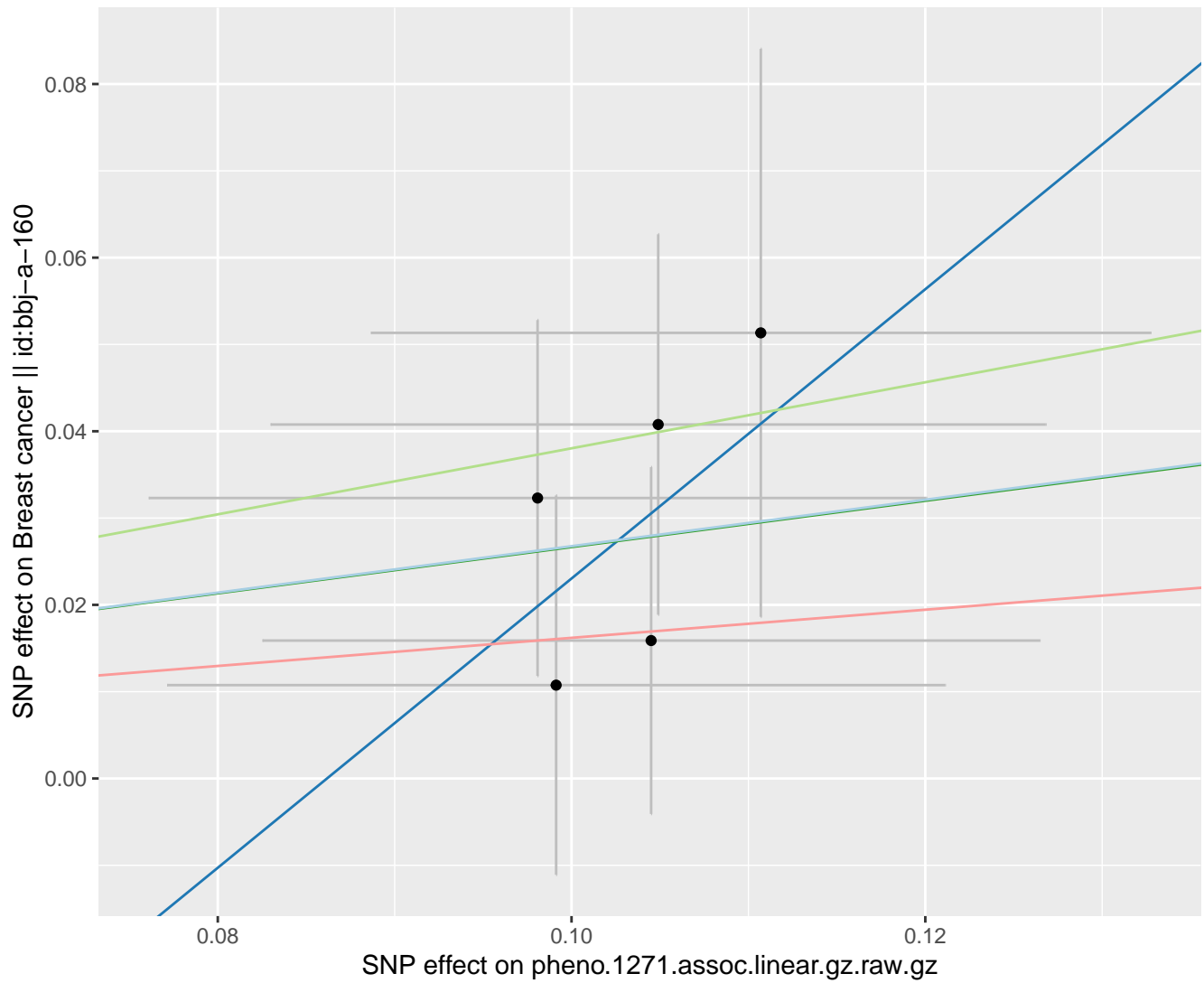

Supplement: Supplementary file 1 [file DataSheet1.ZIP › Supplementary Materials/MR plots for tongue/tongue═╝/Breast cancer/pheno.1271_to_breast cancer_scatter.pdf]

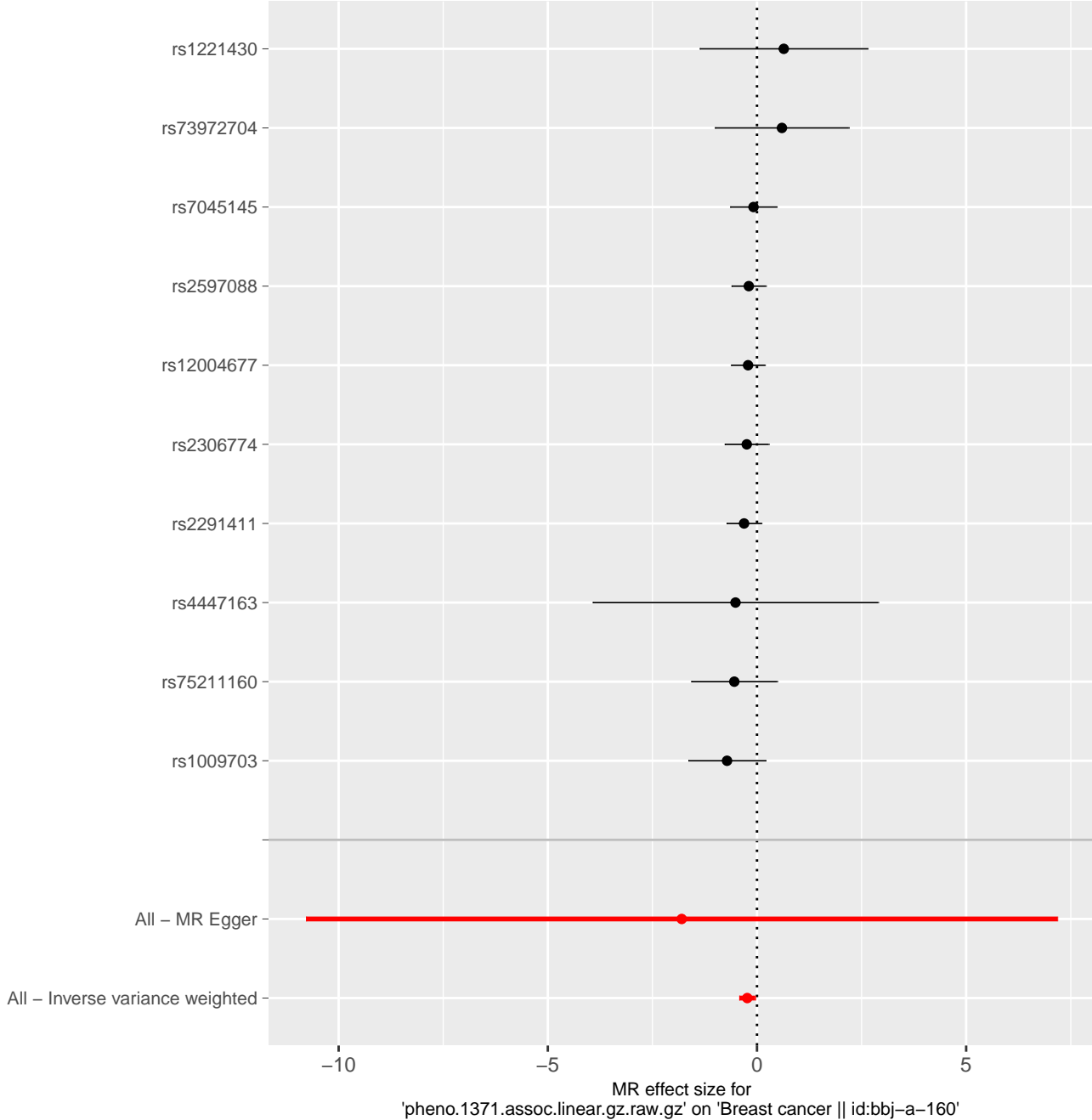

Supplement: Supplementary file 1 [file DataSheet1.ZIP › Supplementary Materials/MR plots for tongue/tongue═╝/Breast cancer/pheno.1371_to_breast cancer_forest.pdf]

# MR Method

- Inverse variance weighted
- MR Egger

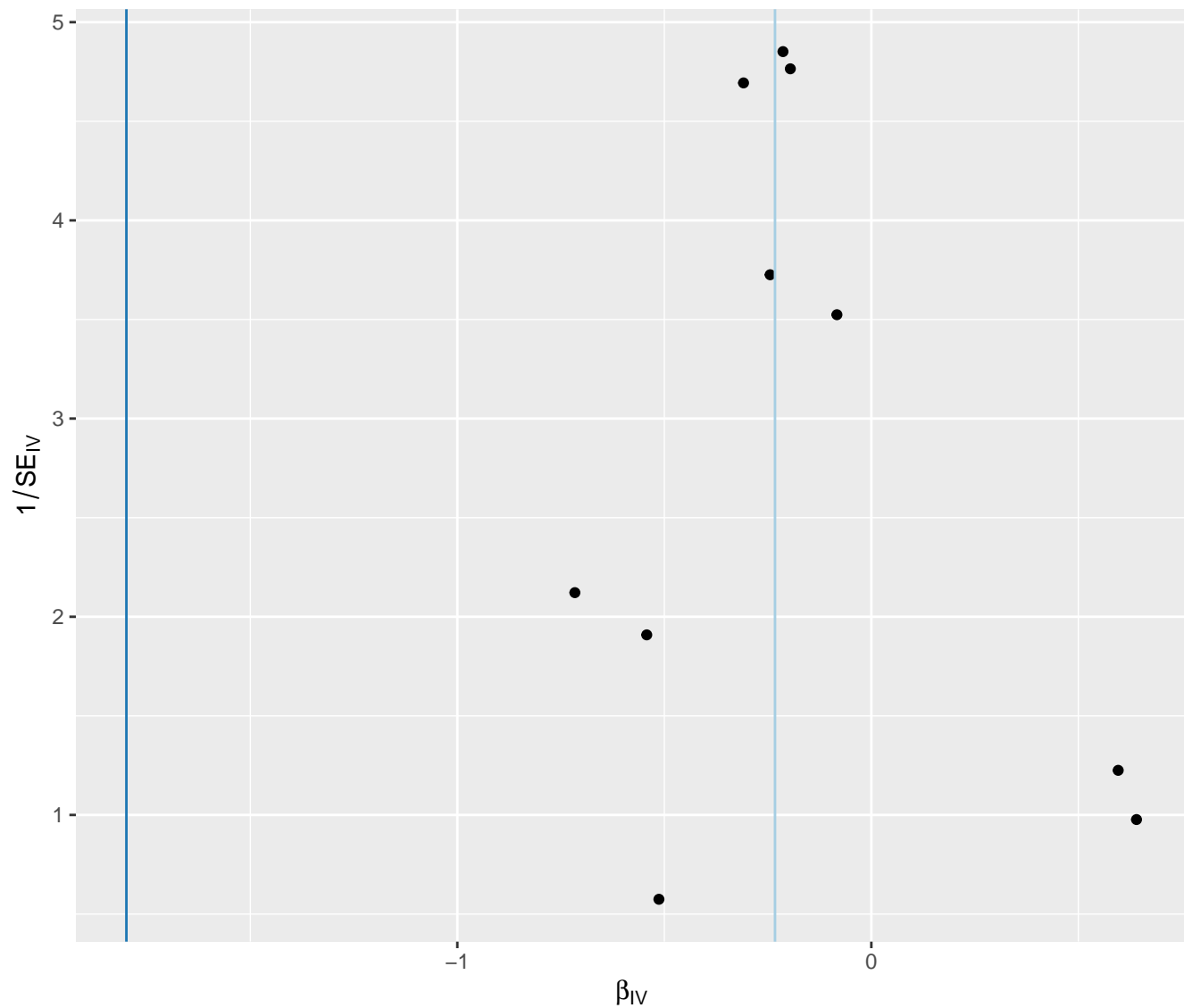

Supplement: Supplementary file 1 [file DataSheet1.ZIP › Supplementary Materials/MR plots for tongue/tongue═╝/Breast cancer/pheno.1371_to_breast cancer_funnel.pdf]

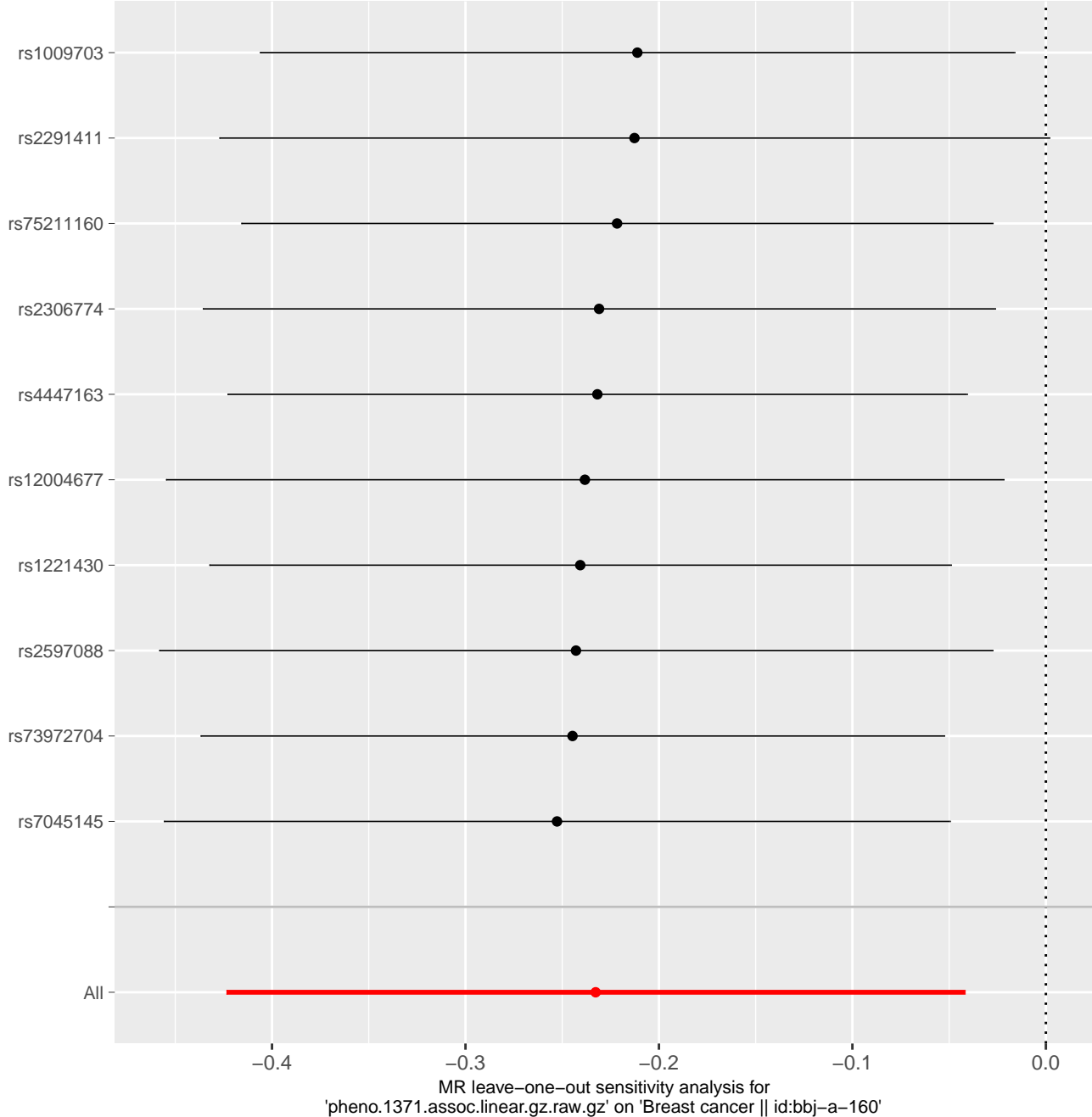

Supplement: Supplementary file 1 [file DataSheet1.ZIP › Supplementary Materials/MR plots for tongue/tongue═╝/Breast cancer/pheno.1371_to_breast cancer_leave_one_out.pdf]

# MR Test

- Inverse variance weighted
- MR Egger
- Simple mode
- Weighted median
- Weighted mode

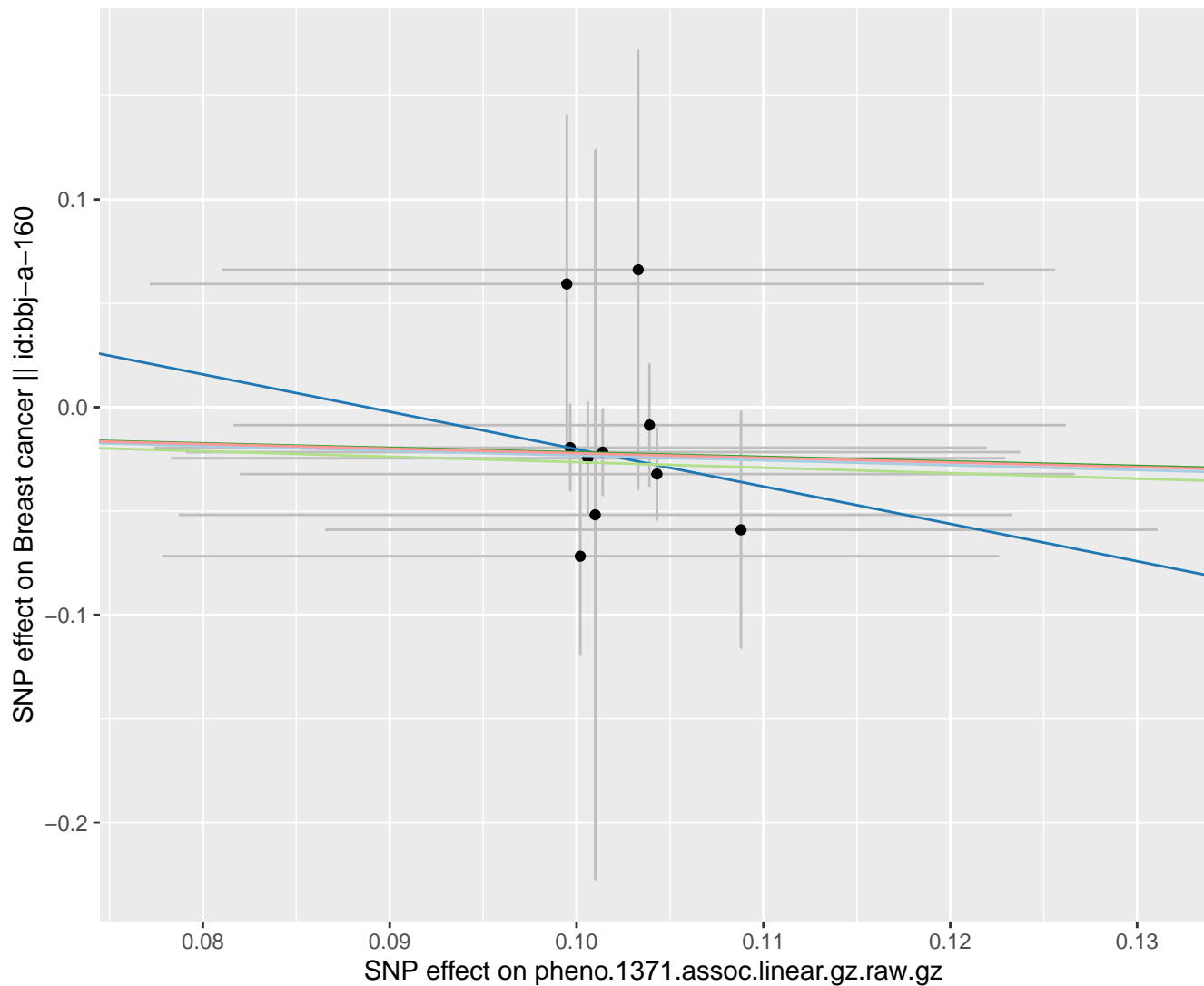

Supplement: Supplementary file 1 [file DataSheet1.ZIP › Supplementary Materials/MR plots for tongue/tongue═╝/Breast cancer/pheno.1371_to_breast cancer_scatter.pdf]

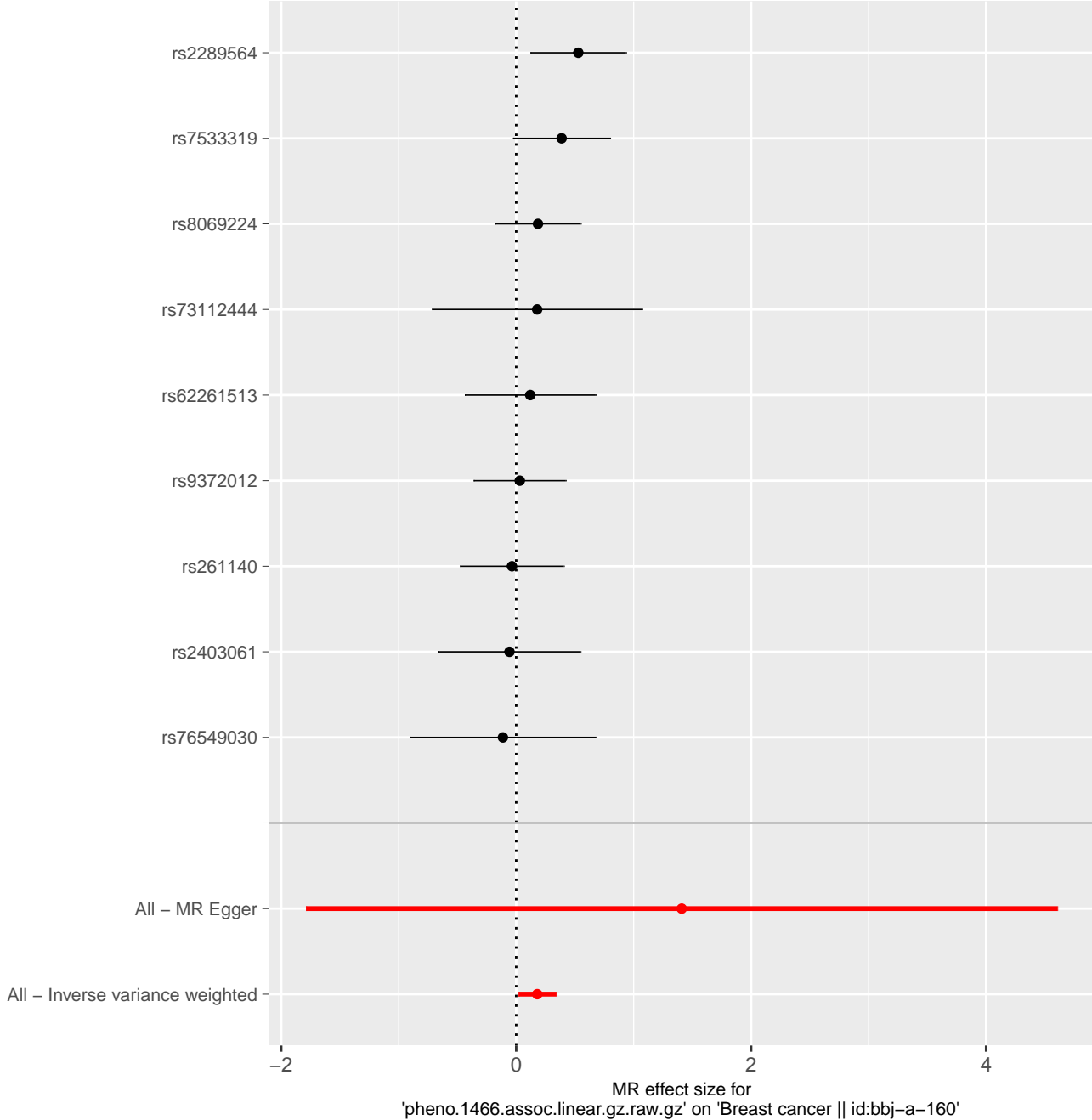

Supplement: Supplementary file 1 [file DataSheet1.ZIP › Supplementary Materials/MR plots for tongue/tongue═╝/Breast cancer/pheno.1466_to_breast cancer_forest.pdf]

# MR Method

- Inverse variance weighted
- MR Egger

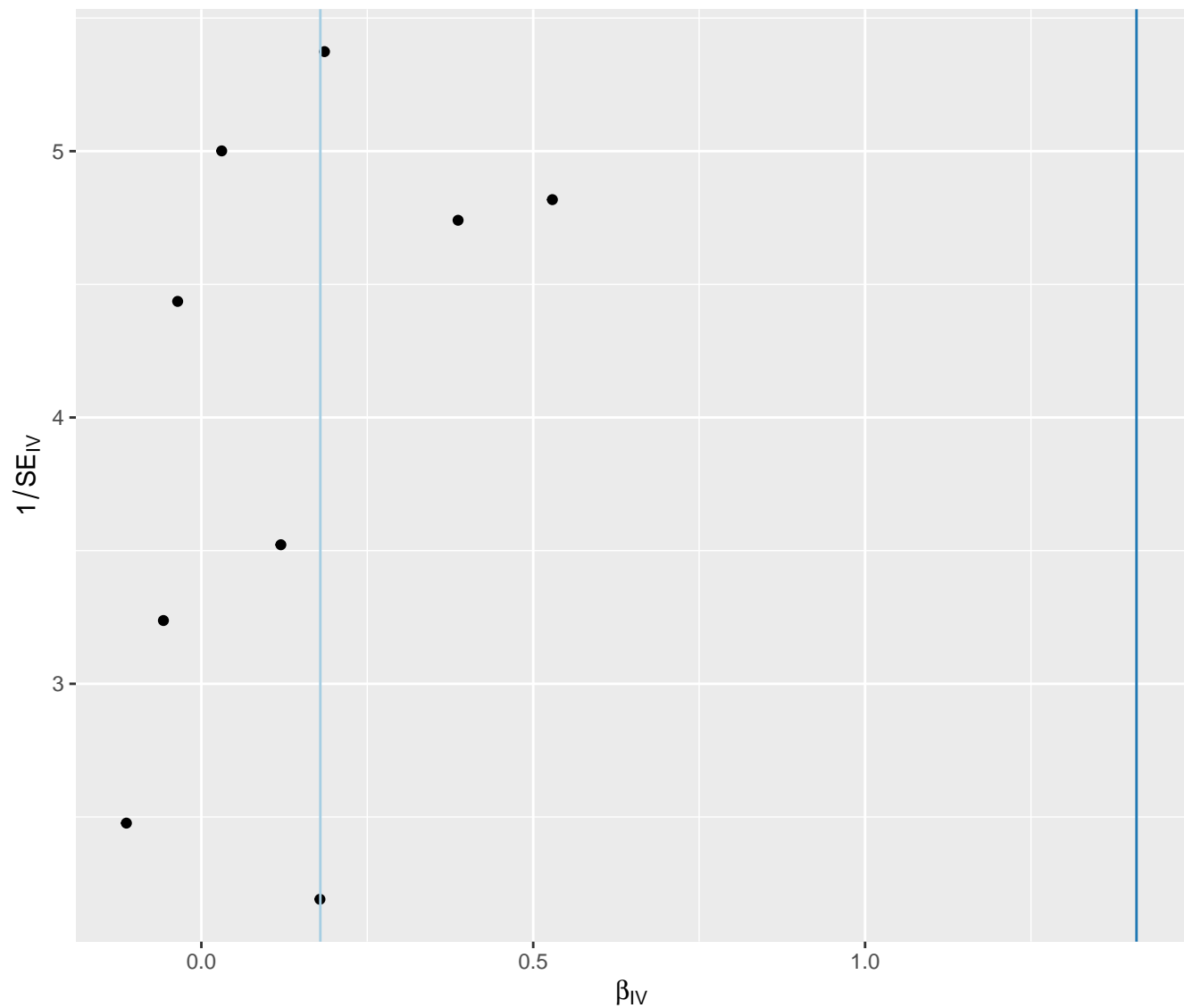

Supplement: Supplementary file 1 [file DataSheet1.ZIP › Supplementary Materials/MR plots for tongue/tongue═╝/Breast cancer/pheno.1466_to_breast cancer_funnel.pdf]

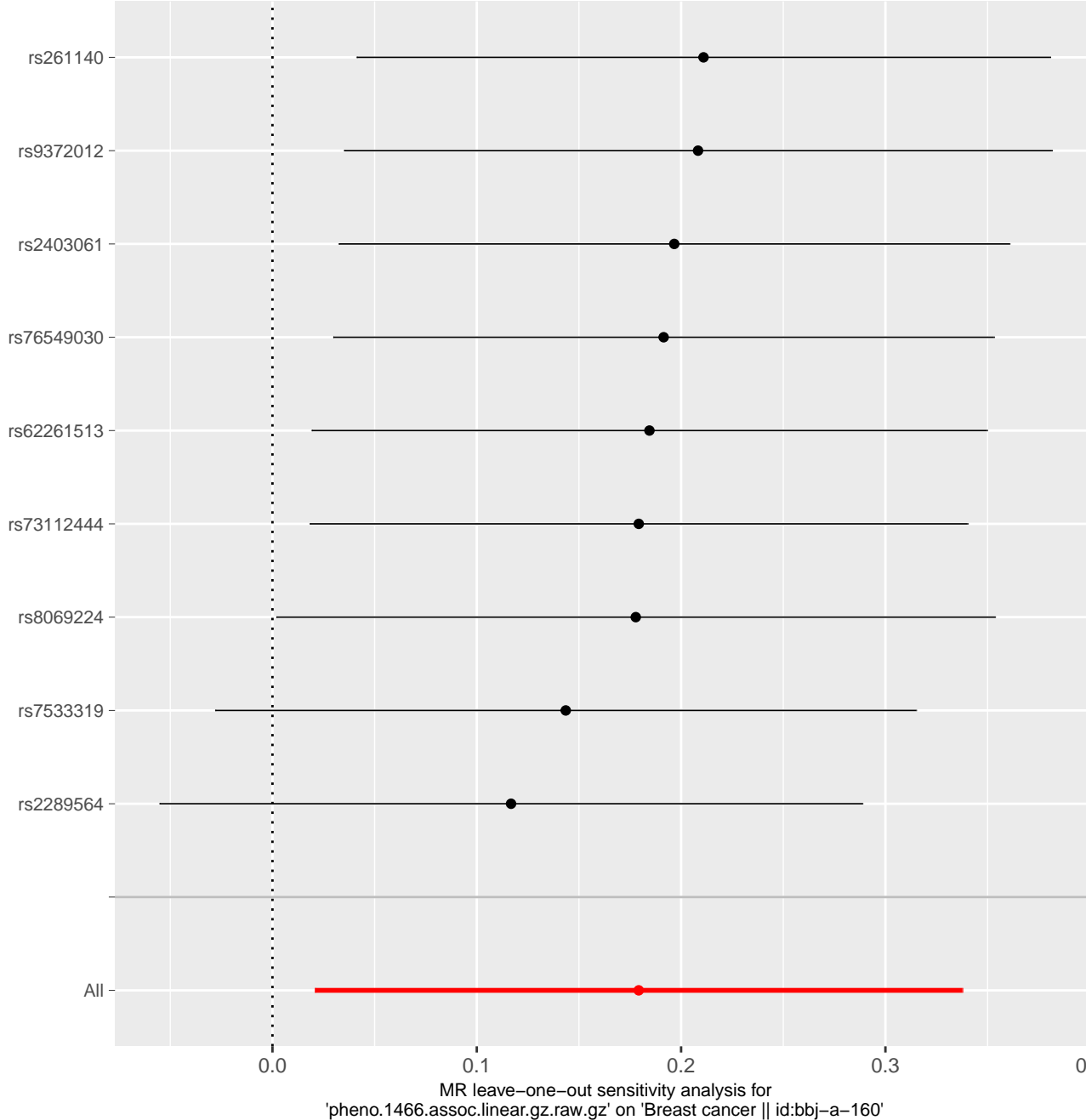

Supplement: Supplementary file 1 [file DataSheet1.ZIP › Supplementary Materials/MR plots for tongue/tongue═╝/Breast cancer/pheno.1466_to_breast cancer_leave_one_out.pdf]

# MR Test

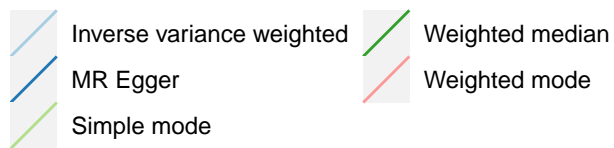

SNP effect on Breast cancer || id:bbj-a-160

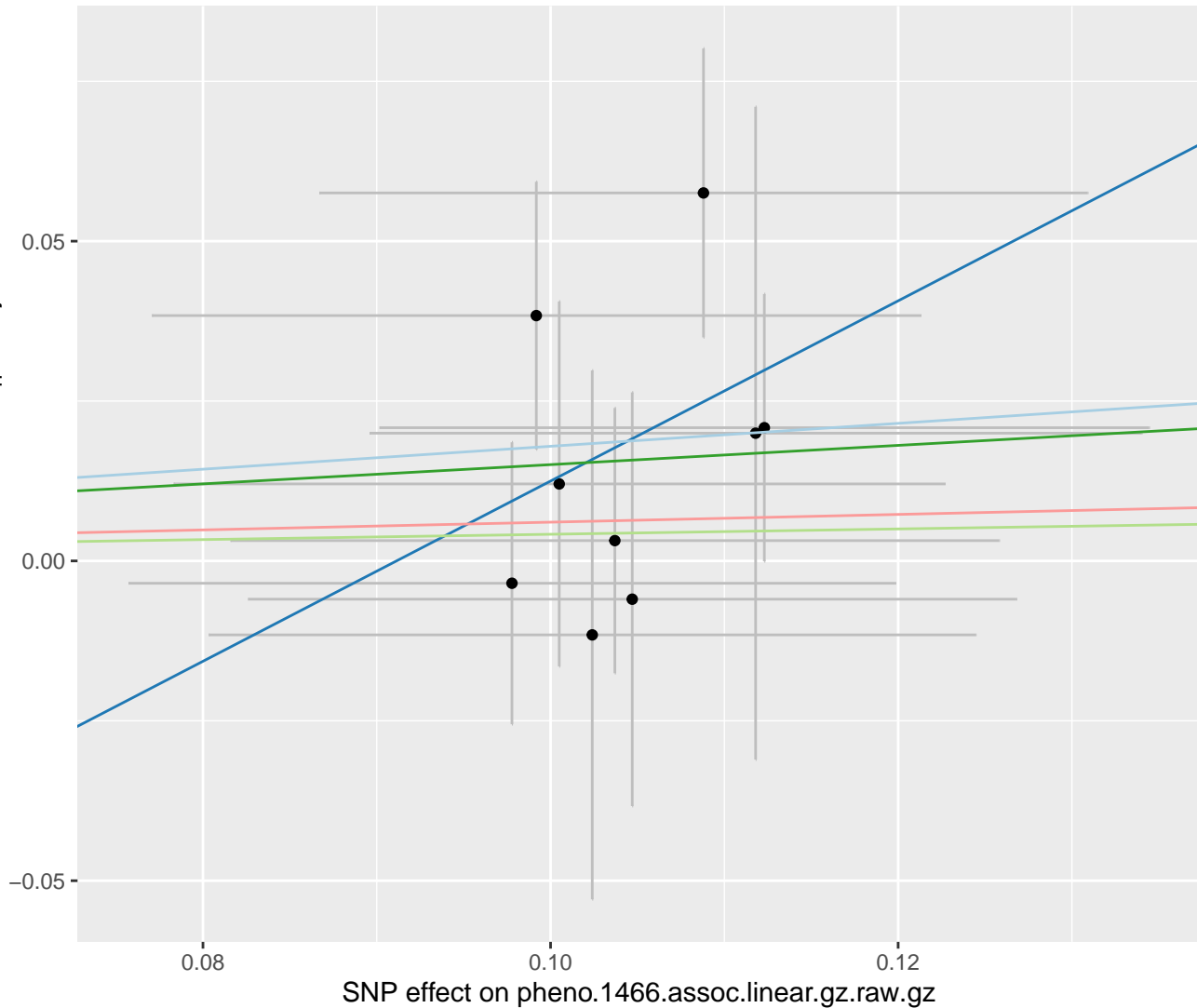

Supplement: Supplementary file 1 [file DataSheet1.ZIP › Supplementary Materials/MR plots for tongue/tongue═╝/Breast cancer/pheno.1466_to_breast cancer_scatter.pdf]

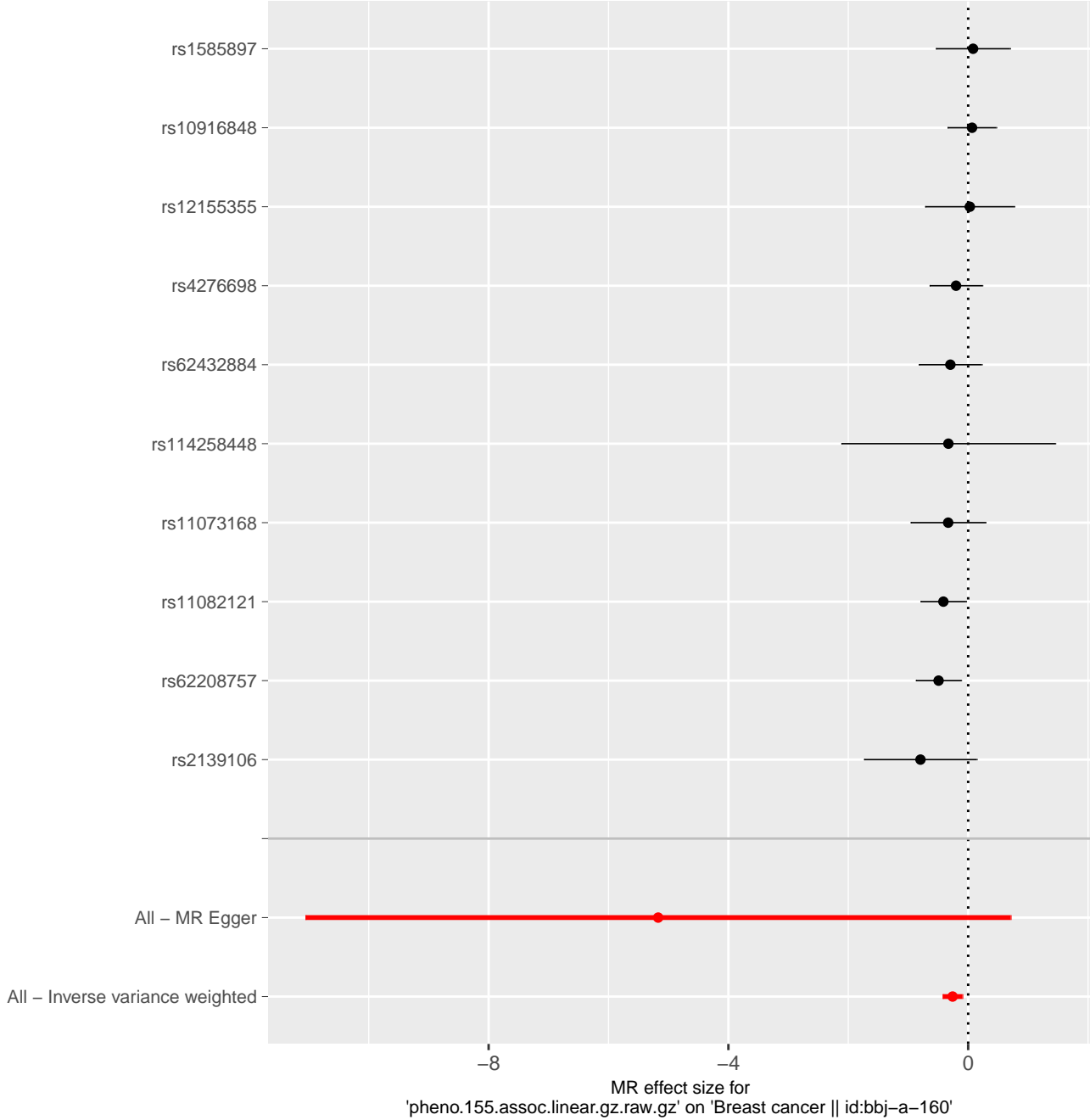

Supplement: Supplementary file 1 [file DataSheet1.ZIP › Supplementary Materials/MR plots for tongue/tongue═╝/Breast cancer/pheno.155_to_breast cancer_forest.pdf]

# MR Method

- Inverse variance weighted
- MR Egger

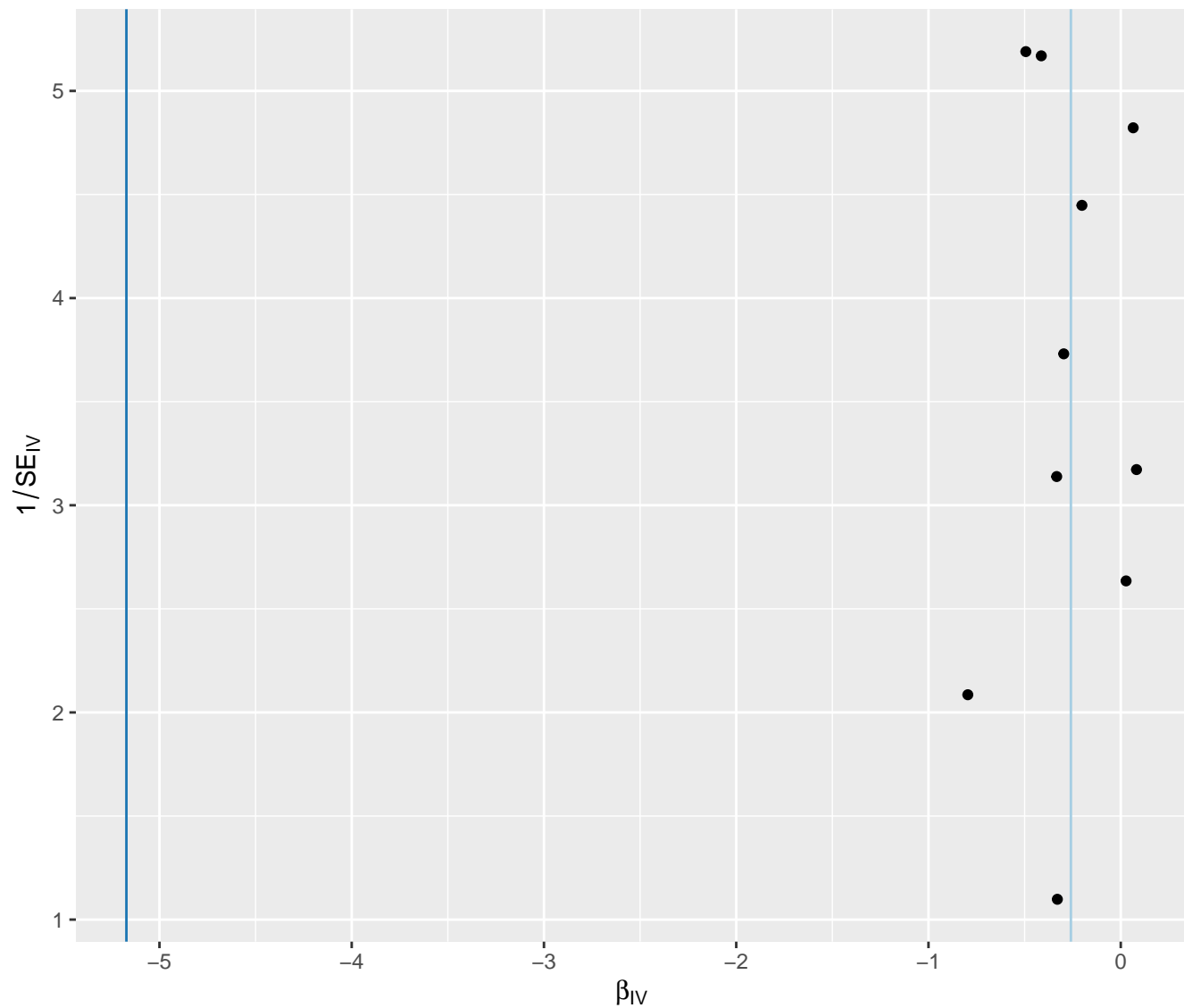

Supplement: Supplementary file 1 [file DataSheet1.ZIP › Supplementary Materials/MR plots for tongue/tongue═╝/Breast cancer/pheno.155_to_breast cancer_funnel.pdf]

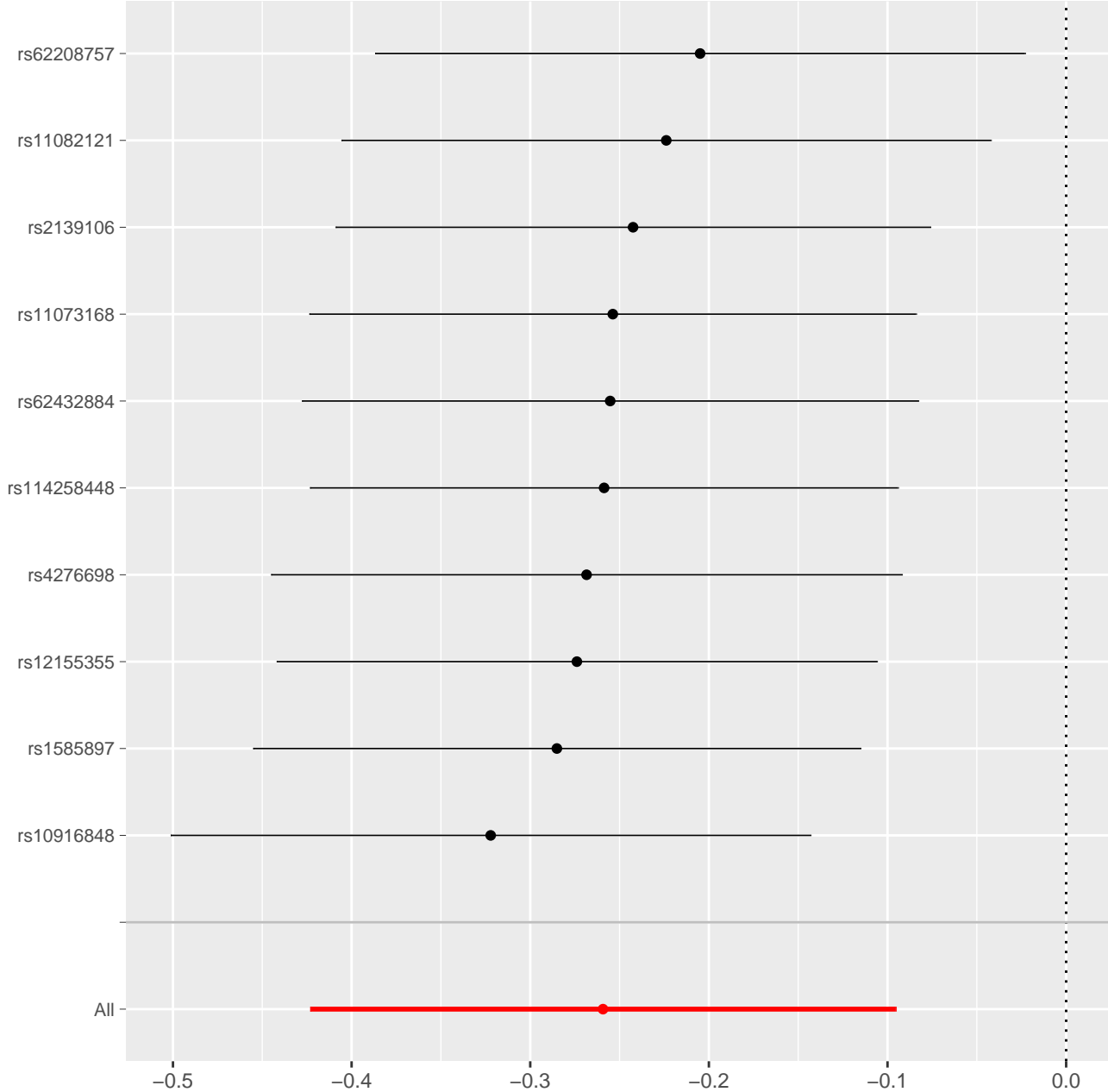

MR leave-one-out sensitivity analysis for  
'pheno.155.assoc.linear.gz.raw.gz' on 'Breast cancer || id:bbj-a-160'

Supplement: Supplementary file 1 [file DataSheet1.ZIP › Supplementary Materials/MR plots for tongue/tongue═╝/Breast cancer/pheno.155_to_breast cancer_leave_one_out.pdf]

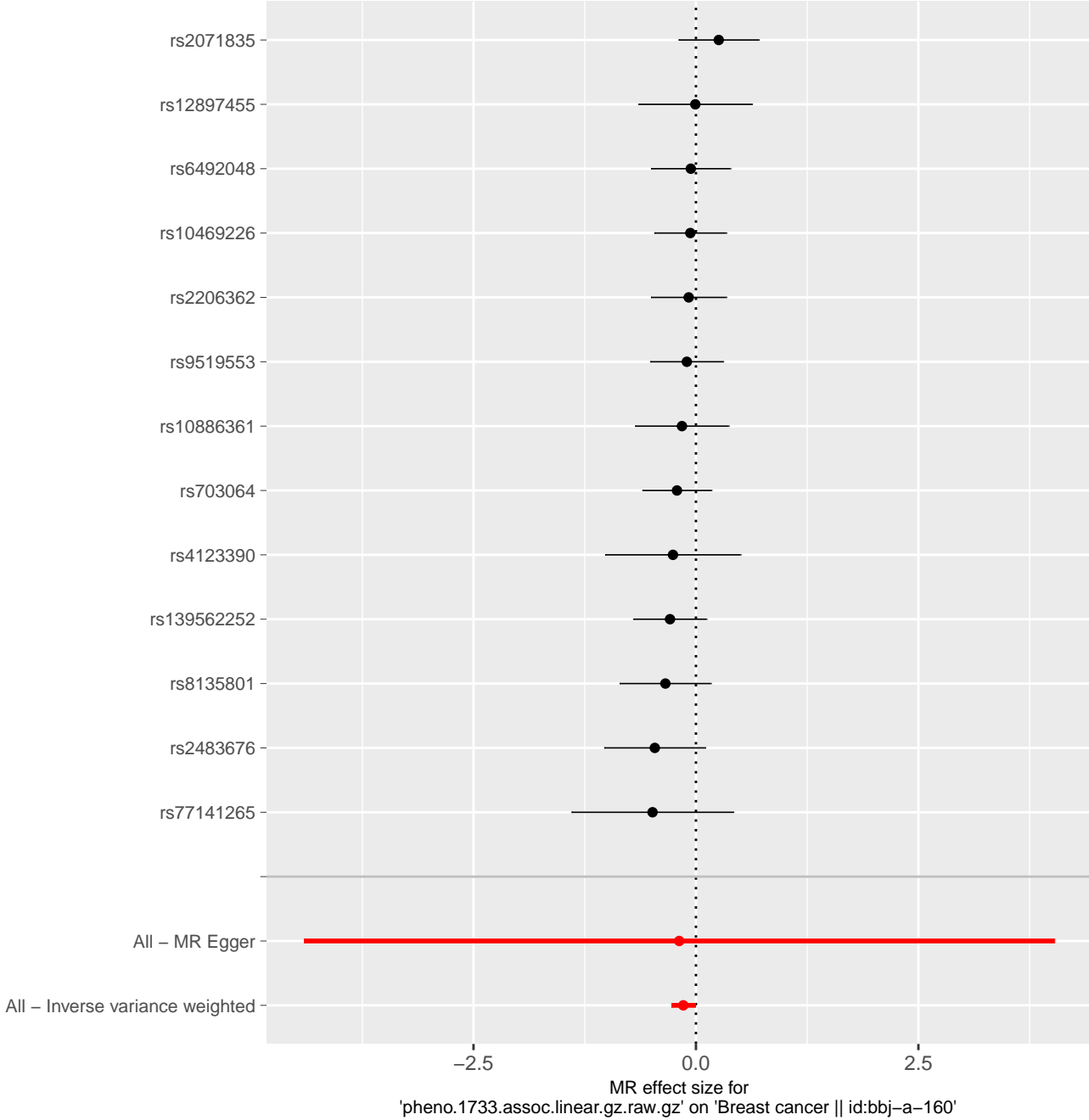

Supplement: Supplementary file 1 [file DataSheet1.ZIP › Supplementary Materials/MR plots for tongue/tongue═╝/Breast cancer/pheno.1733_to_breast cancer_forest.pdf]

# MR Method

- Inverse variance weighted
- MR Egger

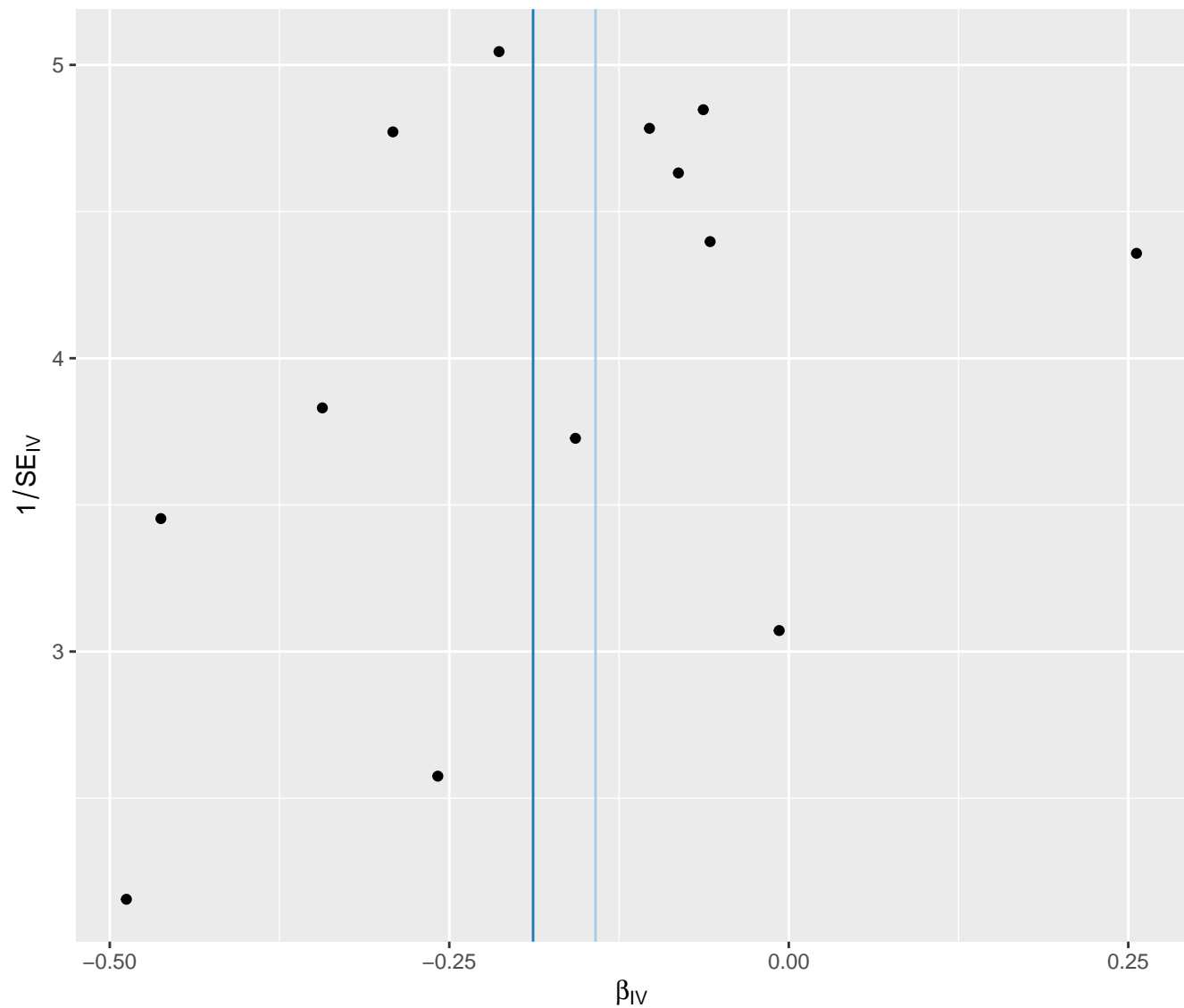

Supplement: Supplementary file 1 [file DataSheet1.ZIP › Supplementary Materials/MR plots for tongue/tongue═╝/Breast cancer/pheno.1733_to_breast cancer_funnel.pdf]

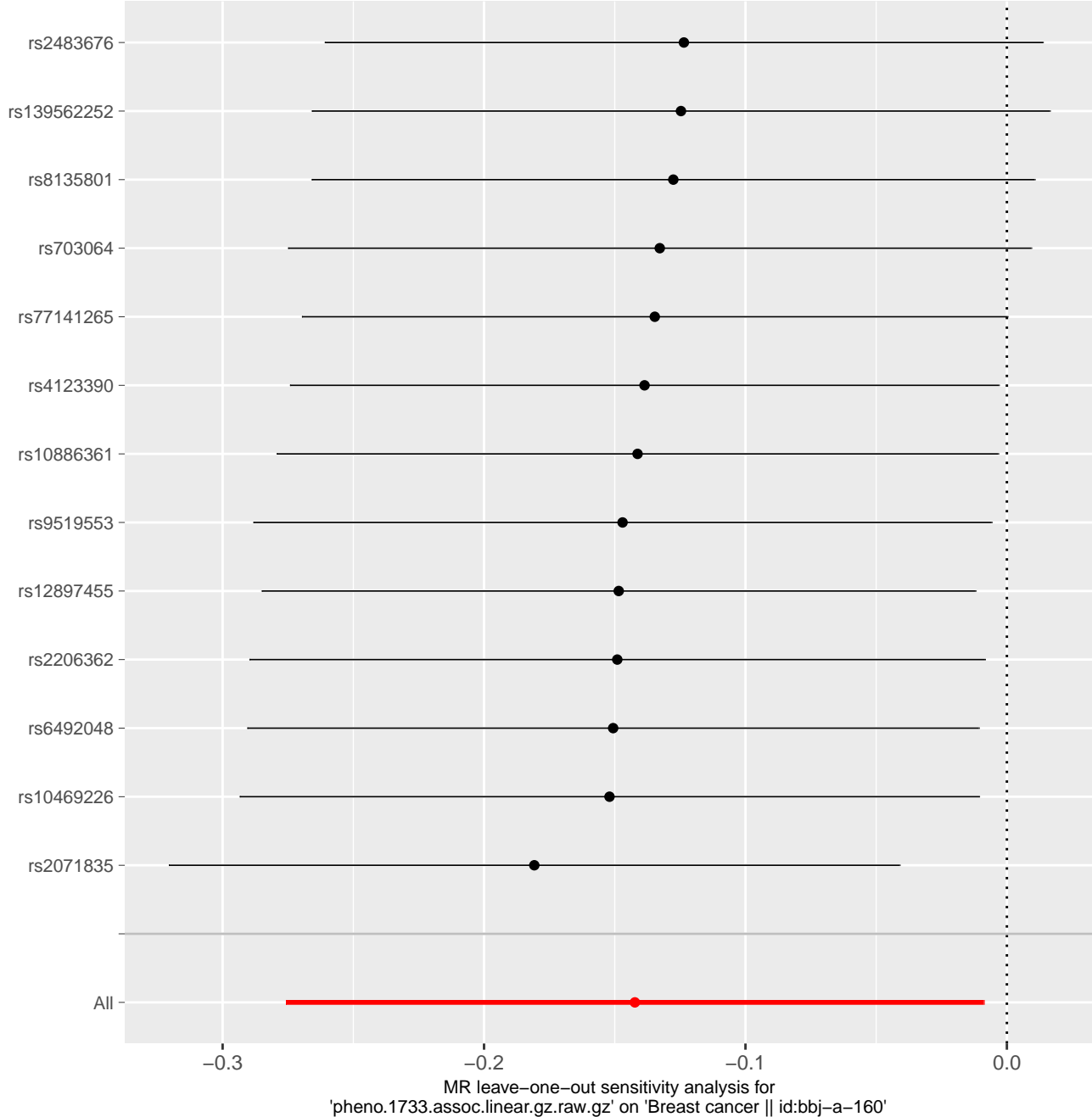

Supplement: Supplementary file 1 [file DataSheet1.ZIP › Supplementary Materials/MR plots for tongue/tongue═╝/Breast cancer/pheno.1733_to_breast cancer_leave_one_out.pdf]

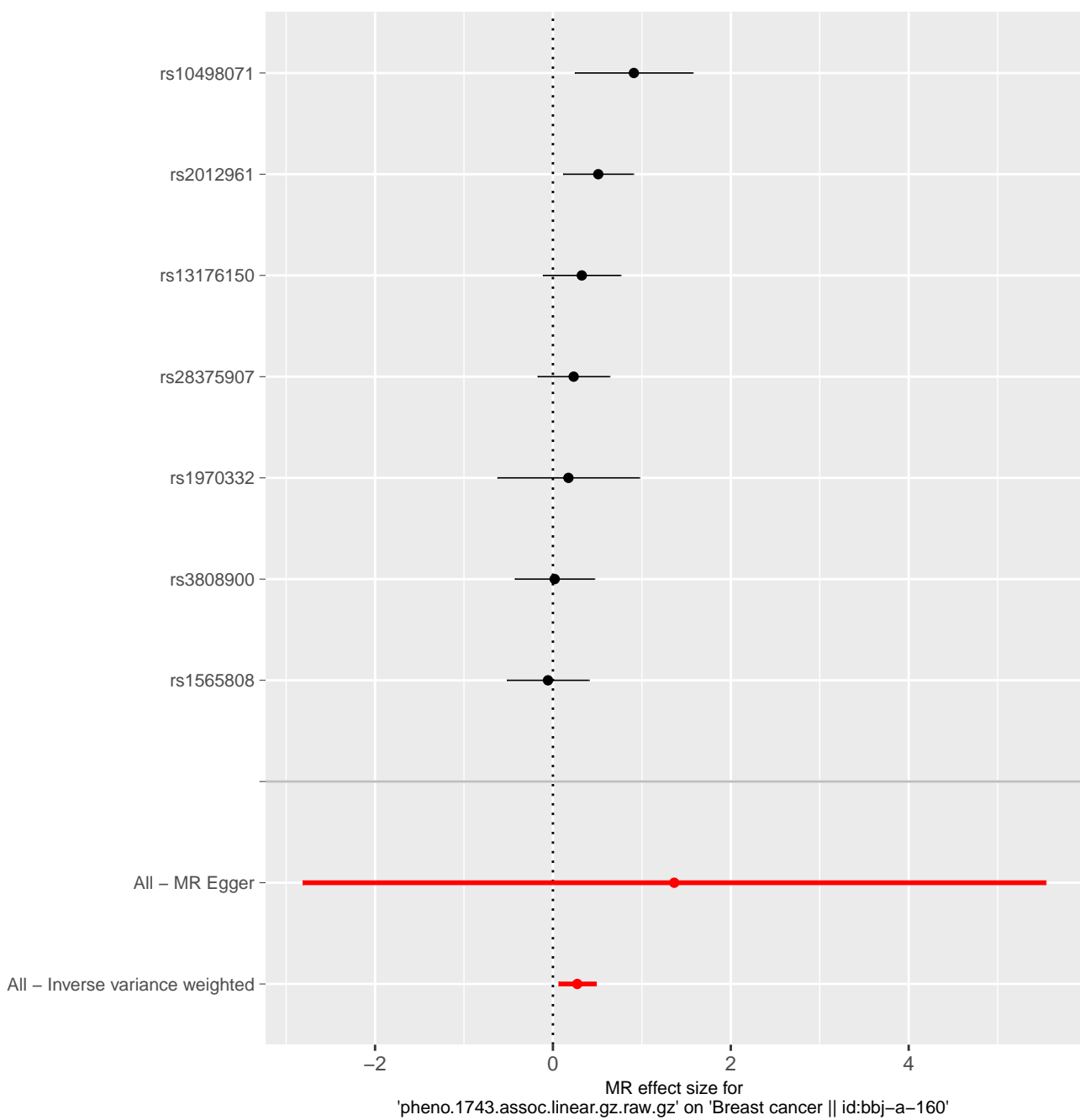

Supplement: Supplementary file 1 [file DataSheet1.ZIP › Supplementary Materials/MR plots for tongue/tongue═╝/Breast cancer/pheno.1743_to_breast cancer_forest.pdf]

# MR Method

- Inverse variance weighted
- MR Egger

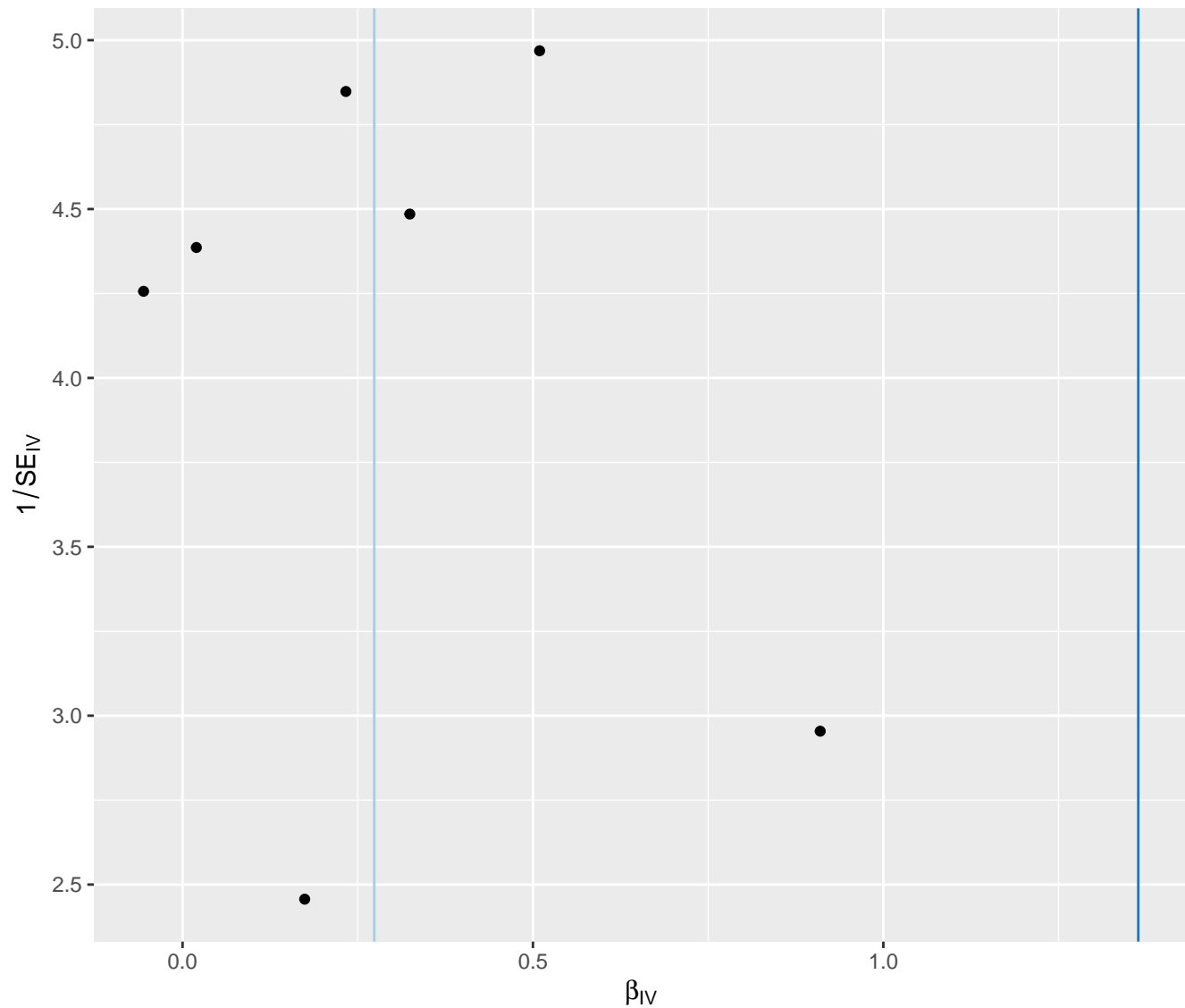

Supplement: Supplementary file 1 [file DataSheet1.ZIP › Supplementary Materials/MR plots for tongue/tongue═╝/Breast cancer/pheno.1743_to_breast cancer_funnel.pdf]

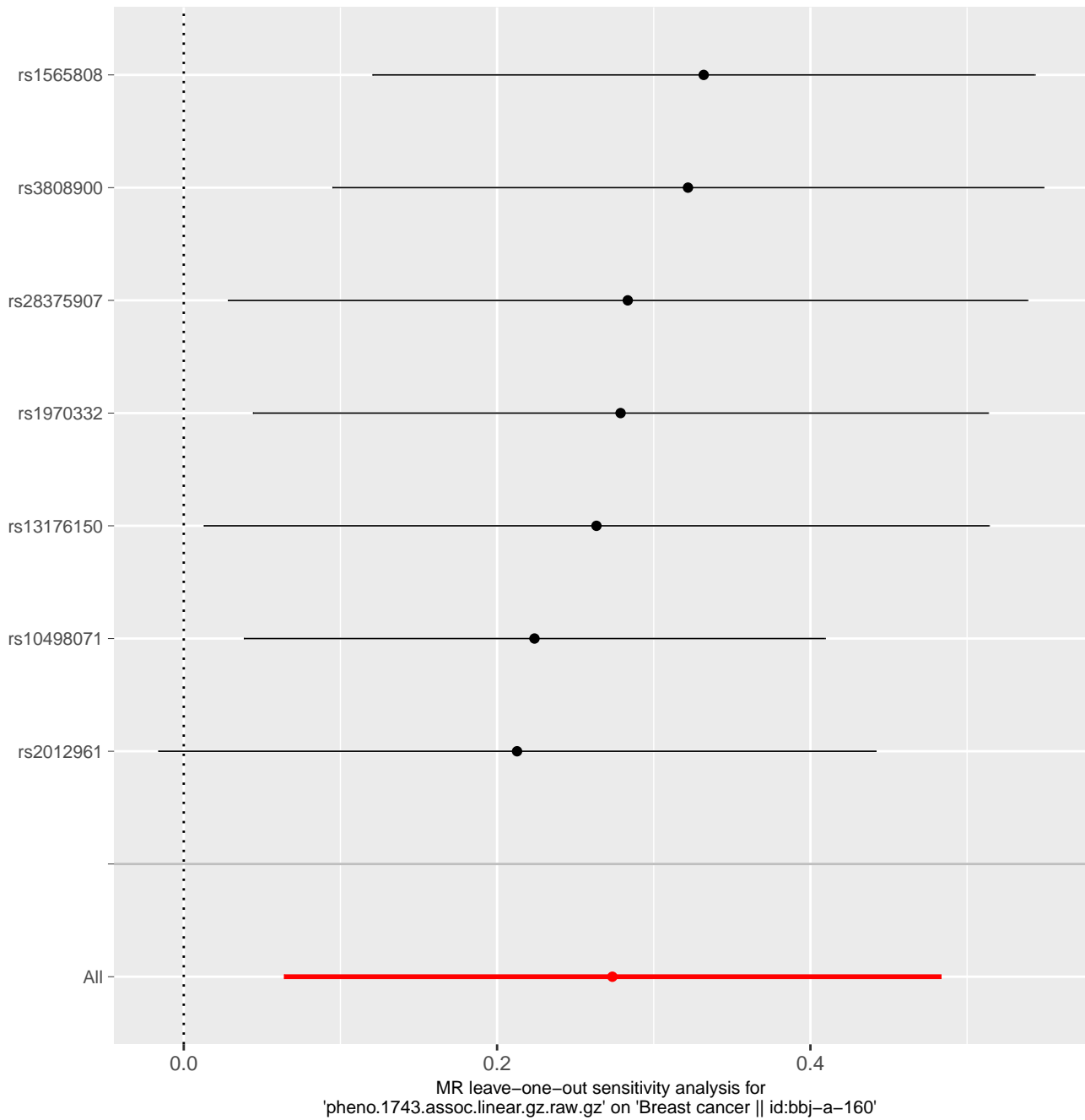

Supplement: Supplementary file 1 [file DataSheet1.ZIP › Supplementary Materials/MR plots for tongue/tongue═╝/Breast cancer/pheno.1743_to_breast cancer_leave_one_out.pdf]

# MR Test

- Inverse variance weighted
- MR Egger
- Simple mode
- Weighted median
- Weighted mode

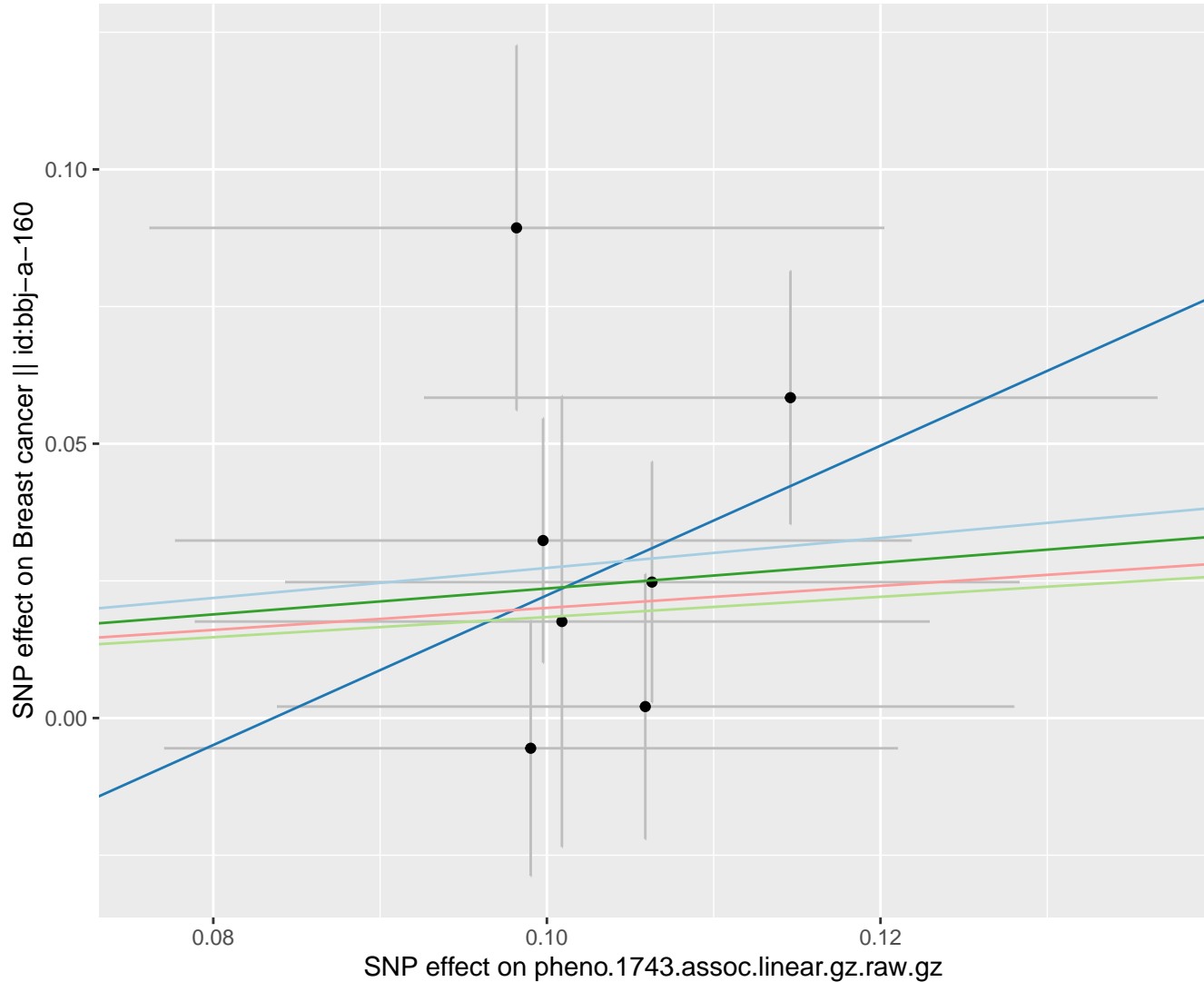

Supplement: Supplementary file 1 [file DataSheet1.ZIP › Supplementary Materials/MR plots for tongue/tongue═╝/Breast cancer/pheno.1743_to_breast cancer_scatter.pdf]

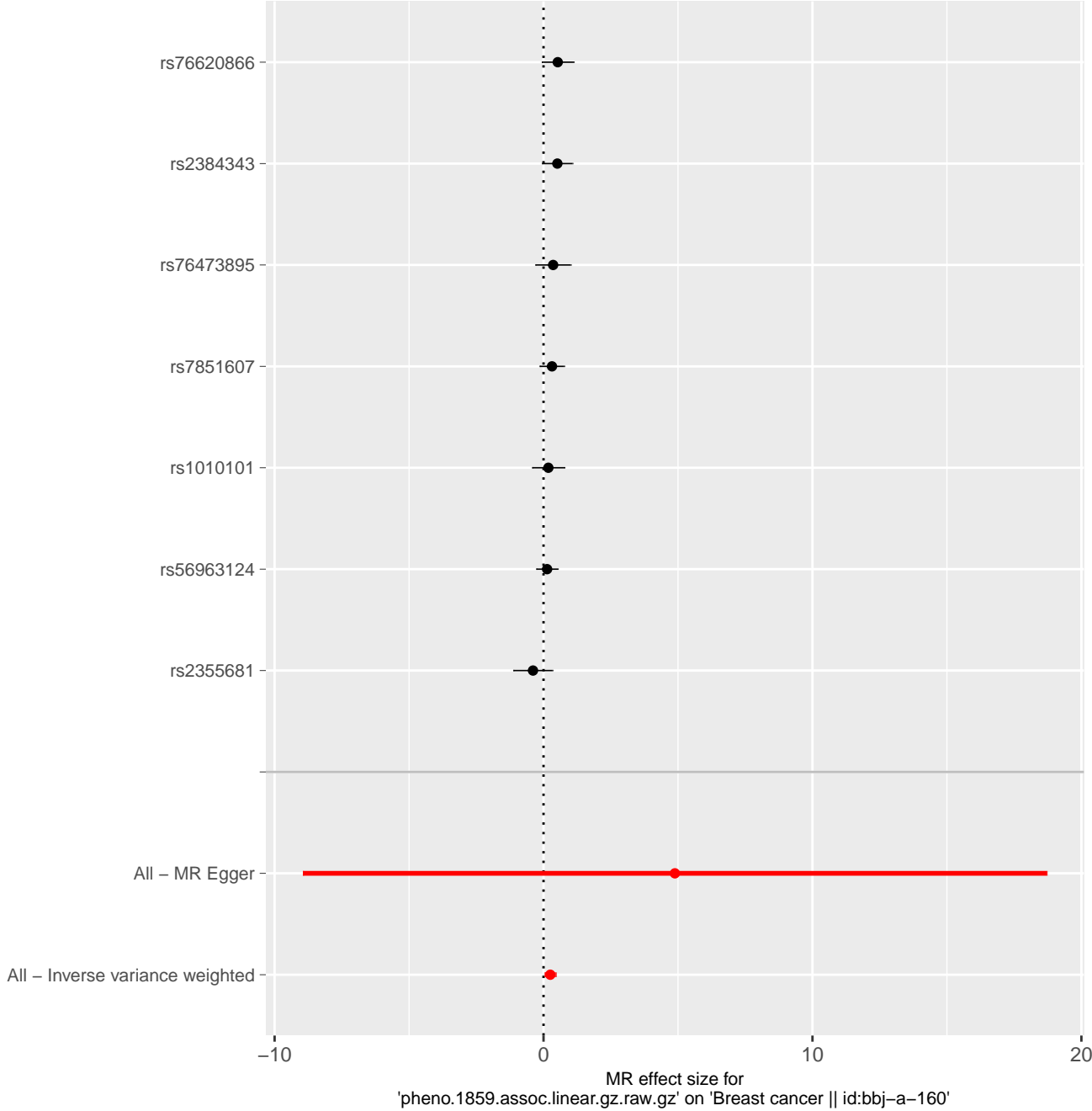

Supplement: Supplementary file 1 [file DataSheet1.ZIP › Supplementary Materials/MR plots for tongue/tongue═╝/Breast cancer/pheno.1859_to_breast cancer_forest.pdf]

# MR Method

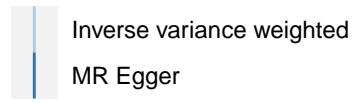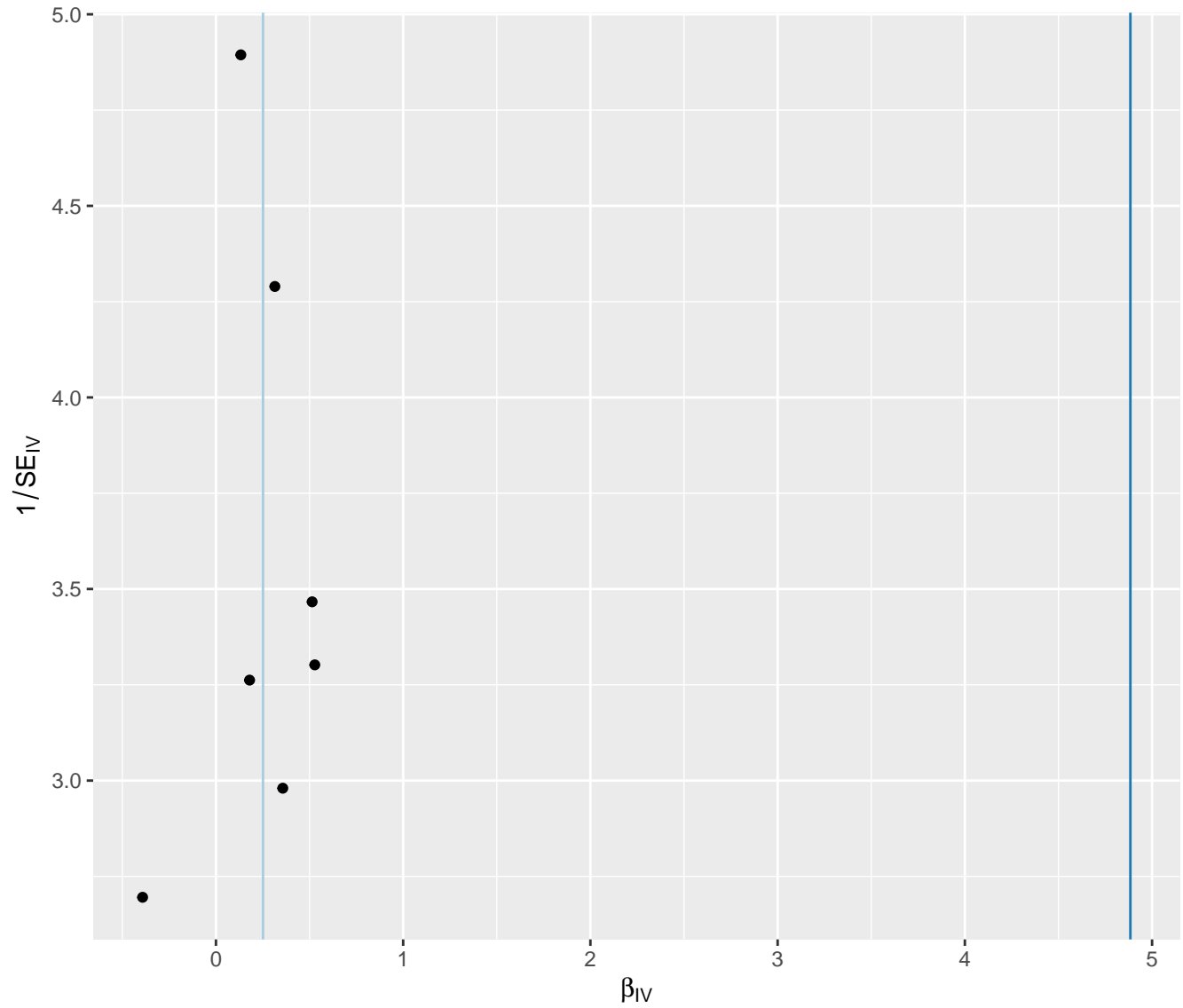

Supplement: Supplementary file 1 [file DataSheet1.ZIP › Supplementary Materials/MR plots for tongue/tongue═╝/Breast cancer/pheno.1859_to_breast cancer_funnel.pdf]

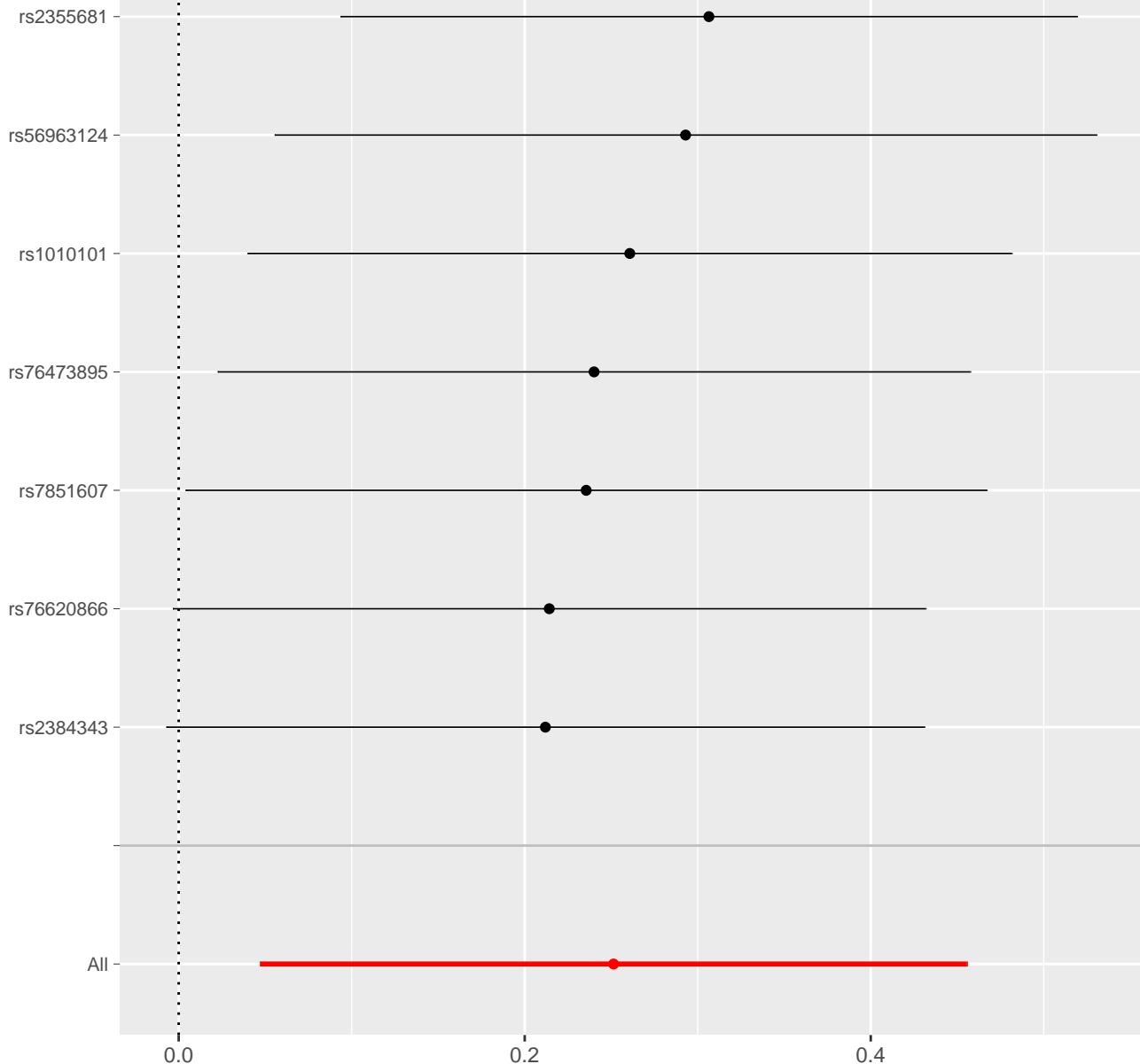

Supplement: Supplementary file 1 [file DataSheet1.ZIP › Supplementary Materials/MR plots for tongue/tongue═╝/Breast cancer/pheno.1859_to_breast cancer_leave_one_out.pdf]

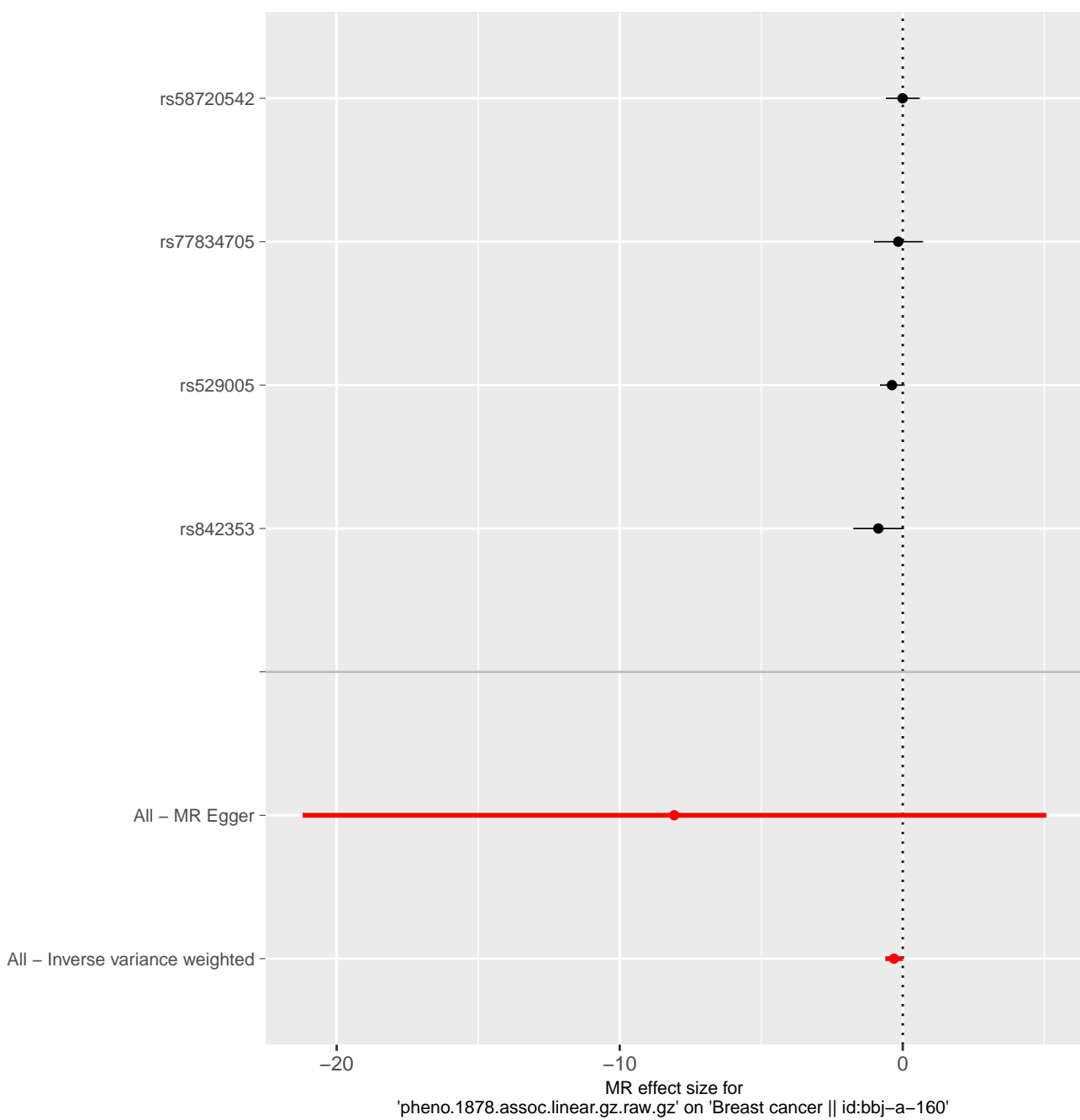

Supplement: Supplementary file 1 [file DataSheet1.ZIP › Supplementary Materials/MR plots for tongue/tongue═╝/Breast cancer/pheno.1878_to_breast cancer_forest.pdf]

# MR Method

- Inverse variance weighted
- MR Egger

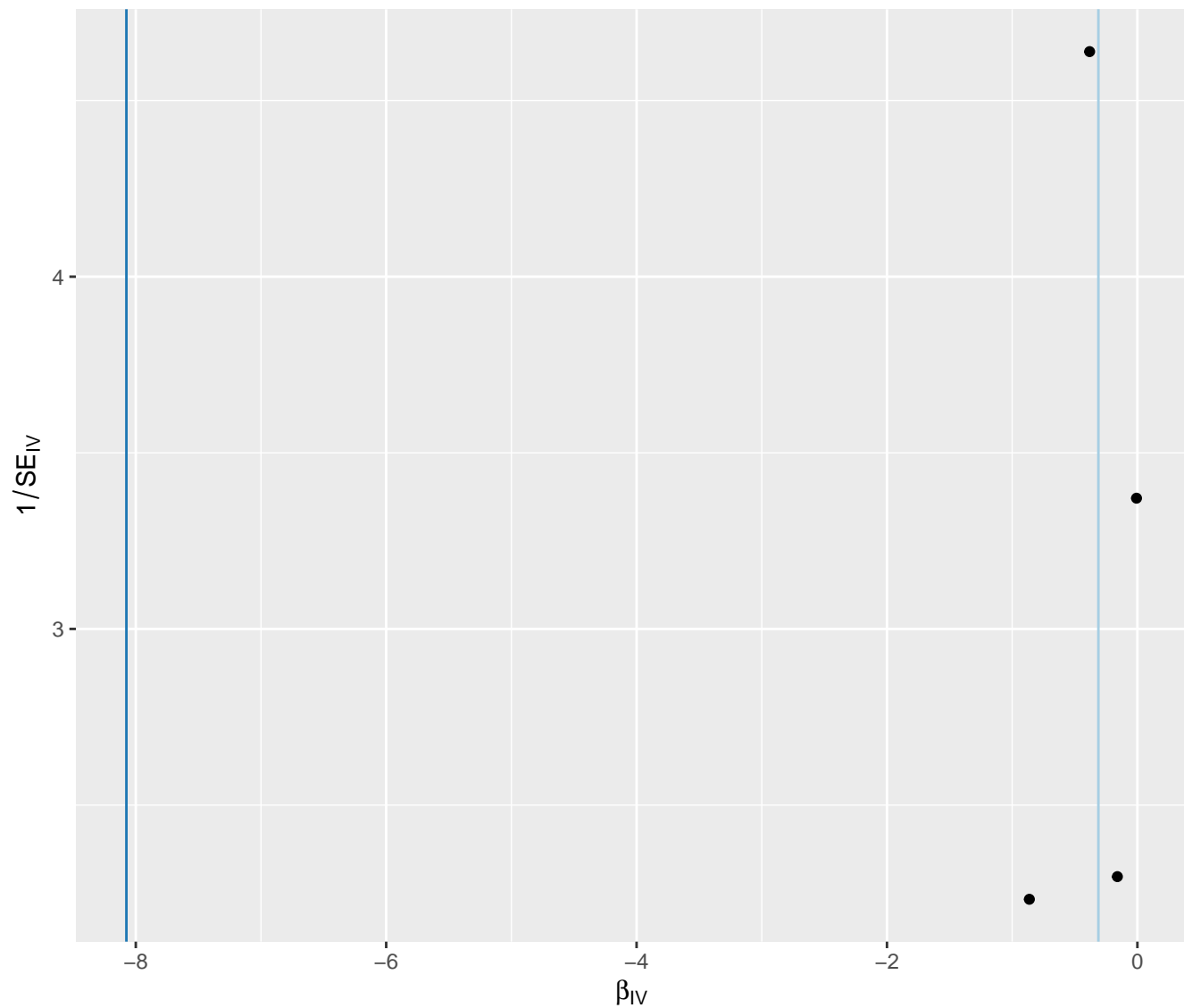

Supplement: Supplementary file 1 [file DataSheet1.ZIP › Supplementary Materials/MR plots for tongue/tongue═╝/Breast cancer/pheno.1878_to_breast cancer_funnel.pdf]

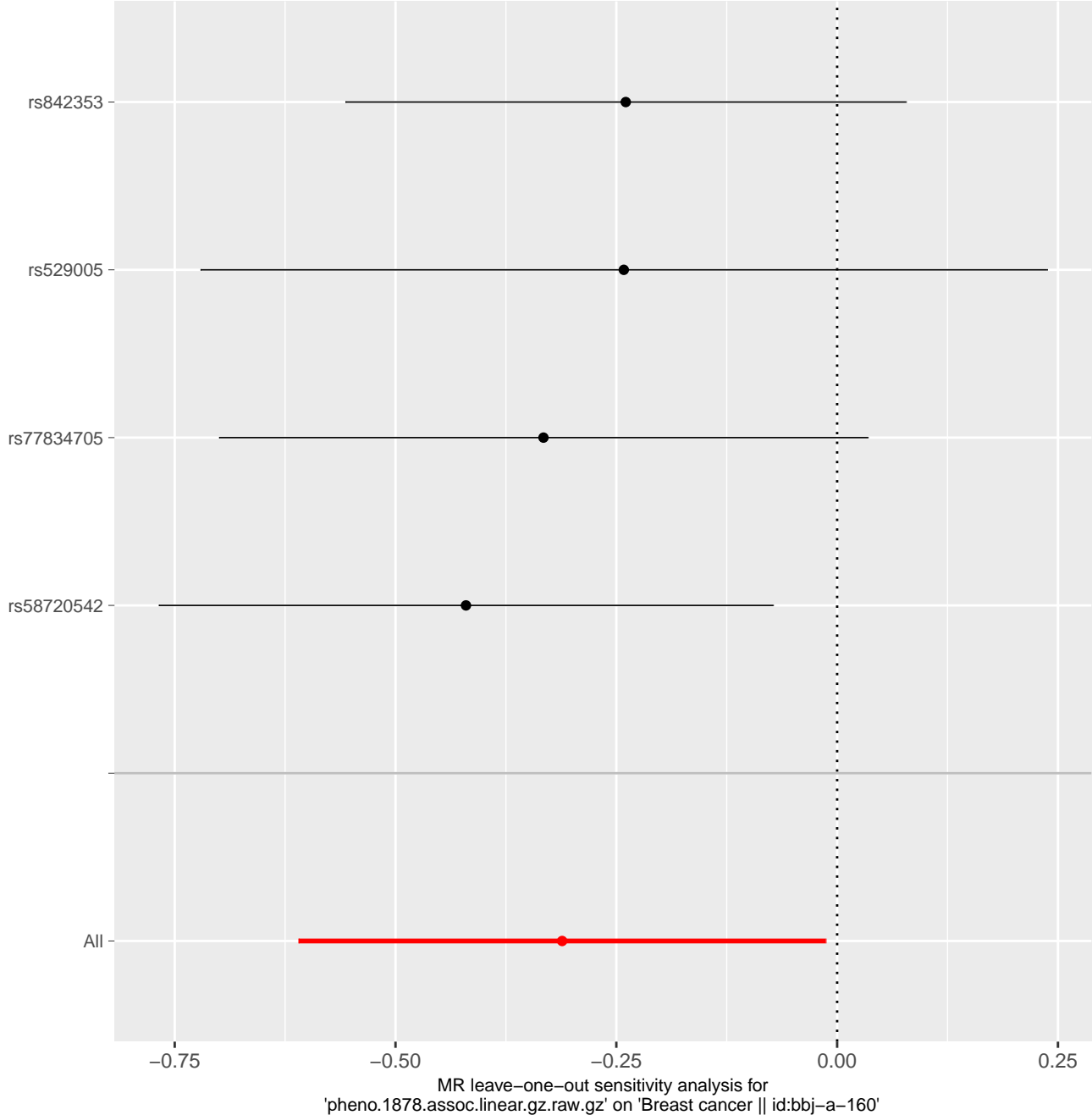

Supplement: Supplementary file 1 [file DataSheet1.ZIP › Supplementary Materials/MR plots for tongue/tongue═╝/Breast cancer/pheno.1878_to_breast cancer_leave_one_out.pdf]

# MR Test

- Inverse variance weighted
- MR Egger
- Simple mode
- Weighted median
- Weighted mode

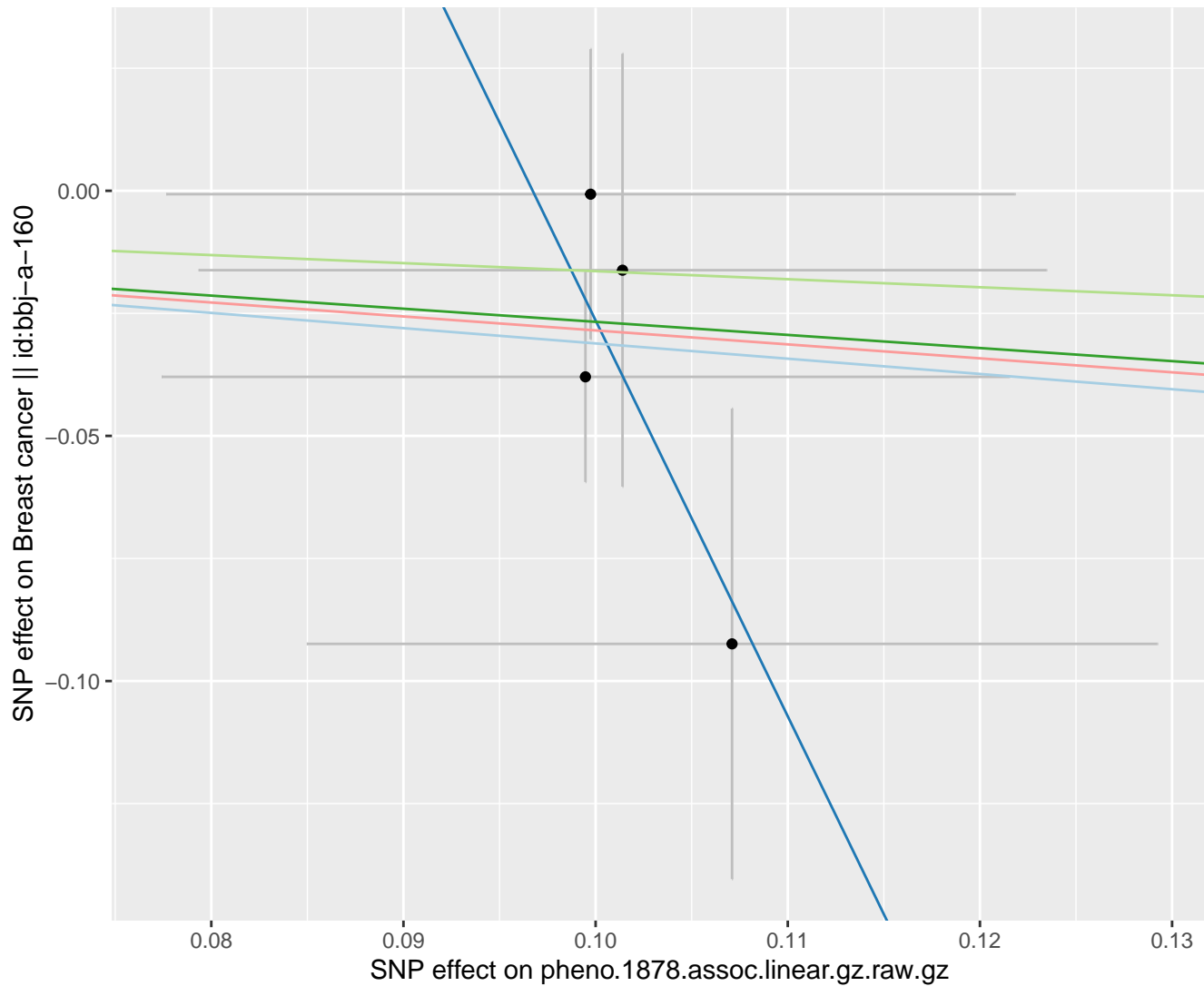

Supplement: Supplementary file 1 [file DataSheet1.ZIP › Supplementary Materials/MR plots for tongue/tongue═╝/Breast cancer/pheno.1878_to_breast cancer_scatter.pdf]

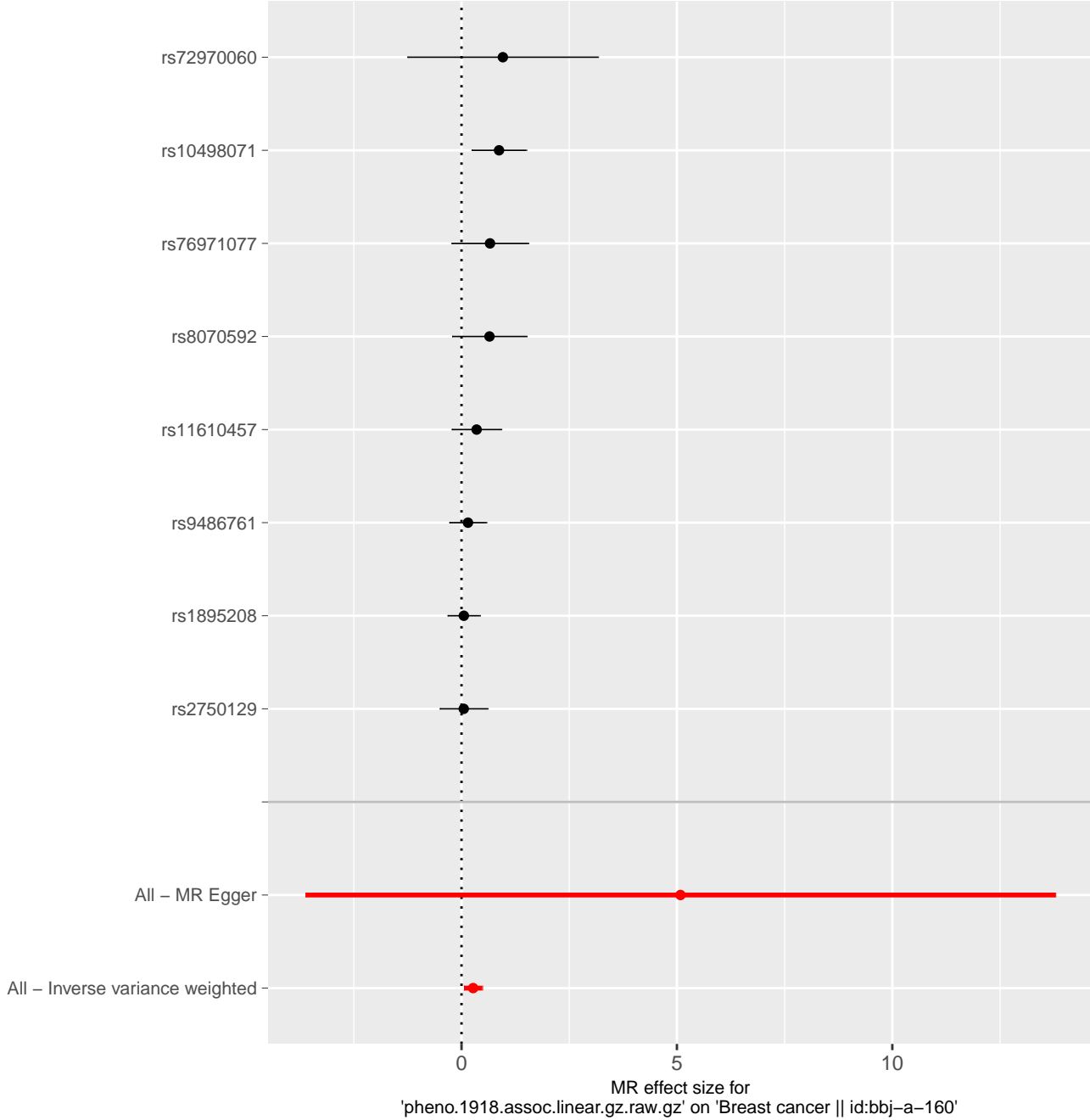

Supplement: Supplementary file 1 [file DataSheet1.ZIP › Supplementary Materials/MR plots for tongue/tongue═╝/Breast cancer/pheno.1918_to_breast cancer_forest.pdf]

# MR Method

- Inverse variance weighted
- MR Egger

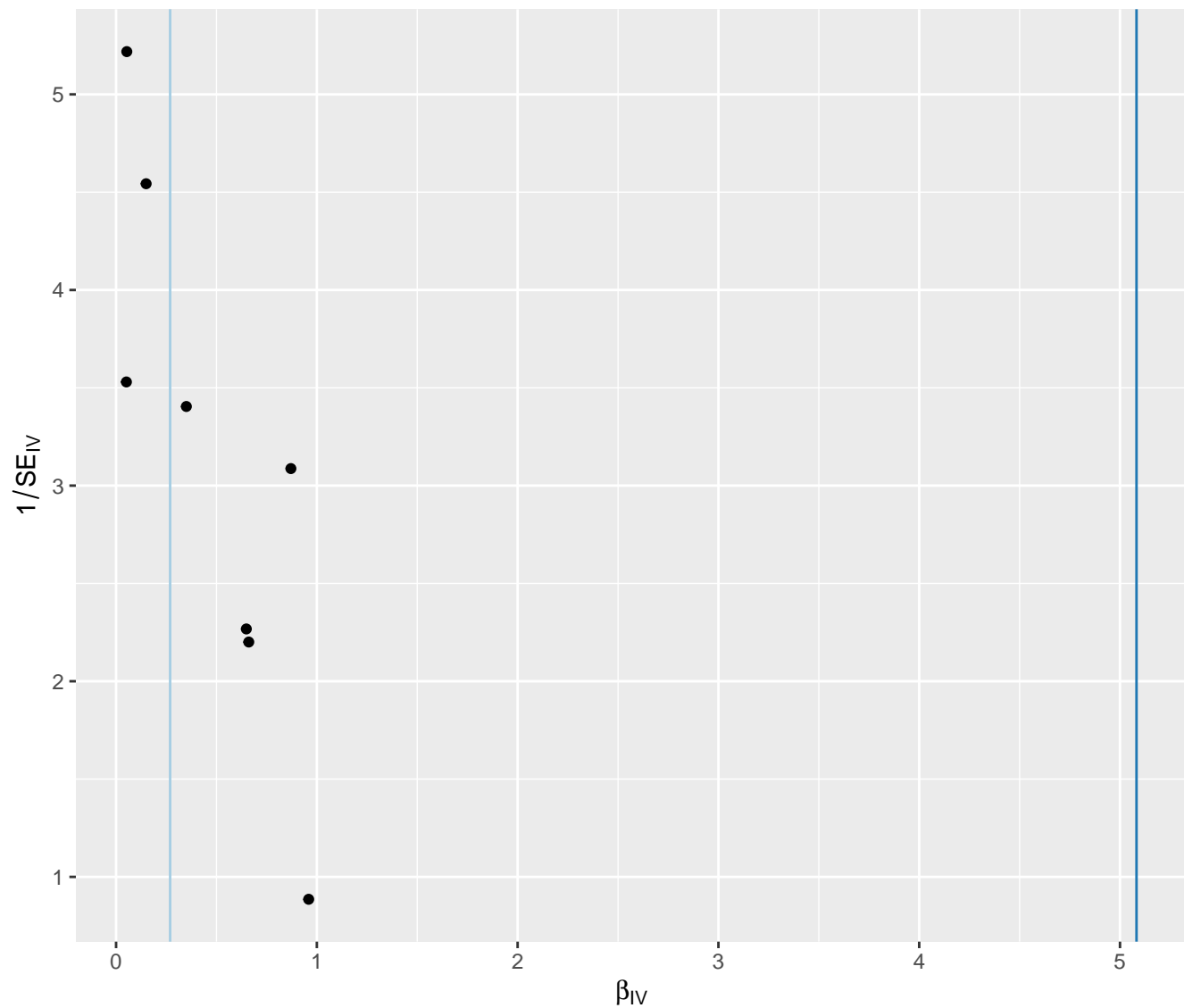

Supplement: Supplementary file 1 [file DataSheet1.ZIP › Supplementary Materials/MR plots for tongue/tongue═╝/Breast cancer/pheno.1918_to_breast cancer_funnel.pdf]

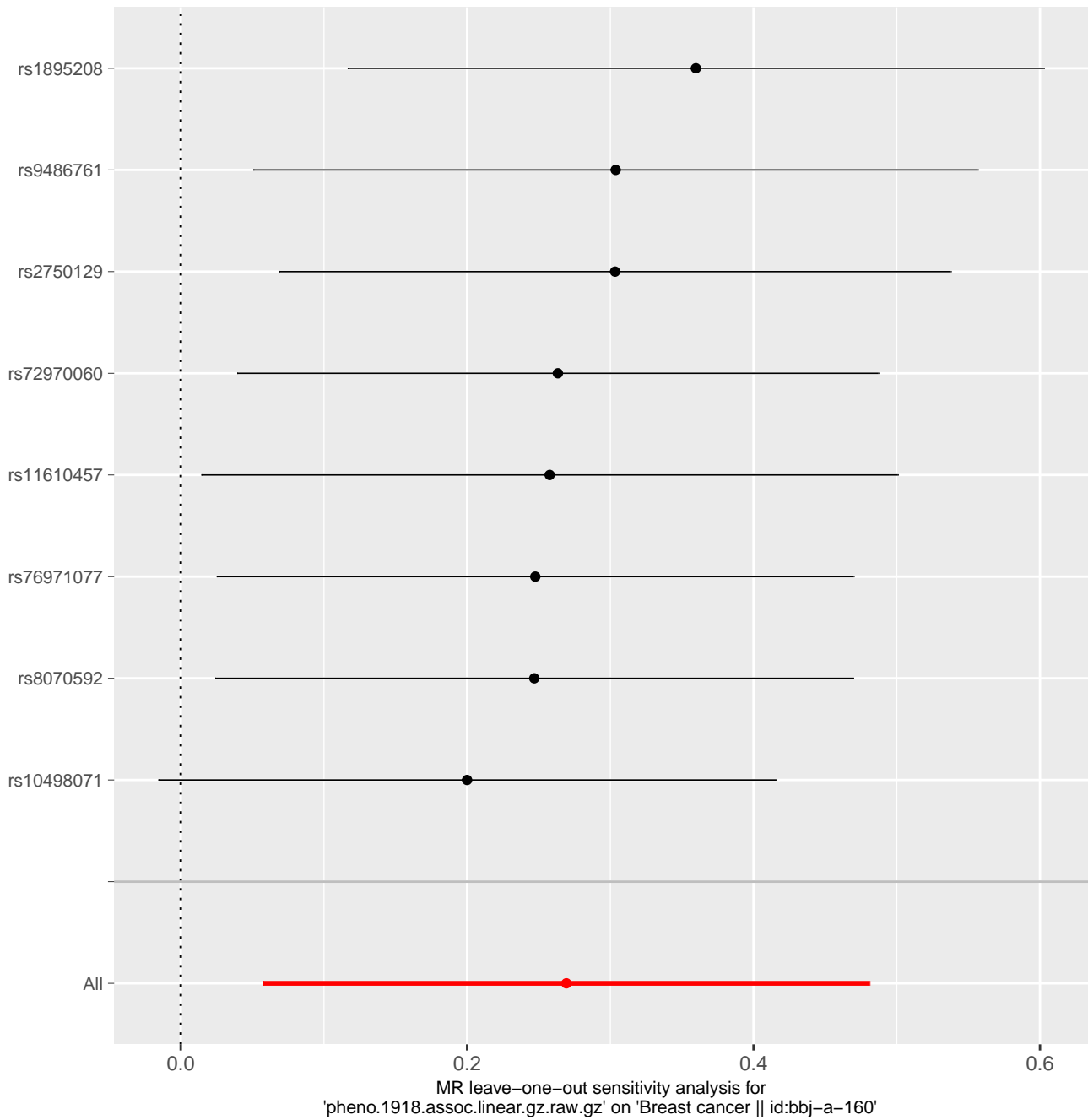

Supplement: Supplementary file 1 [file DataSheet1.ZIP › Supplementary Materials/MR plots for tongue/tongue═╝/Breast cancer/pheno.1918_to_breast cancer_leave_one_out.pdf]

# MR Test

- Inverse variance weighted
- MR Egger
- Simple mode
- Weighted median
- Weighted mode

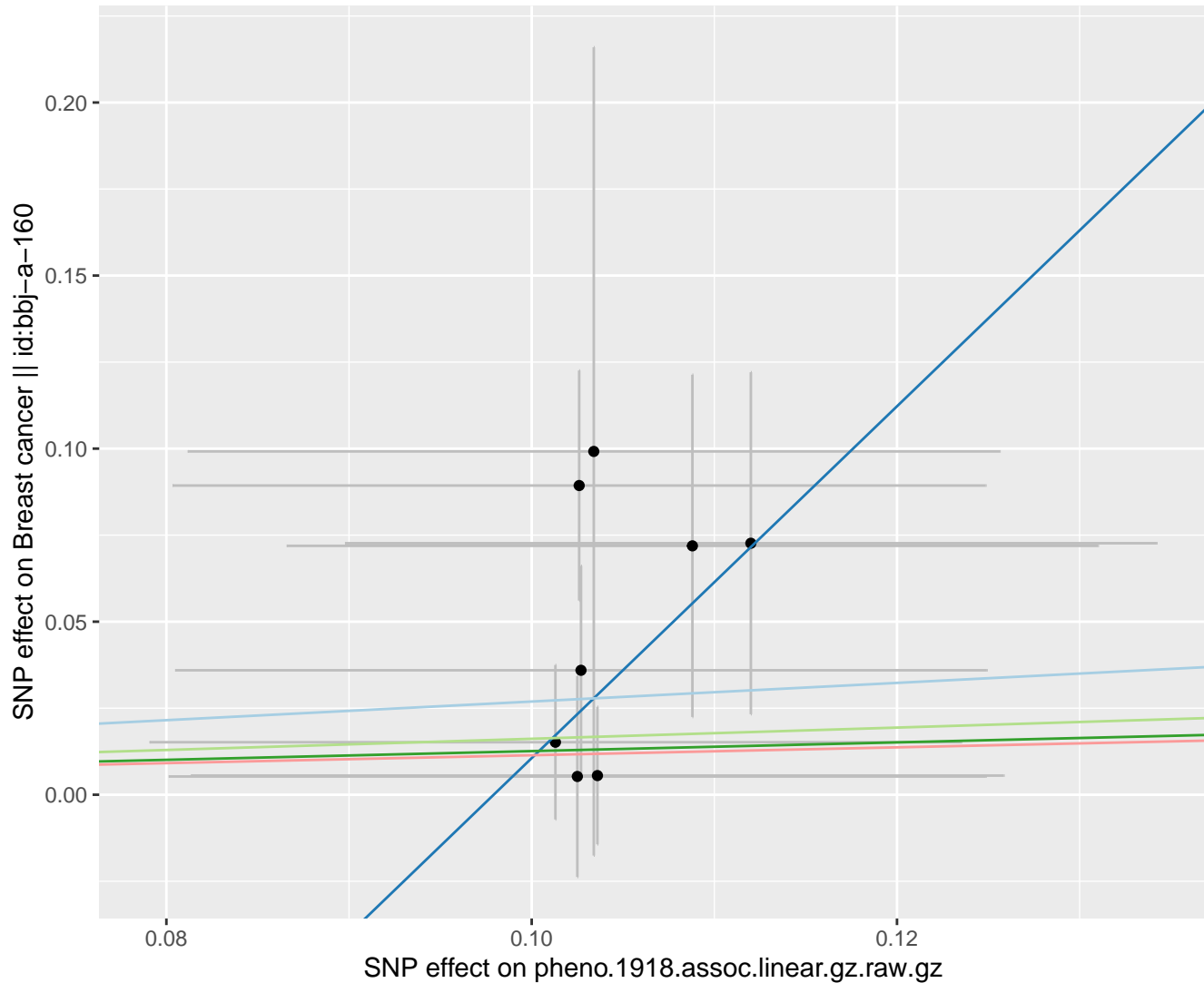

Supplement: Supplementary file 1 [file DataSheet1.ZIP › Supplementary Materials/MR plots for tongue/tongue═╝/Breast cancer/pheno.1918_to_breast cancer_scatter.pdf]

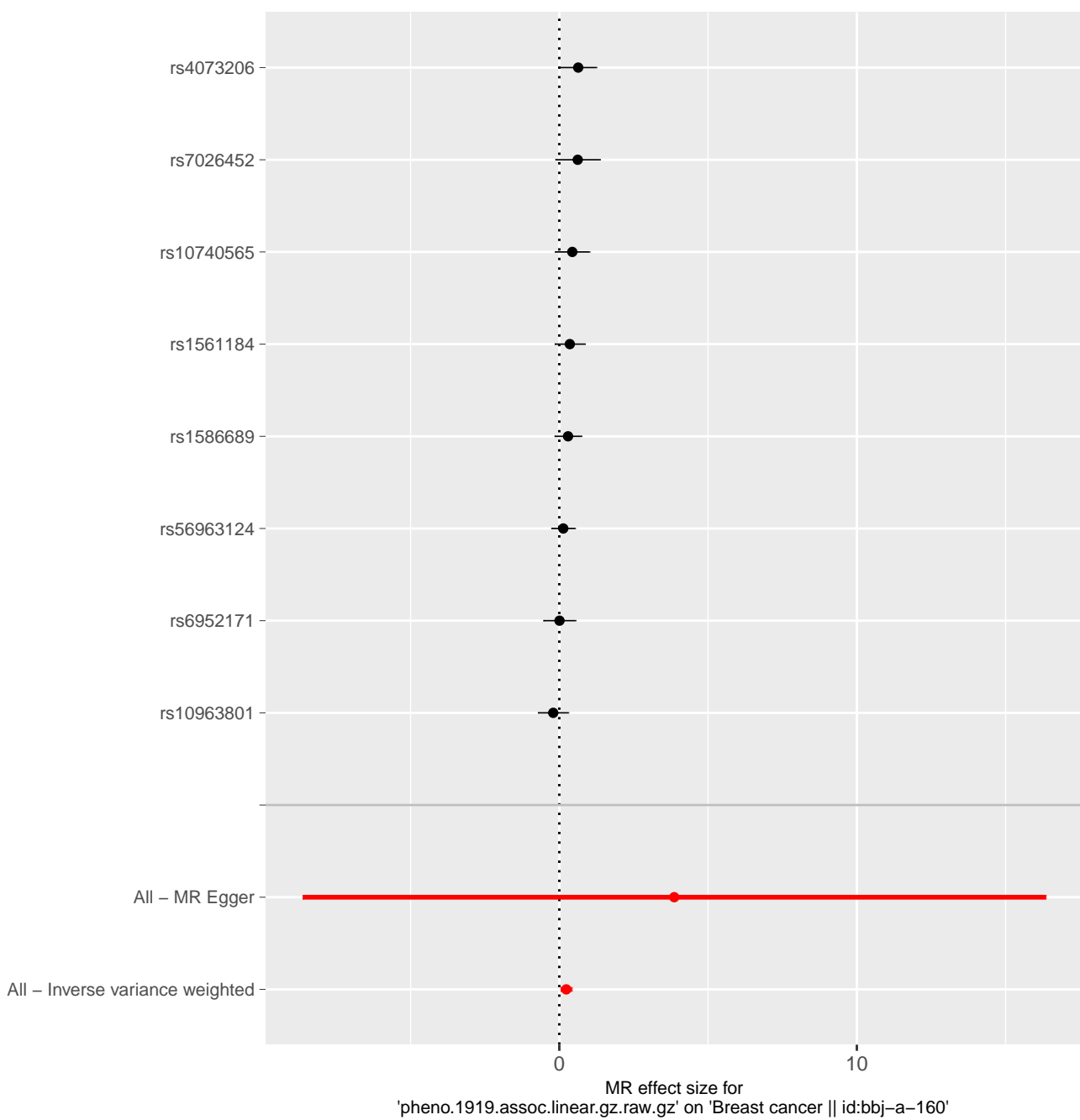

Supplement: Supplementary file 1 [file DataSheet1.ZIP › Supplementary Materials/MR plots for tongue/tongue═╝/Breast cancer/pheno.1919_to_breast cancer_forest.pdf]

# MR Method

Inverse variance weighted  
MR Egger

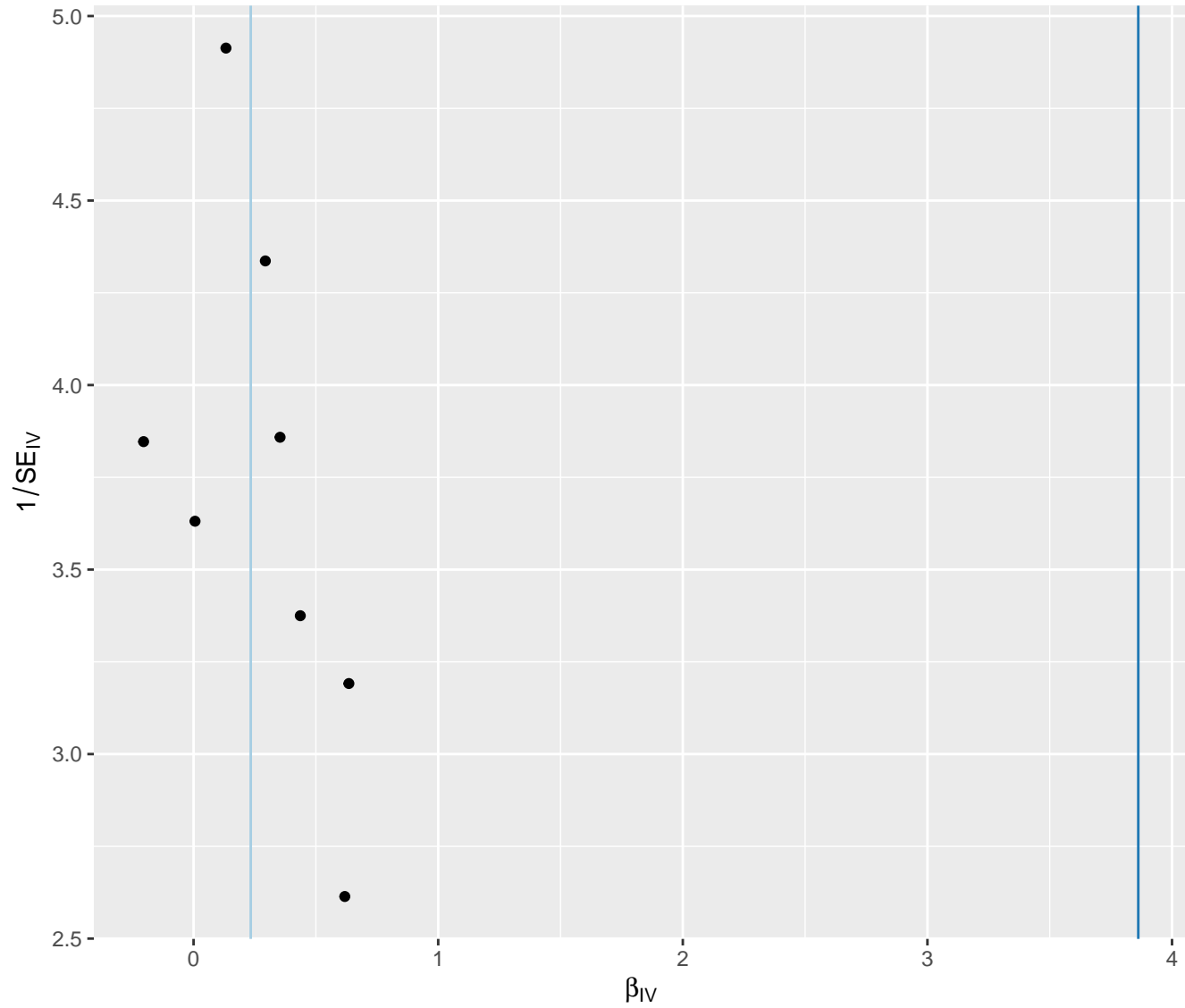

Supplement: Supplementary file 1 [file DataSheet1.ZIP › Supplementary Materials/MR plots for tongue/tongue═╝/Breast cancer/pheno.1919_to_breast cancer_funnel.pdf]

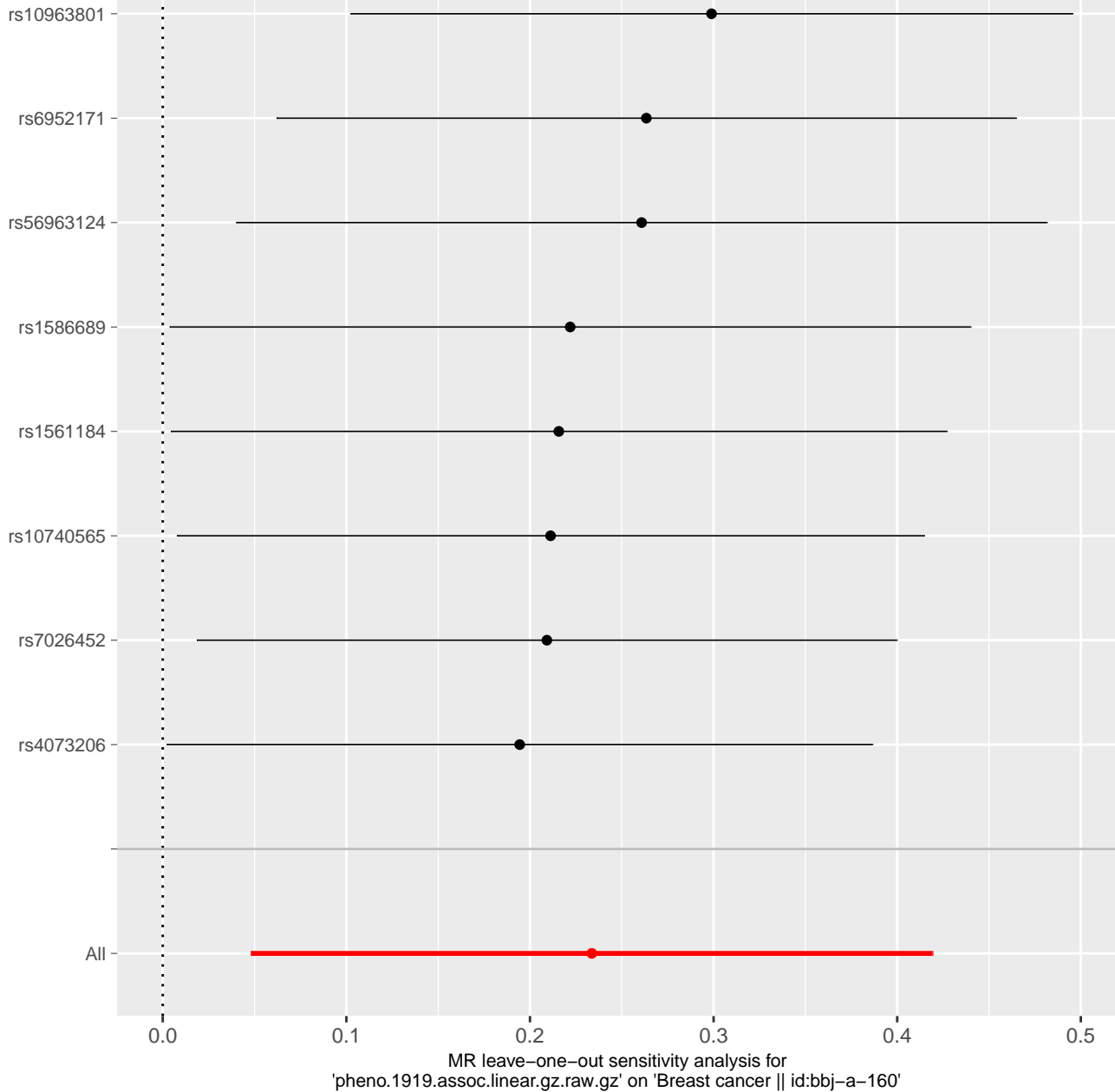

Supplement: Supplementary file 1 [file DataSheet1.ZIP › Supplementary Materials/MR plots for tongue/tongue═╝/Breast cancer/pheno.1919_to_breast cancer_leave_one_out.pdf]

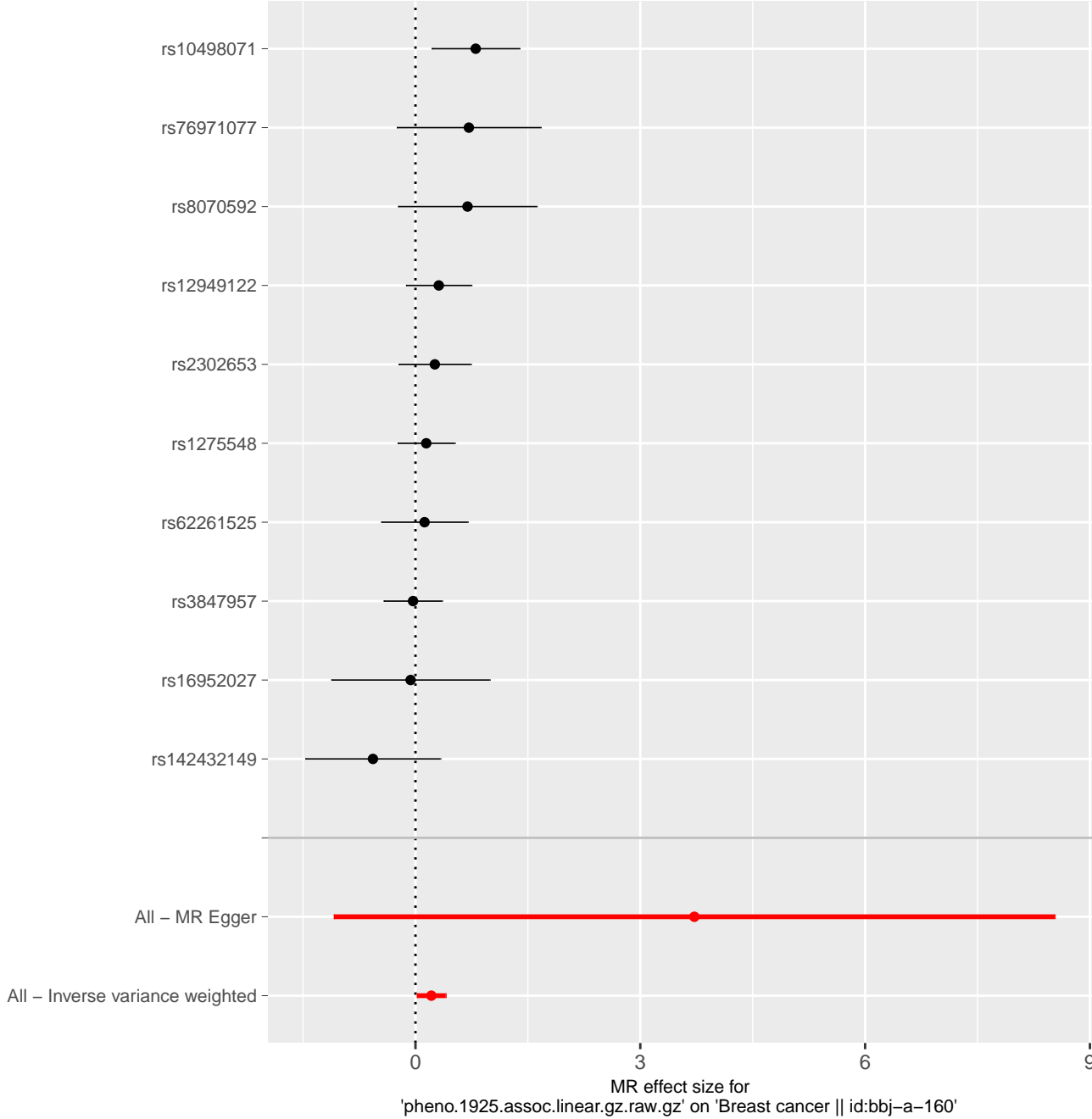

Supplement: Supplementary file 1 [file DataSheet1.ZIP › Supplementary Materials/MR plots for tongue/tongue═╝/Breast cancer/pheno.1925_to_breast cancer_forest.pdf]

# MR Method

- Inverse variance weighted
- MR Egger

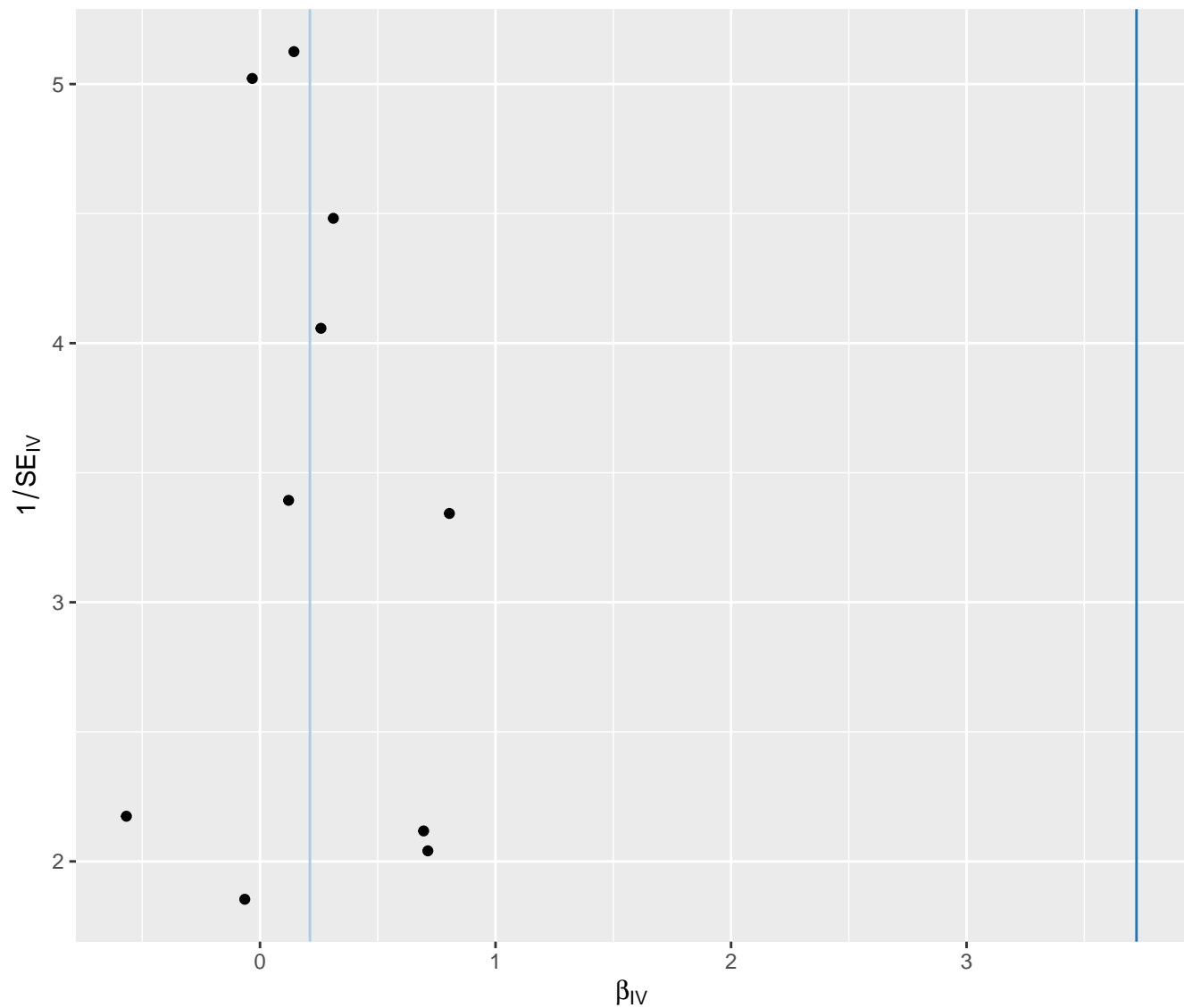

Supplement: Supplementary file 1 [file DataSheet1.ZIP › Supplementary Materials/MR plots for tongue/tongue═╝/Breast cancer/pheno.1925_to_breast cancer_funnel.pdf]

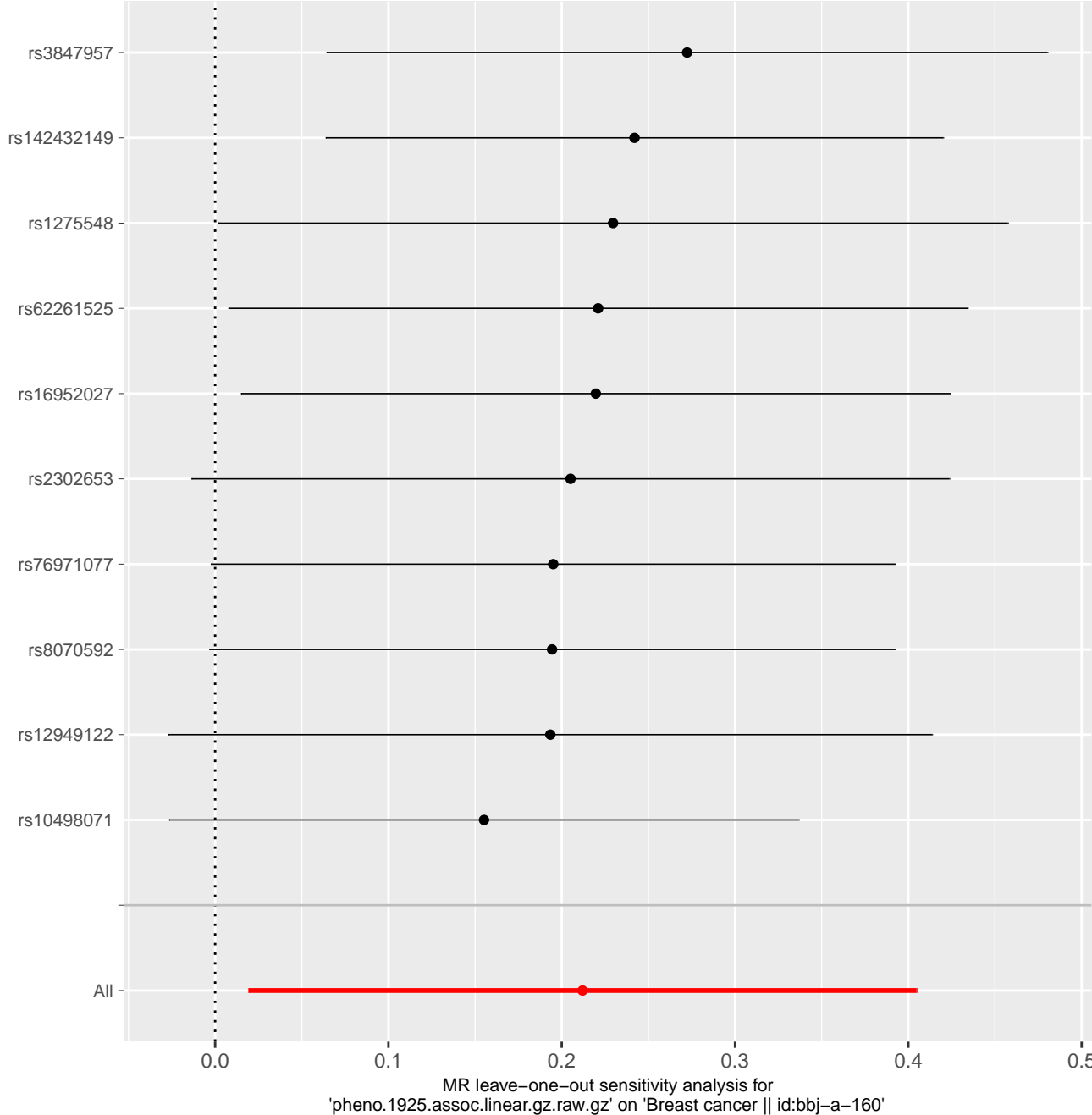

Supplement: Supplementary file 1 [file DataSheet1.ZIP › Supplementary Materials/MR plots for tongue/tongue═╝/Breast cancer/pheno.1925_to_breast cancer_leave_one_out.pdf]

# MR Test

- Inverse variance weighted
- MR Egger
- Simple mode
- Weighted median
- Weighted mode

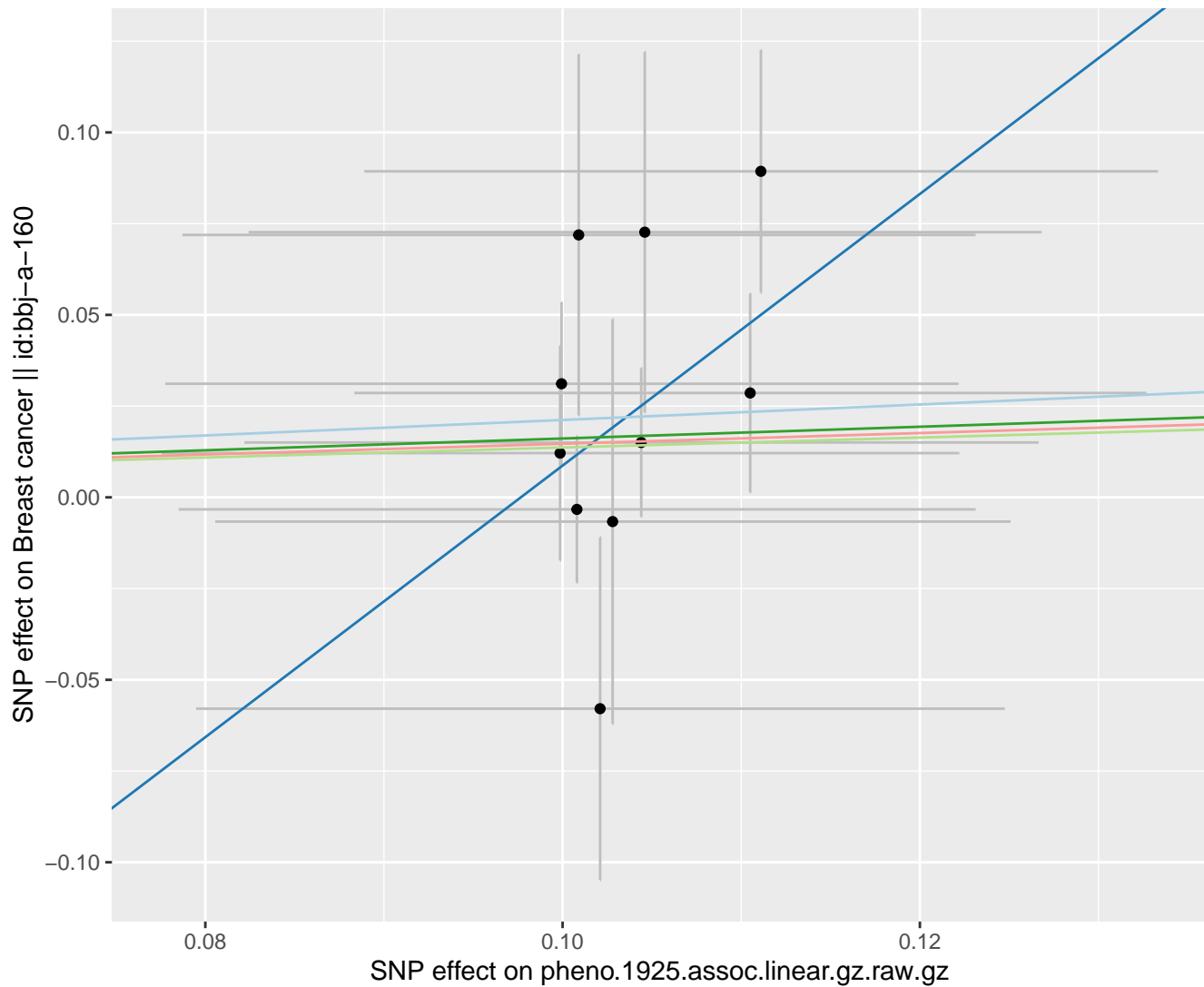

Supplement: Supplementary file 1 [file DataSheet1.ZIP › Supplementary Materials/MR plots for tongue/tongue═╝/Breast cancer/pheno.1925_to_breast cancer_scatter.pdf]

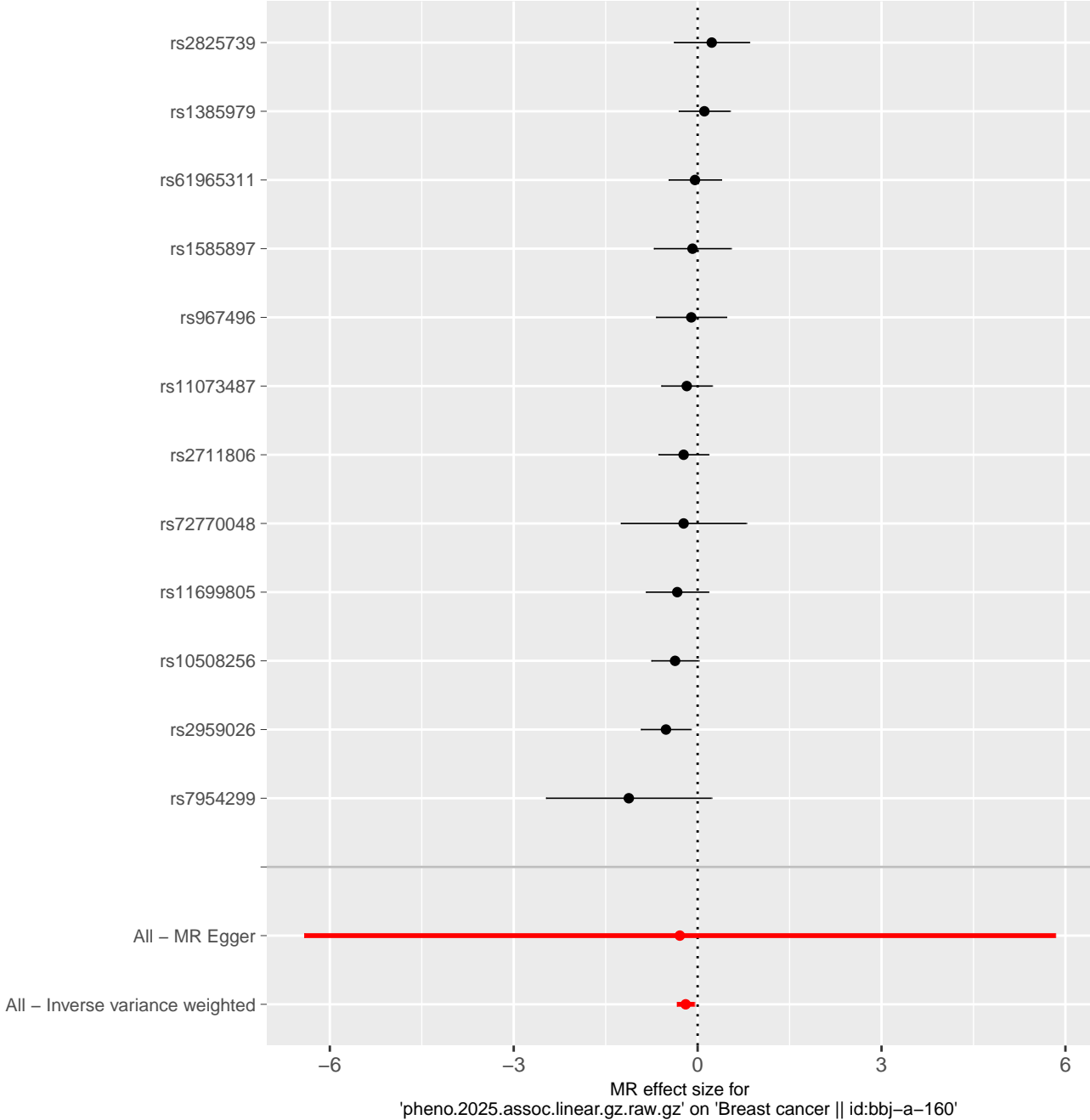

Supplement: Supplementary file 1 [file DataSheet1.ZIP › Supplementary Materials/MR plots for tongue/tongue═╝/Breast cancer/pheno.2025_to_breast cancer_forest.pdf]

# MR Method

- Inverse variance weighted
- MR Egger

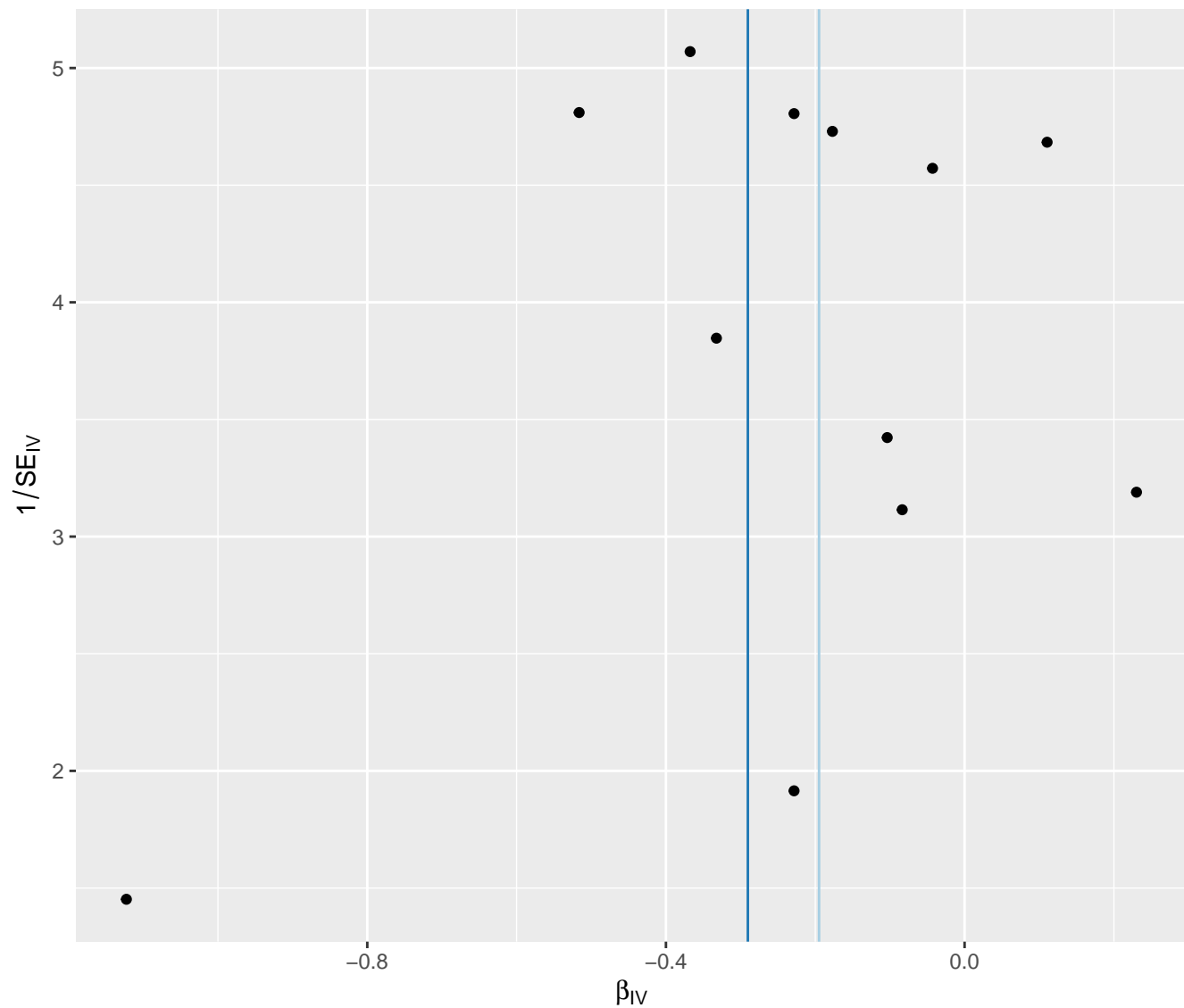

Supplement: Supplementary file 1 [file DataSheet1.ZIP › Supplementary Materials/MR plots for tongue/tongue═╝/Breast cancer/pheno.2025_to_breast cancer_funnel.pdf]

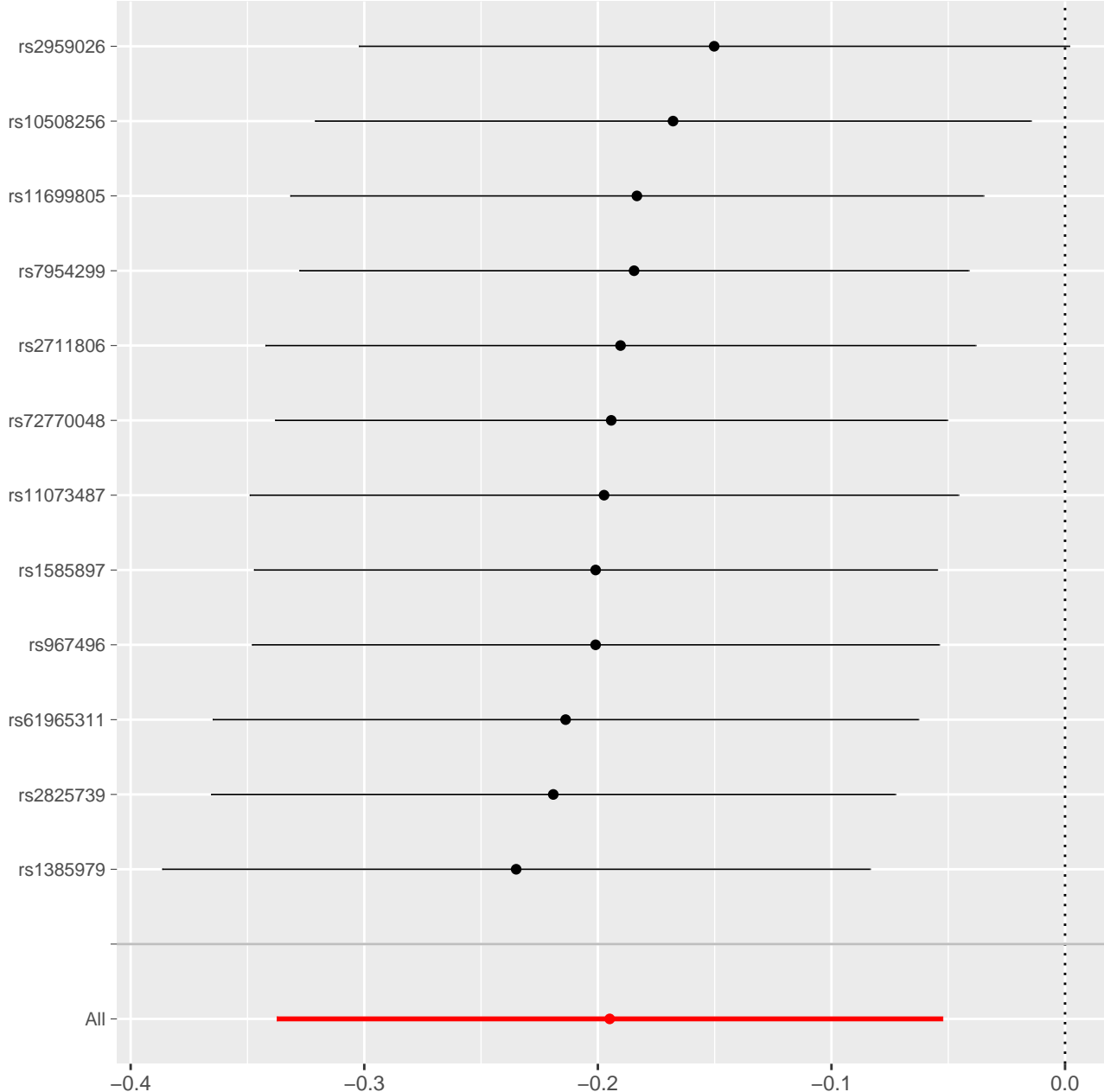

Supplement: Supplementary file 1 [file DataSheet1.ZIP › Supplementary Materials/MR plots for tongue/tongue═╝/Breast cancer/pheno.2025_to_breast cancer_leave_one_out.pdf]

# MR Test

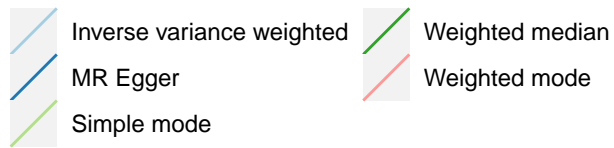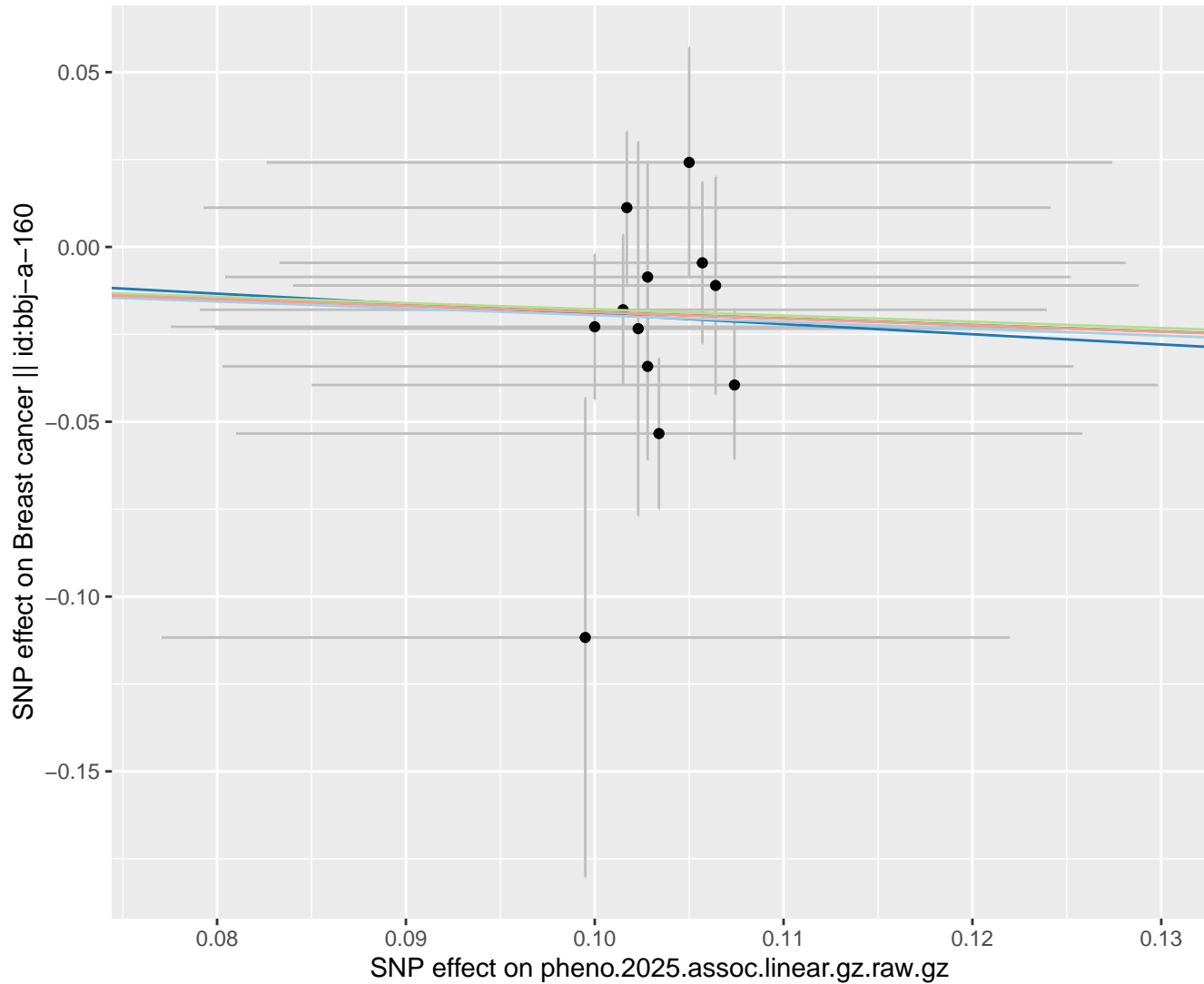

Supplement: Supplementary file 1 [file DataSheet1.ZIP › Supplementary Materials/MR plots for tongue/tongue═╝/Breast cancer/pheno.2025_to_breast cancer_scatter.pdf]

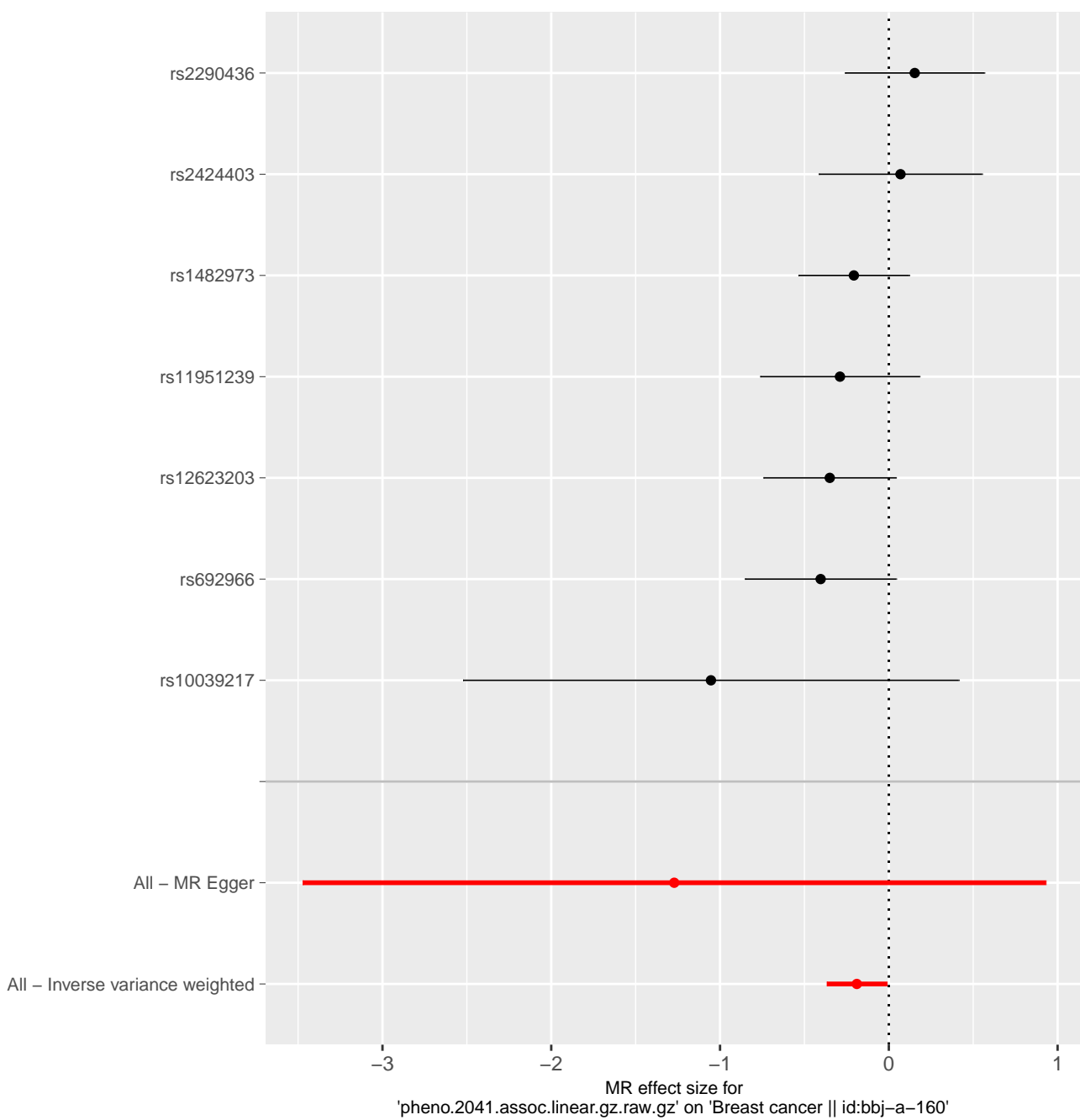

Supplement: Supplementary file 1 [file DataSheet1.ZIP › Supplementary Materials/MR plots for tongue/tongue═╝/Breast cancer/pheno.2041_to_breast cancer_forest.pdf]

# MR Method

- Inverse variance weighted
- MR Egger

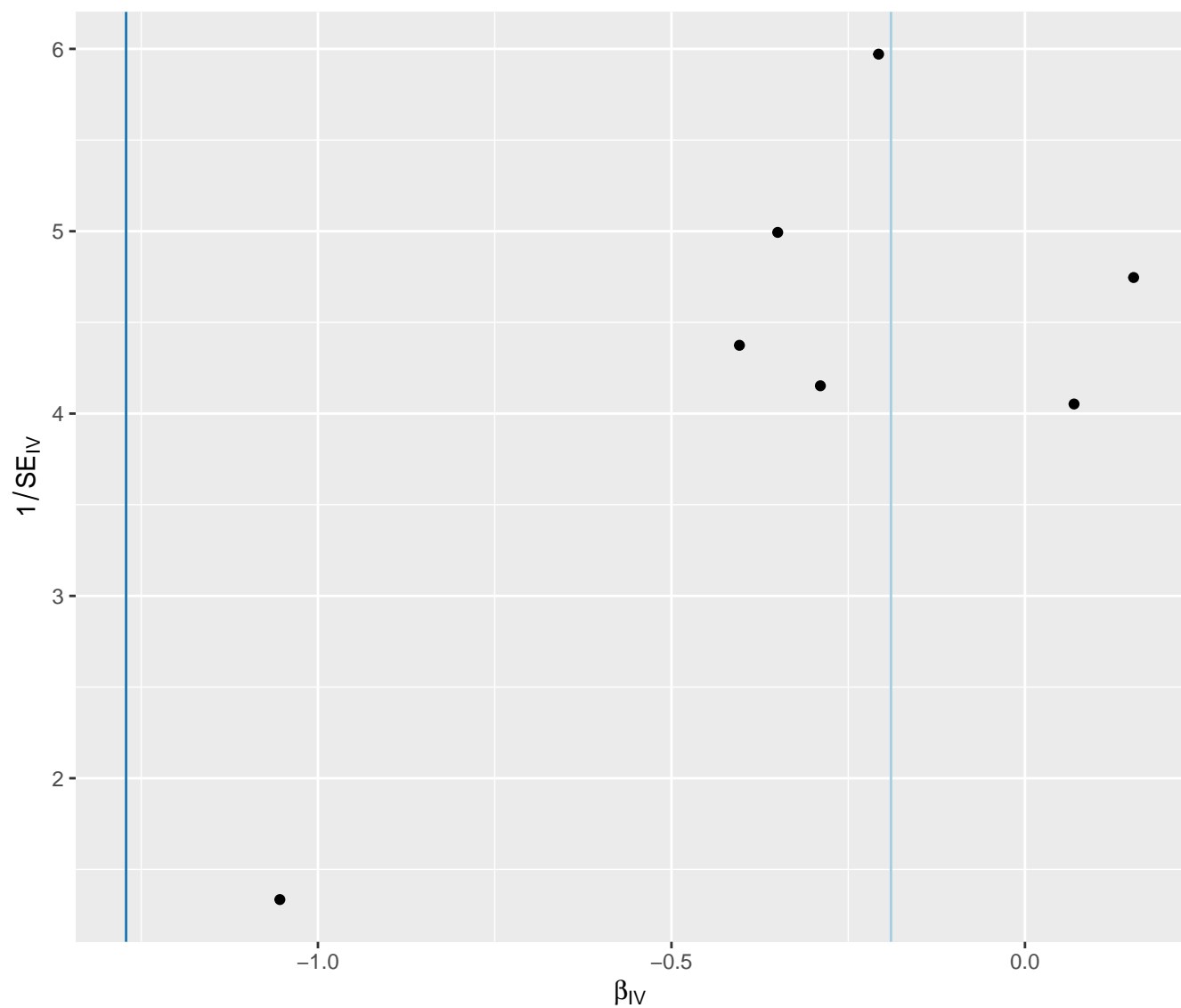

Supplement: Supplementary file 1 [file DataSheet1.ZIP › Supplementary Materials/MR plots for tongue/tongue═╝/Breast cancer/pheno.2041_to_breast cancer_funnel.pdf]

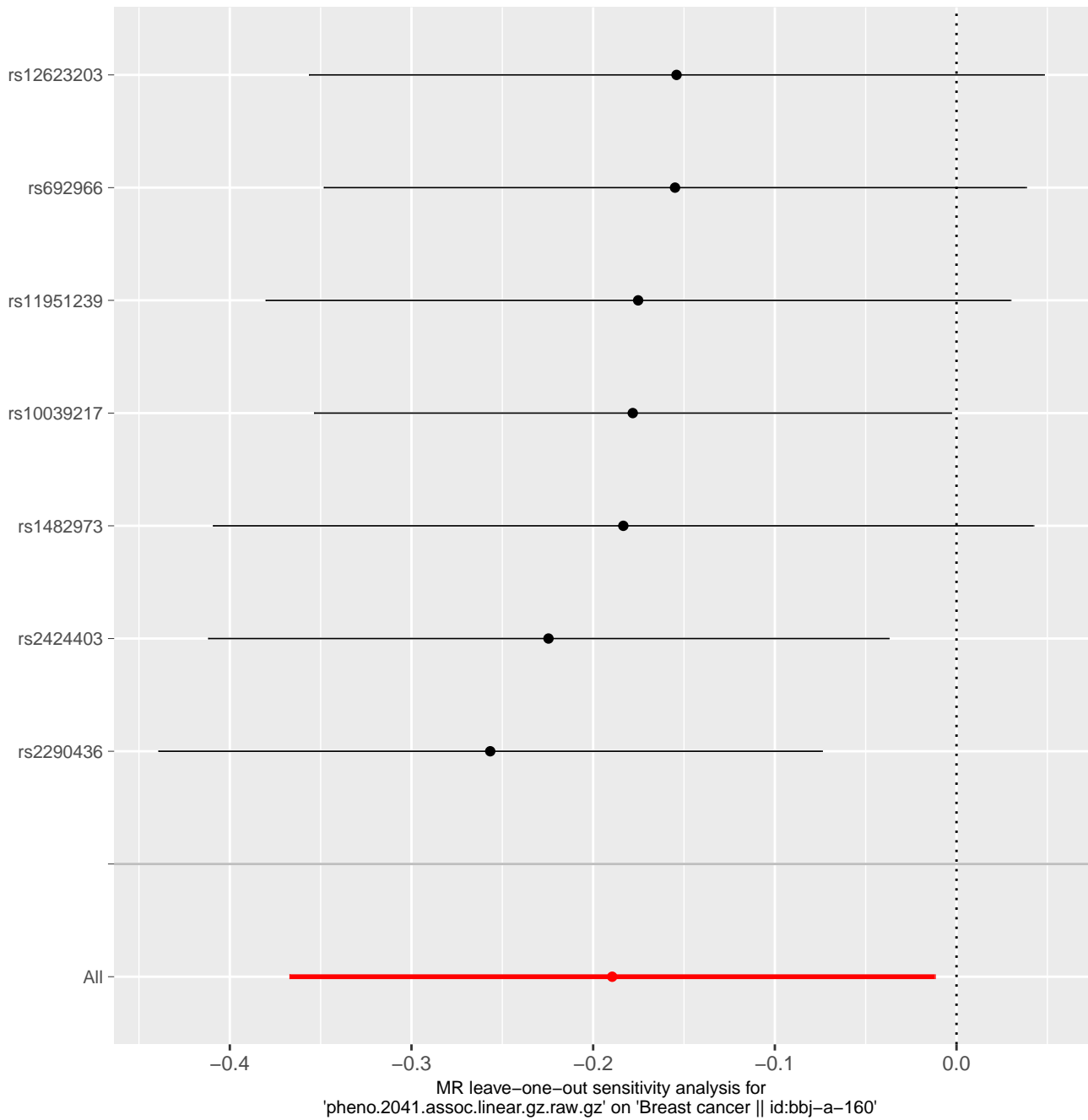

Supplement: Supplementary file 1 [file DataSheet1.ZIP › Supplementary Materials/MR plots for tongue/tongue═╝/Breast cancer/pheno.2041_to_breast cancer_leave_one_out.pdf]

# MR Test

- Inverse variance weighted
- MR Egger
- Simple mode
- Weighted median
- Weighted mode

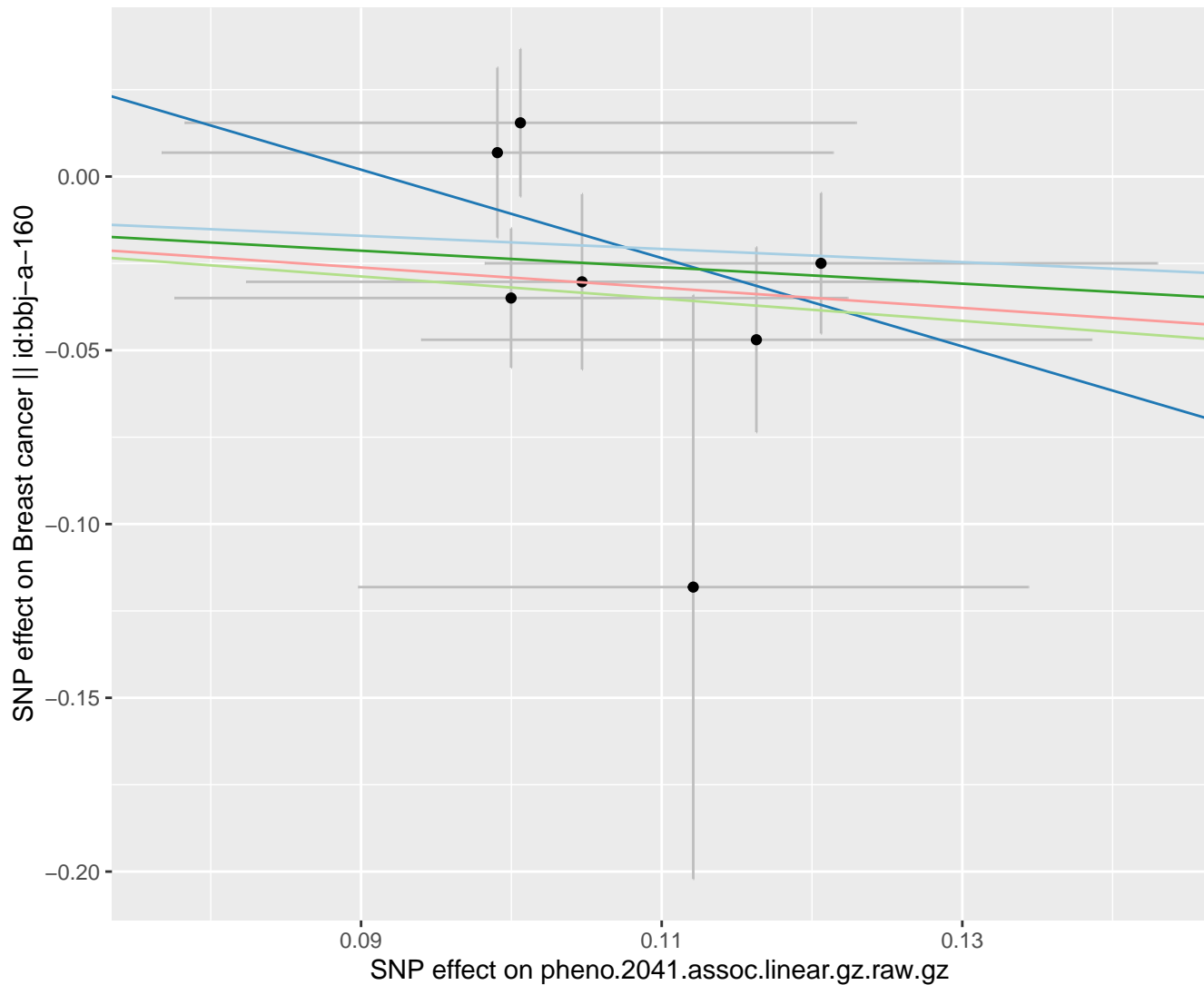

Supplement: Supplementary file 1 [file DataSheet1.ZIP › Supplementary Materials/MR plots for tongue/tongue═╝/Breast cancer/pheno.2041_to_breast cancer_scatter.pdf]

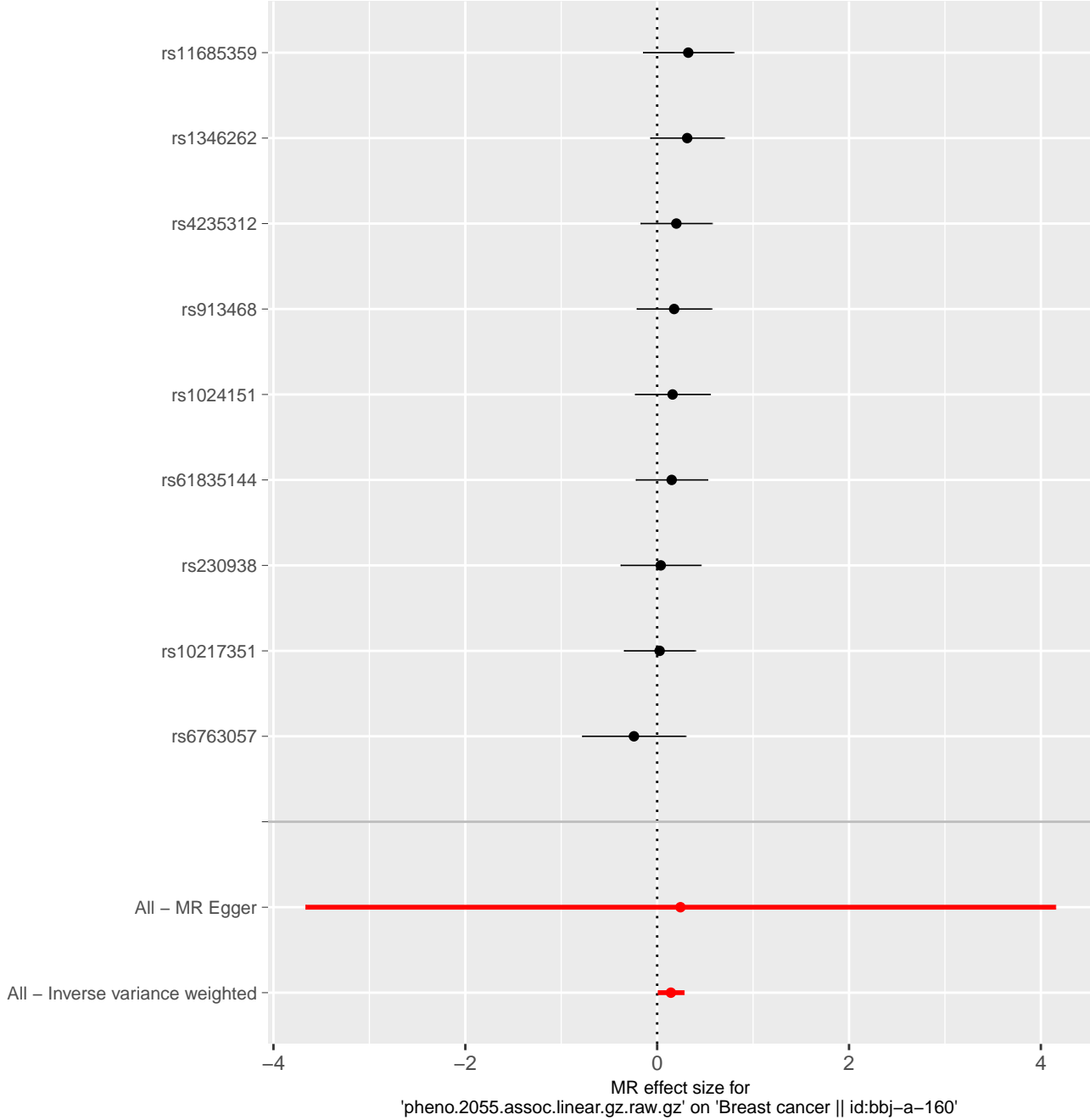

Supplement: Supplementary file 1 [file DataSheet1.ZIP › Supplementary Materials/MR plots for tongue/tongue═╝/Breast cancer/pheno.2055_to_breast cancer_forest.pdf]

# MR Method

Inverse variance weighted

MR Egger

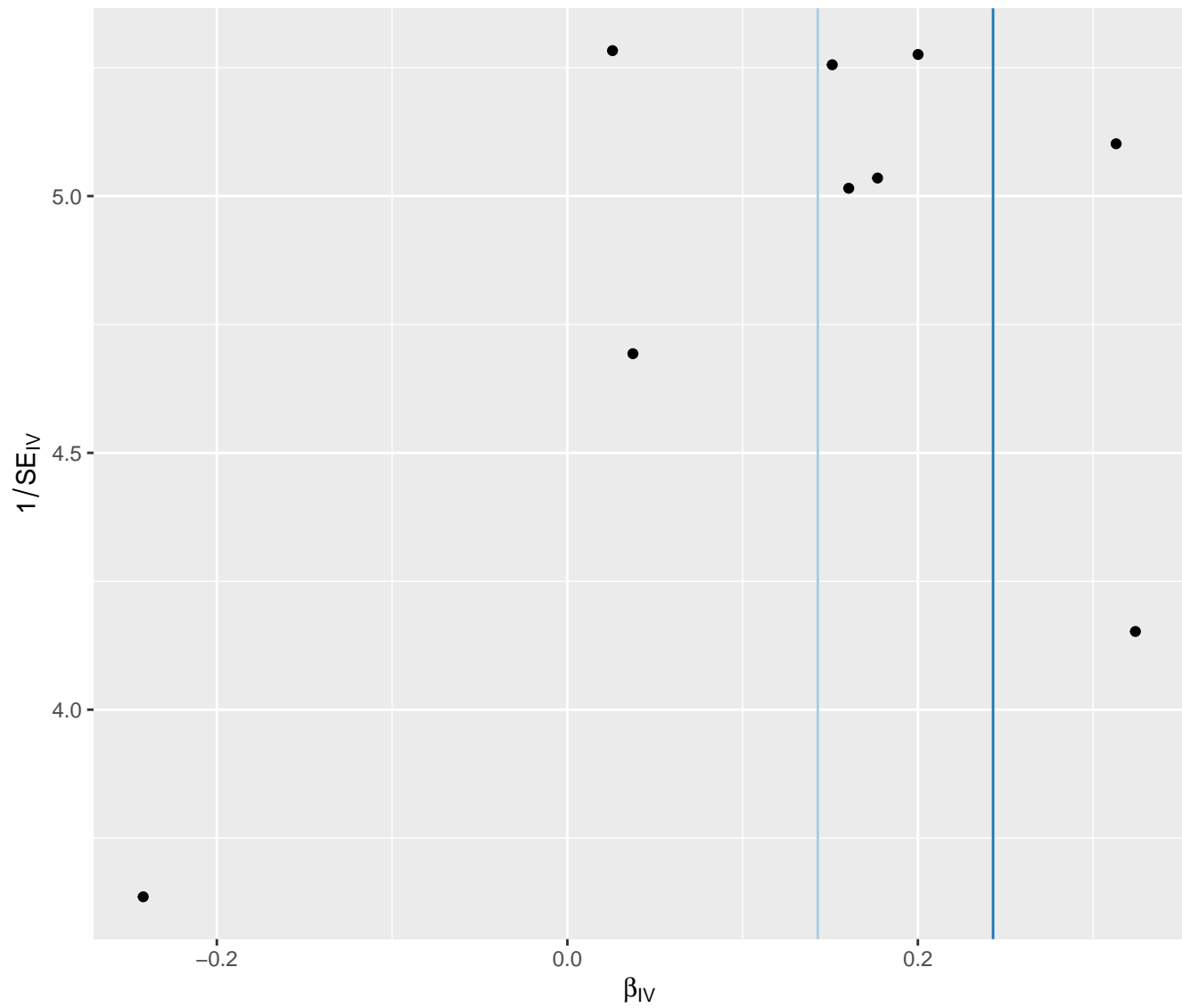

Supplement: Supplementary file 1 [file DataSheet1.ZIP › Supplementary Materials/MR plots for tongue/tongue═╝/Breast cancer/pheno.2055_to_breast cancer_funnel.pdf]

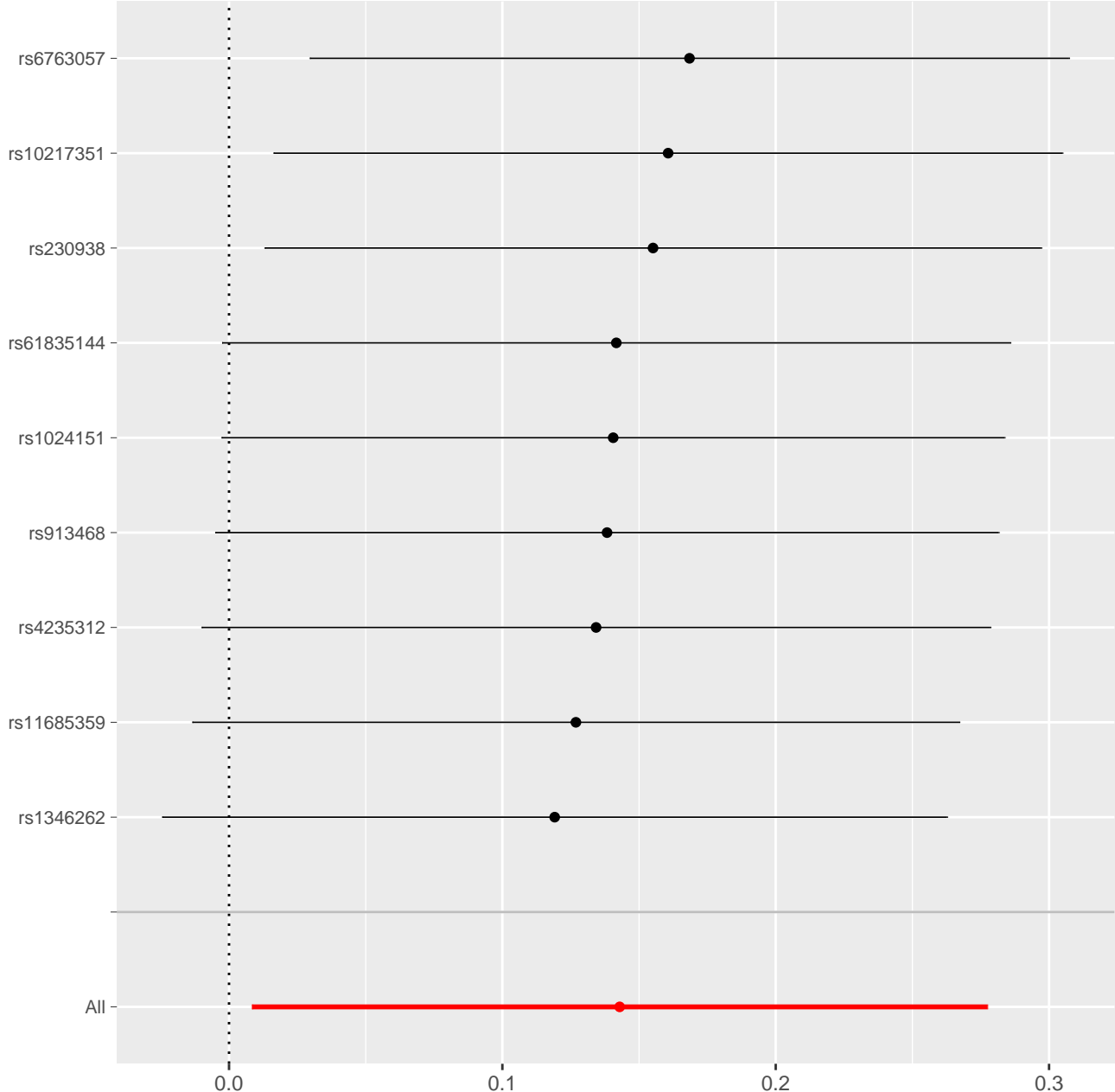

MR leave-one-out sensitivity analysis for  
'pheno.2055.assoc.linear.gz.raw.gz' on 'Breast cancer || id:bbj-a-160'

Supplement: Supplementary file 1 [file DataSheet1.ZIP › Supplementary Materials/MR plots for tongue/tongue═╝/Breast cancer/pheno.2055_to_breast cancer_leave_one_out.pdf]

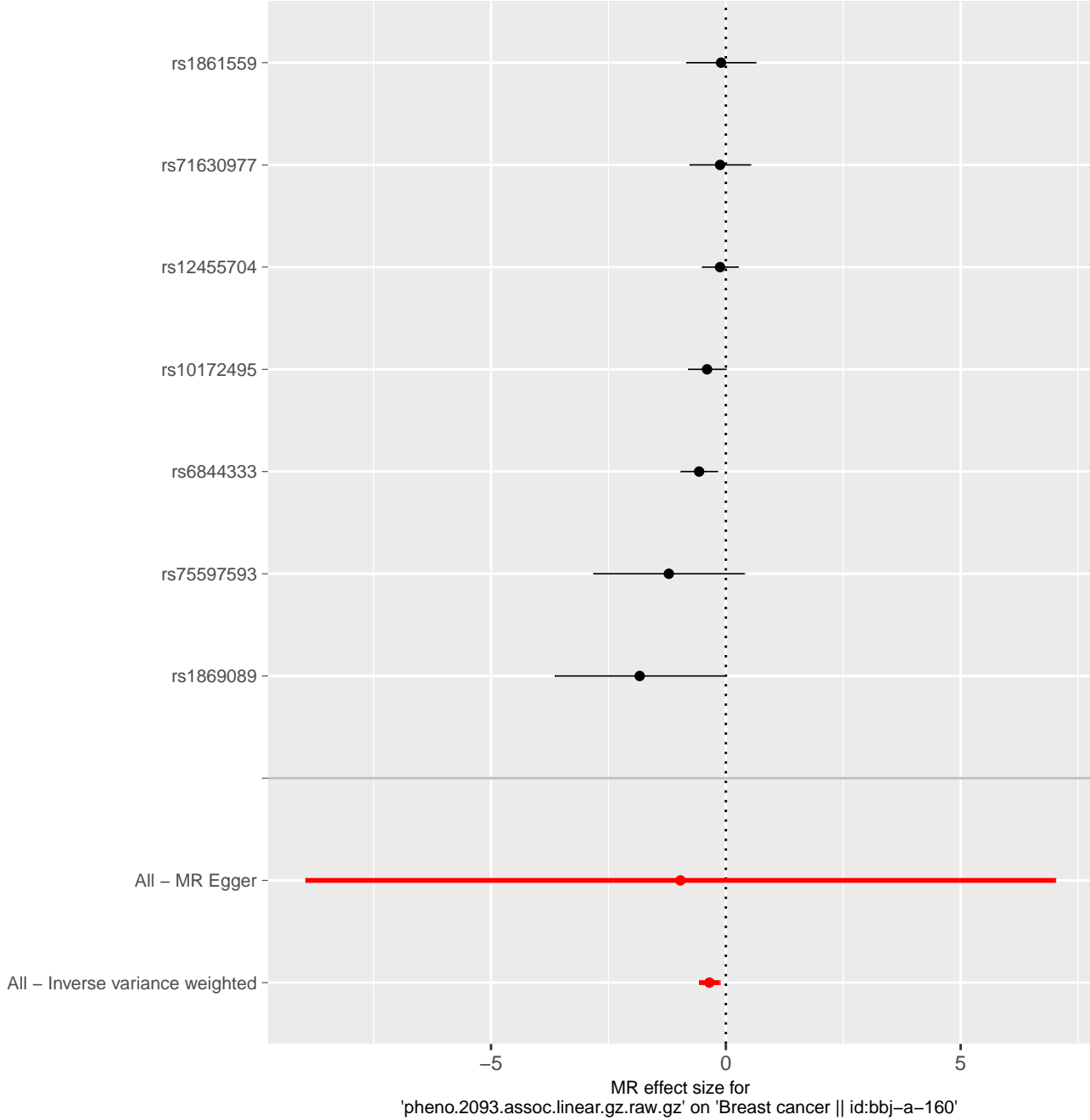

Supplement: Supplementary file 1 [file DataSheet1.ZIP › Supplementary Materials/MR plots for tongue/tongue═╝/Breast cancer/pheno.2093_to_breast cancer_forest.pdf]

# MR Method

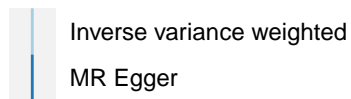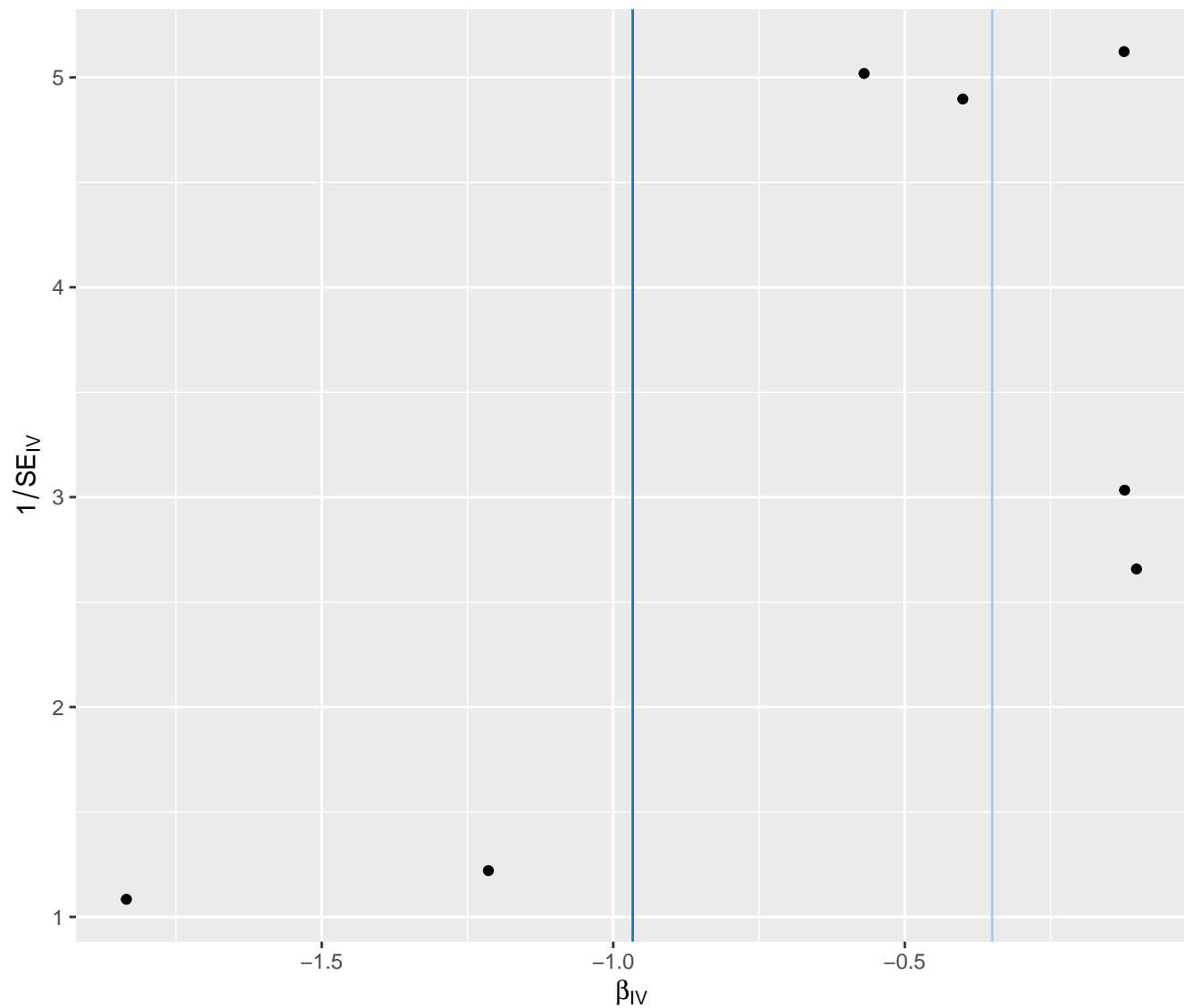

Supplement: Supplementary file 1 [file DataSheet1.ZIP › Supplementary Materials/MR plots for tongue/tongue═╝/Breast cancer/pheno.2093_to_breast cancer_funnel.pdf]

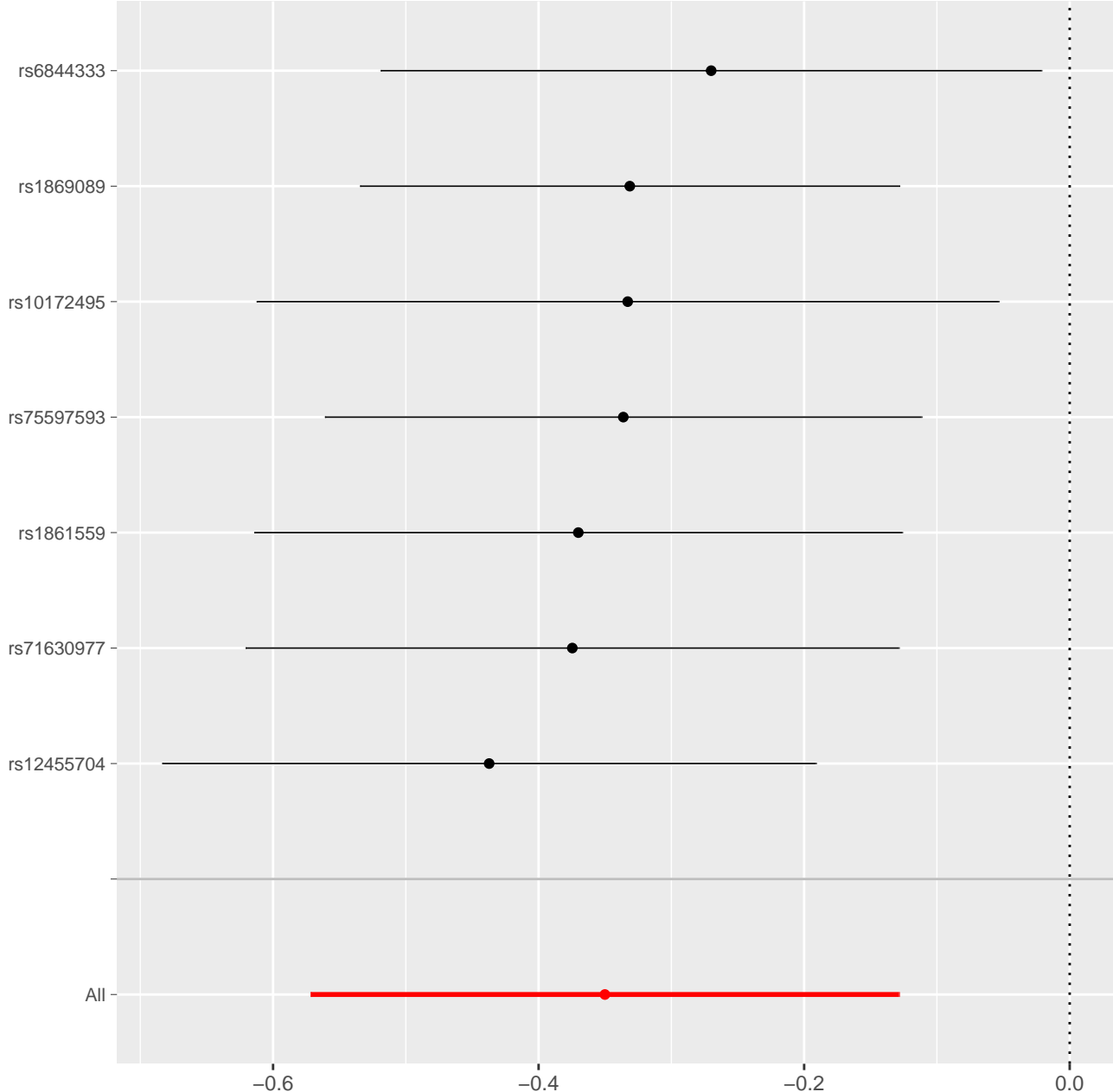

MR leave-one-out sensitivity analysis for  
'pheno.2093.assoc.linear.gz.raw.gz' on 'Breast cancer || id:bbj-a-160'

Supplement: Supplementary file 1 [file DataSheet1.ZIP › Supplementary Materials/MR plots for tongue/tongue═╝/Breast cancer/pheno.2093_to_breast cancer_leave_one_out.pdf]

# MR Test

- Inverse variance weighted
- MR Egger
- Simple mode
- Weighted median
- Weighted mode

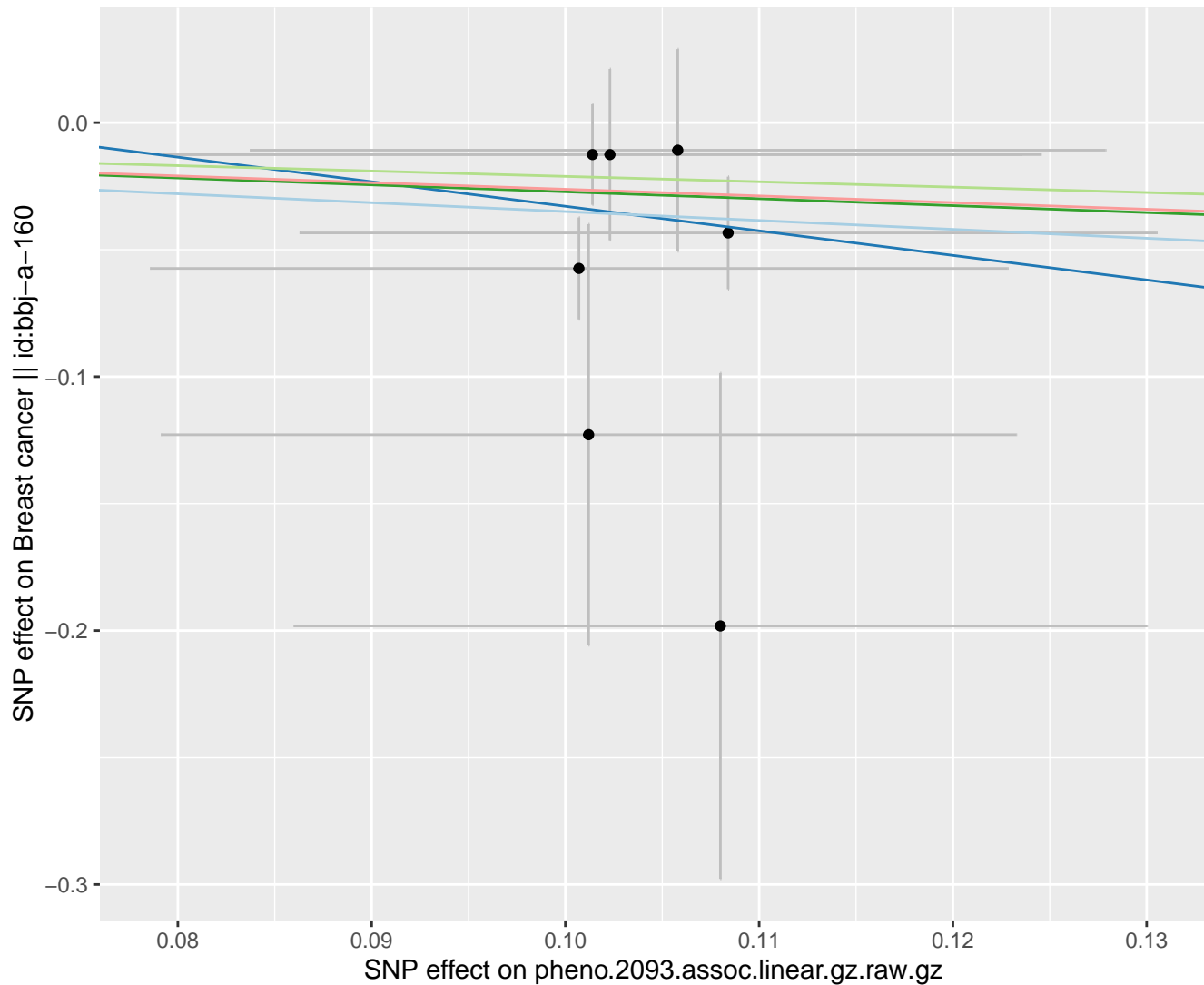

Supplement: Supplementary file 1 [file DataSheet1.ZIP › Supplementary Materials/MR plots for tongue/tongue═╝/Breast cancer/pheno.2093_to_breast cancer_scatter.pdf]

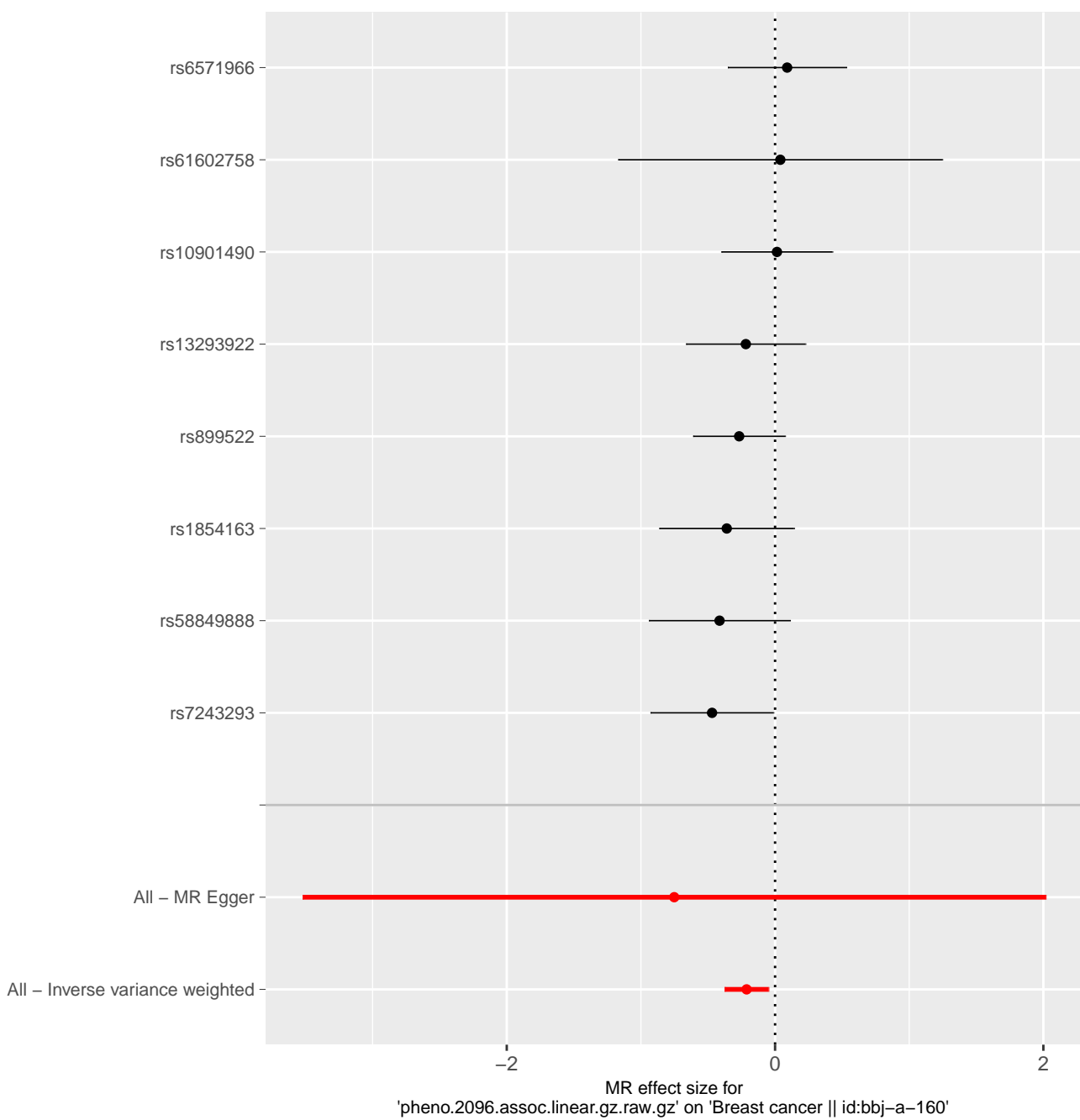

Supplement: Supplementary file 1 [file DataSheet1.ZIP › Supplementary Materials/MR plots for tongue/tongue═╝/Breast cancer/pheno.2096_to_breast cancer_forest.pdf]

# MR Method

- Inverse variance weighted
- MR Egger

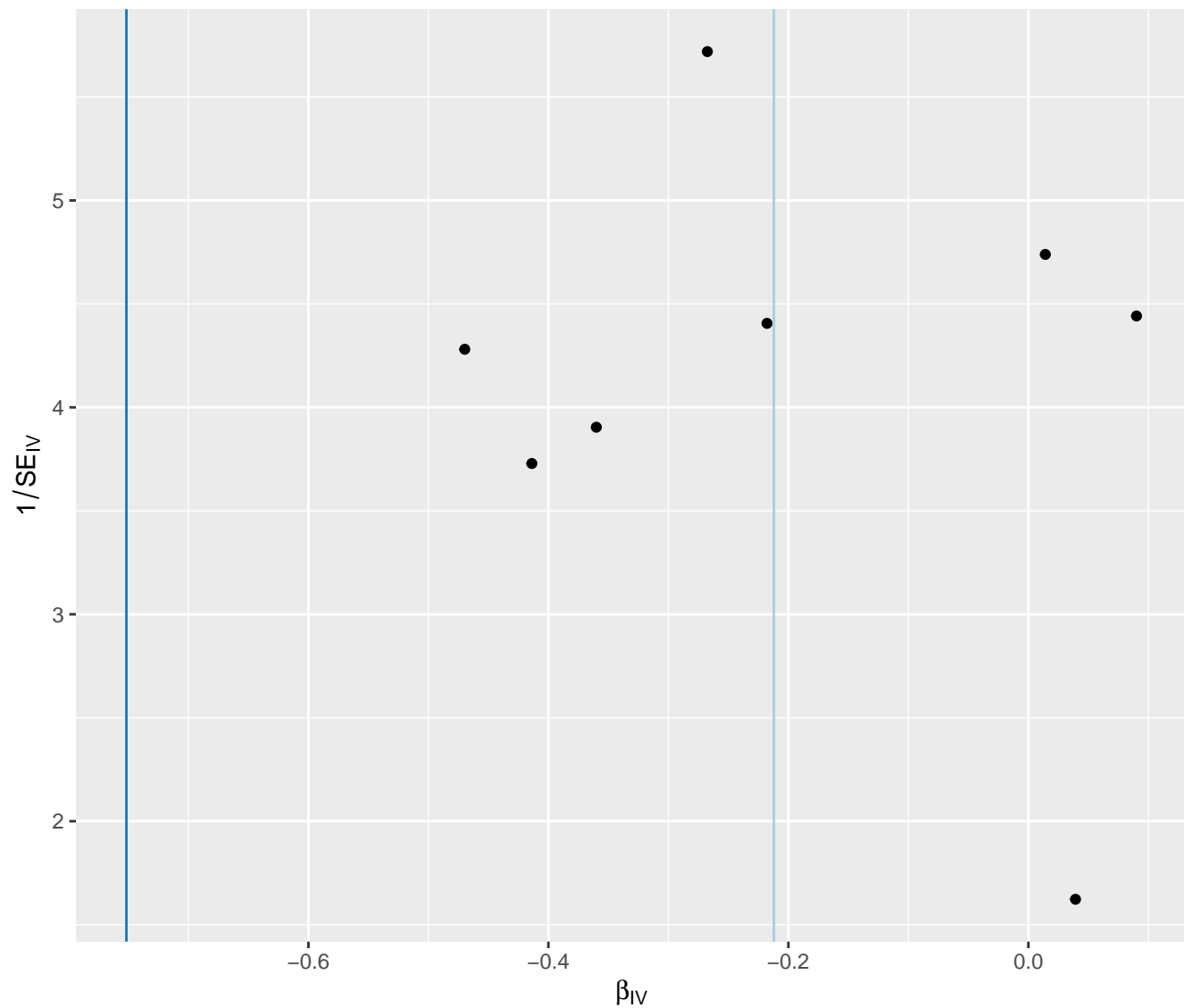

Supplement: Supplementary file 1 [file DataSheet1.ZIP › Supplementary Materials/MR plots for tongue/tongue═╝/Breast cancer/pheno.2096_to_breast cancer_funnel.pdf]

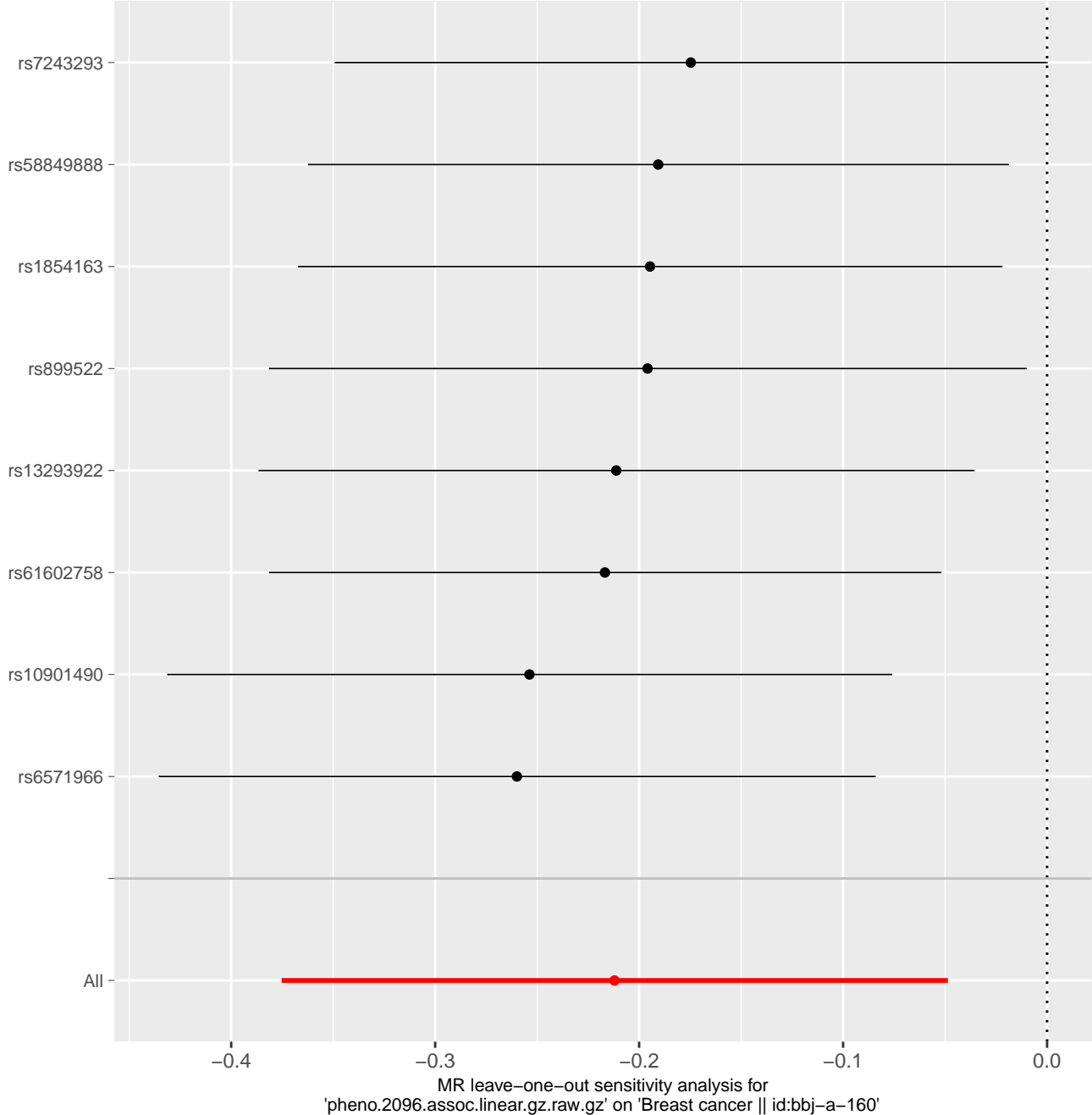

Supplement: Supplementary file 1 [file DataSheet1.ZIP › Supplementary Materials/MR plots for tongue/tongue═╝/Breast cancer/pheno.2096_to_breast cancer_leave_one_out.pdf]

# MR Test

- Inverse variance weighted
- MR Egger
- Simple mode
- Weighted median
- Weighted mode

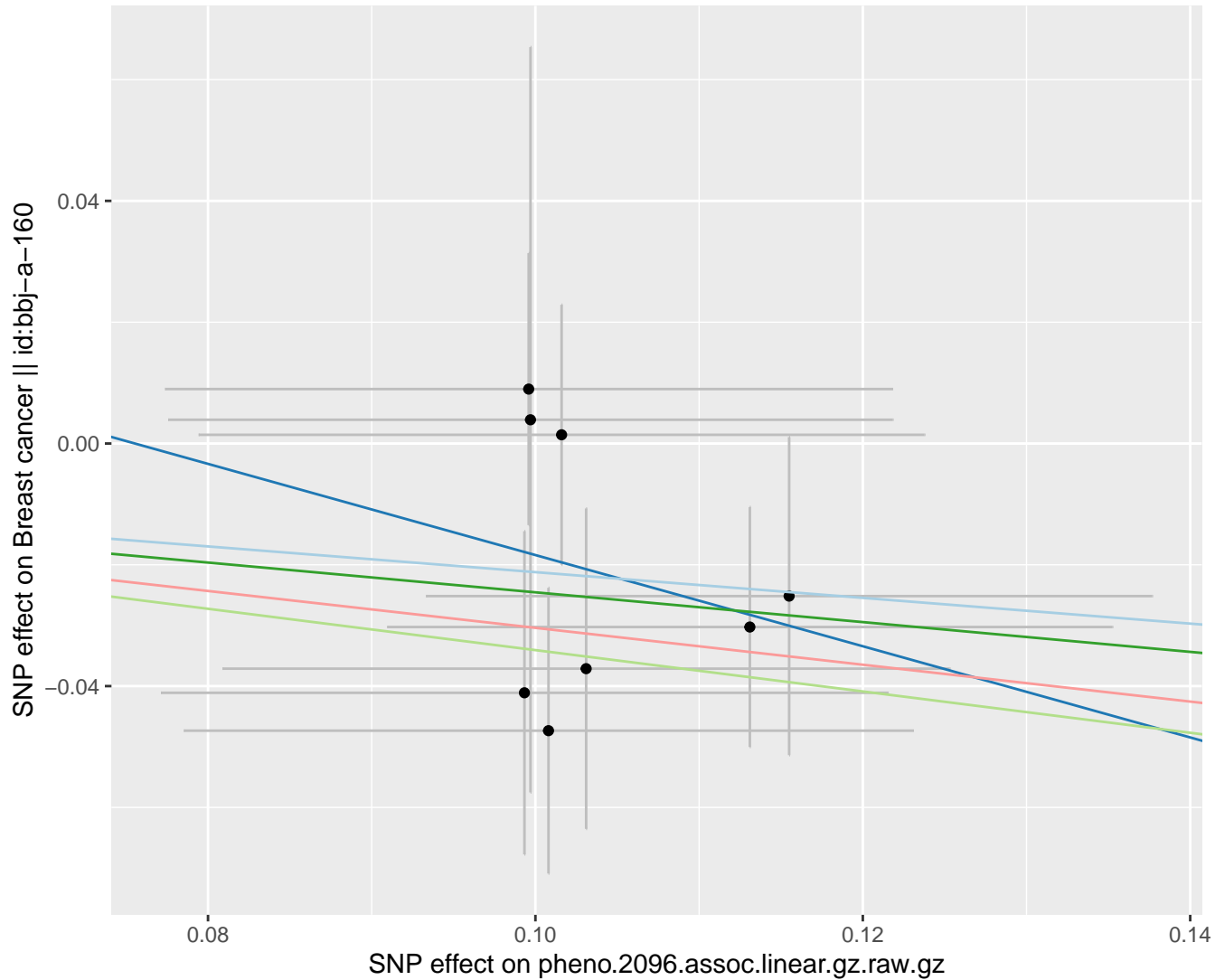

Supplement: Supplementary file 1 [file DataSheet1.ZIP › Supplementary Materials/MR plots for tongue/tongue═╝/Breast cancer/pheno.2096_to_breast cancer_scatter.pdf]

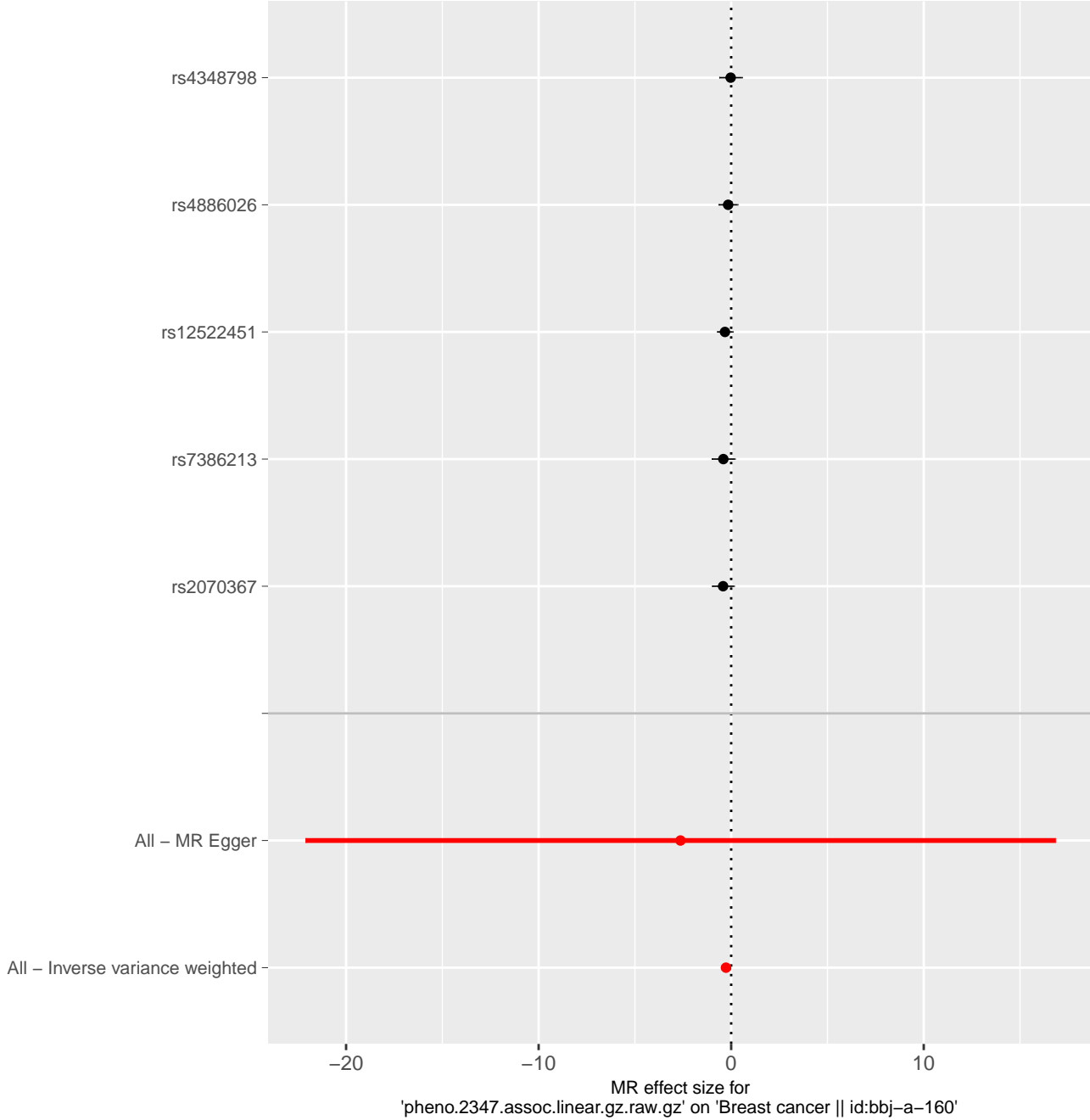

Supplement: Supplementary file 1 [file DataSheet1.ZIP › Supplementary Materials/MR plots for tongue/tongue═╝/Breast cancer/pheno.2347_to_breast cancer_forest.pdf]

# MR Method

- Inverse variance weighted
- MR Egger

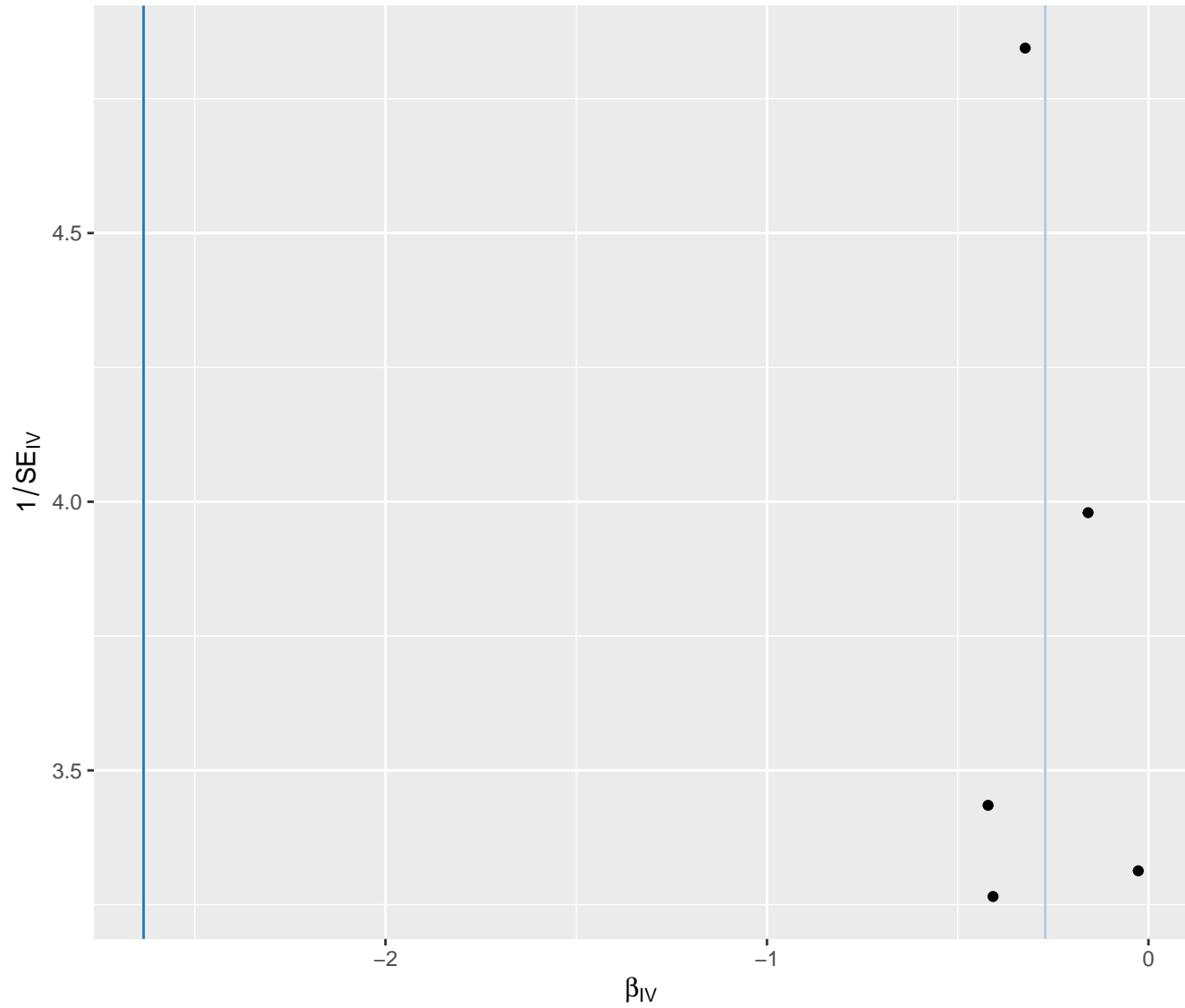

Supplement: Supplementary file 1 [file DataSheet1.ZIP › Supplementary Materials/MR plots for tongue/tongue═╝/Breast cancer/pheno.2347_to_breast cancer_funnel.pdf]

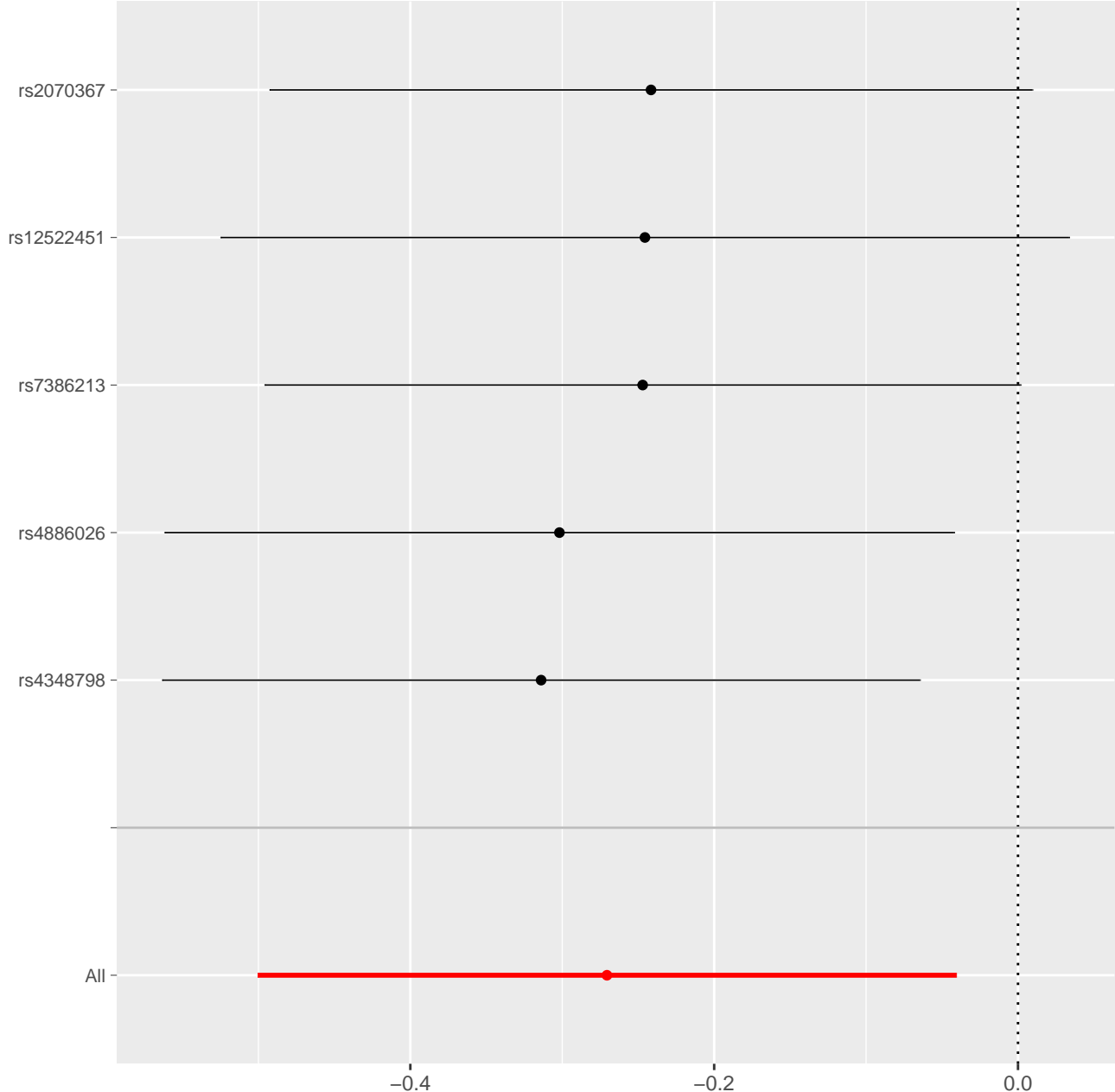

Supplement: Supplementary file 1 [file DataSheet1.ZIP › Supplementary Materials/MR plots for tongue/tongue═╝/Breast cancer/pheno.2347_to_breast cancer_leave_one_out.pdf]

# MR Test

- Inverse variance weighted
- MR Egger
- Simple mode
- Weighted median
- Weighted mode

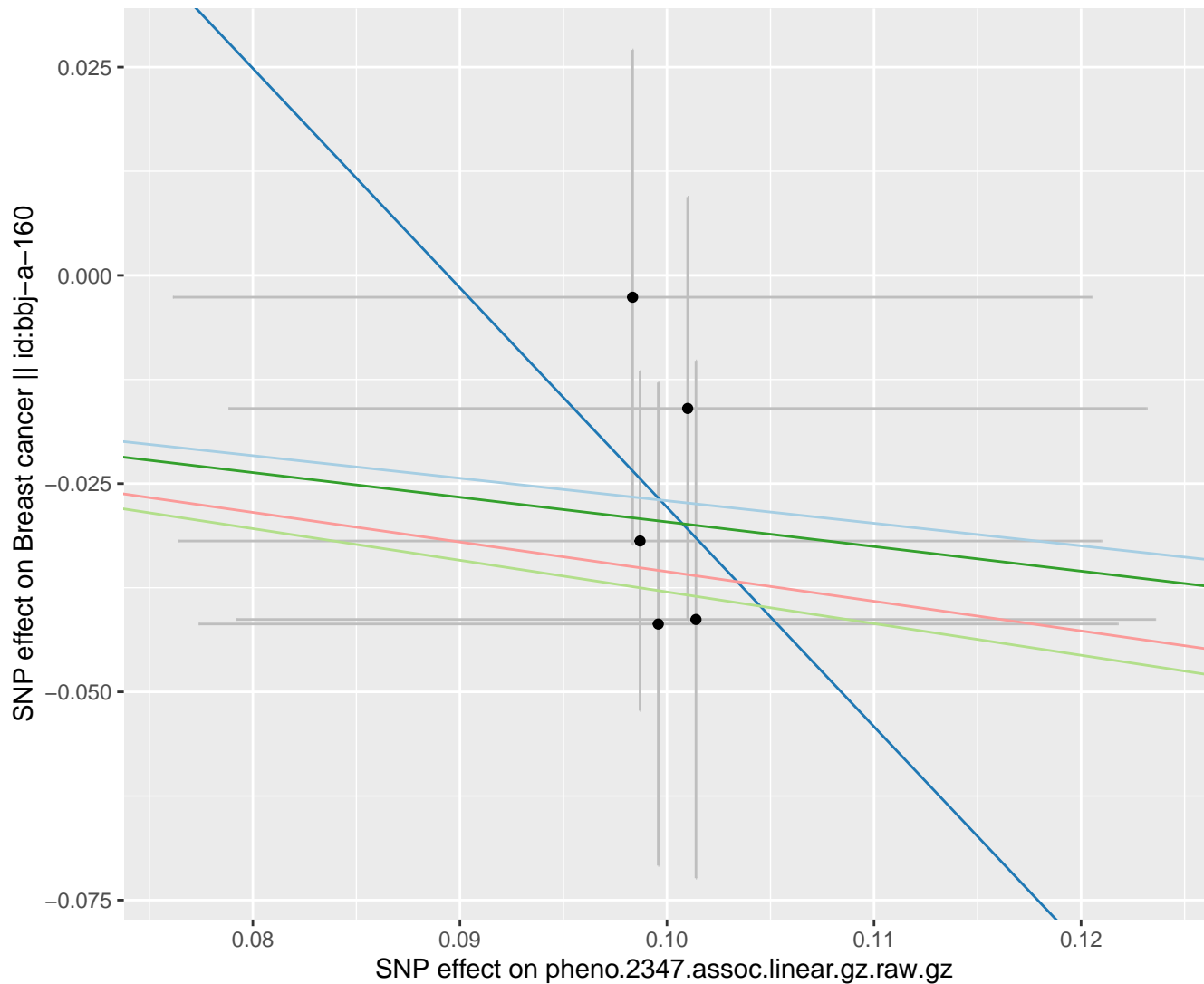

Supplement: Supplementary file 1 [file DataSheet1.ZIP › Supplementary Materials/MR plots for tongue/tongue═╝/Breast cancer/pheno.2347_to_breast cancer_scatter.pdf]

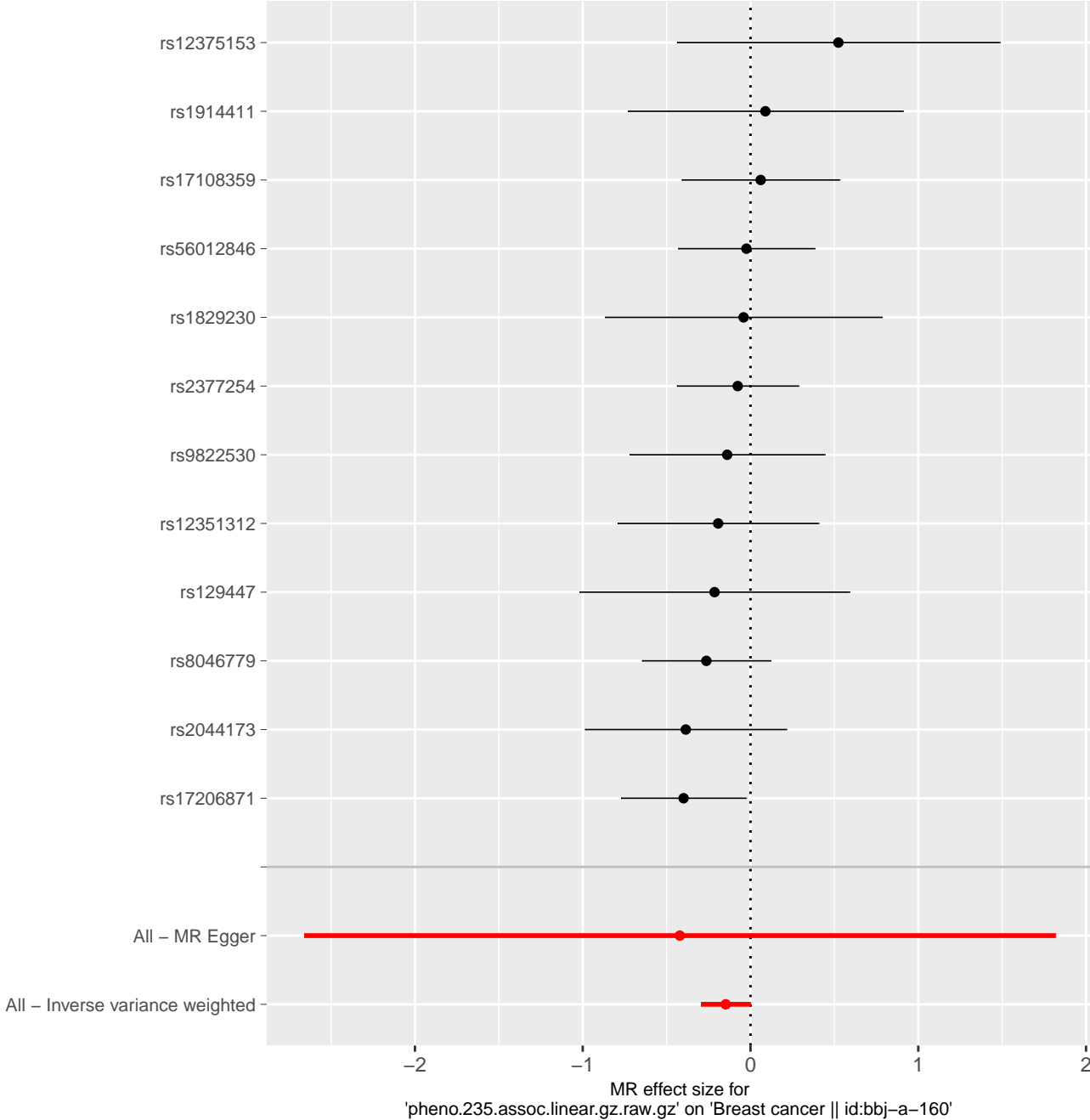

Supplement: Supplementary file 1 [file DataSheet1.ZIP › Supplementary Materials/MR plots for tongue/tongue═╝/Breast cancer/pheno.235_to_breast cancer_forest.pdf]

# MR Method

- Inverse variance weighted
- MR Egger

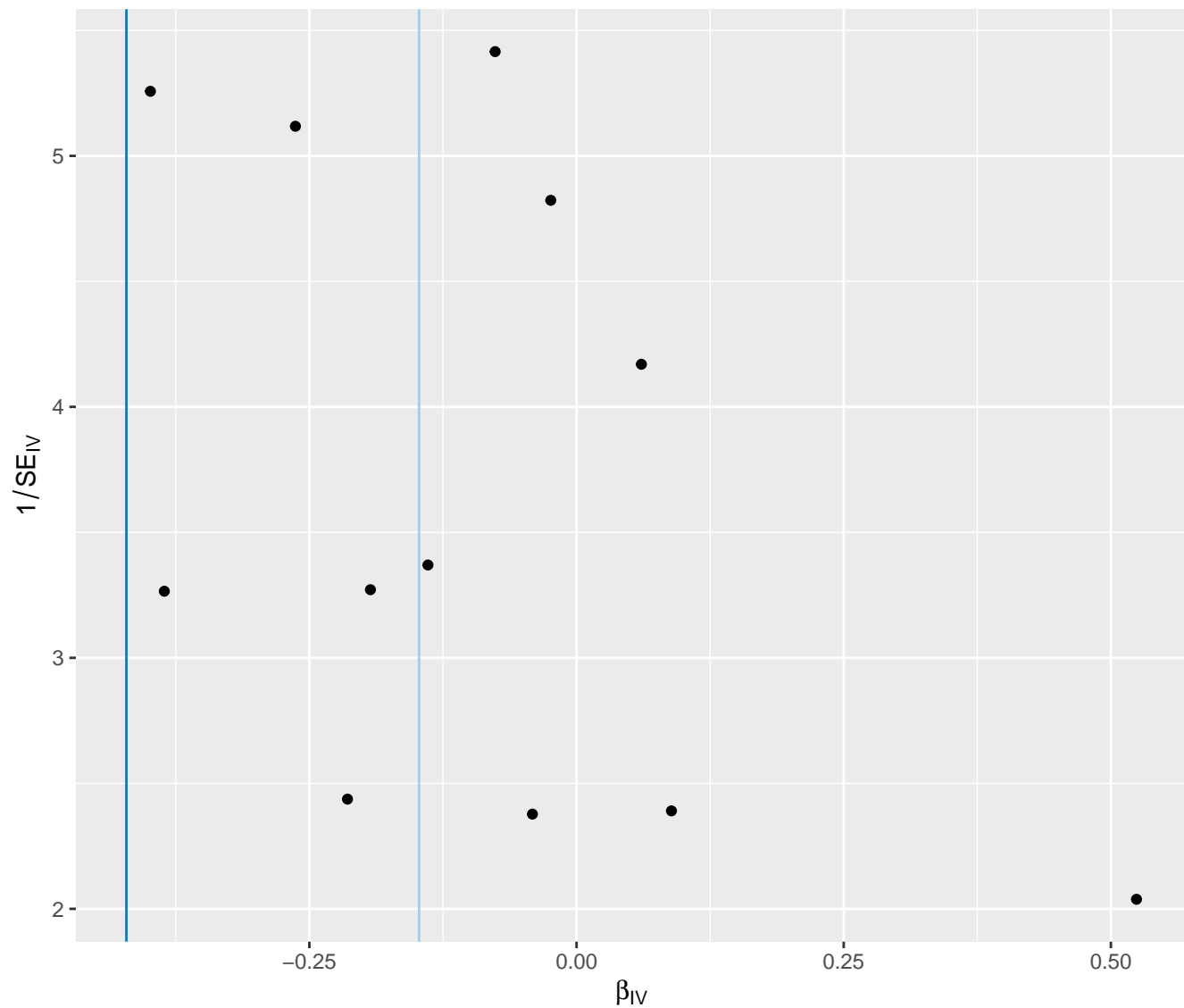

Supplement: Supplementary file 1 [file DataSheet1.ZIP › Supplementary Materials/MR plots for tongue/tongue═╝/Breast cancer/pheno.235_to_breast cancer_funnel.pdf]

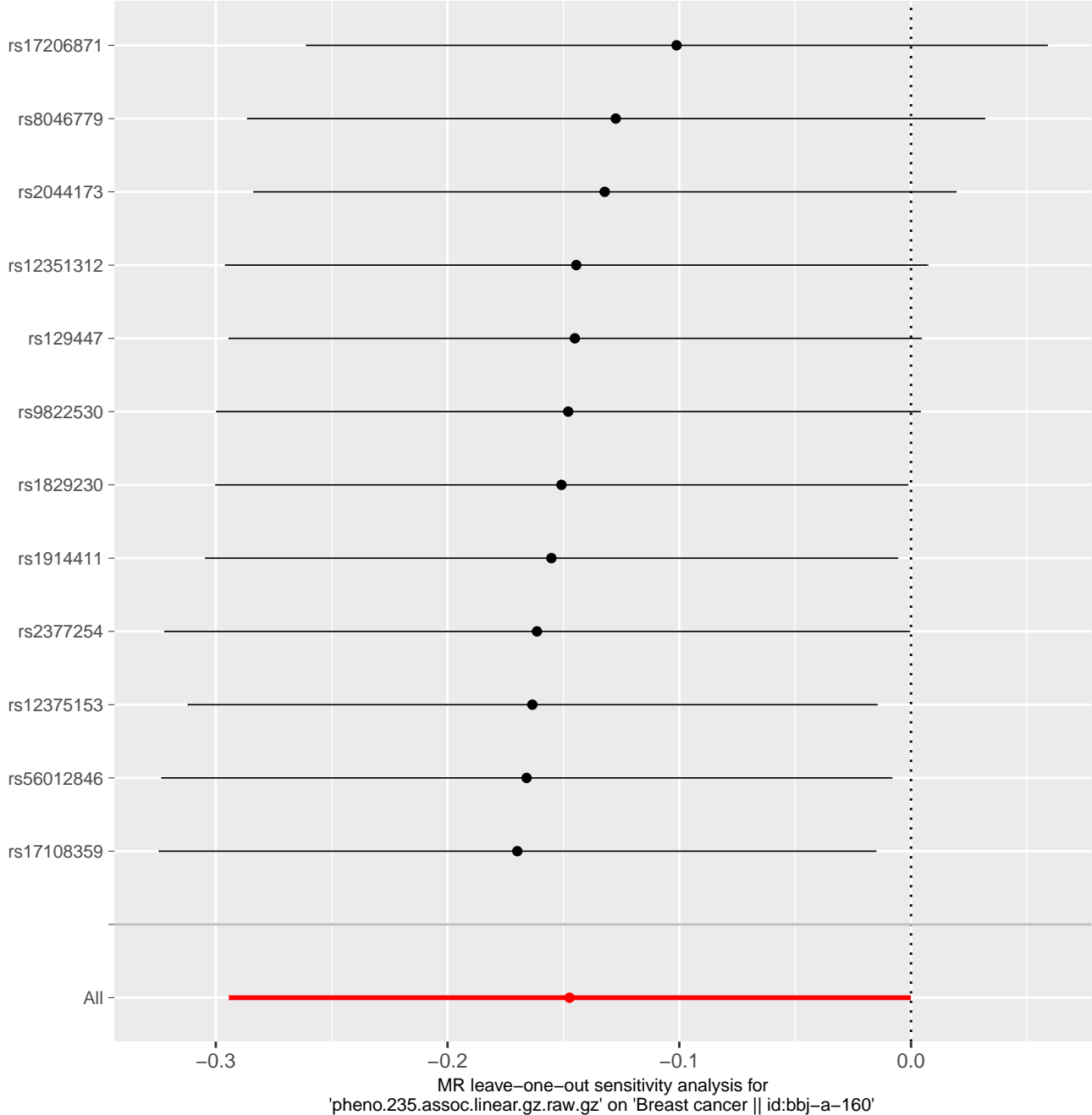

Supplement: Supplementary file 1 [file DataSheet1.ZIP › Supplementary Materials/MR plots for tongue/tongue═╝/Breast cancer/pheno.235_to_breast cancer_leave_one_out.pdf]

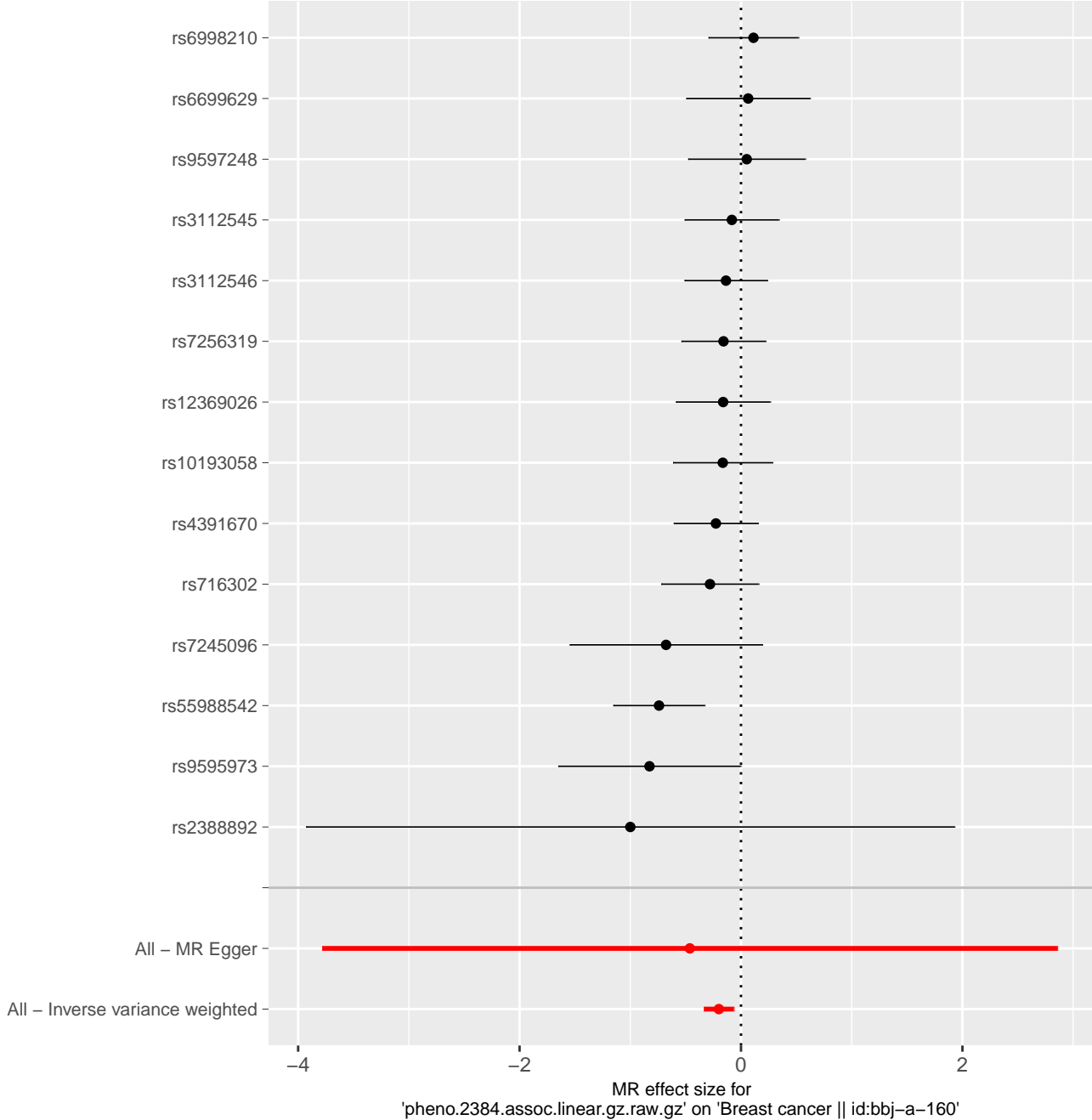

Supplement: Supplementary file 1 [file DataSheet1.ZIP › Supplementary Materials/MR plots for tongue/tongue═╝/Breast cancer/pheno.2384_to_breast cancer_forest.pdf]

# MR Method

- Inverse variance weighted
- MR Egger

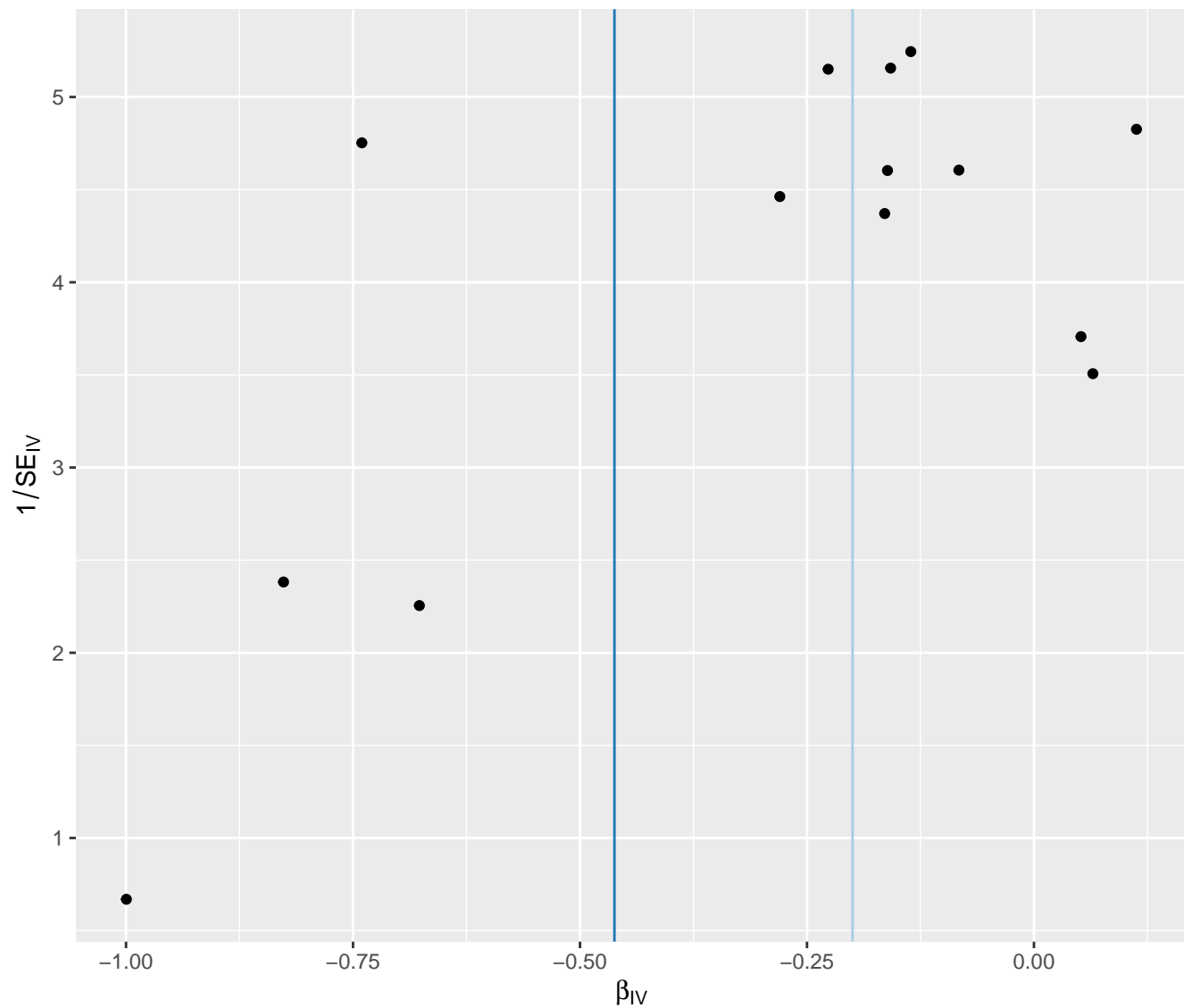

Supplement: Supplementary file 1 [file DataSheet1.ZIP › Supplementary Materials/MR plots for tongue/tongue═╝/Breast cancer/pheno.2384_to_breast cancer_funnel.pdf]

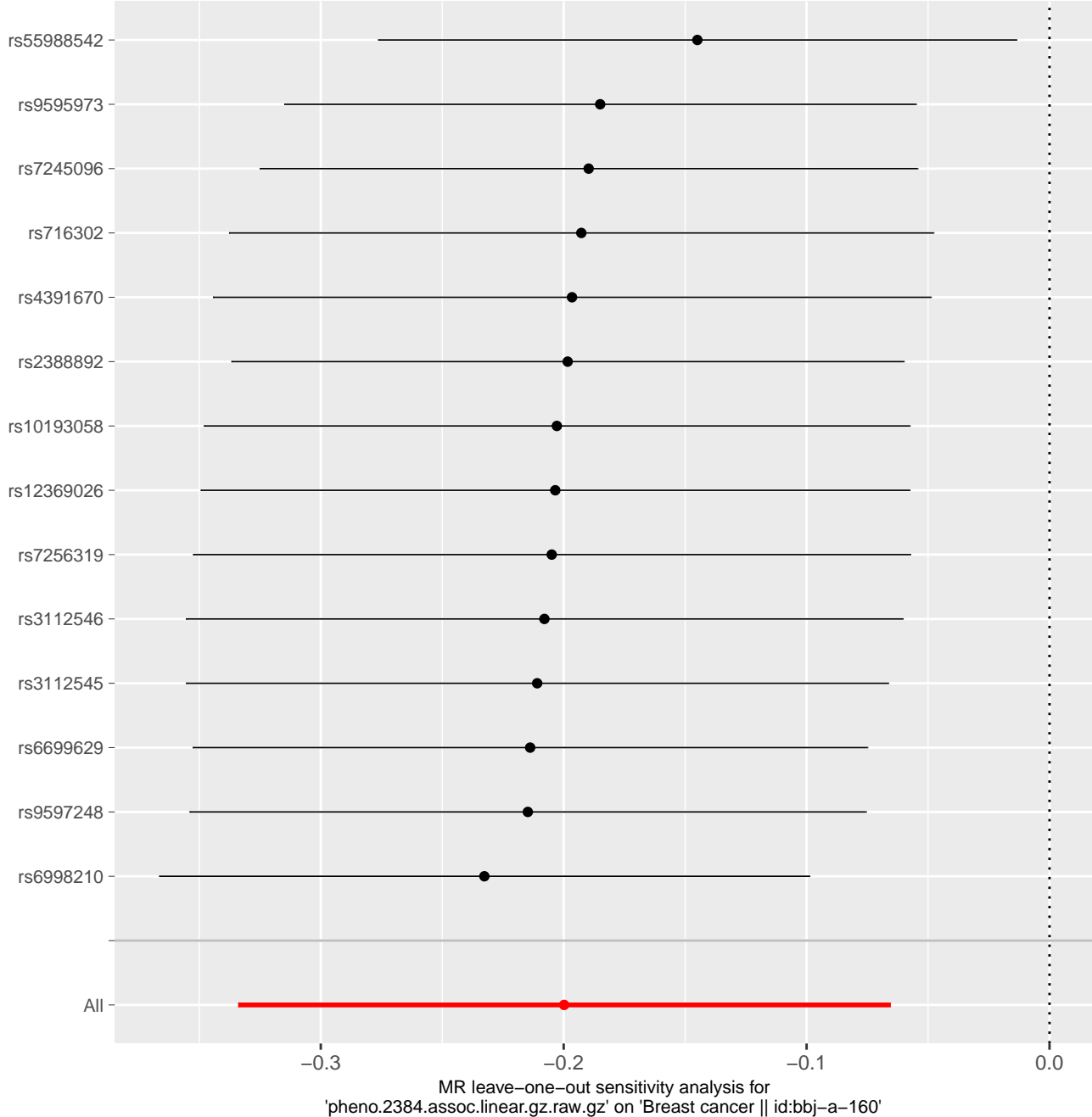

Supplement: Supplementary file 1 [file DataSheet1.ZIP › Supplementary Materials/MR plots for tongue/tongue═╝/Breast cancer/pheno.2384_to_breast cancer_leave_one_out.pdf]

# MR Test

- Inverse variance weighted
- MR Egger
- Simple mode
- Weighted median
- Weighted mode

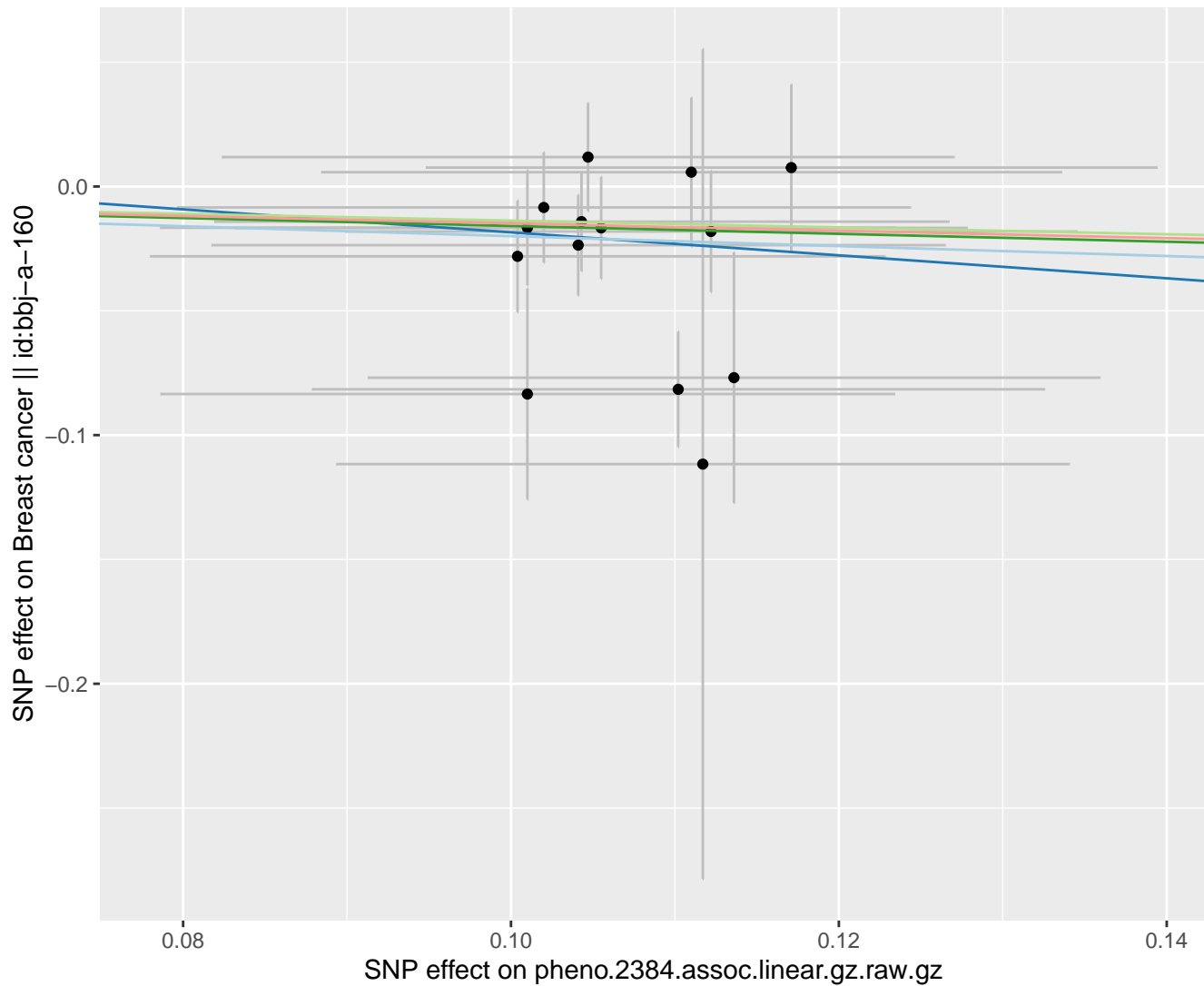

Supplement: Supplementary file 1 [file DataSheet1.ZIP › Supplementary Materials/MR plots for tongue/tongue═╝/Breast cancer/pheno.2384_to_breast cancer_scatter.pdf]

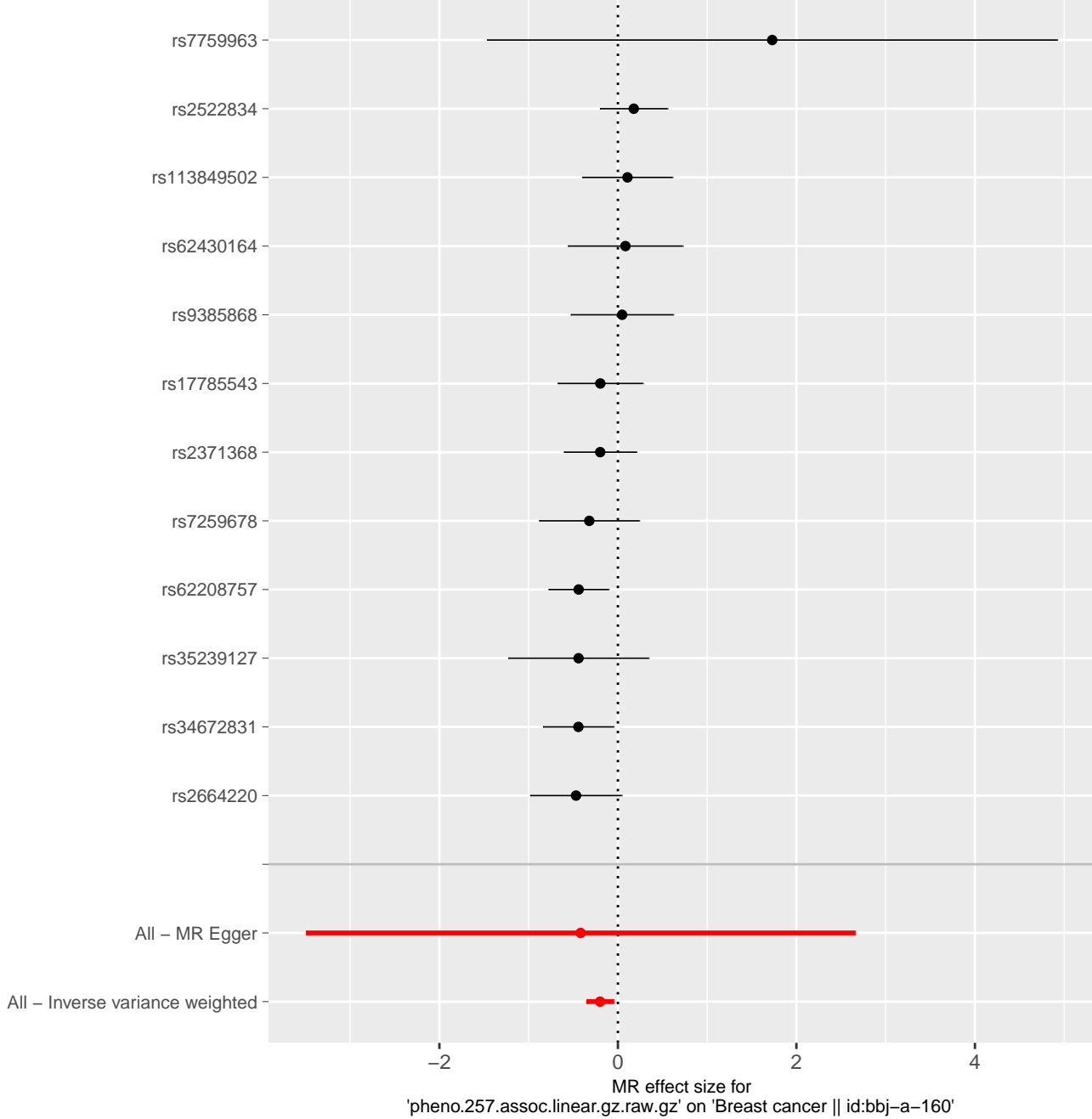

Supplement: Supplementary file 1 [file DataSheet1.ZIP › Supplementary Materials/MR plots for tongue/tongue═╝/Breast cancer/pheno.257_to_breast cancer_forest.pdf]

# MR Method

- Inverse variance weighted
- MR Egger

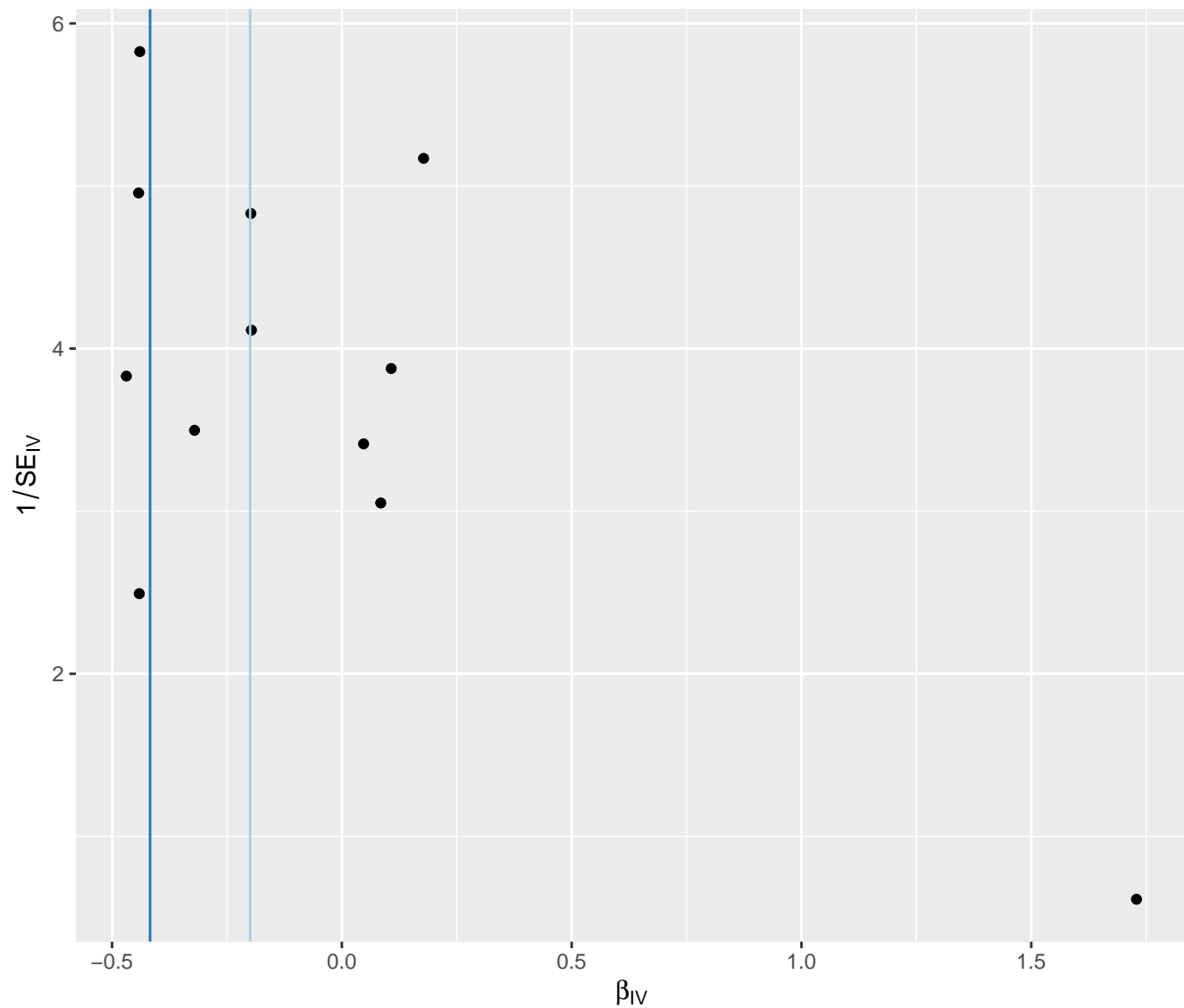

Supplement: Supplementary file 1 [file DataSheet1.ZIP › Supplementary Materials/MR plots for tongue/tongue═╝/Breast cancer/pheno.257_to_breast cancer_funnel.pdf]

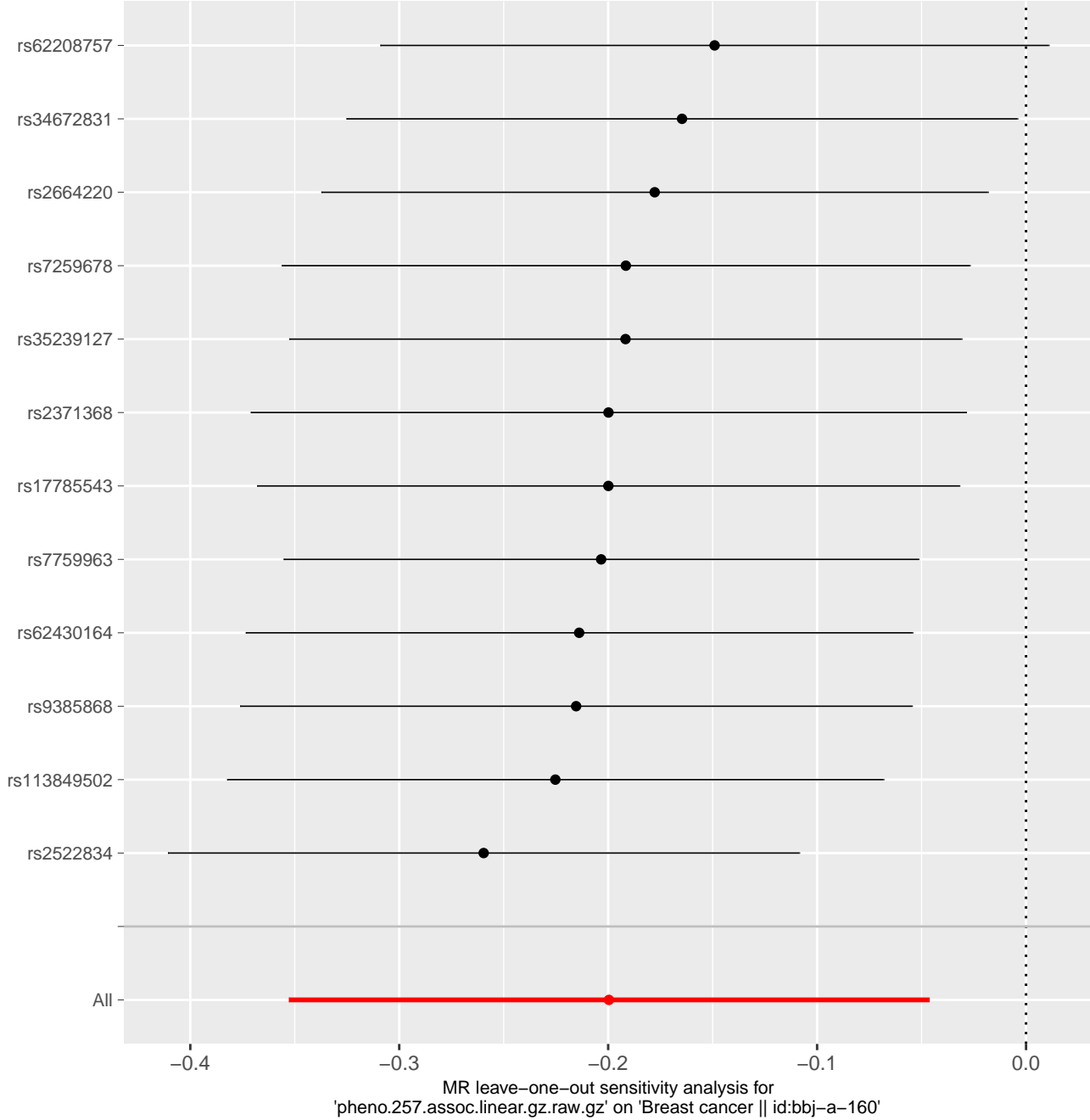

Supplement: Supplementary file 1 [file DataSheet1.ZIP › Supplementary Materials/MR plots for tongue/tongue═╝/Breast cancer/pheno.257_to_breast cancer_leave_one_out.pdf]

# MR Test

- Inverse variance weighted
- MR Egger
- Simple mode
- Weighted median
- Weighted mode

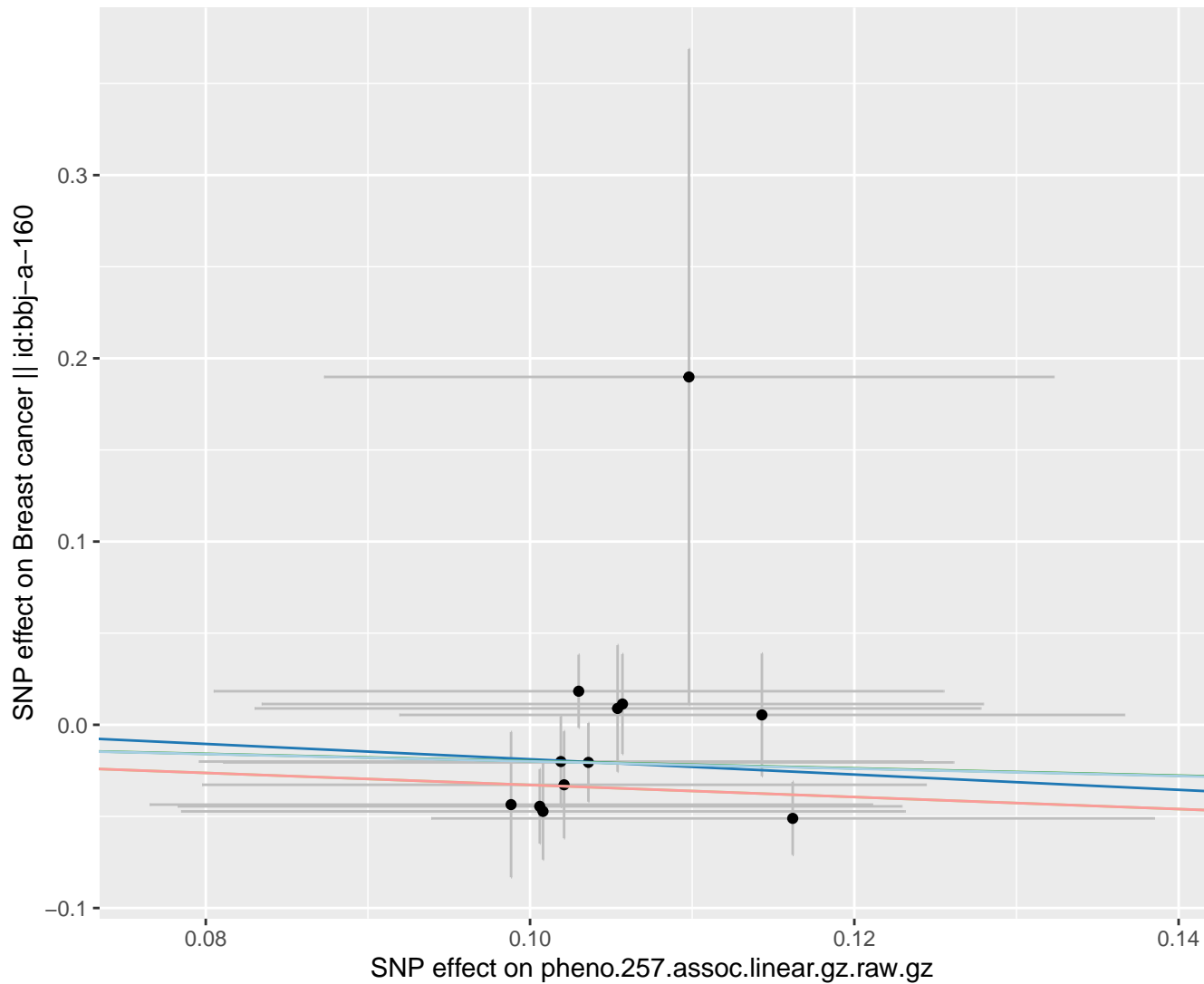

Supplement: Supplementary file 1 [file DataSheet1.ZIP › Supplementary Materials/MR plots for tongue/tongue═╝/Breast cancer/pheno.257_to_breast cancer_scatter.pdf]

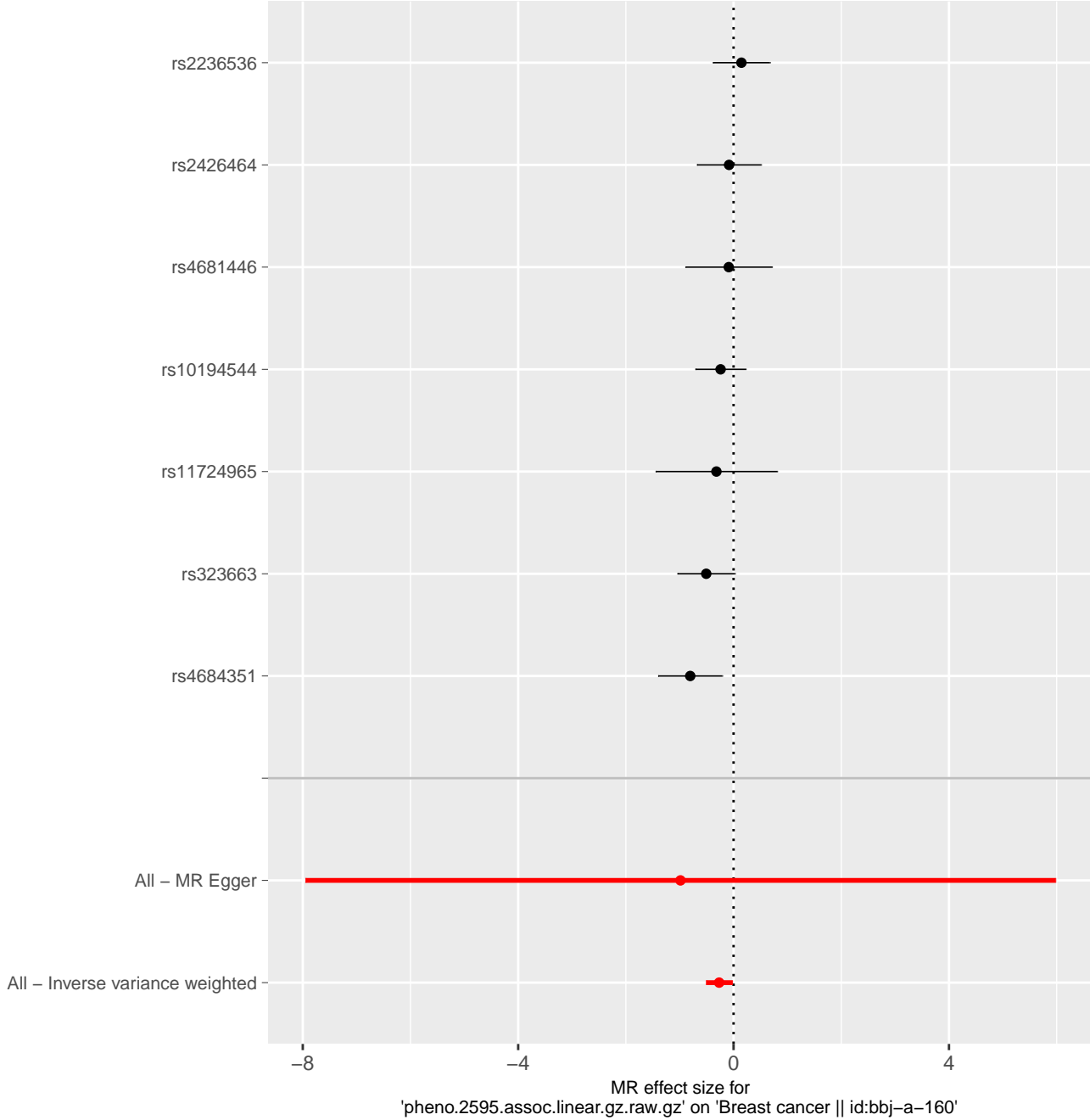

Supplement: Supplementary file 1 [file DataSheet1.ZIP › Supplementary Materials/MR plots for tongue/tongue═╝/Breast cancer/pheno.2595_to_breast cancer_forest.pdf]

# MR Method

- Inverse variance weighted
- MR Egger

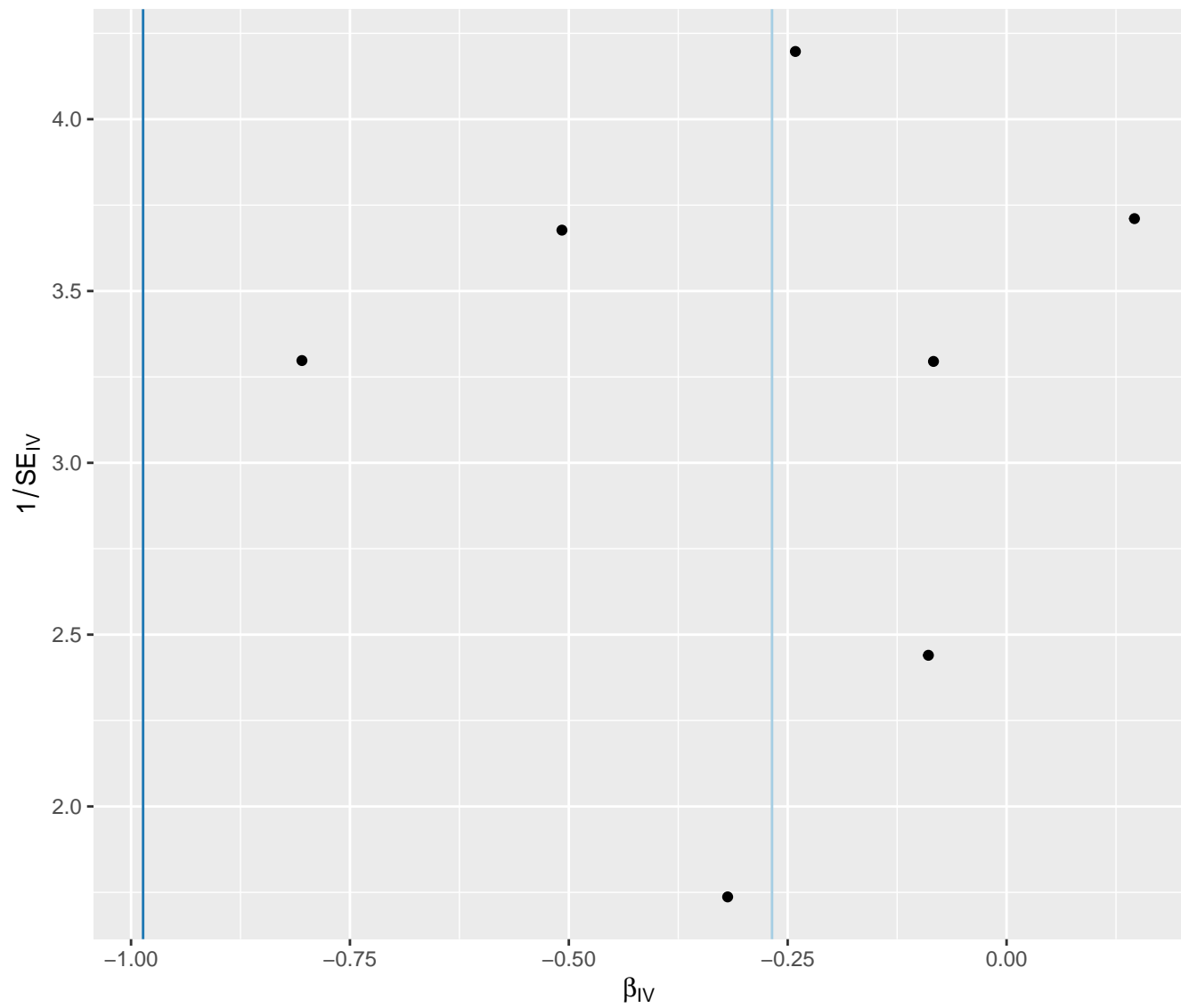

Supplement: Supplementary file 1 [file DataSheet1.ZIP › Supplementary Materials/MR plots for tongue/tongue═╝/Breast cancer/pheno.2595_to_breast cancer_funnel.pdf]

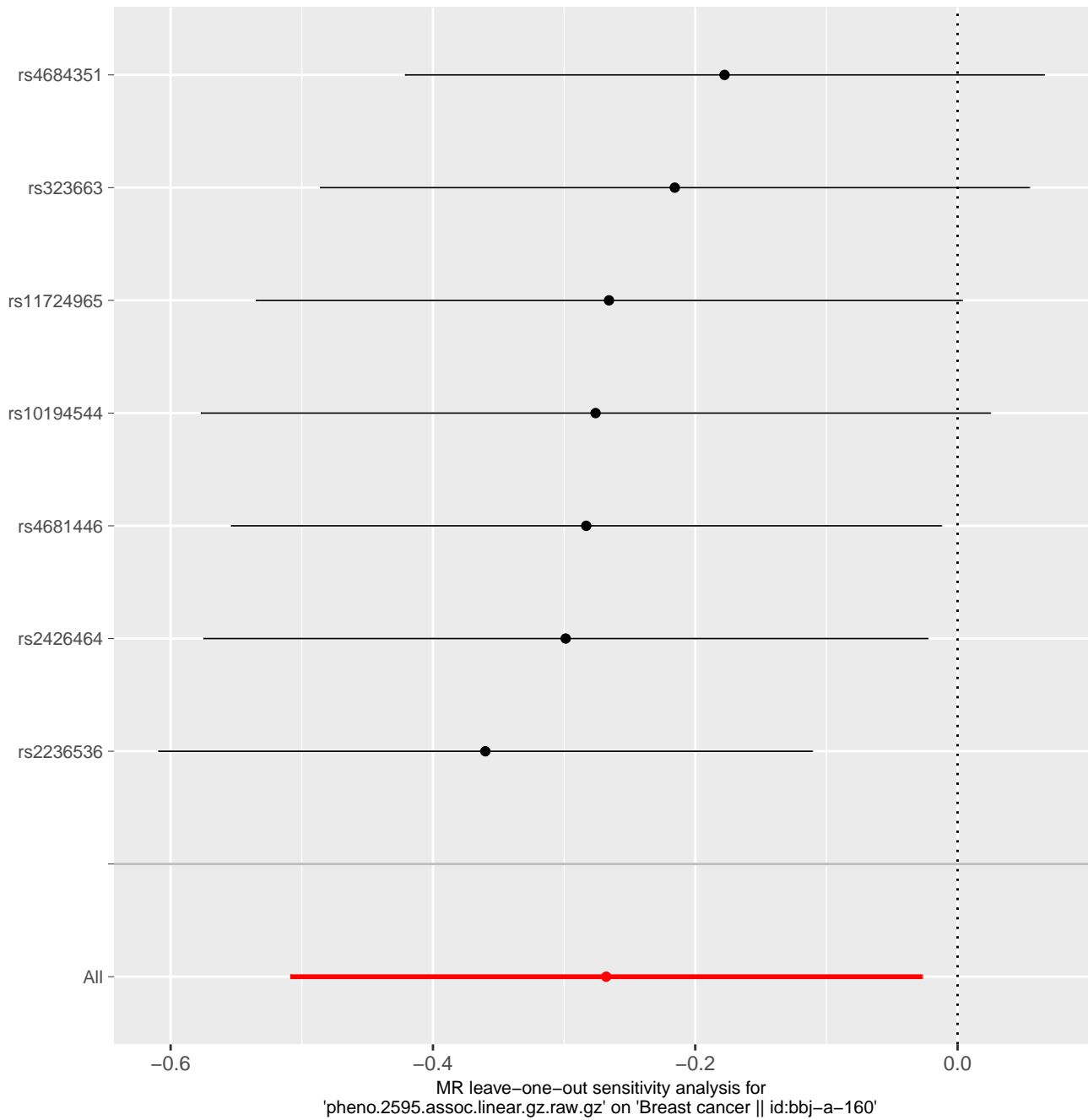

Supplement: Supplementary file 1 [file DataSheet1.ZIP › Supplementary Materials/MR plots for tongue/tongue═╝/Breast cancer/pheno.2595_to_breast cancer_leave_one_out.pdf]

# MR Test

- Inverse variance weighted
- MR Egger
- Simple mode
- Weighted median
- Weighted mode

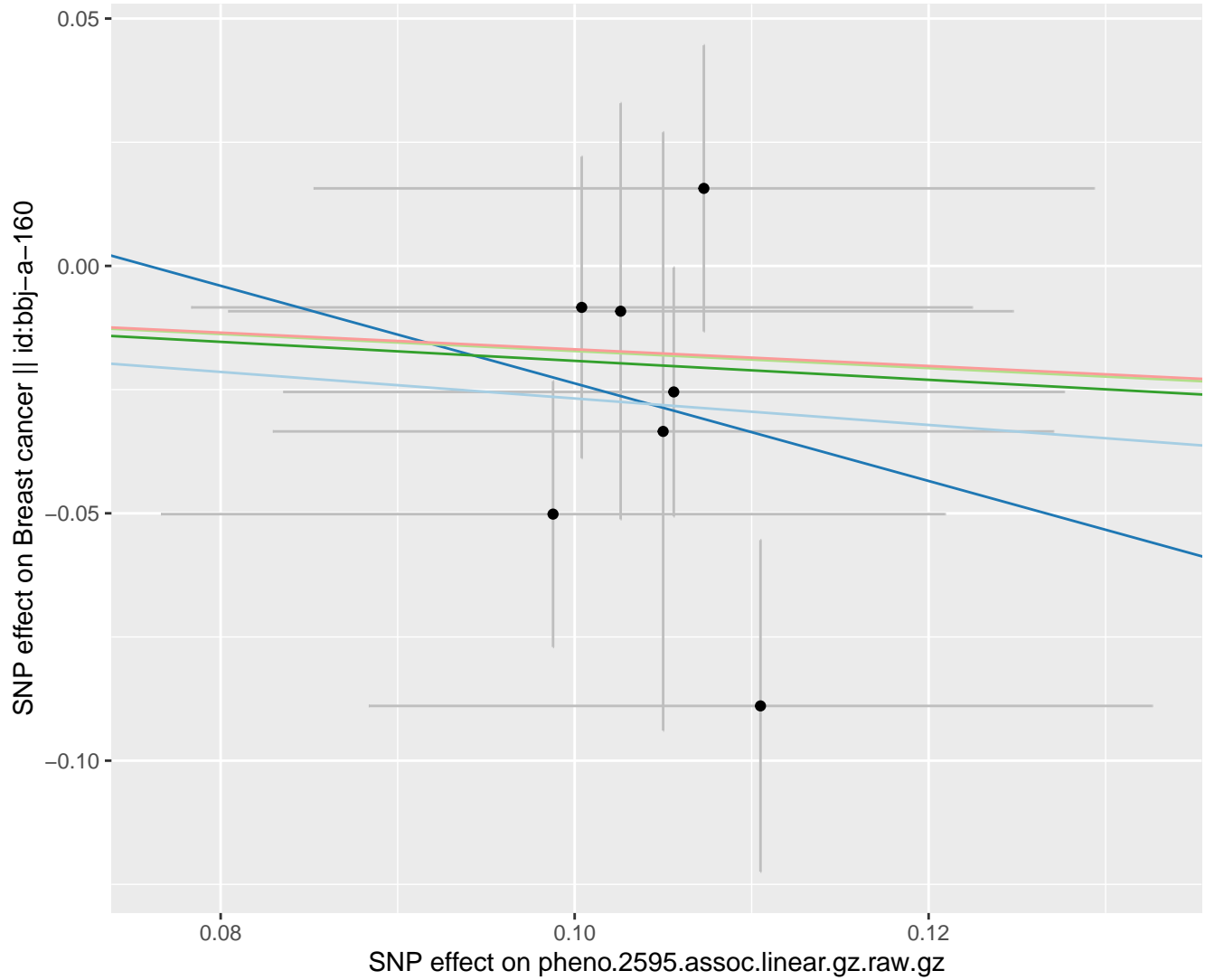

Supplement: Supplementary file 1 [file DataSheet1.ZIP › Supplementary Materials/MR plots for tongue/tongue═╝/Breast cancer/pheno.2595_to_breast cancer_scatter.pdf]

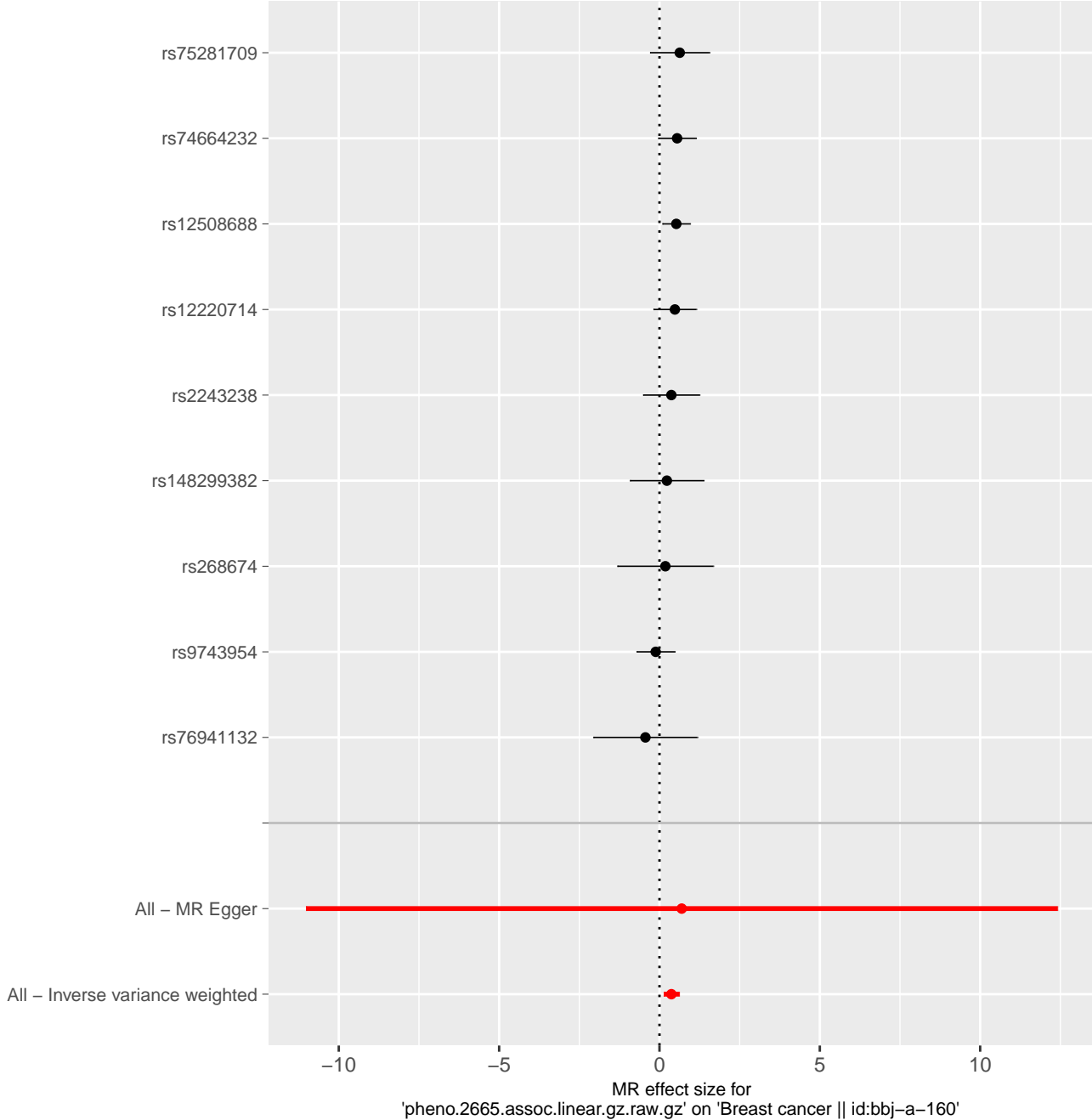

Supplement: Supplementary file 1 [file DataSheet1.ZIP › Supplementary Materials/MR plots for tongue/tongue═╝/Breast cancer/pheno.2665_to_breast cancer_forest.pdf]

# MR Method

- Inverse variance weighted
- MR Egger

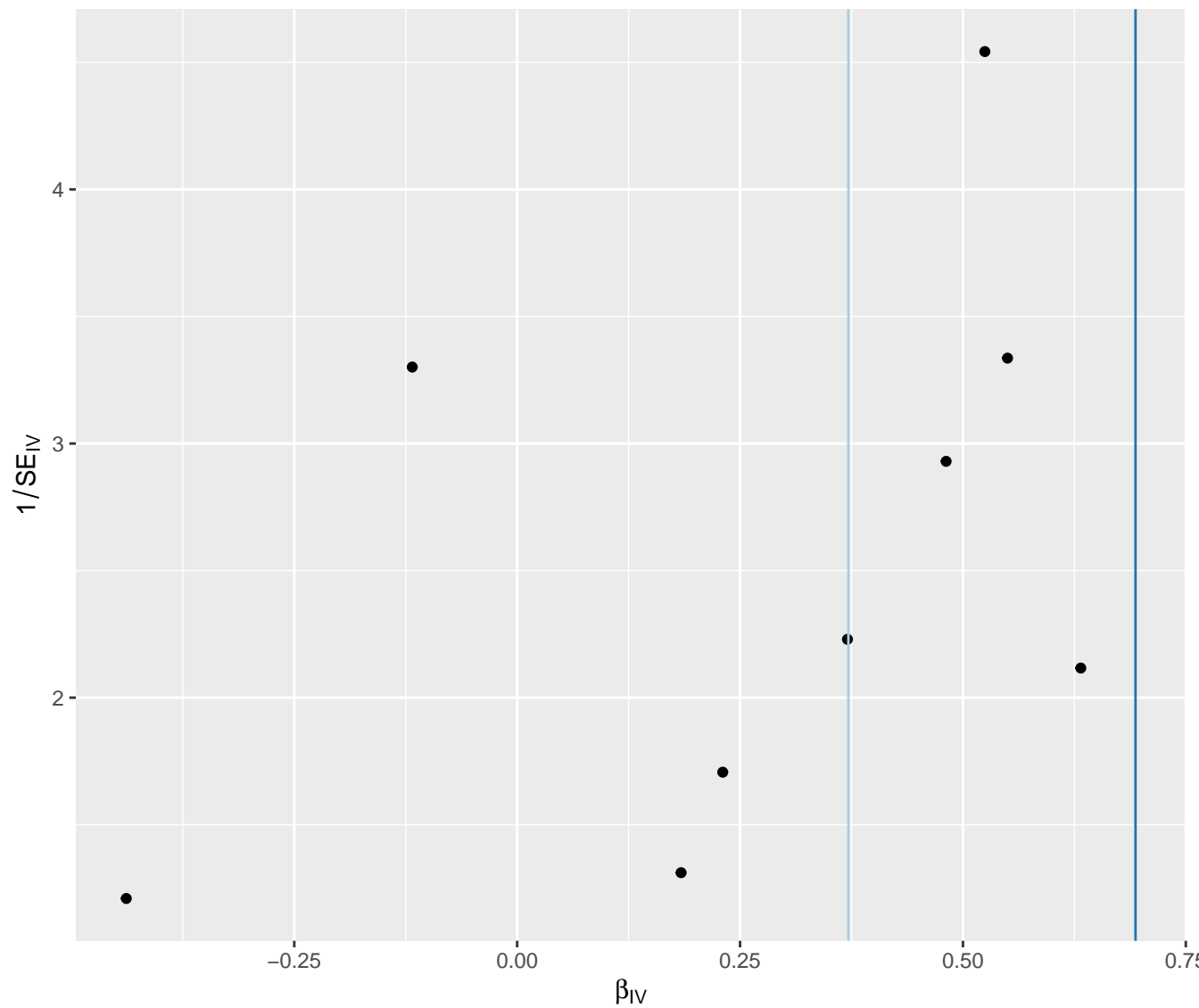

Supplement: Supplementary file 1 [file DataSheet1.ZIP › Supplementary Materials/MR plots for tongue/tongue═╝/Breast cancer/pheno.2665_to_breast cancer_funnel.pdf]

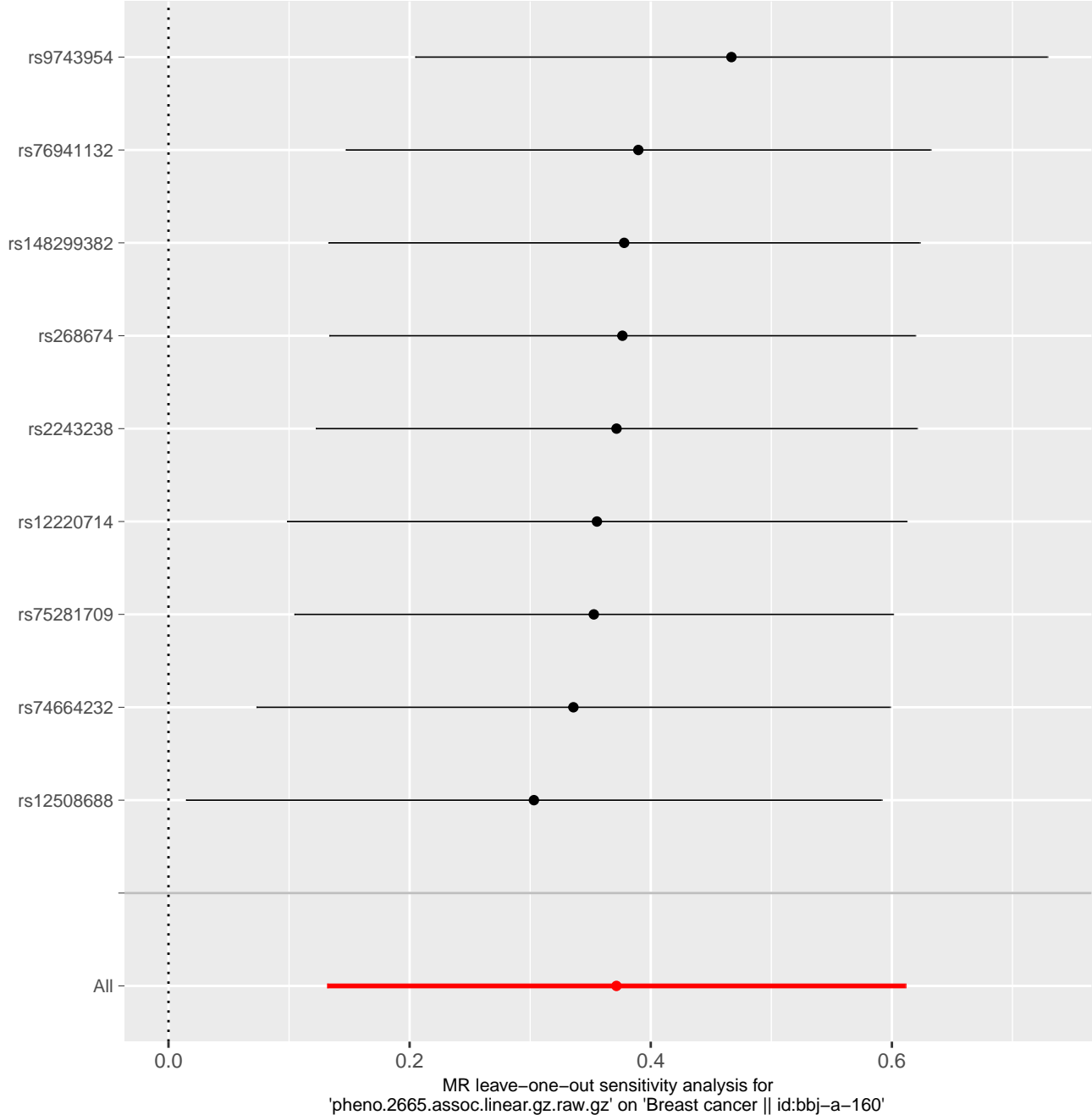

Supplement: Supplementary file 1 [file DataSheet1.ZIP › Supplementary Materials/MR plots for tongue/tongue═╝/Breast cancer/pheno.2665_to_breast cancer_leave_one_out.pdf]

# MR Test

- Inverse variance weighted
- MR Egger
- Simple mode
- Weighted median
- Weighted mode

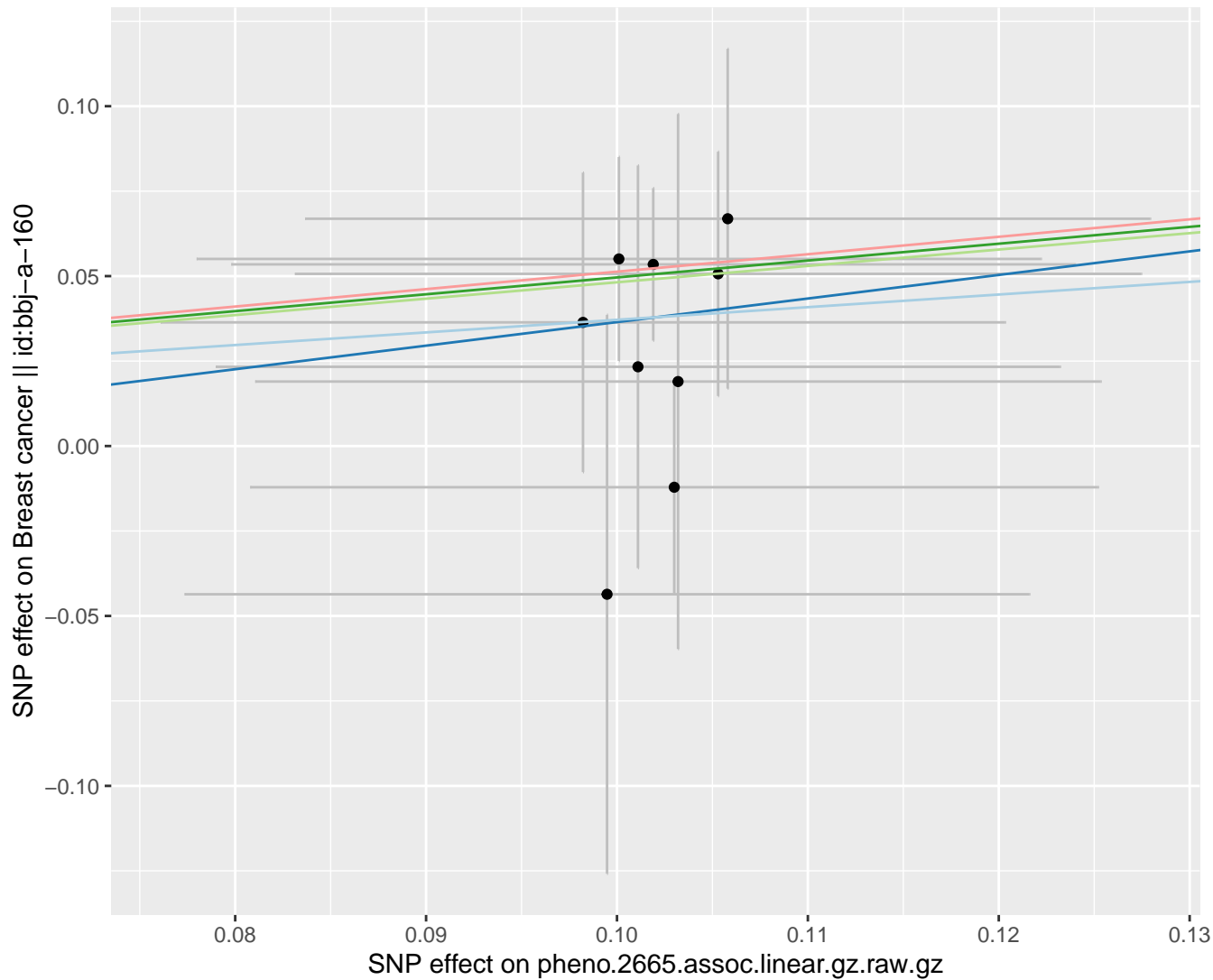

Supplement: Supplementary file 1 [file DataSheet1.ZIP › Supplementary Materials/MR plots for tongue/tongue═╝/Breast cancer/pheno.2665_to_breast cancer_scatter.pdf]

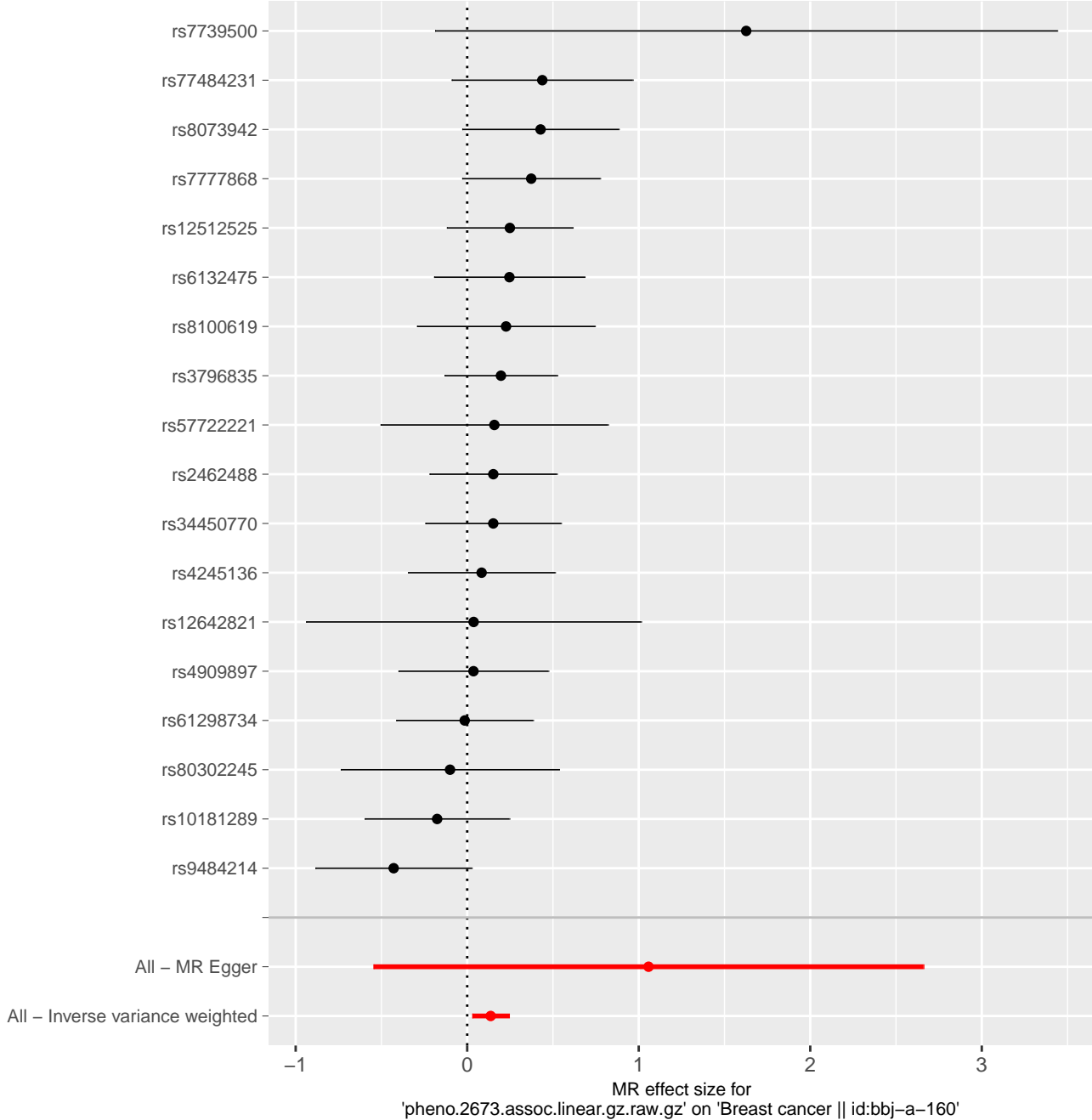

Supplement: Supplementary file 1 [file DataSheet1.ZIP › Supplementary Materials/MR plots for tongue/tongue═╝/Breast cancer/pheno.2673_to_breast cancer_forest.pdf]

# MR Method

- Inverse variance weighted
- MR Egger

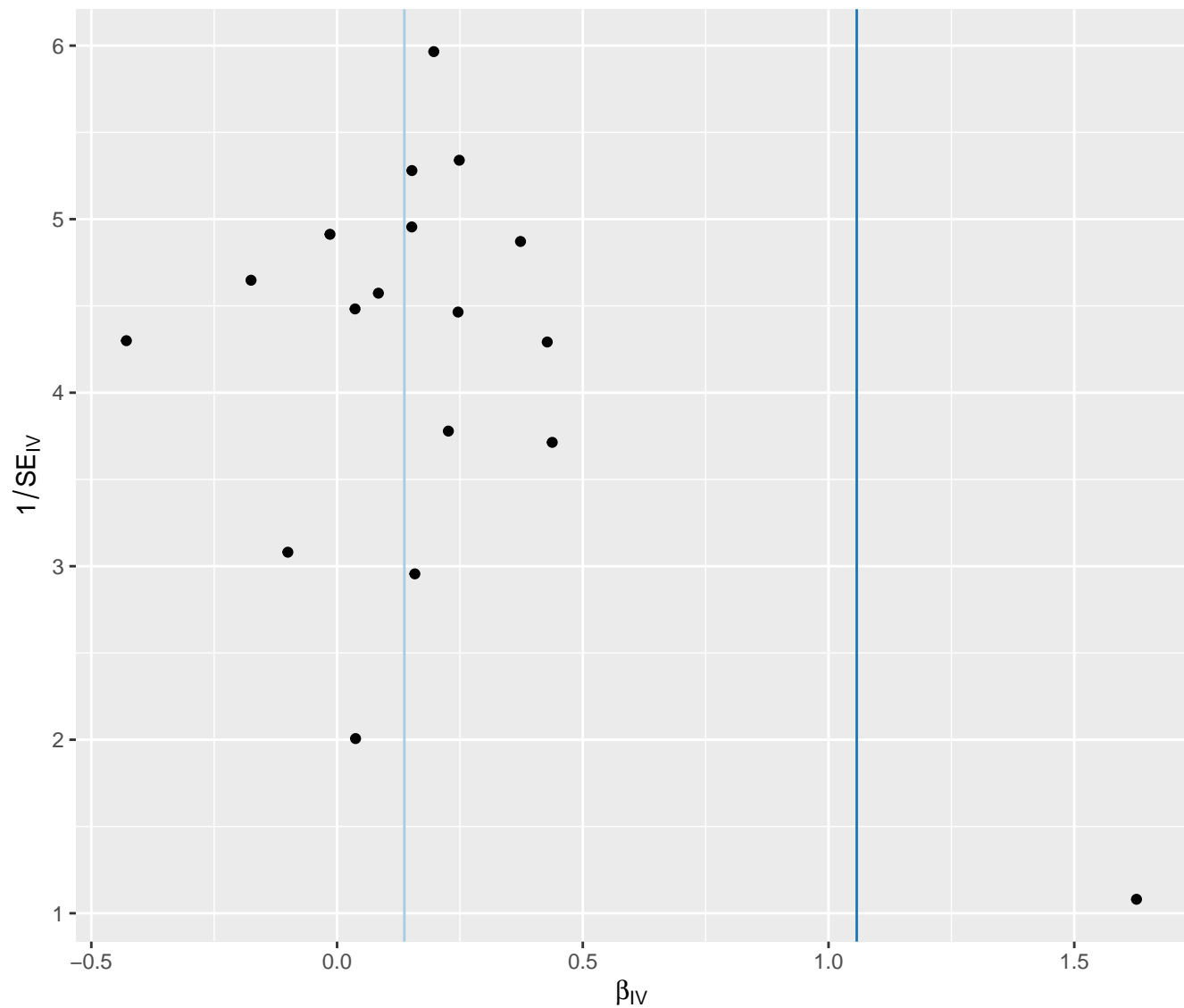

Supplement: Supplementary file 1 [file DataSheet1.ZIP › Supplementary Materials/MR plots for tongue/tongue═╝/Breast cancer/pheno.2673_to_breast cancer_funnel.pdf]

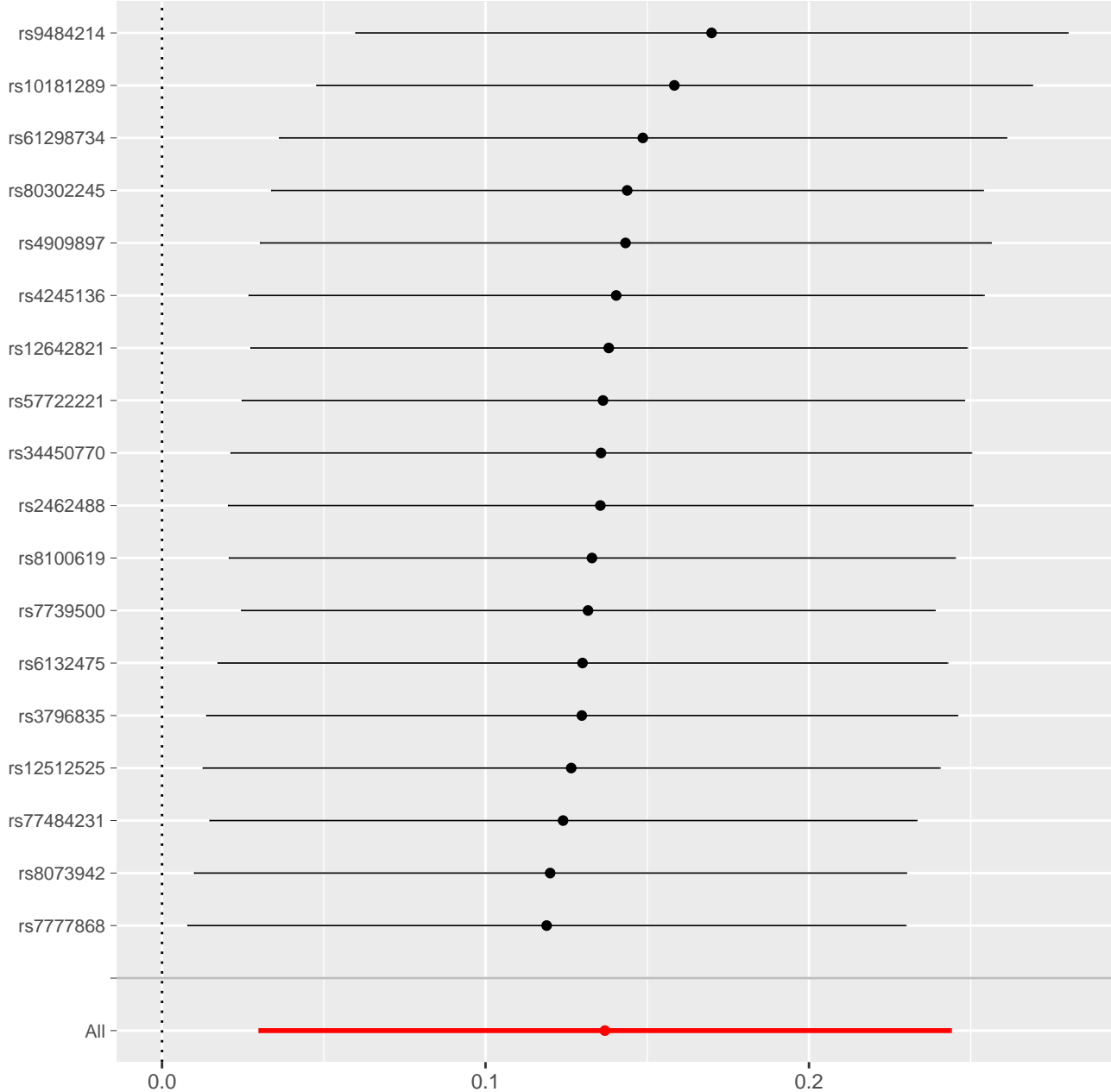

MR leave-one-out sensitivity analysis for  
'pheno.2673.assoc.linear.gz.raw.gz' on 'Breast cancer || id:bbj-a-160'

Supplement: Supplementary file 1 [file DataSheet1.ZIP › Supplementary Materials/MR plots for tongue/tongue═╝/Breast cancer/pheno.2673_to_breast cancer_leave_one_out.pdf]

# MR Test

- Inverse variance weighted
- MR Egger
- Simple mode
- Weighted median
- Weighted mode

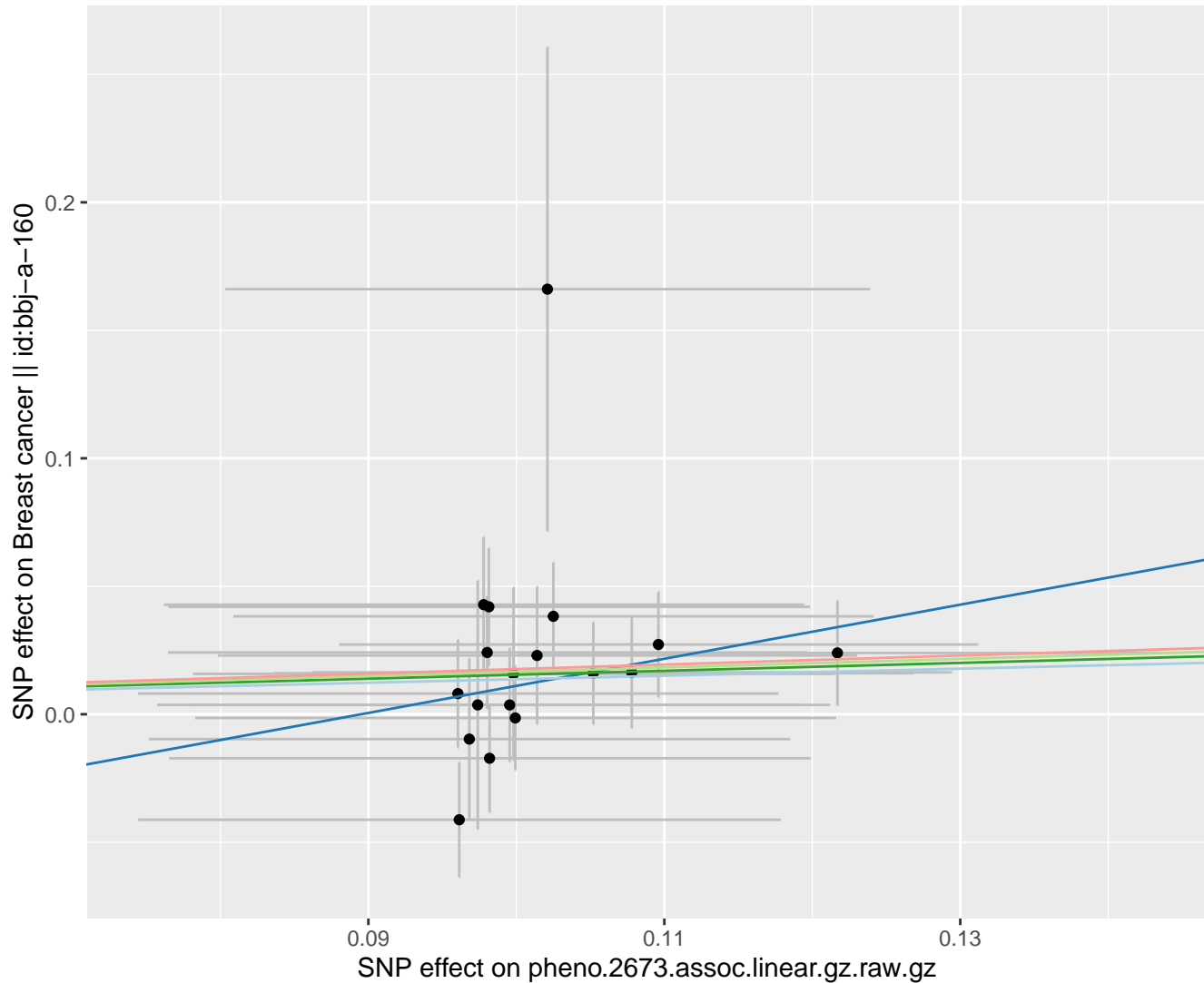

Supplement: Supplementary file 1 [file DataSheet1.ZIP › Supplementary Materials/MR plots for tongue/tongue═╝/Breast cancer/pheno.2673_to_breast cancer_scatter.pdf]

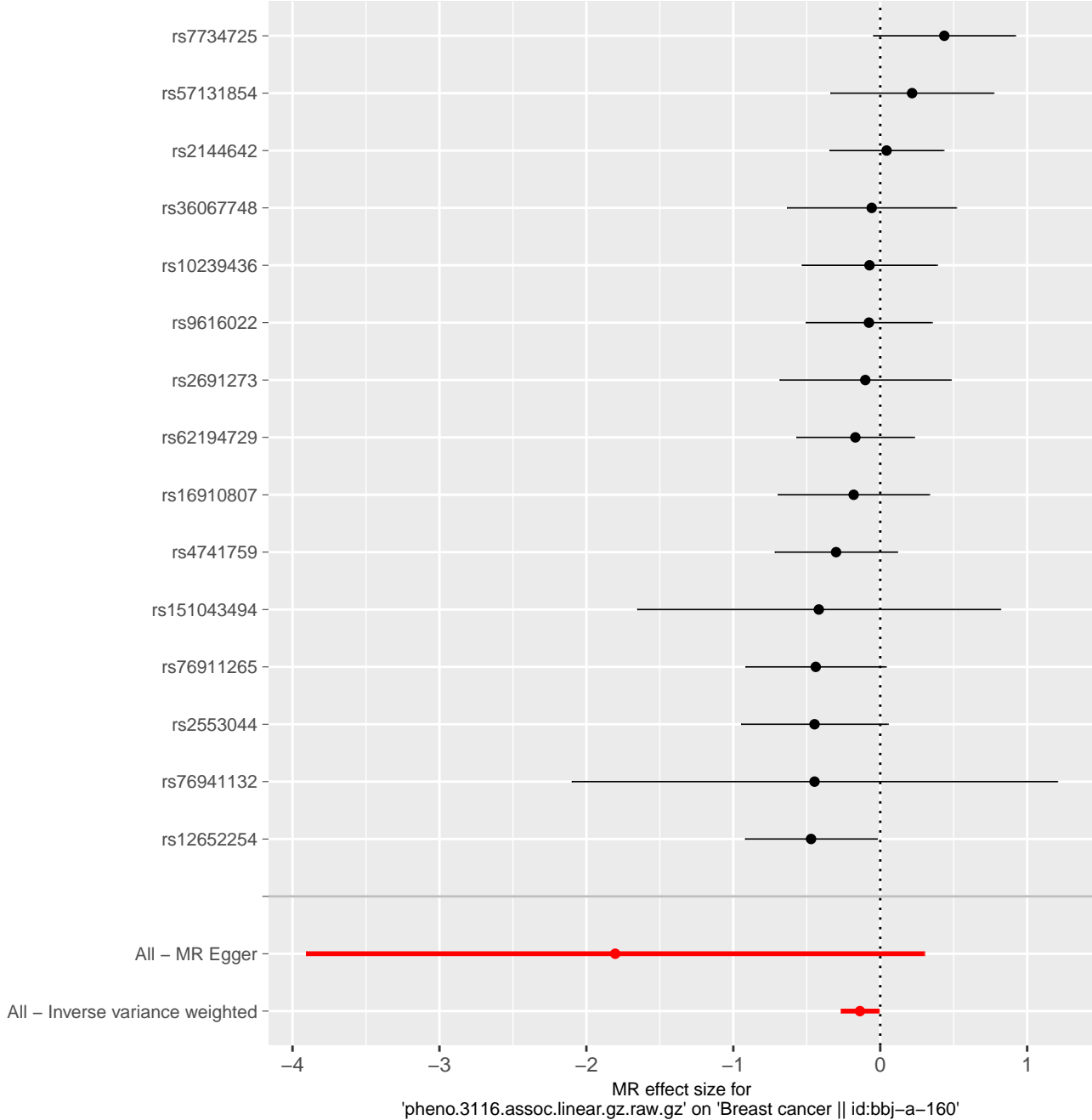

Supplement: Supplementary file 1 [file DataSheet1.ZIP › Supplementary Materials/MR plots for tongue/tongue═╝/Breast cancer/pheno.3116_to_breast cancer_forest.pdf]

# MR Method

- Inverse variance weighted
- MR Egger

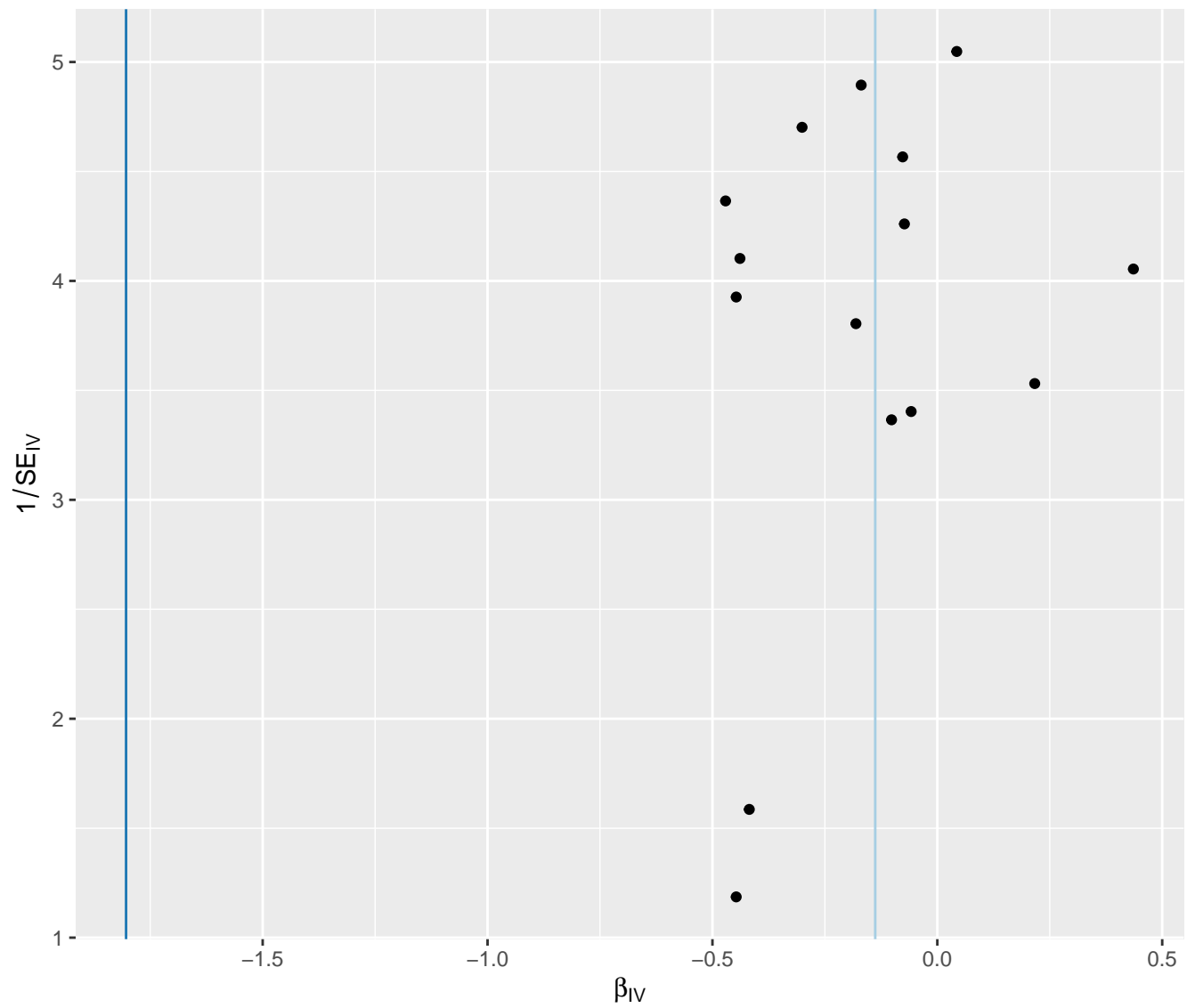

Supplement: Supplementary file 1 [file DataSheet1.ZIP › Supplementary Materials/MR plots for tongue/tongue═╝/Breast cancer/pheno.3116_to_breast cancer_funnel.pdf]
